# Identification of KLHL12 Ligands Using Fragment-Based Methods

**DOI:** 10.1021/acs.jmedchem.5c02931

**Published:** 2026-03-30

**Authors:** Alex G. Waterson, Anish Vadukoot, Somnath Jana, Jianwen Cui, Kelvin Luong, Tyson A. Rietz, Ezequiel Alejandro Madrigal-Carrillo, Brian D. Lehmann, John L. Sensintaffar, Bin Zhao, Kangsa Amporndanai, Zoe A. Petros, William Rush Scaggs, Selena Chacon Simon, Rakesh H. Vekariya, Kwangho Kim, Manikandan Thangaraj, Plamen P. Christov, Taylor M. South, Jiqing Sai, Anusha Thiruvaipati, Charles R. Schmidt, Rebecca Eells, William J. Moore, Edward T. Olejniczak, Jason Phan, Stephen W. Fesik

**Affiliations:** † Department of Pharmacology, 12327 Vanderbilt University School of Medicine, Nashville, Tennessee 37232, United States; ‡ Department of Chemistry, Vanderbilt College of Arts and Sciences, Nashville, Tennessee 37232, United States; § Department of Biochemistry, Vanderbilt University School of Medicine, Nashville, Tennessee 37232, United States; ∥ Molecular Design and Synthesis Center, Vanderbilt Institute of Chemical Biology, Nashville, Tennessee 37232, United States; ⊥ Department of Medicine, Vanderbilt University Medical Center, Nashville, Tennessee 37232, United States; # Reaction Biology Corporation, Malvern, Pennsylvania 19355, United States; ∇ NCI Center for Cancer Research, Frederick, Maryland 21701, United States

## Abstract

Targeted protein degradation can be induced by recruiting a protein of interest to an E3 ligase, resulting in its ubiquitination and subsequent proteasome-mediated degradation. However, only a small number of E3 ligases have been utilized for degradation. Expansion of the repertoire of useful E3 ligases via the identification of ligands to those ligases could broaden the scope and applicability of the degradation paradigm. We have identified KLHL12 as an E3 ligase with higher expression in cancer over normal tissues. We report here the use of NMR-based screening to identify fragments that bind to KLHL12, and X-ray structures of a fragment hit bound to KLHL12. Using this structural information, we optimized the hits, leading to the first reported small molecules that bind to KLHL12 with submicromolar affinity. Derivatives of these compounds may be useful for the construction of PROTACs to selectively degrade protein targets in tumors while sparing normal cells.

## Graphical abstract

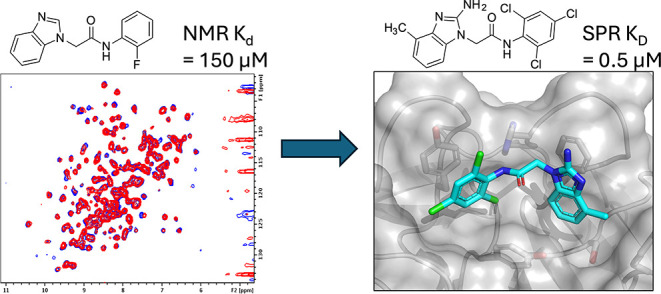

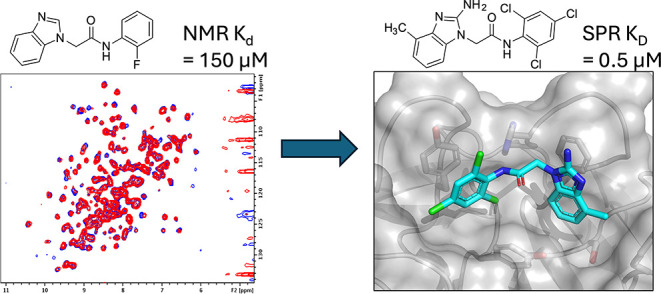

## Introduction

Ubiquitin ligases play a prominent role in the emerging field of targeted protein degradation (TPD). These enzymes can be recruited to a protein target by a molecular glue or Proteolysis Targeting Chimera (PROTAC) molecule, facilitating ubiquitination and proteasomal degradation of that target.^1^ Despite their critical importance, the TPD field remains constrained by the limited number of E3 ubiquitin ligases with known ligands that have been demonstrated to be useful for target degradation.^2^

Validating additional E3 ligases for TPD is likely to have several advantages for therapeutic discovery. For instance, PROTACs that recruit an alternative E3 ligase may broaden the range of degradable disease-associated targets or accomplish the degradation of a target protein where resistance to existing strategies has emerged.^2–7^ Moreover, E3 ligases with tissue-specific expression patterns could allow for selective degradation of target proteins in particular tissues or in specific disease states. A compelling example of this concept would be degradation of an oncoprotein in a tumor while sparing that protein in normal tissues, thus enhancing a therapeutic window by avoiding potential toxicities. This type of tissue specificity has already been demonstrated with the PROTAC DT2216,^8^ which degrades Bcl-xL via the recruitment of the E3 ligase VHL. Due to limited VHL expression in platelets, this agent effectively inhibits tumor growth while avoiding the platelet toxicity associated with Bcl-xL inhibition, which has led to clinically significant thrombocytopenia.^9^

We have previously reported an analysis of the mRNA expression profiles of E3 ligases.^10^ In that study, we determined that some ligases are overexpressed at both the mRNA and protein levels in cancer cells compared to their corresponding expression in normal tissues and demonstrated that NMR-based fragment screening is a useful method for identifying fragments that bind to such ligases.

In this report, we extend that work to Kelch-like protein 12 (KLHL12), showing that this ligase is overexpressed in cancer relative to normal tissues. Using a protein-observed NMR-based fragment screening approach, we identified hits that bind to KLHL12 and determined the X-ray structures of these hits bound to the protein. Leveraging this structural information, we discovered potent small molecules that bind to KLHL12 that may be suitable for the synthesis of PROTACs.

## Results

### Expression Profile of KLHL12

We previously conducted a large-scale analysis of publicly available mRNA data and found that E3 ligases exhibit varied levels of expression across a wide selection of normal and cancer tissues.^10^ Certain ligases show preferential expression in normal tissues, while others are overexpressed in cancers. In the earlier work, we identified CBL-c and TRAF-4 as ligases with enriched transcripts in cancer and validated these differences at the protein level.^10^ Using the same compiled mRNA data set described in that work, we also found that the E3 ligase KLHL12 is more highly expressed in cancer relative to normal tissues (Figures 1A, and S1).

**Figure 1:**
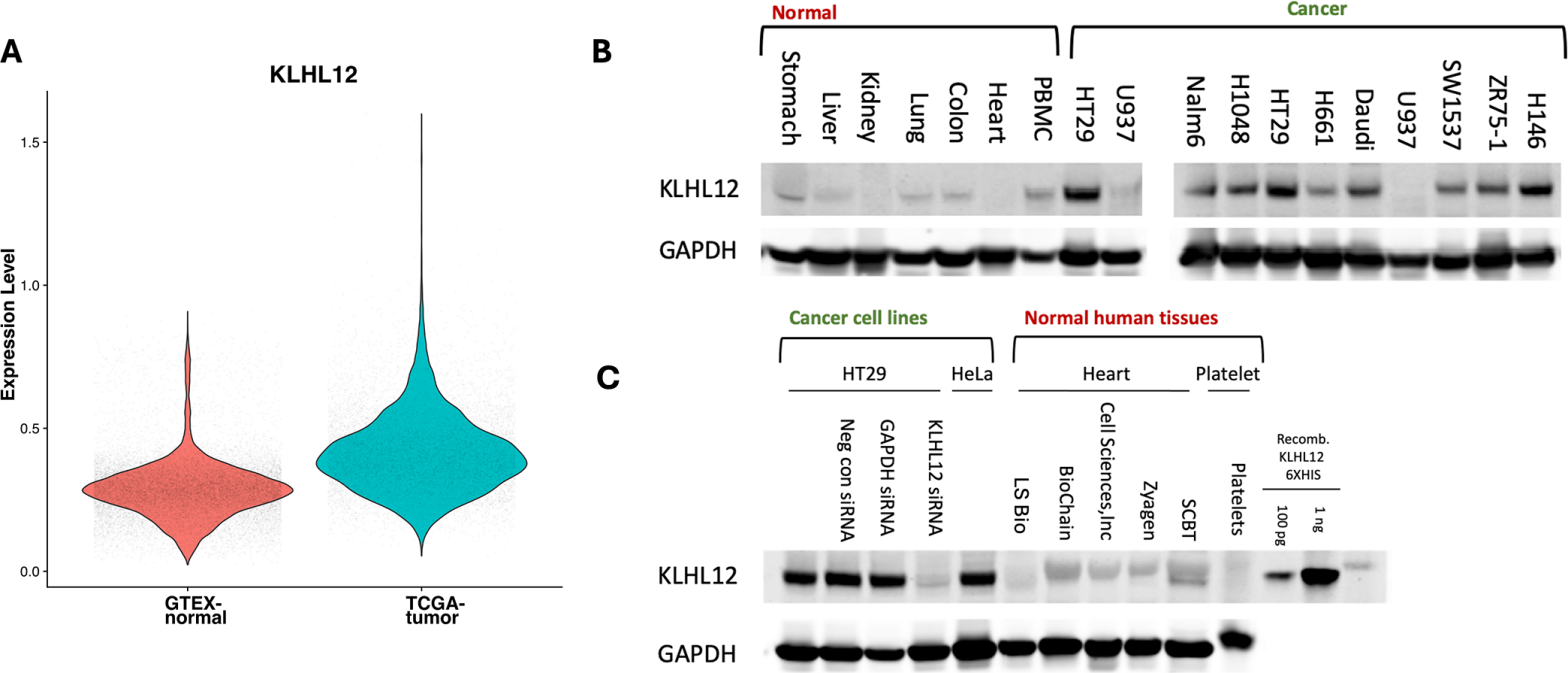
KLHL12 expression profile. (A) Violin plot demonstrates differential KLHL12 transcript expression in cancer (TCGA) versus normal tissues (GTEx). (B) Immunoblot shows KLHL12 protein levels across tumor specimens and cell lines. (C) Immunoblot shows KLHL12 protein levels across commercial heart and platelet tissue samples. Antibody specificity is demonstrated with immunoblots in HT29 cells transfected with control siRNA or an siRNA targeting KLHL12 and with detection of recombinant protein.

Proteins containing Kelch domains are widespread throughout the proteome and contain a characteristic set of 5–7 Kelch motif “blades” arranged into a β-propeller structure^11^ that often mediate protein–protein interactions. A subset of these proteins, known as the Kelch-like (KLHL) family, also contain bric-a-brac, tramtrack, broad complex (BTB) domains, and a BACK domain.^12^ Many of these KLHL family members possess ubiquitin ligase activity, which regulates diverse biological functions through substrate-specific ubiquitination.^13^ Given their structural features and functional roles, the KLHL family represent promising candidate ligases for TPD. Ligands to KEAP1 (also known as KLHL19) have been used to construct PROTACs that degrade BRD4 ^14^ and KEAP1-based PROTACs have been systematically compared to degraders of multiple target proteins that recruit more commonly used ligases.^15^ Additionally, a macrocyclic ligand derived from a DAPK1 peptide fragment was shown to bind to KLHL20 and a conjugate of that peptide with a ligand to BRD4 has been shown to degrade BET proteins.^16^ Further, a large study designed to assess the ability of various proteins to affect protein stability demonstrated that several KLHL family members, including KLHL6, KLHL12, and KLHL13, among others, have strong potential for use in targeted protein degradation.^3^

Motivated by KLHL12′s differential transcript expression and high potential utility in TPD, we investigated the protein expression profile by immunoblotting protein lysates obtained from a small panel of cancer cells and tumor samples (Figure 1B). Our data show robust KLHL12 protein levels in most cancer cell lines, with comparatively low levels in most normal tissues, including negligible expression in heart tissues. To further validate these findings, we examined KLHL12 expression levels in platelets and additional heart tissue samples and used recombinant KLHL12 protein and siRNA-mediated knockdown of KLHL12 in HT29 cells to demonstrate antibody specificity (Figure 1C). Overall, cancer cell lines display higher KLHL12 protein expression compared to normal tissue, with these differences generally greater than those observed in the transcriptomic analysis. Together, these findings suggest that PROTACs that recruit KLHL12 may enable cancer-specific degradation of a target protein.

### Fragment Screen

To utilize KLHL12 for protein degradation, small-molecule ligands that bind to the protein must be discovered. However, outside of KEAP1/KLHL19, small molecule ligands for most Kelch-like proteins remain undescribed. To discover ligands for KLHL12, we used NMR-based fragment screening, a powerful method that is well-suited for the identification of ligands to challenging target proteins.^17^ We have previously described the expression of uniformly ^15^N labeled KLHL12 Kelch domain, have shown that it displays a well-resolved ^1^H–^15^N SOFAST-HMQC spectrum,^18^ and analyzed the binding of peptides based on the native substrate proteins D4.2 and Dvl1/Dsh3 ^19,20^ using both NMR and X-ray crystallography to unambiguously establish NMR shift patterns associated with binding in the substrate pocket of the KLHL12 Kelch domain.

Using a ^1^H–^15^N correlation NMR experiment of uniformly labeled ^15^N KLHL12 Kelch domain, we performed a screen of our internal library of 13,824 fragments arrayed in 12-compound mixtures. After deconvolution of the fragment mixtures, we identified 35 compounds that showed significant shifts in the NMR spectra. Of these, 15 fragments displayed strong shifts that match a portion of the shifts displayed by the peptide substrate, suggesting binding in the corresponding pocket (Figure 2A). To determine the binding affinities of the hits, we assessed the shift magnitude resulting from increasing concentrations of the compounds. The strongest binding fragment hit was benzimidazole **1** (Figure 2B), with an NMR-determined affinity of 150 μM (LE = 0.27). This compound represented one of the major structural clusters found in the hit set. We selected and examined the KLHL12 binding of additional structurally related molecules from our internal compound collection and commercially available close structural analogs. This approach identified additional compounds (**2–6**) that bound to KLHL12 and revealed preliminary fragment-level structure–activity relationships (SAR) that could guide affinity improvements. For example, removal of a benzimidazole nitrogen led to indole **2** with reduced affinity, and particularly important early SAR trends were identified around the aniline portion of the hit compounds. Substituting the fluorine of **1** with a chlorine (**3**) generated a 2-fold increase in affinity, and adding a second fluorine at the other *ortho* position of the ring produced compound **4** which showed tighter KLHL12 binding. Titration of the KLHL12 NMR shifts induced by **4** revealed that it binds below the useful limits of the NMR technique, with some concentrations not in fast exchange on the NMR time scale. Thus, we estimate the affinity to be at or below 35 μM. A *para*-substitution appeared to be tolerated (**5**) and this substitution improved affinity when combined with the *ortho* chlorine (compare **3** and **6**, another analog that appears to bind below the limits of accurate affinity determination by NMR).

**Figure 2:**
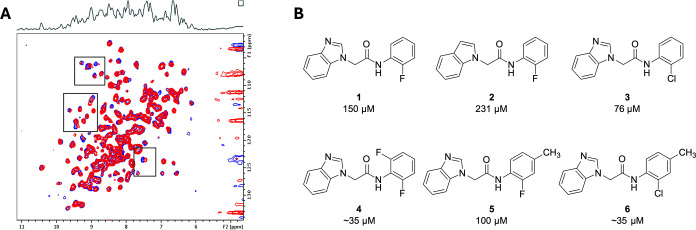
Fragment screening identified hits that bind to KLHL12. (A) SOFAST-HMQC spectrum of KLHL12 in the presence (blue) and absence (red) of fragment hit **1**. Boxed regions highlight specific shift perturbations induced by the fragment. (B) Structure of **1** with NMR-derived affinity as well as closely related hits (**2**–**6**) identified in the screen.

To clarify the binding mode of the benzimidazole hit series, we cocrystallized **1** with the Kelch domain of KLHL12. The electron density of the bound compound, shown in Figure 3A, unambiguously showed binding in the substrate cleft of KLHL12. However, the low resolution of 2.7Å obtained upon refinement of the structure showed that two different binding conformations were possible (Figure 3A). Conformation A (Figure 3B) is stabilized by a likely edge-on π-stack between the aniline ring and Tyr434 and a possible weak face-to-face interaction with Tyr528. Conformation B (Figure 3C) is stabilized by similar interactions, but with opposite ends of the molecule. The benzimidazole core of the compound may also have a π stacking interaction with Phe481 in this conformation.

**Figure 3:**
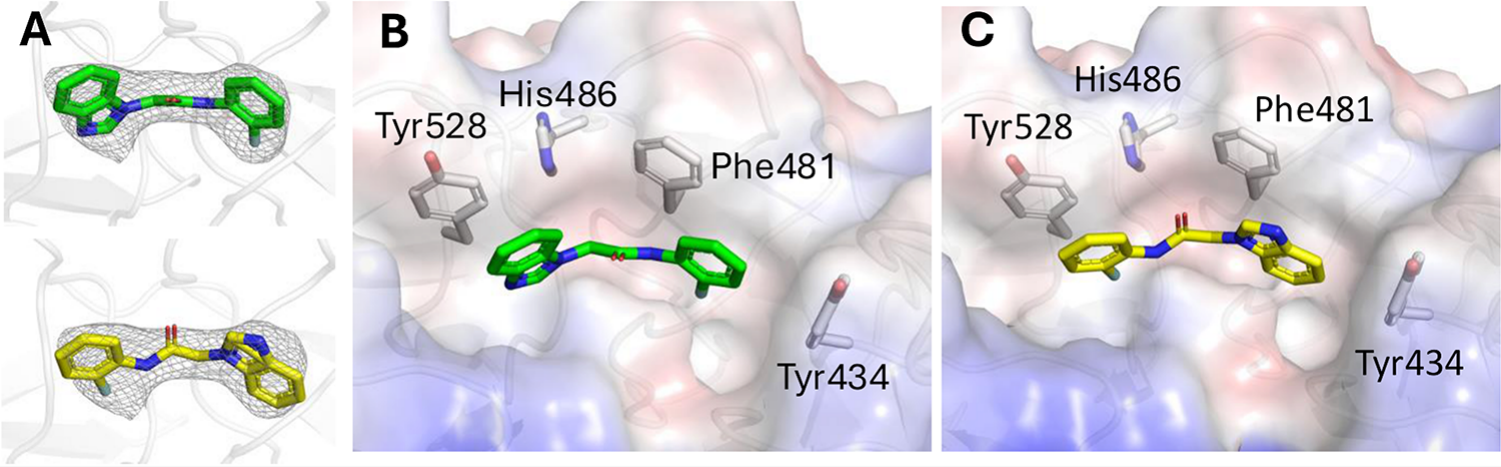
X-ray structure of **1** bound to KLHL12 (9Y8J). (A) Observed electron density. (B) Possible conformation A. (C) Possible conformation B.

### Hit Optimization of Benzimidazole Series

Because of the intermediate exchange characteristics shown by compounds such as **4** and **6**, assessing further affinity advancements requires an assay that can quantitatively assess tighter binding to KLHL12 than possible with NMR. We thus employed a competition-based fluorescence-polarization anisotropy (FPA) assay^21^ using the reported modified Dvl-3 peptide, which has a reported 9 μM affinity to KLHL12.^18^ As shown in Table 1, **6** binds to KLHL12 with an FPA-determined K_i_ of 24.2 μM, consistent with the intermediate exchange observed in the NMR.

**Table 1:**
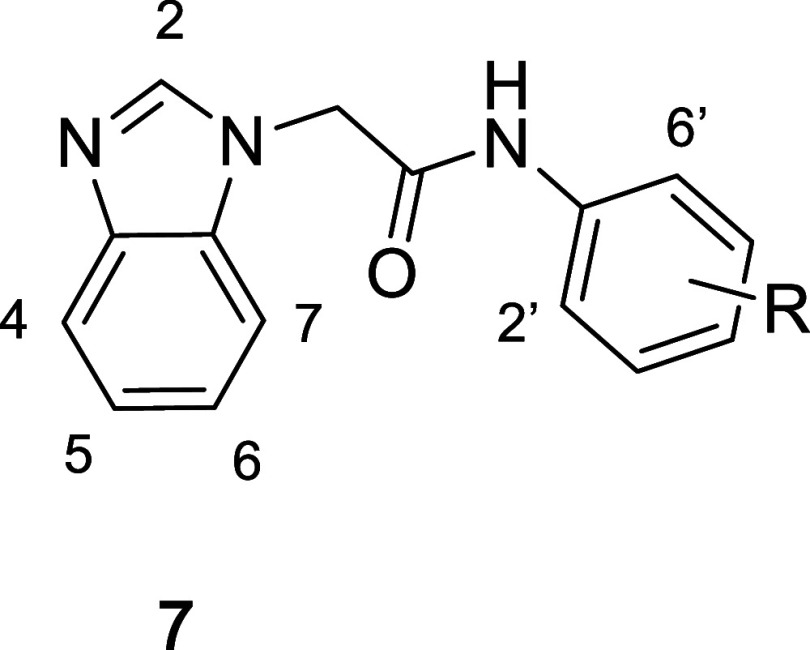
Initial SAR in the Benzimidazole Template

Cmpd	benzimidazole	R	FPA^a^ *K_i_* (μM)
**6**	–H	2′-chloro, 4′-methyl	24.2
**7a**	–H	2′-chloro, 3′,4′-dimethyl	9.9
**7b**	–H	2′,6′-dichloro, 4′-methyl	4.4
**7c**	–H	2′,4′,6′-trichloro	5.9
**7d**	2-methyl	2′,4′,6′-trichloro	9.0
**7e**	2-amino	2′,4′,6′-trichloro	6.5
**7f**	2-dimethylamino	2′,4′,6′-trichloro	4.3
**7g**	2-acetamide	2′,4′,6′-trichloro	3.6
**7h**	2-methylamino	2′,4′,6′-trichloro	15.3
**7i**	2-carboxamide	2′,4′,6′-trichloro	37.8
**7j**	2,4-dimethyl	2′,4′,6′-trichloro	6.5
**7k**	2-amino, 4-methyl	2′,4′,6′-trichloro	0.33
**7l**	2-amino, 4-cyclopropyl	2′,4′,6′-trichloro	3.5^b^

a Average *K_i_* values determined from competition FPA assay using a labeled peptide-derived probe and a minimum of 2 experimental replicates.

b *K_i_* value determined from competition FPA assay using a labeled small molecule probe (**11a**, see Figure 8 and Scheme 8), which has a directly determined KLHL12 affinity of 1.2 μM (Figure 8) and an SPR-determined affinity of 3.6 μM (Table S1).

To capitalize on the potential of KLHL12 to induce TPD, we sought to identify a ligand with superior affinity from which to synthesize PROTACs. We initially targeted 100–200 nM affinity to KLHL12, consistent with ligands that recruit commonly used E3 ligases.^7^ Using **6** as a basis, we first sought these affinity gains by manipulation of the aniline ring substitutions. Adding a 3′-position methyl (**7a**) improved the FPA-determined affinity to around 10 μM. For several analogs, we also assessed binding via an orthogonal biophysical method, surface plasmon resonance (SPR), using His-tagged KLHL12 (see Table S1). The SPR affinity of **7a**, at 19.1 μM, was within 2-fold of the FPA affinity. Inspired by the dual fluorine atoms of **4**, we added a second chlorine leading to anilines with substitutions at the 2′-, 4′-, and 6′-positions (**7b** and **7c**), which exhibited affinities under 10 μM. The tighter binding of the compounds with bis *ortho* substitutions may result from an altered ground state preference, favoring an orthogonal presentation of the aromatic ring with respect to the amide bond, or from the higher lipophilicity imparted by these substitutions increasing hydrophobic interactions with the protein.

Having established some basic SAR preferences around the aniline ring, we turned our attention to the benzimidazole core of the template. Several substitutions were placed at the 2-position of the benzimidazole (**7d**–**7i**), and many of these compounds show similar affinities. Compounds with an amine at the 2-postion (**7e**–**g**) were generally slightly superior to the 2-methyl **7d**. Extending this amine from the ring with a methylene (**7h**) or as a carboxamide (**7i**) showed reduced binding, consistent with the better affinity of **7e** relative to **7d**. Overall, the general tolerance for substitution at this position suggested that this area of the molecule was solvent-exposed, matching with “conformation B” in the original X-ray structure (Figure 3C). Based on this hypothesis, we decided to explore the phenyl portion of the benzimidazole core. Since this region of the molecule likely sits deeper in the binding pocket, we anticipated sharp SAR preferences. Indeed, the addition of a methyl group to the 4-position of the benzimidazole generated compounds **7j** and **7k**, which both displayed higher affinity than their 4-H comparators **7d** and **7e**. The affinity of both analogs **7j** and **7k** were verified by SPR (Table S1); **7k** was confirmed as a submicromolar binder, with **7j** showing an SPR-derived affinity 3-fold worse than **7k**. Few additional alkyl substitutions were tolerated at the 4-position; one of the tolerated moieties was the 4-cyclopropyl group of **7l**, which lost 10-fold affinity compared to **7k**, but was approximately 2-fold better than **7e**.

The X-ray structure of **7k** bound to KLHL12 was solved at 1.3 Å resolution (Figure 4A), allowing more precise determination of the compound binding mode. In keeping with the SAR, the compound was found to bind in a conformation similar to “B” in Figure 3C. The 4-methyl group makes an apparent CH-π interaction with Tyr334, likely explaining the higher affinity of this molecule. The costructure with **7k** also demonstrates that these molecules have multiple solvent-exposed positions that may be suitable for later PROTAC creation from an analog with the desired affinity level (100–200 nM). For example, the 2-amino group of **7k** is oriented directly into the solvent front and may tolerate modifications based on the SAR shown in Table 1. The orientation of the aniline ring also suggests at least two exit vector options for linking– one by replacement of a Cl atom and the other at the *meta* position between the 2′- and 4′-chlorines.

**Figure 4:**
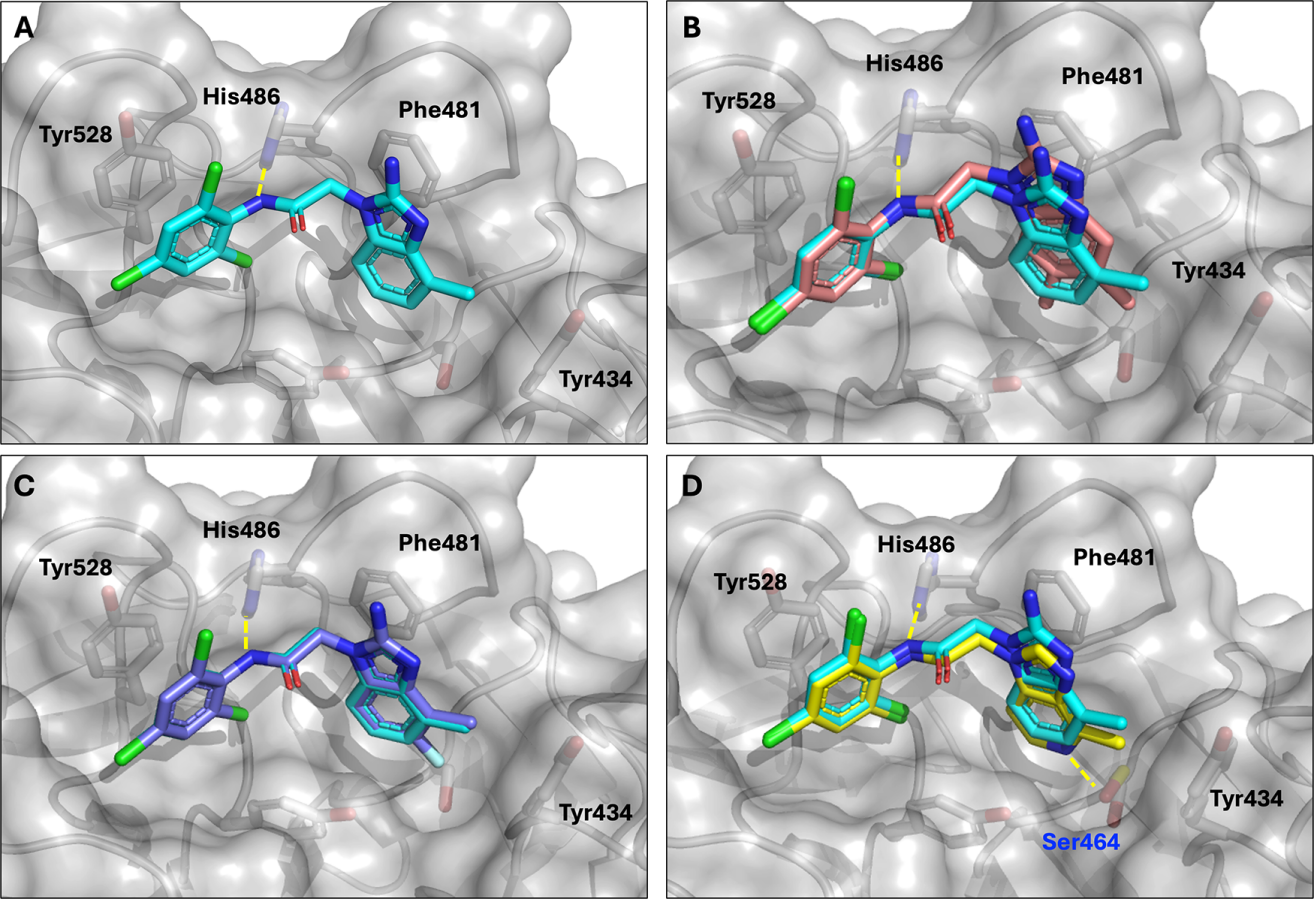
(A) X-ray structure of **7k** bound to KLHL12 (9Y8K). Overlays of **7k** with the bound structures of (B) **8e**, 9Y8L, (C), **8i**, 9Y8M, and (D), **8m**, 9Y8N.

To identify tighter-binding analogs useful for PROTAC synthesis, we explored additional modifications of **7k**. Analysis of the structure of **7k** bound to KLHL12 revealed additional unoccupied space below the benzimidazole ring. Further, the side chain of Ser464 offered the possibility to build in a hydrogen bonding interaction between the protein and the small molecule. To further explore this space, we conducted a more complete survey of substitutions on the benzimidazole (Table 2). Both methoxy and methyl substitutions at the 6- and 7-positions were relatively well-tolerated but displayed flat SAR in the FPA binding assay. Surprisingly, combining the 5- and 6-methyls produced **8e** that showed affinity just under 1 μM. Single halogen substitutions (**8f** and **8g**) showed affinity that roughly matched the corresponding methyl groups (**8d** and **8c** respectively), but the difluoro combination **8h** displayed a significant drop in affinity. Despite the additional space in the pocket evident in the crystal structure and the relative tolerance for small substitutions around the ring, combining additional substitutions with the 4-methyl of **7k** gave compounds such as **8i**–**k** that showed reduced affinity compared to **7k**. An overlay of the X-ray structure of **8e** with **7k** bound to KLHL12 reveals that the substitutions along the bottom of the benzimidazole, particularly at the 7-position, can shift the binding mode of the heterocycle, perhaps explaining the unanticipated lack of affinity improvements and the lack of SAR additivity. Smaller moieties at the 5-position, however, should allow for retention of the original binding mode; this was indeed the case for **8i** (Figure 4C).

**Table 2:**
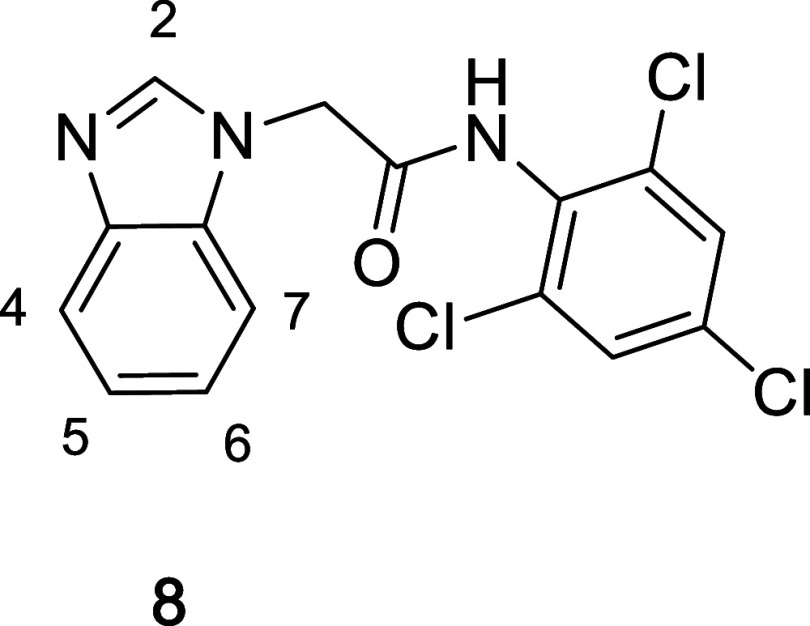
Benzimidazole Substitution SAR

Cmpd	benzimidazole	FPA^a^ *K_i_* (μM)
**8a**	2-amino, 6-methoxy	3.0
**8b**	2-amino, 5-methoxy	4.8
**8c**	2-amino, 6-methyl	7.8
**8d**	2-amino, 5-methyl	5.4
**8e**	2-amino, 5,6-dimethyl	0.98
**8f**	2-amino, 5-fluoro	2.8
**8g**	2-amino, 6-chloro	8.4
**8h**	2-amino, 5,6-difluoro	17.1
**8i**	2-amino, 4-methyl, 5-fluoro	38.4
**8j**	2-amino, 4,5-dimethyl	6.7
**8k**	2-amino, 4,6-dimethyl	4.6
**8l**	2-amino, 7-(N)	20.5
**8m**	4-methyl, 5-(N)	5.2
**8n**	4,6-dimethyl, 5-(N)	10.5

a Average *K_i_* values determined from competition FPA assay using a labeled peptide-derived probe and a minimum of 2 experimental replicates.

Nitrogen atoms in the ring were also explored. The addition of a nitrogen at the 7-position (**8l**) was unfavorable. Inclusion of the heteroatom at the 5-position, in combination with the 4-methyl of **7k** produced **8m**, which showed affinity of around 5 μM even without the 2-amino substitution. The addition of a 6-methyl further reduced affinity by 2-fold. As shown in Figure 4D, the X-ray structure of **8m** bound to KLHL12 showed that, in contrast to the above analogs, the benzimidazole sits slightly deeper in the binding pocket. Further, while most compound-bound X-rays show minimal movement of residue side chains, this costructure showed a small movement of Ser464 toward the compound, which brings the nitrogen of the molecule and the oxygen of the amino acid within 2.8 Å, indicating a likely hydrogen bond.

### Occupying the Cleft

SAR investigations in the pocket below and around the benzimidazole failed to significantly improve the affinity of **7k**. Thus, additional opportunities to increase binding affinity by filling in unused space in the pocket and/or building in interactions between the small molecule and the protein were sought. One such opportunity is represented by unoccupied space beyond Tyr528. The 4′-position chlorine in compounds such as **7k** is located just beyond the side chain of this amino acid, at the entry point of a narrow, and open, “cleft” in the protein. Thus, the 4′-position was selected as a logical point from which to extend the molecules into this region. Table 3 shows several of the compounds that we prepared and evaluated to obtain increased occupation of the cleft region.

**Table 3:**
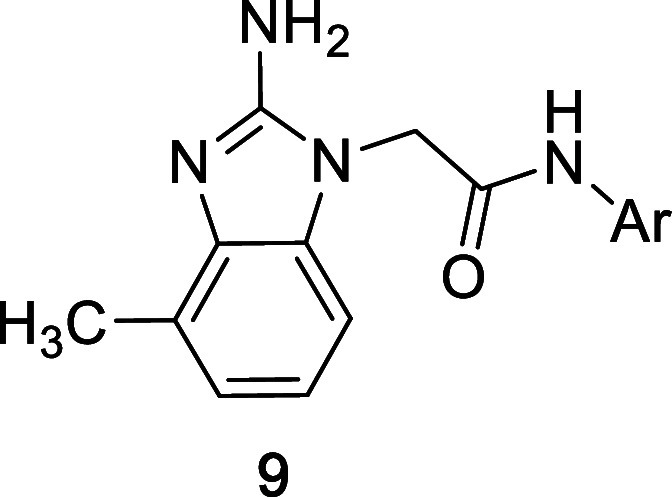

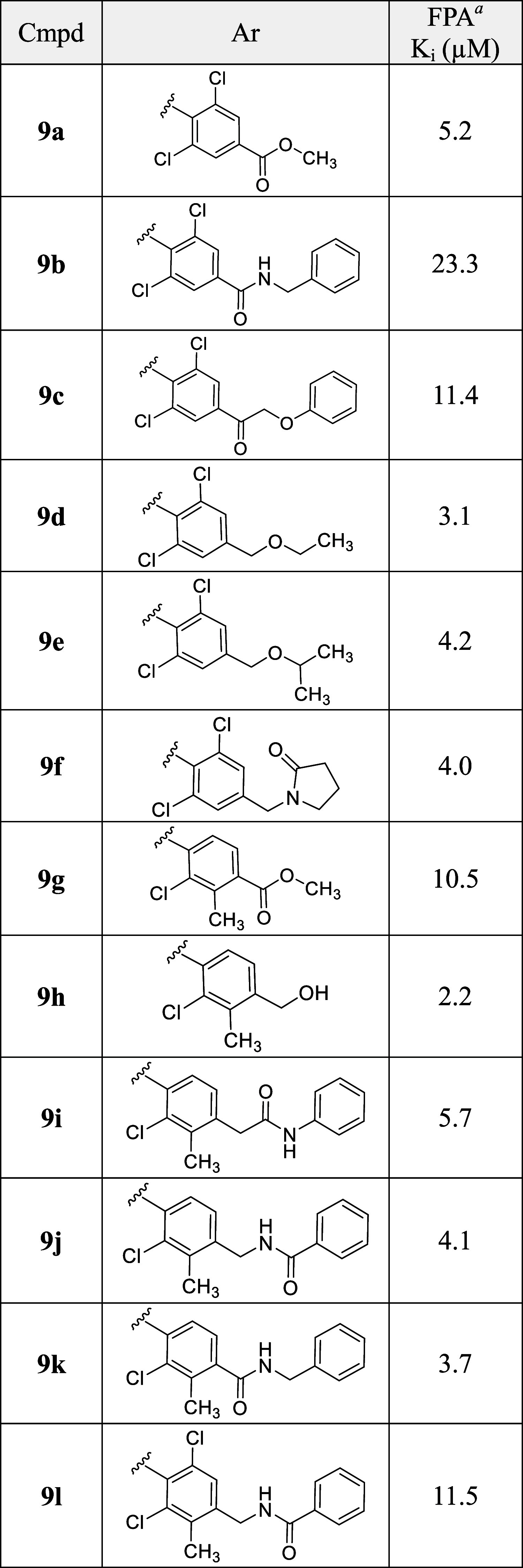
Aniline Extensions to Explore the Cleft

a Average *K_i_* values determined from competition FPA assay using a labeled peptide-derived probe and a minimum of 2 experimental replicates.

The ester moiety of **9a** showed a reduction in binding affinity in the FPA assay compared to **7k**. Further extensions of carbonyl-based moieties, such as the amide of **9b** and the ether-bearing ketone of **9c**, further eroded affinity. As shown in Figure 5A, the X-ray structure of **9c** bound to KLHL12 shows that the compound retains the binding mode of **7k**, and the extensions from the aniline extend down the cleft, as designed. Residues Phe289 and Gln293 are shifted with respect to their positions found in compounds with a 4-chlorine on the aniline. While the 4′ extension of **9c** occupies the cleft, the geometric arrangement of these compounds features suboptimal dihedral angles, likely inducing an energetic penalty that results in the lack of affinity improvement. We thus sought to remove the 4′-position carbonyl and explore this cleft with a more flexible 4′-position extension. Ethers **9d** and **9e** as well as amide **9f** extend from a 4′-methylene on the aniline ring. While binding better than **9b** and **9c**, these compounds show affinity that is no better than **9a** by both FPA assay and SPR (Table S1) and remain reduced in binding affinity compared with **7k**. The X-ray structure of **9e** bound to KLHL12 shows a binding mode very similar to **9c** (Figure 5B), with similar side chain positions for Gln293 and Phe289.

**Figure 5:**
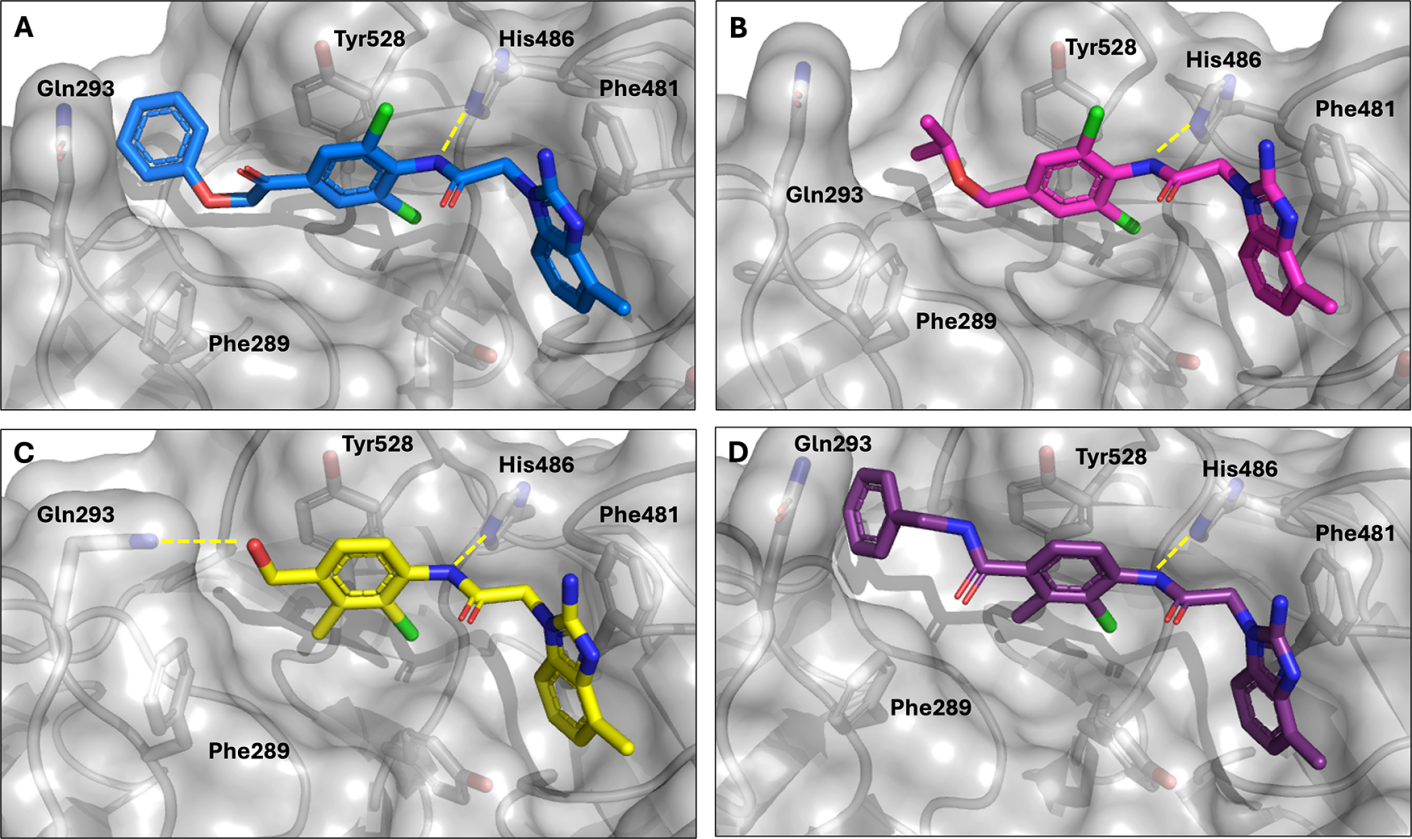
X-ray structures of cleft-targeted molecules bound to KLHL12. (A) **9c**, 9Y8O, (B) **9e**, 9Y8Q, (C) **9h**, 9Y8R, and (D) **9k**, 9Y8S.

Based on these X-ray costructures and the lack of affinity improvement with cleft occupation, we elected to attempt to reposition the cleft-facing extensions by utilizing a different aniline arrangement. Inclusion of the 3′-position methyl on the ester (**9g**) did not increase affinity relative to **9a**, but the hydroxyl-containing **9h** displayed ∼2 μM binding to KLHL12 by both FPA and SPR (Table S1). Analysis of the structure of **9h** bound to KLHL12 (Figure 5C) shows that the 3′-methyl raised the aniline ring slightly and that the hydroxyl group is, as expected, oriented at the entry way to the cleft region. In this case, Gln293 has moved to accommodate a likely hydrogen bond with the hydroxyl. Again, however, several variations on chemical moieties replacing this hydroxyl moiety with substitutions that might occupy the cleft and generate improvements in affinity failed to deliver on that goal (**9i**–**9k**) and placing the second *ortho* chlorine back onto the aniline ring in **9l** further eroded binding. As shown in Figure 5D, the reoriented substitution on **9k** that was intended to reach the cleft did, in fact, do so. In summary, no affinity improvements have been noted with any compound that explored this area of the pocket, even those for which the X-ray structures definitively show that the compounds fill this space.

### Occupying the Proline Pocket

As occupation of the cleft area failed to improve affinity, we turned to other unused space available in the small molecule binding pocket. Examining the structure of **7k** bound to the protein (Figure 4A), we observed that additional space was also present beyond Tyr434 and that this space is occupied by a proline residue in the previously described peptide-bound structure.^18^ Accessing this pocket was expected to present a challenge, as the space is roughly orthogonal to the plane of the benzimidazole ring. The benzimidazole 4-position methyl group was chosen as a likely pivot point from which to begin exploration of the proline subpocket. However, based on the limited tolerance for alkyl substitutions at this position, we anticipated narrow SAR. Thus, a small selection of cyclic amines was extended from this methyl to produce guiding SAR. As shown in Table 4, the piperazine **10a** was not tolerated, but the morpholine **10b** represented a more attractive option. The X-ray structure of **10b** bound to KLHL12 (Figure 6A) showed that the morpholine was oriented in the appropriate direction, but did not penetrate deeply into the subpocket; instead, it remained above the pocket, in the vicinity of Arg392.

**Figure 6:**
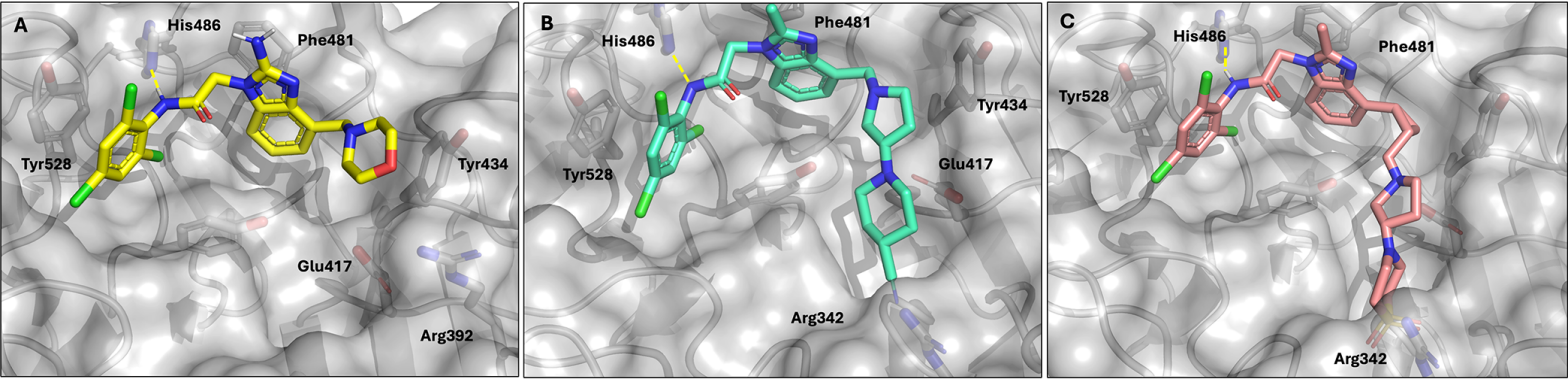
X-ray structures of (A) **10b**, 9Y8T, (B) **10j**, 9Y8U, and (C) **10q**, 9Y8V, bound to KLHL12.

**Table 4:**
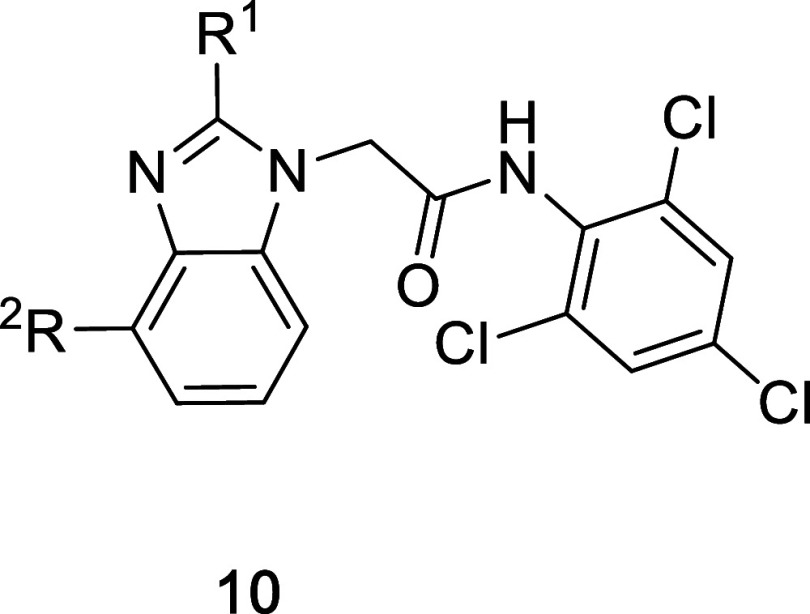

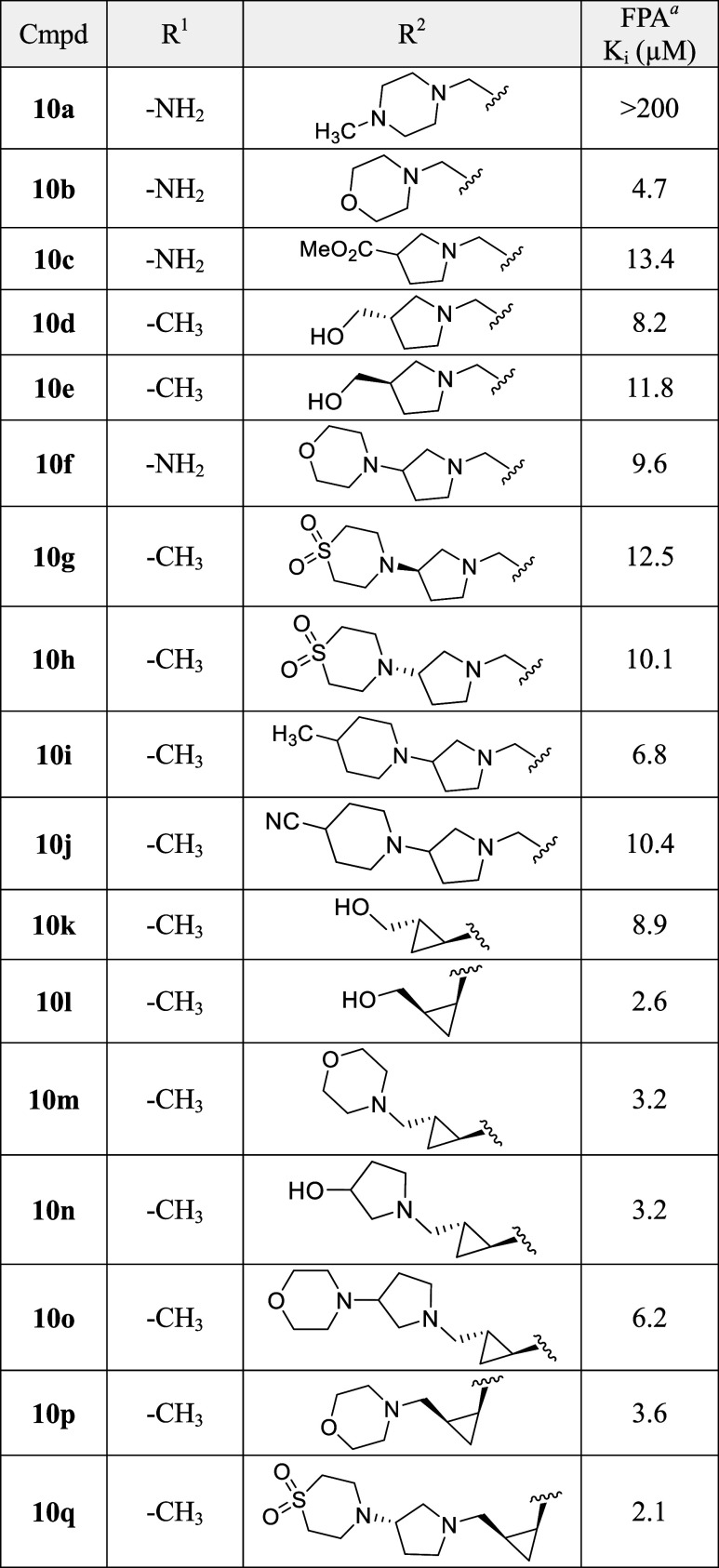
Benzimidazole Extensions to Access the Proline Pocket Region

a Average *K_i_* values determined from competition FPA assay using a labeled small molecule probe (**11a**, see Scheme S13) and a minimum of 2 experimental replicates.

It was evident that further extensions from the ring would be required to bind more deeply into the pocket. Substituted pyrrolidines **10c**–**10j** were prepared using a selection of small, polar functionality (e.g., alcohols **10d,e**) and ring systems with an array of substitutions. In many cases, we were unable to access the desired analogs with an amine at the benzimidazole 2-position, so a 2-methyl was used instead to gauge the impact of the changes. While most of these analogs were predicted by molecular modeling to interact with residues in and around the pocket (not shown), the SAR shown by these analogs was largely flat, with affinity for each molecule hovering near 10 μM. We obtained an X-ray structure of **10j** bound to KLHL12. As shown in Figure 6B, a single enantiomer of the racemic compound crystallized with the protein. The cyano group is observed to extend into the unoccupied space, likely interacting with Arg417. The bound conformation is also likely stabilized by a charge interaction between the piperidine nitrogen and Glu417.

While providing proof of principle that this subpocket can be accessed, we hypothesized that tighter binding compounds could be obtained if we could increase conformational rigidity while maintaining the positive interactions with Tyr434 within a small space. Molecular modeling (not shown) determined that the cyclopropane of **7l** may offer suitably constrained vectors for further elaboration. While **7l** does not equal the affinity of **7k**, the 3.5 μM affinity was deemed suitable for continuing the push into the unused space. Again, elaboration of the cyclopropane proved difficult with the 2-aminobenzimidazole, so a 2-methylbenzimidazole (e.g., **7j**) was used as a basis for the SAR exploration. *cis* and *trans* isomers **10k** and **10l** were prepared, each with a potentially functionalizable hydroxyl group. The *cis* isomer **10l** was found to bind ∼3-fold better than the *trans* isomer **10k**, showing a small improvement over **7j**, but not matching the affinity of **7k**. We prepared extensions from the cyclopropane of each isomer to determine whether we could fill the subpocket and drive an affinity improvement. Amine rings in the *trans* configuration (**10m**–**o**) could show a small improvement over **10k**, with both **10m** and **10n** displaying ∼3 μM affinity by FPA and ∼2 μM affinity by SPR (Table S1). Both the *cis*-isomer-derived morpholine **10p** and oxo-thiomorpholine of **10q** bound to KLHL12 with similar affinities as **10l** by FPA, but surprisingly showed SPR-derived affinities much better than these values (Table S1). Indeed, at ∼0.3 μM, both compounds show SPR affinity roughly matching, or perhaps slightly exceeding, the binding of **7k**. The reason for the increased binding shown by SPR relative to the FPA data is unclear. While it is possible that the compounds may display altered binding kinetics by virtue of additional interactions in the pocket, the SPR binding kinetics for **10p** and **10q** are not significantly different compared to those shown by **7k** (Table S1). The X-ray structure of **10q** bound to the protein (Figure 6C) showed that the sulfonyl group of the oxothiomorpholine was, like the cyano of **10j**, placed deeply into the pocket, engaging in a likely hydrogen bond with Arg342. Also similar to **10j**, the extension was anchored in the pocket via a charge interaction with Glu417. Interestingly, while **10j** and **10q** are stereochemical mixtures, a single stereoisomer of each crystallized in the active site, suggesting that a purified analog might have increased affinity.

### Kelch Domain Selectivity

The KLHL proteins are a large family, with each member containing a Kelch motif with a conserved β-propeller structure.^11,12^ The KLHL12 ligands described above bind to the KLHL12 Kelch domain and, due to this conserved protein motif, might also bind other family members. To gauge the potential for this off-target binding, we first evaluated homology within the family to determine which of the KLHL family members were closest in structure to KLHL12.

We acquired protein sequence data of the KLHL proteins from the UniProt database using accession codes listed in Supporting Table S3. The sequences of the Kelch domain of the KLHL proteins were aligned by Clustal Omega 2.1 ^22^ and ranked by sequence identity percentage to KLHL12 (Supporting Table S4). Ten of these KLHL family proteins share sequence identity above 35% and are thus considered highly homologous with KLHL12. Some of these proteins have reported structures, including KLHL19 (KEAP1)^23^ and KLHL20.^24^ For those that did not have available crystal or electron microscopy structures, we generated homology models using AlphaFold 2.0 ^25^ and overlaid these models to the KLHL12-**7k** crystal structure along with available X-ray structures (Figure 7).

**Figure 7:**
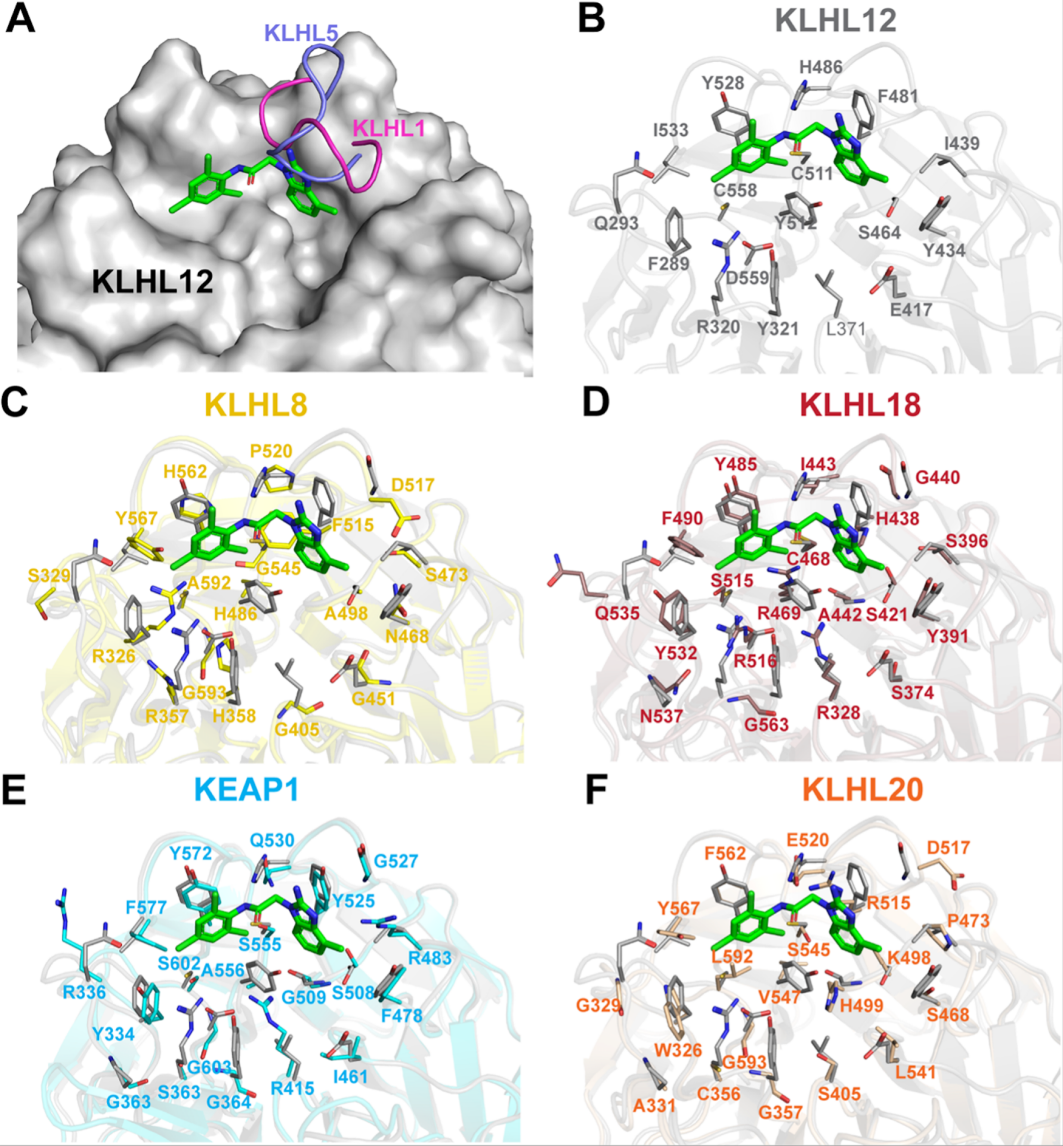
Structural analysis of KLHL12 and other KLHL proteins. (A) An extending loop in KLHL1 (magenta) and KLHL5 (purple) may disrupt binding of KLHL12 ligands. (B) Crystal structures of KLHL12 with **7k** (9Y8K, green sticks) labeled with residue names and numbers. Overlaid structures of highly conserved KLHL proteins (C) KLHL8 (Alphafold prediction), (D) KLHL18 (Alphafold prediction), (E) KLHL19 (KEAP1, 1ZGK, teal), and (F) KLHL20 (6GY5, orange).

From this analysis, we observed a loop that can potentially shield the ligand binding pocket in KLHL1 and KLHL5 (Figure 7A). Due to this loop, KLHL12 ligands are unlikely to show strong affinity for KLHL1 and KLHL5. The other eight higher homology KLHL proteins were further examined by aligning only the ligand binding pocket and analyzing sequence identity and similarity to KLHL12. We found that KLHL8, KLHL18, and KLHL19 (KEAP1) show the highest sequence identity in the binding pocket, with KLHL18 and KEAP1 showing >50% similarity. This suggests that KLHL18 and KEAP1 represent the most likely proteins to which KLHL12 ligands might bind.

Since KLHL19/KEAP1 represents a protein with high Kelch domain homology to KLHL12 and has been validated for use in TPD,^14^ we sought to determine if binding to KEAP1 was a significant possibility with molecules in the benzimidazole series. We assessed the binding FPA probe **11a** (Scheme 8), to both KLHL12 and KEAP1 using fluorescence polarization anisotropy.^21^ The saturation binding curve for **11a** in the presence of increasing concentrations of KLHL12 shows an affinity of 1.2 μM (Figure 8), whereas saturation was not reached with KEAP1, indicating weak binding. Per our homology analysis (Figure 7), this weak binding may be attributable to the lack of the key interaction of the ligand with KLHL12 His486. In KEAP1, this residue is Gln530, which will alter the H-bonding interaction with the ligand. Other high-homology family members, including KLHL18, failed to express in *Escherichia coli*; we were thus unable to determine binding to these family members by this method. The promising specificity of binding to KLHL12 over the closely related KEAP1/KLHL19 suggests that compounds in this series may bind selectively to KLHL12 compared with other Kelch domain family members. However, full assessment of the binding specificity of additional analogs across the family remains unexplored.

**Figure 8:**
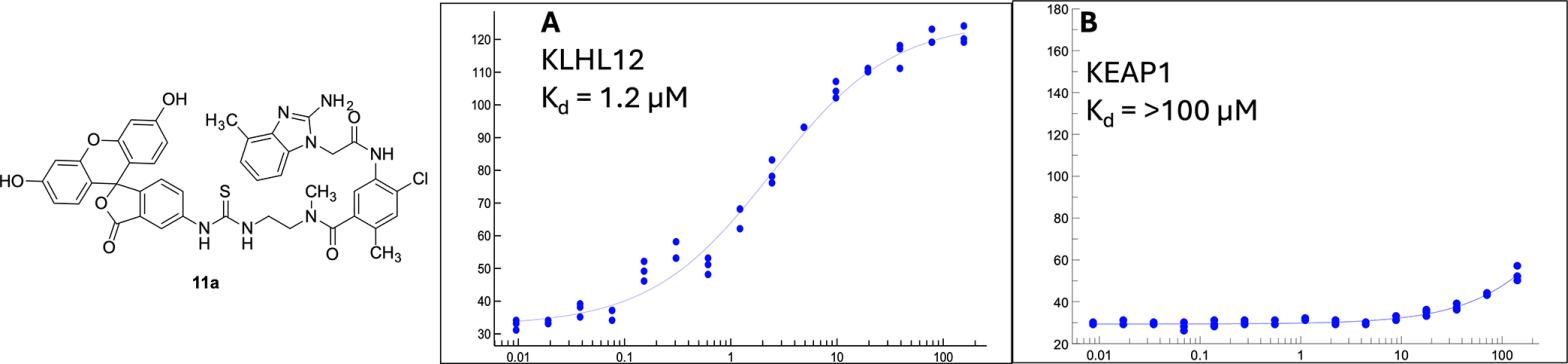
Saturation binding studies with FPA probe **11a** shows selective binding for KLHL12 (A) over KLHL19/KEAP1 (B).

### Cellular Target Engagement

To assess the ability of the KLHL12 ligands to bind to the protein in a cellular context, we developed a nanoBRET-format assay using HEK293 cells transfected with a KLHL12-NanoLuc fusion construct. Competition with labeled tracer **11b** (see Scheme 8) over a dose range produces an IC_50_ that is correlated with cellular target engagement. We performed this assay at a short time point in cells in which the membrane had been permeabilized with the digitonin treatment (Figure 9A) and in a longer, 2h time point in intact cells (Figure 9B). IC_50_ values determined from these experiments is shown in Table 5.

**Figure 9:**
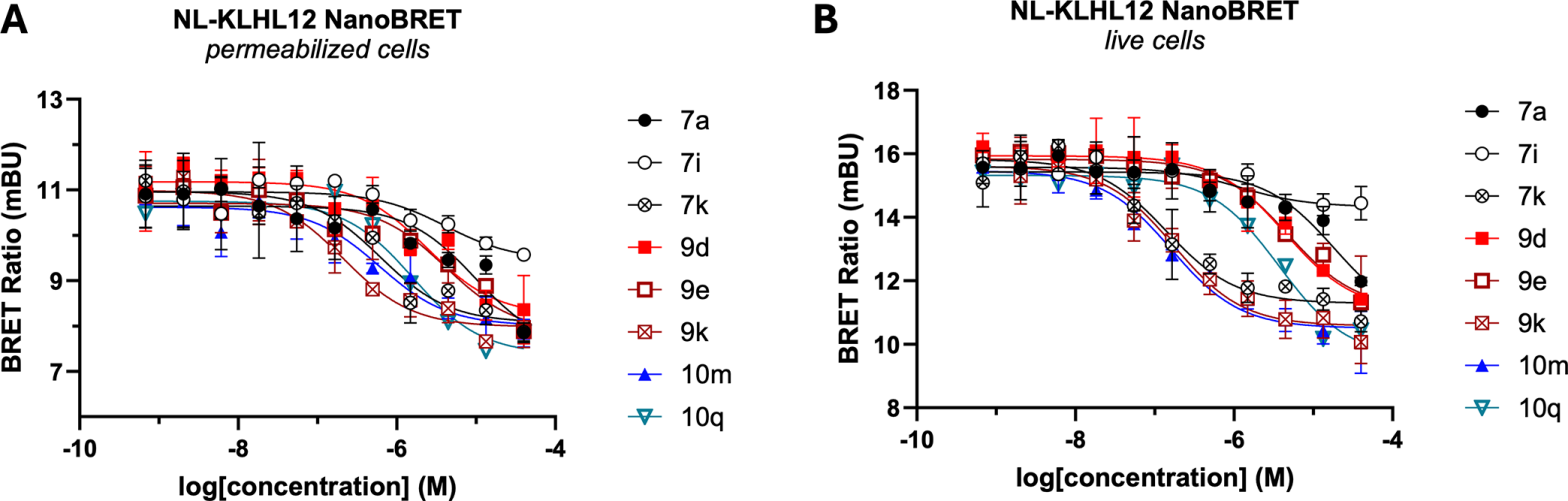
Competition curves indicating displacement of tracer **11b** following treatment of HEK293 cells transfected with a NanoLuc-KLHL12 fusion protein over a range of test compound concentrations.

**Table 5:** IC_50_ Values Determined in the NanoBRET Target Engagement Assay for Selected Compounds

Cmpd	IC_50_ (μM) permeabilized^a^	IC_50_ (μM) intact^b^
**7a**	9.9	>40
**7i**	27.5	>40
**7k**	0.62	0.14
**9d**	2.5	28.6
**9e**	4.2	30.4
**9k**	0.21	0.18
**10m**	0.61	0.16
**10q**	1.6	3.8

a 15 min assay using 200 μM of tracer **11b**.

b 2-h assay using 500 μM of tracer **11b**

The minimally substituted benzimidazole analogs **7a**, **7i**, and **7k** showed affinity to cellular KLHL12 in the permeabilized version of the NanoBRET assay that roughly corresponds to the FPA-derived values, with **7k** displaying the highest affinity of 0.62 μM, similar to the affinities determined by both FPA and SPR. The more elaborated analogs displayed less correlation with the biochemical assay values overall. While compounds **9d** and **9e** showed permeabilized cell affinity in a similar range as their FPA and SPR data, **9k** and **10m** exhibited higher affinity to cellular KLHL12 and **10q** showed reduced binding. The reasons for the binding discrepancies may vary from compound to compound; we speculate that these may be due to varying degrees of nonspecific binding to cellular proteins or perhaps due to differences in the NanoLuc-fusion KLHL12 compared with the protein construct used in the biochemical assays.

Analogs with submicromolar binding to cellular KLHL12 in the permeabilized assay, including **7k**, **9k**, and **10m**, also exhibit high affinity in intact cells, indicating that the permeability of the compounds is generally nonlimiting. Indeed, for **7k** and **10m**, target engagement in intact cells was found to be superior to that in the permeabilized cells, potentially due to the longer timeline of the assay in the intact cell setting.

### Synthesis of Analogs

The KLHL12 ligands described in this manuscript were prepared using a wide variety of synthetic sequences. The general synthesis of most of the reported compounds is shown in Scheme 1. The synthesis starts with the acylation of an aniline derivative **12**, substituted with one or more R^1^ groups, using 2-bromoacetyl chloride (or, in one case, chloroacetyl chloride) in toluene to form the corresponding bromoacetamide **13**. For the majority of the compounds reported in this manuscript, the key intermediate **13** could be converted directly to a final analog by regioselective reaction^26^ with an appropriately substituted benzimidazole **17**, typically using ethanol at 78 °C (condition f in Scheme 1). For compounds requiring the synthesis of an elaborated benzimidazole that was not commercially available and/or for to access specific substitution patterns on the benzimidazole, including **8a**–**d**, **8f**, **8g**, and **8i–n**, the bromide **13** was instead converted to primary amine **14** using aqueous ammonium hydroxide in methanol at 40–45 °C. A subsequent nucleophilic aromatic substitution of **14** with an electron-deficient fluoronitrobenzene derivative in the presence of triethylamine as a base using DMF as the solvent allowed the synthesis of intermediate **15**. The nitroaromatic intermediate **15** was then subjected to catalytic hydrogenation at ambient temperature under a hydrogen atmosphere using platinum­(IV) oxide as the catalyst in a 1:1 mixture of dichloromethane (DCM) and methanol (MeOH), resulting in the corresponding aniline derivative **16**. The final functionalization to obtain the analogs was achieved via alternative conditions starting from **16**. Under condition e1, treatment of **16** with cyanogen bromide in 1:1 MeOH:DCM under argon generated 2-aminobenzimidazole-type products (analogs with R^3^ = NH_2_). Under condition e2, treatment of **16** with triethyl orthoformate or triethyl orthoacetate in methanol led to 1H-benzimidazoles (analogs with R^3^ = H) or 2-methyl-1*H*-benzimidazoles (analogs with R^3^ = CH_3_), respectively.

**Scheme 1:**
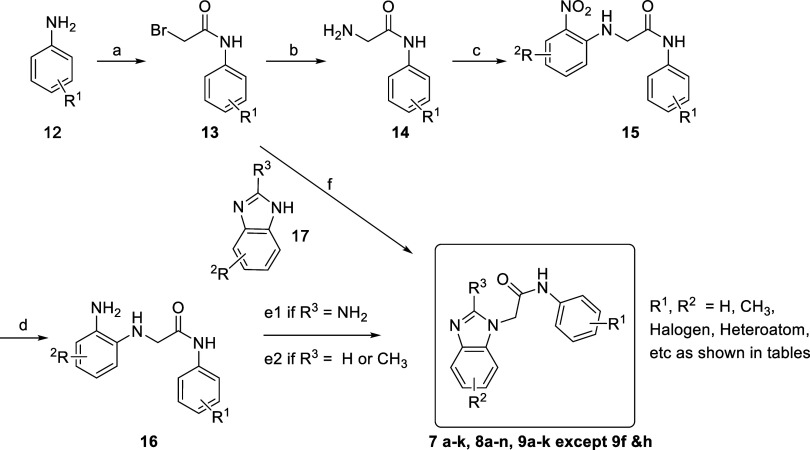
General Routes for the Preparation of Benzimidazole Analogs in Tables 1–3 ^a^

a Reagents and conditions: (a) 2-bromoacetyl chloride, toluene, 90 °C, 12 h, 55–90%; (b) NH_4_OH, MeOH, reflux, 16h, 75–90%; (c) fluoronitrobenzene derivative, TEA, DMF, 75 °C, 30–70%; (d) H_2_, Pt­(IV)­O; MeOH:DCM, 1–3 h, quantitative; (e1) CNBr, MeOH, rt, 30 min-3 h, 35–60%; (e2) triethyl orthoformate or triethyl orthoacetate, MeOH, rt, 15 min- 1 h, 35–75%; (f) appropriately substituted benzimidazole, EtOH, 78 °C, 12 h, 30–70%.

Analog **7l** was prepared in a similar fashion, but with a benzimidazole that was synthesized according to the procedures outlined in Scheme 2. Aryl bromide **18** was subjected to palladium-mediated coupling with a trifluoroborate to install the cyclopropane, resulting in **19**. Benzimidazole cyclization was accomplished using cyanogen bromide in methanol, and then benzimidazole **20** was converted to analog **7l** using the standard conditions outlined above, in which bromide **13** is 2-bromo-*N*-(2,4,6-trichlorophenyl)­acetamide. For this reaction and related 4-substituted benzimidazoles, regioselective alkylation at the 1-position nitrogen was observed due to steric interference from the 4-postion alkyl group.

**Scheme 2:**
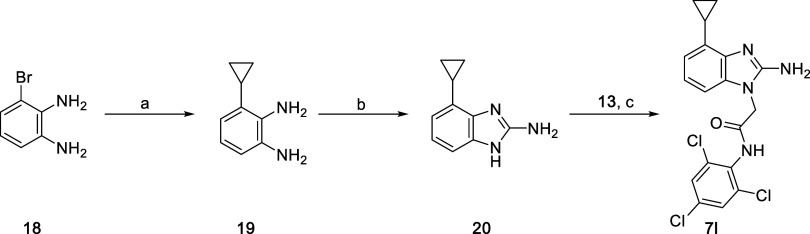
Synthetic Route for Analog **7l** ^a^

a Reagents and conditions: (a) Potassium cyclopropyltrifluoroborate, Pd­(OAc)_2_, Cs_2_CO_3_, Cy_3_P in toluene/H_2_O, 120 °C, o/n; (b) CNBr, MeOH, o/n; (c) **13**, EtOH, 90 °C, 12h.

The synthesis of the benzimidazole derivatives **9** shown in Table 3 that are intended to access the cleft region required the synthesis of anilines **12** using several classical functional group interconversions, the details of which are reported in the Supporting Information. However, selected analogs featured late-stage diversification strategies performed after construction of the elaborated template. Scheme 3 shows one such strategy that was used to access analogs **9f** and **9h**. Compounds **9a** and **9g** were prepared from commercially available anilines via the standard route shown in Scheme 1. Reduction of these compounds with lithium aluminum hydride (LiAlH_4_) in THF provided **21** (from **9a**) and **9h** (from **9g**). To access **9f**, the hydroxyl group of **21** was first converted to bromide **22** using phosphorus tribromide in dichloromethane at 0 °C, and then the bromide was displaced with pyrrolidinone under basic conditions.

**Scheme 3:**
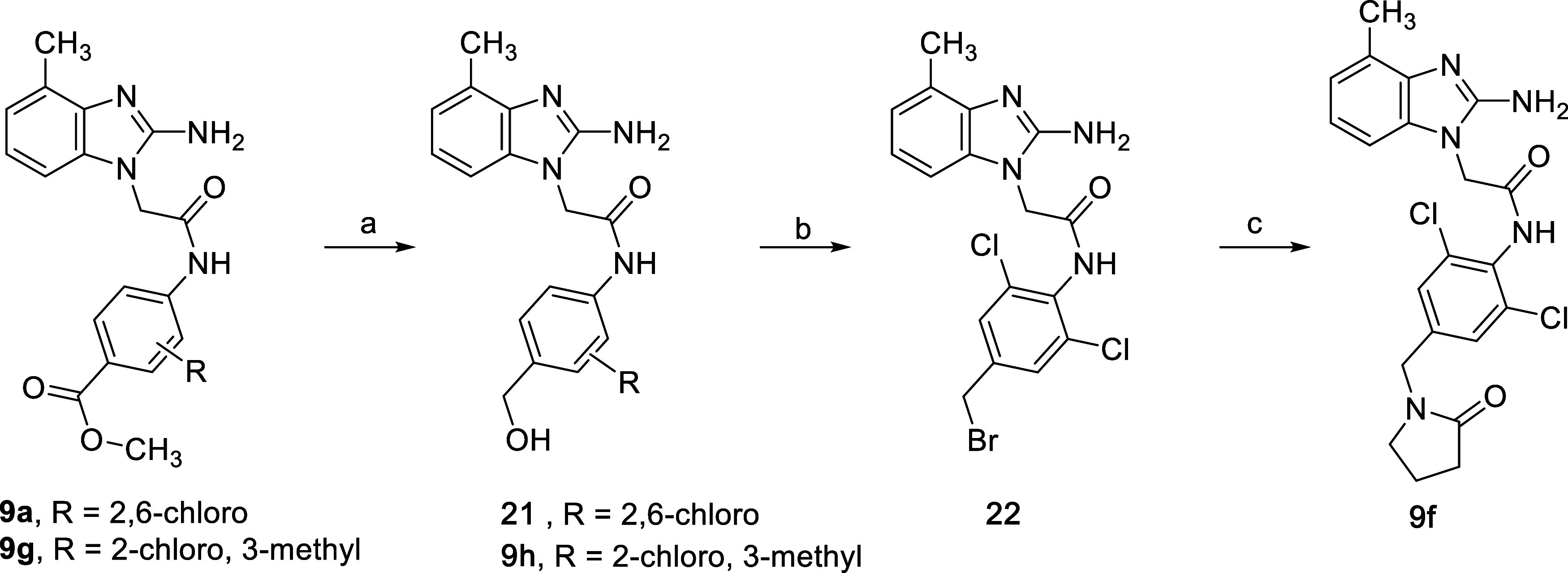
Preparation of **9f** and **9h** ^a^

a Reagents and conditions: (a) LiAlH_4_, THF, rt, 30–60%; (d) PBr_3_, DCM, 0 °C, 12 h, 60%; (e) pyrrolidin-2-one, NaH, rt, 12 h, 40%.

The synthetic chemistry used to prepare the analogs in Table 4 that access the S5 region of KLHL12 was highly varied and typically required derivatization of the compounds after the basic scaffold was assembled. Compounds **10a–c** were prepared as shown in Scheme 4. Intermediate **23** was prepared from 2-amino-4-bromobenzimidazole per the standard route in Scheme 1 and then coupled with ae trifluoroborate derivative using palladium-catalyzed couplings to provide the final analogs.

**Scheme 4:**
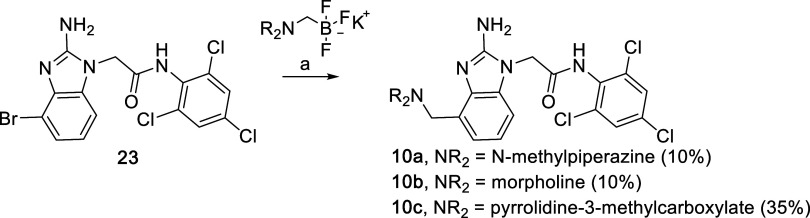
Benzimidazole Derivatization to Prepare **10a**–**c** ^a^

a Reagents and conditions: (a) PdCl_2_(1,1′-bis­(di*tert*-butylphosphino)­ferrocene), Cs_2_CO_3_, THF/H_2_O, 100 °C, 12 h.

The synthesis of analogs **10d-e** and **10g–j** relied on nucleophilic displacement instead of coupling. For these analogs, bromide **24** was first prepared using straightforward transformations as described in the Supporting Information. The functionalized amines were installed using S_N_2 displacement reactions using the available pyrrolidines and cesium carbonate in DMF (Scheme 5).

**Scheme 5:**
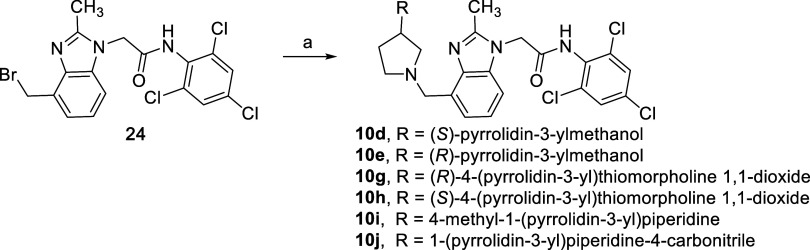
Benzimidazole Derivatization to Prepare Pyrrolidine Analogs **10d**–**e** and **10g**–**j** ^a^

a Reagents and conditions: (a) Substituted pyrrolidine, Cs_2_CO_3_, DMF, rt, 1h, 35–75%.

The synthesis of **10f** incorporated the substituted pyrrolidine using a reductive amination instead of nucleophilic displacement (Scheme 6). Using the standard route in Scheme 1, benzimidazole **25**, bearing an alkene, was prepared (see Supporting Information). Dihydroxylation and oxidative cleavage using OsO_4_ and NaIO_4_ gave the aldehyde **26**, which underwent reductive amination with 4-(pyrrolidin-3-yl)­morpholine and NaBH­(OAc)_3_ in a DMF/AcOH mixture to furnish compound **10f**.

**Scheme 6:**
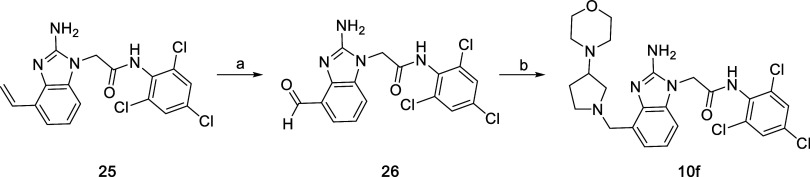
Preparation of **10f** ^a^

a Reagents and conditions (a) OsO_4_, NaIO_4_, 2,6-lutidine, dioxane-H_2_O (3:1), rt, 3 h, quantitative; (b) 4-(pyrrolidin-3-yl)­morpholine, AcOH, NaBH­(OAc)_3_, DMF, 50 °C-rt, 16 h, 38%.

The heavily elaborated cyclopropane-containing benzimidazoles **10m**–**q** were prepared from **10k** and **10l**, according to the procedures outlined in Scheme 7. These cyclopropane derivatives were isolated as *cis* or *trans* isomers, without control of absolute stereochemistry. The *trans* cyclopropane **10k** was synthesized via LiAlH_4_ reduction of intermediate **27** (see Supporting Information) in 90% yield, while the corresponding *cis* cyclopropane **10l** was synthesized from an acid-mediated deprotection of **28** (also described in the Supporting Information). The corresponding aldehydes **29** and **30** were obtained via oxidation of the alcohols using Swern conditions or with the use of Dess-Martin periodinane (DMP). Finally, analogs **10m**–**q** were synthesized by reductive aminations with a variety of amines, using standard conditions.

**Scheme 7:**
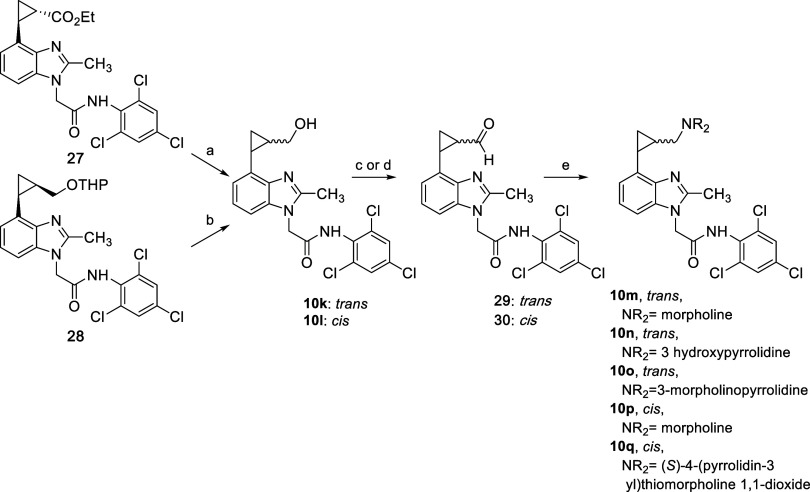
Preparation of Cyclopropanes **10m**–**q** ^a^

a Reagents and conditions: (a) LiAlH_4_, THF, 1 h, 90%, (b) TsOH·H_2_O, MeOH, rt, 3 h, 62%; (c) DMP, CH_2_Cl_2_, 5h, quantitative; (d) (COCl)_2_, DMSO, TEA, THF, −78 °C, 0.5 h, quantitative (e) amine, AcOH, NaBH­(OAc)_3_, DMF, rt, 1–3 h, 20–40%.

The fluorescein-labeled FPA probe **11a** was prepared using procedures similar to those described for analog synthesis (Scheme 8). The available ester-functionalized nitroarene **31** was reduced using standard conditions and the resulting aniline **32** was acylated with bromoacetyl chloride to enable formation of the benzimidazole **34**. To accomplish the synthesis of the probe, the substitutions on the aniline were modified using standard conditions, leading to the functionalized amine **37**. This amine was reacted with fluorescein isothiocyanate (FITC) to produce **11a**. The boron-dipyrromethene-labeled tracer for the NanoBRET target engagement assay was prepared by the reaction of **11b** with an *N*-hydroxysuccinimide derivative bearing a boron-dipyrromethene moiety.

**Scheme 8:**
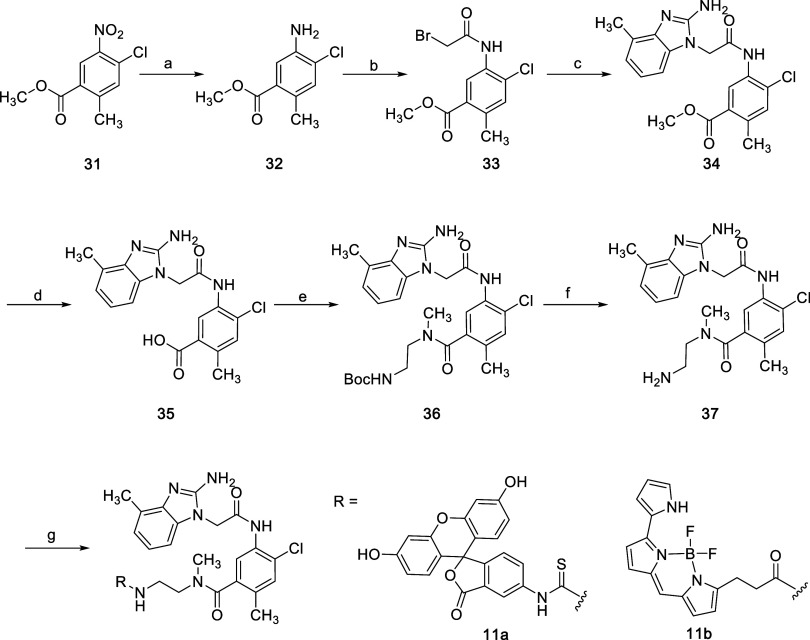
Synthetic Route for Probes **11a** and **11b** ^a^

a Reagents and conditions: (a) SnCl_2_·HCl, MeOH:Water (9:1), 65 °C, 2 h, 80%; (b) 2-bromoacetyl chloride, Toluene, 90 °C, 12 h, 92%; (c) 4-methyl-1*H*-benzo­[*d*]­imidazol-2-amine, Toluene, 90 °C, 12 h, 47%; (d) potassium trimethylsilanolate, THF, rt, 60%; (e) *tert*-butyl (2-(methylamino)­ethyl)­carbamate, HATU, DIEA, DMF, rt, 12h, 28%; (f) TFA, CH_2_Cl_2_, 0 °C-rt, 3 h, quantitative; (g) for **11a:** 3′,6′-dihydroxy-5-isothiocyanato-3*H*-spiro­[isobenzofuran-1,9′-xanthen]-3-one, DIEA, DMF, rt, 2h, 42%. For **11b**: 2,5-dioxopyrrolidin-1-yl 3-(5,5-difluoro-7-(1H-pyrrol-2-yl)-5*H*-5λ^4^,6λ^4^-dipyrrolo­[1,2-c:2′,1′-*f*]­[1,3,2]­diazaborinin-3-yl)­propanoate, DIEA, DMF, rt, 2h, 70%.

## Discussion and Conclusions

KLHL12 is an underexplored E3 ligase with strong potential for use in TPD.^3^ We have shown that KLHL12 is overexpressed in cancer cells relative to normal tissues, suggesting that a KLHL12-recruiting PROTAC may induce cancer-specific degradation of target proteins. Realizing this potential necessarily requires the identification of high-quality and well-validated small molecule KLHL12 ligands. While a peptide that binds to KLHL12 has been previously described,^18^ small molecule KLHL12 ligands have not been reported. Here we demonstrate that NMR-based fragment screening can be used to identify small molecules that bind to KLHL12, namely the moderately potent benzimidazole fragment **1**, which was shown by X-ray crystallography to bind to the substrate recognition domain of KLHL12.

Initial optimization of the fragment hit yielded compound **7k**, with submicromolar affinity and high ligand efficiency (LE)­of 0.38 by FPA-derived measurement and 0.37 by SPR, a significant improvement over **1**. Further structure-based optimization based on **7k** met with mixed success. Attempts to extend the ligand into an adjacent unoccupied cleft near Tyr528 did not improve affinity, despite crystallographically confirmed occupation of this cleft. Similarly, efforts to access a small subpocket occupied by a previously reported peptide binder were hampered by narrow SAR and chemical synthesis challenges. While good affinity was obtained, this came at the expense of significantly reduced ligand efficiency—**10q** shows an LE of 0.19 by FPA and 0.22 by SPR. Furthermore, the fragment-derived compounds showed the ability to bind to cellular KLHL12 in a NanoBRET assay, with **7k**, **9k**, and **10m** demonstrating engagement of the protein at submicromolar levels in intact cells and ones in which the membrane has been compromised by the addition of digitonin.

The identification of tight-binding ligands to KLHL12 as reported here is only one of the steps necessary for development of a KLHL12-based PROTAC. Another major consideration is the establishment of a linking strategy to connect the KLHL12 ligand with a ligand to a protein of interest. Notably, the ligands described here have significant solvent-exposed regions which may serve as viable locations for linker attachment. The 2-position of the benzimidazole is one such possibility, and the aniline moiety has multiple sites from which a linker may extend into the solvent. Indeed, FPA probe **11a**, with a short linker to a fluorescein (Figure 8 and Scheme 8), demonstrates tolerance for extensions similar to those required for PROTAC creation, albeit with some loss of affinity to KLHL12.

While our structure-guided design efforts that led to affinity improvements over the initial hit **1** did not achieve our target affinity of 100–200 nM to initiate PROTAC construction, our work to discover the first small molecules demonstrated to bind to KLHL12 nonetheless provides a promising foundation for the future design and synthesis of KLHL12-based PROTACs.

## Materials and Methods

### RNA-seq Analysis

As reported previously^10^ a list of E3 ligase and accessory genes was compiled from several existing resources (Cell Signaling, hUbiquitome database, UbiProt Database, and DUDE v. 1.0, available at https://esbl.nhlbi.nih.gov/Databases/KSBP2/Targets/Lists/E3-ligases/).^27^ Raw count reads for 11,057 tumors spanning 20 cancer types from TCGA (GDC-PANCAN.htseq_counts.tsv) were downloaded (https://xenabrowser.net/). Details of alignment parameters can be found at https://docs.gdc.cancer.gov/Data/Bioinformatics_Pipelines/Expression_mRNA_Pipeline/. Normal RNA-seq raw counts (GTEx_Analysis_2017–06–05_v8) for 17,382 tissues from rapid autopsies were obtained from the GTEx portal (https://gtexportal.org/). Raw counts from TCGA and GTEx were merged by ensemble gene id, normalized to read depth, scaled (10,000) and log transformed. Differentially expressed genes were identified with the Van Elteren test,^28^ a stratified version of nonparametric Wilcoxon rank-sum test. All statistical tests were performed in R (*version* 4.4).

### Protein Expression Analysis

Cancer cell lines were washed in PBS and resuspended in RIPA lysis buffer containing 1× Thermo Scientific Halt Protease and Phosphatase Inhibitor Cocktail. Lysates were adjusted to a final concentration of 2 mg/mL in LI-COR 1× protein loading buffer. All total protein lysates from normal tissues were purchased from LSBio except platelet lysates which were purchased from Santa Cruz Biotechnology. The heart lysates were purchased from LSBio, Cell Sciences Inc., Santa Cruz Biotechnology, Biochan, and Zyagen. Total protein heart lysates received from Cell Sciences, Biochain, LSBio, and Santa Cruz Biotechnology were received in lysis buffer and were either at or adjusted to 2 mg/mL. Samples from Zyagen were provided at 5 mg/mL in protein lysis buffer and were diluted to 2 mg/mL in RIPA and 1× protein loading buffer. Western blot analysis was carried out using XCell SureLock Blot Module (Thermo Fisher Scientific) on nitrocellulose membranes and blocked in Blocking One blocking buffer (Nacalai Tesque). KLHL12 antibody was purchased from Santa Cruz Biotechnology (Antibody D-1) and GAPDH antibodies were purchased from Cell Signaling Technology (14C10). IRDye Secondary antibodies were purchased from LI-COR. Image analysis was performed on a LI-COR Odyssey Imaging System.

### Protein Expression and Purification

The genes containing the Kelch domain of human KLHL12 (residues 279–567) and KEAP1 (residues 321–609) were synthesized with codon optimization for *E. coli* and cloned in the pET28a­(+) vector by GenScript. All plasmids were transformed into the BL21­(DE3) strain *E. coli*. The bacteria were cultured in Luria–Bertani broth or M9 minimal media containing ^15^NH_4_Cl supplemented with 50 mg/mL Kanamycin at 37 °C until the optical density at 600 nm reached 0.8 before inducing protein expression by the addition of 0.5 mM IPTG at 15 °C for 40 h. The cell pellet was harvested by centrifugation at 5000*g* for 15 min, resuspended in lysis buffer (50 mM HEPES pH 7.5, 500 mM NaCl, 5%glycerol, 10 mM imidazole, 5 mM BME) and lysed in APV2000 lab homogenizer (SPX flow). Cell lysate was centrifuged at 15000*g* for an hour and loaded onto HisTrap FF column (Cytiva). The column was washed with 10 column volumes of lysis buffer and eluted with elution buffer (50 mM HEPES pH 7.5, 500 mM NaCl, 5% glycerol, 500 mM imidazole, 5 mM BME) using a linear gradient program from 0 to 100% elution buffer over 10 column volumes. The fractions containing target proteins were pooled, the His-tag was cleaved by adding Thrombin, and the mixture was dialyzed against lysis buffer without imidazole at 4 °C overnight. Tag-free proteins were loaded on a HisTrap column. The flowthrough was concentrated and subjected to HiLoad 26/600 Superdex75 pg (Cytiva) and eluted using size exclusion buffer (25 mM HEPES pH 7.0, 300 mM NaCl, 3 mM DTT) for X-ray crystallography and the enzymatic assays or NMR buffer (25 mM HEPES pH 7.0, 100 mM NaCl, 3 mM DTT) for the NMR-based fragment screen. Protein concentration was quantified by the Pierce 660 nm assay (ThermoFisher).

### NMR Experiments

All NMR experiments were performed at 25 °C using a 600 MHz Bruker Avance III spectrometer equipped with a 5 mm single-axis x-gradient cryoprobe and a Bruker SampleJet. Gradient-enhanced, two-dimensional ^1^H–^15^N heteronuclear multiple-quantum coherence (SOFAST-HMQC)^29^ spectra of KLHL12 were recorded using 24 scans of 12 min acquisition times and analyzed using Topspin 4.1.4 (Bruker). Our in-house fragment library of 13,824 compounds was screened as mixtures of 12 fragments prepared in 12 96-well plates. Each NMR sample was made using 15 μM of ^15^N-labeled KLHL12, 800 μM of each fragment, and 5% DMSO-*d*_6_ for spectrometer locking in 5 mm-diameter NMR tubes. Hit mixtures were identified by comparing the chemical shifts of the backbone resonances to a ligand-free KLHL12 spectrum and then deconvoluted by screening individual fragments.

SOFAST-HMQC titration experiments were used to determine binding affinity of the fragment hits identified from the screen. The changes in ^1^H–^15^N chemical shifts of backbone resonances upon the addition of increasing concentrations of the fragments (0.125–2 mM) were analyzed. The binding affinities of the fragments were calculated using the Hill equation model in Prism 10 (GraphPad).

### KLHL12 SPR Binding

All SPR experiments were performed on a Biacore 8K or 8K+ (Cytiva) using Series S NTA sensor chips (Cytiva, cat# BR100532). His-tagged KLHL12 was immobilized to the sensor chip surface on flow cell 2 using a His-tag capture/couple approach in 10 mM HEPES pH 7.4, 150 mM NaCl, 0.05% Tween-20 (HBS-P+), 0.5 mM TCEP to levels of ∼5500 RU. Following conditioning with 350 mM EDTA and capture of 0.5 mM NiCl_2_, the chip surface was activated with a 7 min injection of EDC and NHS. KLHL12 was injected at a concentration of 10 μg/mL with a contact time of 300 s and a flow rate of 5 μL/min. The chip surface was deactivated with a 7 min injection of 1 M ethanolamine pH 8.5 and washed with a 1 min injection of 350 mM EDTA to remove Ni^2+^ and any ligand that was not covalently coupled. Flow cell 1 was activated with EDC/NHS and deactivated with ethanolamine as a reference surface. Analytes were diluted in DMSO to 20× the highest concentration to be measured. The 20× stocks were diluted in HBS-P+, 3 mM EDTA (HBS-EP+), 0.5 mM TCEP to obtain 1× solutions with 5% DMSO in running buffer. A 2-fold serial dilution in running buffer with 5% DMSO was used to generate 10 doses. These were injected from lowest to highest concentration using a contact time of 80 s, a dissociation time of 120 s, and a flow rate of 30 μL/min. Data for each sample were collected in at least duplicate to ensure reproducibility. Data were analyzed using Biacore Insight Evaluation Software (Cytiva). All data were doubled referenced (blank- and reference-subtracted) and solvent corrected. Data were fit using a steady state affinity model using data 5 s before injection end with a 5 s window.

### Competitive FPA Binding Assay for KLHL12

A previously described peptide fragment from protein disheveled 3 conjugated with FITC chromophore^18^ was used as a fluorescent probe. The tested compounds were serially diluted at 2-fold for 10 dilutions. The highest compound concentration in the final reaction was 0.5 mM. Diluted compounds were added to each well of a 384-well black plate (Greiner 781900) at 1 μL per well. KLHL12 protein was prepared to 100 μM and 10 μL was added to each well. The probe was finally added to each well above at 10 μL, 100 nM and incubated at room temperature on a plate shaker for 20 min. The plate was read on the plate reader (Biotek Cytation3). The polarized fluorescence (anisotropy) was plotted and analyzed with software XLfit (IDBS, United Kingdom). *K_i_* values were determined from the data using a variation of the Cheng-Prusoff equation in a manner to that previously described.^21^ Small-molecule derived *K_i_* values were determined in the same way using a slight variation of the assay conditions using the labeled probe compound **11a** (Scheme S13).

### Sequence Alignment and Homology Models

All sequences of Kelch-like proteins (KLHLs) were obtained from UniProt database. The protein sequences of the Kelch domain of each protein were aligned and analyzed by Clustal Omega 2.1.^22^ All homology models were predicted and generated by Alphafold 2.0 ^25^ using the sequences of Kelch domain of each KLHL protein.

### Saturation Binding Assay for KEAP1

Protein was serially diluted at 2-fold for 15 dilutions. 10 μL of the diluted protein was added to each well of 384-well black plate (Greiner 781900). An equal volume of fluorescent probe (**11**) was added to the same wells as above. The mixtures were incubated at room temperature on a plate shaker for 20 min and read on a plate reader (Biotek Cytation3). The polarized fluorescence (anisotropy) was plotted and analyzed with software XLfit (IDBS, United Kingdom).

### Protein Crystallography

10 mg/mL KLHL12 was mixed with a 200 mM DMSO stock of the desired ligands at the final concentrations of 5 mM ligand and 2.5% DMSO and incubated at 4 °C overnight. Crystallization drops of 1 μL of KLHL12 mixture +1 μL of reservoir solution were set as hanging drops, streak seeded using a cat whisker with the 100× diluted microseeds stock and incubated at 18 °C allowing vapor diffusion against the corresponding reservoir solution. The crystals were obtained in 0.1 M Bis-Tris pH 5.5, 0.2 M MgCl_2_ and 25% PEG3350, and cryo-protected in mother liquor supplemented with 25% glycerol before being flash-frozen in liquid nitrogen. X-ray experiments were performed at 100 K on the Life Sciences Collaborative Access Team (LS-CAT) Sector-21 beamlines at the Advanced Photon Source (APS), Argonne National Laboratory, on beamline 8.2.1 at Advance Light Source, Lawrence Berkeley National Laboratory, and on beamline 17-ID-1 at National Synchrotron Light Source II, Brookhaven National Laboratory. Diffraction data were indexed, integrated, and scaled with iMosflm.^30^ Phasing was accomplished by molecular replacement with Phaser^31^ using the structure of KLHL12 (PDB: 2VPJ) as the starting model. Ligand models were built by AceDRG^32^ and manually added to the corresponding electron density. Co-crystallized KLHL12 structures with ligands were determined by several cycles of refinement using Phenix^33^ and manual modeling with COOT.^34^

### KLHL12 NanoBRET Assay

Protocols were adapted from Promega’s instructions included in the FuGENE HD Transfection and Nano-Glo Luciferase Assay System Technical Manuals. To prepare the cells for transient expression of Nanoluciferase-KLHL12, HEK293 cells were plated and grown to 80% confluency in DMEM (gibco, 11965092) containing 10% Fetal Bovine Serum (Sigma, 21G638). Cells were harvested with trypsin and resuspended at 2 × 10^5^ cells/mL in a total volume of 20 mL of media in a T75 flask. The Nanoluc-KLHL12 DNA expression vector (5 μg, Promega), Carrier DNA expression vector (5 μg, Promega, E488A), and Fugene (30 μL, Promega, E231A) were combined in 1 mL of OptiMEM (gibco, 11058021) and placed at room temperature for 15 min while occasionally tapping to mix the tube. The DNA/Fugene HD mixture was added to the cells and left to incubate at 37 °C, 5% CO_2_ overnight. The following day, a compound dilution series was prepared for each compound from 20 mM DMSO stocks at a 1:3 dilution scheme over an 11-point curve. From each dilution, 1 μL of compound was mixed with 49 μL OptiMEM in a 384-well plate (Greiner, 781280). Transfected cells were harvested and resuspended at 2 × 10^5^ cells/mL in OptiMEM. A digitonin solution (Sigma, D141) was prepared in OptiMEM with a final concentration of 0.5 mg/mL. Dilutions of tracer **11b** were prepared in tracer dilution buffer (Promega, N2191) at 200 μM (permeabilized cells) and 500 μM (live cells). For permeabilized cell mode, 30 μL of cells were mixed with 4 μL of compound, 2 μL of tracer **11b** (200 μM), and 4 μL digitonin solution in a 384-well solid white plate (Greiner, 781904, nonbinding). The plate was placed at room temperature for 15 min. The NanoLuc substrate solution (Promega, N157C) was prepared by adding 30 μL substrate (5 mM in 100% ethanol) to 5 mL OptiMEM. 20 μL of the diluted substrate mixture was added to each well and read on BioTek Cytation3 (450/610 filter cube). For live cell mode, 30 μL of cells were mixed with 4 μL of compound, 2 μL of tracer **11b** (500 μM), and 4 μL OptiMEM in a 384-well solid white plate (Greiner, 781904, nonbinding). The plate was placed at 37 °C, 5% CO_2_ for 2 h. After 2 h, the NanoLuc substrate solution was prepared by adding 30 μL substrate (5 mM in 100% ethanol) and 10 μL extracellular NanoLuc inhibitor (Promega, N235B) to 5 mL OptiMEM. 20 μL of the diluted substrate/inhibitor mixture was added to each well and read on BioTek Cytation3 (450/610 filter cube).

### Synthesis and Characterization of Compounds

#### General Experimental Information

All NMR spectra were recorded at room temperature on a 400 MHz AMX Bruker spectrometer. ^1^H chemical shifts are reported in δ values in ppm downfield with the deuterated solvent as the internal standard. Data are reported as follows: chemical shift, multiplicity (s = singlet, d = doublet, t = triplet, q = quartet, br = broad, m = multiplet), integration, coupling constant (Hz). Low resolution mass spectra were obtained on an Agilent 1200 series 6140 mass spectrometer with electrospray ionization. All samples were of ≥ 95% purity as analyzed by LC-UV/vis-MS. Analytical HPLC was performed on an Agilent 1200 series with UV detection at 214 and 254 nm along with ELSD detection. LC/MS parameters were as follows: Phenomenex-C18 Kinetex column, 50 × 2.1 mm^2^, 2 min gradient, 5% (0.1% TFA/MeCN)/95% (0.1% TFA/H_2_O) to 100% (0.1% TFA/MeCN). Preparative purification was performed on a Gilson HPLC (Phenomenex-C18, 100 × 30 mm^2^, 10 min gradient, 5 → 95% MeCN/H2O with 0.1% TFA) or by automated flash column chromatography (Isco, Inc. Combiflash). Solvents for extraction, washing, and chromatography were HPLC grade. All reagents were purchased from chemical suppliers and used without purification.

### General Synthetic Procedure for the Preparation of Analogs **7a**–**k**, **8a**–**n** and **9a**–**k**

#### Synthesis of **13**

A substituted aniline derivative, either commercially available or prepared according to the procedures in the Supporting Information (2.0 g, 1.0 equiv, 0.01 mol) was placed in a round-bottom flask, and toluene (15 mL) was added. The mixture was allowed to stir until homogeneous, and 2-bromoacetyl chloride (2.0 g, 0.9 mL, 1.1 equiv, 0.01 mol) was added dropwise over 5 min at room temperature. The reaction mixture was heated at reflux for 12 h and then allowed to cool to room temperature. The solid was filtered and triturated with hexane, then filtered again. The crude product was used without further purification.

#### Synthesis of **14**

Bromide **13** (8.7 g, 27 mmol, 1.0 equiv) was placed in a round-bottom flask, and aqueous ammonium hydroxide (100 mL) and methanol (35 mL) were added. The mixture was allowed to stir for 12 h at 40–45 °C. After 12 h, the solvents were evaporated under reduced pressure. The residue was triturated with hexane and filtered using a Büchner funnel. The product **14** was obtained as a white solid and used in the next step without further purification.

#### Synthesis of **15**

To a solution containing **14** (1.0 equiv) in DMF (2 mL), the appropriate fluoronitrobenzene (1.1 equiv) was added, followed by triethylamine (3.0 equiv). The reaction mixture was stirred at 75 °C overnight, then cooled to room temperature, and quenched by the addition of a saturated aqueous NaHCO_3_ solution. The mixture was extracted with EtOAc (3 × 20 mL). The organic layers were combined and evaporated to dryness, yielding a yellow solid. The crude product was washed with heptanes and collected by filtration using a Büchner funnel. The resulting solid was dried under vacuum to afford compound **15** which was used in the subsequent step without further purification.

#### Synthesis of **16**

To a solution containing **15** (1.0 equiv) in DCM and MeOH was added platinum­(IV) oxide (0.1 equiv). The mixture was degassed using house vacuum (3 times) and backfilled with H_2_ gas using a balloon. The reaction mixture was allowed to stir at room temperature under an atmosphere of hydrogen for 12 h. After Celite filtration, the filtrate was dried over anhydrous Na_2_SO_4_ and concentrated to dryness under reduced pressure to afford **16** as yellow solid, which was used without further purification.

The final compounds **7a**–**k**, **8a**–**n** and **9a**–**k** (except **7l**, **9f** and **9h**) were obtained using the appropriate conditions as described below. The syntheses of the intermediates required (if not commercially available) are described in the Supporting Information.

Condition e1: A solution of **16** (1.0 equiv) in a 1:1 mixture of MeOH/DCM was stirred at room temperature for 15 min under an argon atmosphere. Cyanogen bromide (1.3 equiv) was then added, and the reaction mixture was stirred for an additional 15 min before being concentrated under reduced pressure. The residue was partitioned between a biphasic system of 10%MeOH/DCM and a saturated aqueous sodium bicarbonate solution. The organic layer was extracted three times with DCM, dried over anhydrous sodium sulfate (Na_2_SO_4_), filtered, and concentrated. The crude product was purified by reverse-phase high-performance liquid chromatography (RP-HPLC) using a water/acetonitrile (H_2_O/ACN) gradient with 0.1% TFA to obtain the final product as a solid.

Condition e2: A mixture of **16** (1.0 equiv), triethylorthoformate (1.2 equiv), and 4-toluenesulfonic acid hydrate (0.1 equiv) in a mixture of 1:1 mixture of MeOH:DCM was allowed to stir at room temperature for 1 h. The reaction mixture was then concentrated to dryness under reduced pressure. The crude residue was purified by reverse-phase high-performance liquid chromatography (RP-HPLC) using a gradient (5–95%, H_2_O/ACN, 0.1% TFA) to afford the product as a solid.

Condition f: To a solution containing corresponding benzimidazole **17** (1.0 equiv) in EtOH (3 mL), the appropriately substituted bromo acetamide **13** (1.09 equiv) was added. The reaction mixture was allowed to stir at 90 °C overnight. The mixture was allowed to cool to room temperature and concentrated to dryness. The residue was quenched by the addition of a saturated aqueous sodium bicarbonate solution. The organics were extracted using a 10% methanol:dichloromethane solution (3 × 15 mL), dried over anhydrous Na_2_SO_4_, and concentrated under reduced pressure. The crude product was purified by reverse-phase high-performance liquid chromatography (RP-HPLC) using a gradient (5–95%, H_2_O/ACN, 0.1% TFA) to afford the required product as a solid.

#### 2-(Benzimidazol-1-yl)-*N*-(2-chloro-3,4-dimethylphenyl)­acetamide (**7a**)

12 mg, 12%, Condition f. LCMS: *R*_T_ = 1.317 min, >95%@215 and 254 nm, *m*/*z* = 314.2 [M + H]^+^. 1H NMR (400 MHz, DMSO-*d*_6_) δ 9.93 (s, 1H), 8.24 (s, 1H), 7.67 (dd, *J* = 7.6, 1.3 Hz, 1H), 7.55 (d, *J* = 7.9 Hz, 1H), 7.42 (d, *J* = 8.2 Hz, 1H), 7.25 (dtd, *J* = 21.3, 7.2, 1.2 Hz, 2H), 7.12 (d, *J* = 8.2 Hz, 1H), 5.25 (s, 2H), 2.31 (s, 3H), 2.27 (s, 3H).

#### 2-(Benzimidazol-1-yl)-*N*-(2,6-dichloro-4-methylphenyl)­acetamide (**7b**)

Condition f. LCMS: *R*_T_ = 1.429 min, >95%@215 and 254 nm, *m*/*z* = 334.1 [M + H]^+^. 1H NMR (400 MHz, DMSO-*d*_6_) δ 10.33 (s, 1H), 8.25 (s, 1H), 7.67 (dd, *J* = 7.8, 1.2 Hz, 1H), 7.55–7.48 (m, 1H), 7.41–7.37 (m, 2H), 7.25 (dtd, *J* = 24.3, 7.3, 1.2 Hz, 2H), 5.23 (s, 2H), 2.31 (s, 3H).

#### 2-(Benzimidazol-1-yl)-*N*-(2,4,6-trichlorophenyl)­acetamide (**7c**)

4 mg, 4%, Condition f. LCMS: *R*_T_ = 0.811 min, >98%@215 and 254 nm, *m*/*z* = 354.2 [M + H]^+^. ^1^H NMR (400 MHz, CD_3_OD) δ 9.34 (s, 1H), 7.93–7.81 (m, 2H), 7.71–7.64 (m, 2H), 7.62 (s, 2H), 5.60 (s, 2H).

#### 2-(2-Methylbenzimidazol-1-yl)-*N*-(2,4,6-trichlorophenyl)­acetamide (**7d**)

Condition f. LCMS: *R*_T_ = 0.840 min, >95%@215 and 254 nm, *m*/*z* = 368.2 [M + H]^+^. ^1^H NMR (400 MHz, CDCl_3_) δ 7.78–7.69 (m, 1H), 7.68–7.60 (m, 1H), 7.58–7.46 (m, 2H), 7.38 (s, 2H), 5.32 (s, 2H), 2.88 (s, 3H).

#### 2-(2-Aminobenzimidazol-1-yl)-*N*-(2,4,6-trichlorophenyl)­acetamide (**7e**)

Twenty-five mg, 15%, Condition f. LCMS: *R*_T_ = 0.841 min, >95%@215 and 254 nm, *m*/*z* = 369.2 [M + H]^+^. ^1^H NMR (400 MHz, CD_3_OD) δ 7.61 (s, 2H), 7.44 (dt, *J* = 6.9, 2.0 Hz, 2H), 7.40–7.32 (m, 2H), 5.20 (s, 2H).

#### 2-[2-(Dimethylamino)­benzimidazol-1-yl]-*N*-(2,4,6-trichlorophenyl)­acetamide (**7f**)

Twenty-one mg, 20%, Condition f. LCMS: *R*_T_ = 0.840 min, >98%@215 and 254 nm, *m*/*z* = 397.3 [M + H]^+^.^1^H NMR (400 MHz, CD_3_OD) δ 7.64 (s, 2H), 7.50 (dddd, *J* = 7.8, 4.6, 3.4, 1.6 Hz, 2H), 7.46–7.38 (m, 2H), 5.31 (s, 2H), 3.40 (s, 6H).

#### 2-(2-Acetamidobenzimidazol-1-yl)-*N*-(2,4,6-trichlorophenyl)­acetamide (**7g**)

35 mg, 21%, Condition f. LCMS: *R*_T_ = 0.897 min, >98%@215 and 254 nm, *m*/*z* = 411.2 [M + H]^+^. ^1^H NMR (400 MHz, DMSO-*d*_6_) δ 7.78 (s, 2H), 7.52 (d, *J* = 22.6 Hz, 2H), 7.21 (t, *J* = 8.3 Hz, 2H), 5.07 (d, *J* = 35.8 Hz, 2H), 2.10 (d, *J* = 27.5 Hz, 3H).

#### 2-[2-(Aminomethyl)­benzimidazol-1-yl]-*N*-(2,4,6-trichlorophenyl)­acetamide (**7h**)

31 mg, 38%, Condition f. LCMS: *R*_T_ = 1.451 min, >98%@215 and 254 nm, *m*/*z* = 383.1 [M + H]^+^. 1H NMR (400 MHz, DMSO-*d*_6_) δ 10.66 (s, 1H), 8.64–8.59 (bs, 2H), 7.80 (s, 2H), 7.67 (dd, *J* = 27.0, 7.9 Hz, 2H), 7.39–7.23 (m, 2H), 5.32 (s, 2H), 4.43 (s, 2H).

#### 1-[2-Oxo-2-(2,4,6-trichloroanilino)­ethyl]­benzimidazole-2-carboxamide (**7i**)

25 mg, 30%, Condition f. LCMS: *R*_T_ = 0.978 min, >98%@215 and 254 nm, *m*/*z* = 397.3 [M + H]^+^. ^1^H NMR (400 MHz, DMSO-*d*_6_) δ 10.36 (s, 1H), 8.30 (s, 1H), 7.85–7.81 (m, 1H), 7.77 (t, *J* = 1.0 Hz, 0H), 7.76 (s, 2H), 7.62 (d, *J* = 8.1 Hz, 1H), 7.37 (dddd, *J* = 27.2, 8.2, 7.1, 1.2 Hz, 2H), 5.71 (s, 2H).

#### 2-(2,4-Dimethyl-1*H*-benzo­[*d*]­imidazol-1-yl)-*N*-(2,4,6-trichlorophenyl)­acetamide (**7j**)

85 mg, 81%, Condition f. LCMS: *R*_T_ = 1.481 min, purity >95%, *m*/*z* = 383.9 [M + H]^+^; 1H NMR (400 MHz, DMSO-*d*_6_) δ 10.69 (s, 1H), 7.80 (s, 2H), 7.22 (d, *J* = 8 Hz, 1H), 7.49 (t, *J* = 8 Hz, 1H), 7.38 (d, *J* = 8 Hz, 1H), 5.56 (s, 2H), 2.81 (s, 3H), 2.63 (s, 3H).

#### 2-(2-Amino-4-methylbenzimidazol-1-yl)-*N*-(2,4,6-trichlorophenyl)­acetamide (**7k**)

42 mg, 23%, Condition f. LCMS: *R*_T_ = 0.937 min, >98%@215 and 254 nm, *m*/*z* = 383.3 [M + H]^+^. 1H NMR (400 MHz, DMSO-*d*_6_) δ 10.26 (s, 1H), 7.71 (s, 2H), 6.89 (dd, J = 5.5, 3.4 Hz, 1H), 6.83–6.73 (m, 2H), 6.43 (s, 2H), 4.84 (s, 2H), 2.36 (s, 3H).

#### 2-(2-Amino-6-methoxybenzimidazol-1-yl)-*N*-(2,4,6-trichlorophenyl)­acetamide (**8a**)

42 mg, 69%, Condition e1. LCMS: *R*_T_ = 0.879 min, >98%@215 and 254 nm, *m*/*z* = 399.3 [M + H]^+^. ^1^H NMR (400 MHz, DMSO-*d*_6_) δ 10.52 (s, 1H), 8.69 (s, 2H), 7.81 (s, 2H), 7.31 (d, *J* = 8.7 Hz, 1H), 7.01 (d, *J* = 2.4 Hz, 1H), 6.89 (dd, *J* = 8.7, 2.4 Hz, 1H), 5.16 (s, 2H), 3.78 (s, 3H).

#### 2-(2-Amino-5-methoxybenzimidazol-1-yl)-*N*-(2,4,6-trichlorophenyl)­acetamide (**8b**)

20 mg, 13%, Condition e1. LCMS: *R*_T_ = 0.908 min, >98%@215 and 254 nm, *m*/*z* = 399.2 [M + H]^+^. ^1^H NMR (400 MHz, DMSO-*d*_6_) δ 10.51 (s, 1H), 8.73 (s, 2H), 7.80 (s, 2H), 7.28 (d, *J* = 8.8 Hz, 1H), 6.97 (d, *J* = 2.4 Hz, 1H), 6.91 (dd, *J* = 8.8, 2.4 Hz, 1H), 5.14 (s, 2H), 3.79 (s, 3H).

#### 2-(2-Amino-6-methylbenzimidazol-1-yl)-*N*-(2,4,6-trichlorophenyl)­acetamide (**8c**)

48 mg, 44%, Condition e1. LCMS: *R*_T_ = 0.911 min, >98%@215 and 254 nm, *m*/*z* = 383.3 [M + H]^+^. ^1^H NMR (400 MHz, DMSO-*d*_6_) δ 10.58 (s, 1H), 8.75 (s, 2H), 7.81 (s, 2H), 7.30 (d, *J* = 8.1 Hz, 1H), 7.18 (s, 1H), 7.15–7.08 (m, 1H), 5.15 (s, 2H), 2.39 (s, 3H).

#### 2-(2-Amino-5-methylbenzimidazol-1-yl)-*N*-(2,4,6-trichlorophenyl)­acetamide (**8d**)

Seventeen mg, 10%, Condition e1. LCMS: *R*_T_ = 0.917 min, >98%@215 and 254 nm, *m*/*z* = 383.2 [M + H]^+^. 1H NMR (400 MHz, CD_3_OD) δ 7.61 (s, 2H), 7.34–7.23 (m, 2H), 7.19 (d, *J =* 8.3 Hz, 1H), 5.16 (s, 2H), 2.47 (s, 3H).

#### 2-(2-Amino-5,6-dimethylbenzimidazol-1-yl)-*N*-(2,4,6-trichlorophenyl)­acetamide (**8e**)

Twenty-five mg, 35%, Condition f. LCMS: *R*_T_ = 1.655 min, >98%@215 and 254 nm, *m*/*z* = 397.0 [M + H]^+^. 1H NMR (400 MHz, DMSO-*d*_6_) δ 10.58 (s, 1H), 8.69 (s, 2H), 7.80 (s, 2H), 7.17 (d, *J* = 17.6 Hz, 2H), 5.13 (s, 2H), 2.29 (s, 3H), 2.28 (s, 3H).

#### 2-(2-Amino-5-fluorobenzimidazol-1-yl)-*N*-(2,4,6-trichlorophenyl)­acetamide (**8f**)

Twenty-one mg, 27%, Condition e1. LCMS: *R*_T_ = 1.498 min, >98%@215 and 254 nm, *m*/*z* = 388.9 [M + H]^+^. 1H NMR (400 MHz, CD_3_OD) δ 7.60 (s, 2H), 7.46–7.33 (m, 1H), 7.23 (dd, *J* = 8.3, 2.4 Hz, 1H), 7.13 (td, *J* = 9.2, 2.4 Hz, 1H), 5.19 (s, 2H).

#### 2-(2-Amino-6-chlorobenzimidazol-1-yl)-*N*-(2,4,6-trichlorophenyl)­acetamide (**8g**)

38 mg, 25%, Condition e1. LCMS: *R*_T_ = 0.982 min, >98%@215 and 254 nm, *m*/*z* = 403.2 [M + H]^+^. ^1^H NMR (400 MHz, DMSO-*d*_6_) δ 10.53 (s, 1H), 8.84 (s, 2H), 7.81 (s, 2H), 7.53 (d, *J* = 1.9 Hz, 1H), 7.40 (d, *J* = 8.5 Hz, 1H), 7.32 (dd, *J* = 8.5, 1.9 Hz, 1H), 5.19 (s, 2H).

#### 2-(2-Amino-5,6-difluorobenzimidazol-1-yl)-*N*-(2,4,6-trichlorophenyl)­acetamide (**8h**)

Condition f. LCMS: *R*_T_ = 0.938 min, >98%@215 and 254 nm, *m*/*z* = 405.2 [M + H]^+^. ^1^H NMR (400 MHz, DMSO-*d*_6_) δ 10.45 (s, 1H), 8.70 (s, 2H), 7.80 (s, 2H), 7.60 (dd, *J* = 10.3, 6.8 Hz, 1H), 7.50 (dd, *J* = 10.2, 6.9 Hz, 1H), 5.16 (s, 2H).

#### 2-(2-Amino-5-fluoro-4-methylbenzimidazol-1-yl)-*N*-(2,4,6-trichlorophenyl)­acetamide (**8i**)

Twenty-five mg, 21%, Condition e1. LCMS: *R*_T_ = 0.972 min, >98%@215 and 254 nm, *m*/*z* = 403.2 [M + H]^+^. ^1^H NMR (400 MHz, DMSO-*d*_6_) δ 10.48 (s, 1H), 8.60 (s, 2H), 7.79 (s, 2H), 7.24–7.09 (m, 2H), 5.15 (s, 2H), 2.36 (d, *J* = 1.5 Hz, 3H).

#### 2-(2-Amino-4,5-dimethylbenzimidazol-1-yl)-*N*-(2,4,6-trichlorophenyl)­acetamide (**8j**)

35 mg, 35%, Condition e1. LCMS: *R*_T_ = 1.578 min, >98%@215 and 254 nm, *m*/*z* = 396.9 [M + H]^+^. ^1^H NMR (400 MHz, DMSO-*d*_6_) δ 10.30 (s, 1H), 7.75 (s, 2H), 6.81 (d, *J* = 7.9 Hz, 1H), 6.73 (d, *J* = 8.0 Hz, 1H), 6.53–6.42 (m, 2H), 4.88 (s, 2H), 2.28 (s, 3H), 2.23 (s, 3H).

#### 2-(2-Amino-4,6-dimethylbenzimidazol-1-yl)-*N*-(2,4,6-trichlorophenyl)­acetamide (**8k**)

48 mg, 29%, Condition e1. LCMS: *R*_T_ = 1.048 min, >98%@215 and 254 nm, *m*/*z* = 397.2 [M + H]^+^. ^1^H NMR (400 MHz, DMSO-*d*_6_) δ 10.56 (s, 1H), 8.56 (s, 2H), 7.79 (s, 2H), 7.02–6.92 (m, 2H), 5.12 (s, 2H), 2.39 (s, 3H), 2.34 (s, 3H).

#### 2-(2-Aminoimidazo­[4,5-*b*]­pyridin-3-yl)-*N*-(2,4,6-trichlorophenyl)­acetamide (**8l)**

37 mg, 35%, Condition e1. LCMS: *R*_T_ = 0.878 min, >98%@215 and 254 nm, *m*/*z* = 370.2 [M + H]^+^. ^1^H NMR (400 MHz, DMSO-*d*_6_) δ 10.50 (s, 1H), 8.94 (s, 2H), 8.21 (dd, *J* = 5.1, 1.4 Hz, 1H), 7.78 (s, 3H), 7.31 (dd, *J* = 7.9, 5.1 Hz, 1H), 5.15 (s, 2H).

#### 2-(4-Methylimidazo­[4,5-*c*]­pyridin-1-yl)-*N*-(2,4,6-trichlorophenyl)­acetamide (**8m**)

66 mg, 30%, Condition e2. LCMS: *R*_T_ = 0.731 min, >98%@215 and 254 nm, *m*/*z* = 368.01 [M + H]^+^. ^1^H NMR (400 MHz, CD_3_OD) δ 8.77 (s, 1H), 8.48 (d, *J* = 6.4 Hz, 1H), 8.01 (d, *J* = 6.4 Hz, 1H), 7.62 (s, 2H), 5.59 (s, 2H), 3.09 (s, 3H).

#### 2-(4,6-Dimethylimidazo­[4,5-*c*]­pyridin-1-yl)-*N*-(2,4,6-trichlorophenyl)­acetamide (**8n**)

Fifteen mg, 21%, Condition e2. LCMS: *R*_T_ = 0.789 min, >98%@215 and 254 nm, *m*/*z* = 383.2 [M + H]^+^. ^1^H NMR (400 MHz, DMSO-*d*_6_) δ 10.51 (s, 1H), 8.23 (s, 1H), 7.76 (s, 2H), 7.19 (s, 1H), 5.21 (s, 2H), 2.66 (s, 3H), 2.50 (s, 6H).

#### Methyl 4-(2-(2-Amino-4-methyl-1*H*-benzo­[*d*]­imidazol-1-yl)­acetamido)-3,5-dichlorobenzoate (**9a**)

390 mg, 70%, Condition f. LCMS: *R*_T_ = 0.924 min, >95%@215 and 254 nm, *m*/*z* = 407.3 [M + H]^+^. ^1^H NMR (400 MHz, DMSO-*d*_6_) δ 7.95 (s, 2H), 6.92 (dd, *J* = 6.5, 2.5 Hz, 1H), 6.84–6.74 (m, 2H), 6.40 (s, 2H), 4.86 (s, 2H), 3.86 (s, 3H), 2.36 (s, 3H).

#### 4-(2-(2-Amino-4-methyl-1*H*-benzo­[*d*]­imidazol-1-yl)­acetamido)-*N*-benzyl-3,5-dichlorobenzamide (**9b**)

42 mg, 65%, Condition f. LCMS: *R*_T_ = 1.394 min, >95%@215 and 254 nm, *m*/*z* = 483.0 [M + H]^+^; ^1^H NMR (400 MHz, DMSO-*d*_6_) δ10.68 (s, 1H), 9.28 (t, *J* = 8 Hz, 1H), 8.59 (s, 2H), 8.03 (s, 2H), 7.32 (m, 3H), 7.25–7.27 (m, 1H), 7.20 (d, *J* = 4 Hz, 2H), 7.11 (t, *J* = 4 Hz, 1H), 5.18 (s, 2H), 4.47 (d, *J* = 4 Hz, 2H), 2.45 (s, 3H).

#### 2-(2-Amino-4-methyl-1*H*-benzo­[*d*]­imidazol-1-yl)-*N*-(2,6-dichloro-4-(2-phenoxyacetyl)­phenyl)­acetamide (**9c**)

60 mg, 37%, Condition f. LCMS: *R*_T_ = 1.014 min, >95%@215 and 254 nm, *m*/*z* = 483.3 [M + H]^+^. ^1^H NMR (400 MHz, DMSO-*d*_6_) δ 10.74 (s, 1H), 8.59 (s, 2H), 8.14 (s, 2H), 7.34–7.19 (m, 4H), 7.13 (q, *J* = 4.5 Hz, 1H), 7.04–6.92 (m, 3H), 5.59 (s, 2H), 5.20 (s, 2H), 2.46 (s, 3H).

#### 2-Bromo-*N*-(2,6-dichloro-4-(ethoxymethyl)­phenyl)­acetamide (**9d**)

Twenty-five mg, 45%, Condition f. LCMS: *R*_T_ = 1.540 min, >95%@215 and 254 nm, *m*/*z* = 408.1 [M + H]^+^; ^1^H NMR (400 MHz, DMSO-*d*_6_) δ 10.49 (s, 1H), 8.74 (s, 2H), 7.47 (s, 2H), 7.19 (m, 2H), 7.10 (t, *J* = 4 Hz, 1H), 5.16 (s, 2H), 4.46 (s, 2H), 3.50 (q, *J* = 8 Hz, 2 H), 2.44 (s, 3H), 1.15 (t, *J* = 4 Hz, 3H).

#### 2-(2-Amino-4-methylbenzimidazol-1-yl)-*N*-[2,6-dichloro-4-(propan-2-yloxymethyl)­phenyl]­acetamide (**9e**)

40 mg, 70%, Condition f. LCMS: *R*_T_ = 1.540 min, >95%@215 and 254 nm, *m*/*z* = 408.1 [M + H]^+^; ^1^H NMR (400 MHz, DMSO-*d*_6_) δ 10.24 (s, 1H), 8.15 (s, 1H), 7.46 (s, 2H), 6.93–6.95 (m, 1H), 6.81–6.85 (m, 2H), 6.69 (br, 2H), 4.92 (s, 2H), 4.46 (s, 2H), 3.61–3.67 (m, 1H), 2.07 (s, 3H), 1.13 (d, *J* = 8 Hz, 6H).

#### Methyl 4-(2-(2-Amino-4-methyl-1*H*-benzo­[*d*]­imidazol-1-yl)­acetamido)-3-chloro-2-methylbenzoate (**9g**)

36 mg, 33%, Condition f. LCMS: *R*_T_ = 0.902 min, >98%@215 and 254 nm, *m*/*z* = 387.0 [M + H]^+^. ^1^H NMR (400 MHz, DMSO-*d*_6_) δ 9.80 (s, 1H), 7.92 (d, *J* = 8.7 Hz, 1H), 7.74 (d, *J* = 8.7 Hz, 1H), 6.94–6.87 (m, 1H), 6.79 (q, *J* = 3.6 Hz, 2H), 6.44 (s, 2H), 4.99 (s, 2H), 3.83 (s, 3H), 2.58 (s, 3H), 2.38 (s, 3H).

#### 2-(2-Amino-4-methyl-1*H*-benzo­[*d*]­imidazol-1-yl)-*N*-(2-chloro-3-methyl-4-(2-oxo-2-(phenylamino)­ethyl)­phenyl)­acetamide (**9i**)

65 mg, 41%, Condition f. LCMS: *R*_T_ = 1.009 min, >98%@215 and 254 nm, *m*/*z* = 462.4 [M + H]^+^. ^1^H NMR (400 MHz, DMSO-*d*_6_) δ 10.18 (s, 1H), 10.06 (s, 1H), 8.55 (s, 2H), 7.61–7.47 (m, 3H), 7.36–7.16 (m, 5H), 7.15–7.00 (m, 2H), 5.15 (s, 2H), 3.77 (s, 2H), 2.45 (s, 3H), 2.35 (d, *J* = 6.1 Hz, 3H).

#### *N*-(4-(2-(2-Amino-4-methyl-1*H*-benzo­[*d*]­imidazol-1-yl)­acetamido)-3-chloro-2-methylbenzyl)­benzamide (**9j**)

25 mg, 27%, Condition f. LCMS: *R*_T_ = 0.982 min, >98%@215 and 254 nm, *m*/*z* = 462.2 [M + H]^+^. ^1^H NMR (400 MHz, DMSO-*d*_6_) δ 10.07 (s, 1H), 8.97 (t, *J* = 5.7 Hz, 1H), 8.53 (s, 2H), 7.90–7.83 (m, 2H), 7.58–7.43 (m, 4H), 7.29–7.15 (m, 3H), 7.11 (d, *J* = 7.4 Hz, 1H), 5.13 (s, 2H), 4.48 (d, *J* = 5.7 Hz, 2H), 2.44 (s, 3H), 2.40 (s, 3H).

#### 4-(2-(2-Amino-4-methyl-1*H*-benzo­[*d*]­imidazol-1-yl)­acetamido)-*N*-benzyl-3-chloro-2-methylbenzamide (**9k**)

22 mg, 16%, Condition f. LCMS: *R*_T_ = 0.986 min, >95%@215 and 254 nm, *m*/*z* = 462.4 [M + H]^+^. ^1^H NMR (400 MHz, DMSO-*d*_6_) δ 10.15 (s, 1H), 8.93 (t, *J* = 6.1 Hz, 1H), 8.52 (s, 2H), 7.65 (d, *J* = 8.3 Hz, 1H), 7.39–7.22 (m, 7H), 7.19 (t, *J* = 7.7 Hz, 1H), 7.11 (d, *J* = 7.5 Hz, 1H), 5.16 (s, 2H), 4.43 (d, *J* = 6.0 Hz, 2H), 2.45 (s, 3H), 2.36 (s, 3H).

#### *N*-(4-(2-(2-Amino-4-methyl-1*H*-benzo­[*d*]­imidazol-1-yl)­acetamido)-3,5-dichloro-2-methylbenzyl)­benzamide (**9l**)

47 mg, 85%, Condition f. LCMS: *R*_T_ = 0.960 min, >98%@215 and 254 nm, *m*/*z* = 497.4 [M + H]^+^. ^1^H NMR (400 MHz, DMSO-*d*_6_) δ 10.46 (s, 1H), 9.03 (t, *J* = 4 Hz, 1H), 8.66 (s, 1H), 7.88 (d, *J* = 8 Hz, 2H), 7.54 (m, 1H), 7.46–7.50 (m, 2H), 7.37 (s, 1H), 7.20 (m, 2H), 7.11 (m, 1H), 5.15 (s, 2H), 4.49 (d, *J* = 4 Hz, 2H), 2.44 (s, 3H), 2.38 (s, 3H).

### Synthetic Procedure for the Preparation of Analog **7l**

#### 3-Cyclopropylbenzene-1,2-diamine (**19**)

A mixture of 3-bromobenzene-1,2-diamine (250 mg, 1.34 mmol), potassium cyclopropyltrifluoroborate (396 mg, 2.67 mmol), Pd­(OAc)_2_ (45 mg, 0.20 mmol), Cy_3_P (112 mg, 0.40 mmol), and Cs_2_CO_3_ (1.31 g, 4.0 mmol) in toluene (3.0 mL) and H_2_O (1.0 mL) was degassed with N_2_ for 15 min. The reaction mixture was heated at reflux (oil bath, 120 °C) overnight. After cooling to room temperature, the mixture was diluted with EtOAc (20 mL) and H_2_O (20 mL), then filtered through Celite. The filter cake was washed with EtOAc (3 × 10 mL), and the combined filtrates were transferred to a separatory funnel. The organic layer was washed with brine (10 mL), dried over Na_2_SO_4_, filtered, and concentrated under reduced pressure to afford crude 3-cyclopropylbenzene-1,2-diamine, which was used directly in the next step without purification. LCMS: *R*_T_ = 0.320 min, >90%@215 and 254 nm, *m*/*z* = 149.3 [M + H] ^+^.

#### 4-Cyclopropyl-1*H*-benzo­[*d*]­imidazol-2-amine (20)

Crude 3-cyclopropylbenzene-1,2-diamine (36 mg, 0.24 mmol) was dissolved in MeOH (2.5 mL, 0.5–1.0 M). Cyanogen bromide (35.7 mg, 0.24 mmol) was added, and the reaction was stirred overnight at room temperature. The mixture was concentrated under reduced pressure to yield crude 4-cyclopropyl-1*H*-benzo­[*d*]­imidazol-2-amine which was used directly in the next step without purification. LCMS: *R*_T_ = 0.715 min, >90%@215 and 254 nm, *m*/*z* = 174.4 [M + H] ^+^.

#### 2-(2-Amino-4-cyclopropyl-1*H*-benzo­[*d*]­imidazol-1-yl)-*N*-(2,4,6-trichlorophenyl)­acetamide (**7l**)

To a solution containing 4-cyclopropyl-1*H*-benzo­[*d*]­imidazol-2-amine (25.0 mg, 0.31 mmol) in EtOH (3.0 mL) at rt was added 2-bromo-*N*-(2,4,6-trichlorophenyl)­acetamide (99.7 mg, 0.31 mmol). The reaction mixture was heated at 90 °C for 16 h and concentrated under reduced pressure. The crude was purified by silica-gel chromatography (10% MeOH/DCM) to give 2-(2-amino-4-cyclopropyl-1*H*-benzo­[*d*]­imidazol-1-yl)-*N*-(2,4,6-trichlorophenyl)­acetamide (15.0 mg, 25%) as a white solid. LCMS: *R*_T_ = 1.670 min, >95%@215 and 254 nm, *m*/*z* = 410.1 [M + H] ^+^; 1H NMR (400 MHz, DMSO-*d*_6_) δ 10.53 (s, 1H), 8.66 (s, 2H), 7.77 (s, 2H), 7.17 (m, 2H), 6.82 (d, *J* = 8 Hz, 1H), 5.16 (s, 2H), 2.20 (dd, *J* = 4 Hz, 1H), 1.05 (m, 2H), 0.79 (m, 2H).

### Synthetic Procedure for the Preparation of Analogs **9f** and **9h**

#### 2-(2-Amino-4-methyl-1*H*-benzo­[*d*]­imidazol-1-yl)-*N*-(2,6-dichloro-4-(hydroxymethyl)­phenyl)­acetamide (**21**)

To a stirred solution containing LiAlH_4_ (43.1 mg, 1.13 mmol) in tetrahydrofuran (10 mL) at room temperature, a solution containing methyl 4-(2-(2-amino-4-methyl-1*H*-benzo­[*d*]­imidazol-1-yl)­acetamido)-3,5-dichlorobenzoate (**9a**) (350 mg, 859 μmol) in THF (10 mL) was added dropwise. The resulting mixture was heated at reflux for 2 h and the excess hydride was quenched by sequential addition of water (1 mL), 4 N aqueous NaOH (1 mL), and brine (2 mL). The mixture was then filtered through a Celite pad and washed with ethyl acetate. The organic solvent was concentrated under reduced pressure, and the residue was washed multiple times with diethyl ether. The resulting solid precipitate 2-(2-amino-4-methyl-1*H*-benzo­[*d*]­imidazol-1-yl)-*N*-(2,6-dichloro-4-(hydroxymethyl)­phenyl)­acetamide was collected by filtration and used directly in the subsequent step without further purification. LCMS: *R*_T_ = 0.808 min, >90% 215 and 254 nm, *m*/*z* = 397.3 [M + H]^+^.

#### 2-(2-Amino-4-methyl-1*H*-benzo­[d]­imidazol-1-yl)-*N*-(2-chloro-4-(hydroxymethyl)-3-methylphenyl)­acetamide (**9h**)

Compound **9h** was prepared analogously to the procedure described above using **9g** as the starting material and purified by RP-HPLC using a gradient (5–95%, H_2_O/ACN, 0.1% TFA) to afford the title compound (13.3 mg, 57%). LCMS: *R*_T_ = 0.761 min, >98%@215 and 254 nm, *m*/*z* = 359.3 [M + H]^+^. ^1^H NMR (400 MHz, DMSO-*d*_6_) δ 10.04 (s, 1H), 8.58 (s, 2H), 7.53 (d, *J* = 8.3 Hz, 1H), 7.35–7.24 (m, 2H), 7.20 (t, *J* = 7.7 Hz, 1H), 7.12 (d, *J* = 7.5 Hz, 1H), 5.22 (s, 1H), 5.15 (s, 2H), 4.50 (s, 2H), 2.46 (s, 3H), 2.32 (s, 3H).

#### 2-(2-Amino-4-methyl-1*H*-benzo­[*d*]­imidazol-1-yl)-*N*-(4-(bromomethyl)-2,6-dichlorophenyl)­acetamide (**22**)

Phosphorus tribromide (PBr_3_, 58 mg, 20 μL, 0.21 mmol) was added dropwise to a 0 °C solution containing 2-(2-amino-4-methyl-1*H*-benzo­[*d*]­imidazol-1-yl)-*N*-(2,6-dichloro-4-(hydroxymethyl)­phenyl)­acetamide (90 mg, 0.24 mmol) in DCM (10 mL). The reaction mixture was allowed to warm to room temperature and stirred overnight. Water was carefully added to the mixture, and the layers were separated. The organic layer was washed with brine, dried over anhydrous Na_2_SO_4_ and concentrated under reduced pressure. The crude product was used directly in the next step without further purification. LCMS: *R*_T_ = 0.971 min, >85%@215 and 254 nm, *m*/*z* = 441.2 [M + H]^+^.

#### 2-(2-Amino-4-methyl-1*H*-benzo­[*d*]­imidazol-1-yl)-*N*-(2,6-dichloro-4-((2-oxopyrrolidin-1-yl)­methyl)­phenyl)­acetamide (**9f**)

To a suspension of sodium hydride (NaH, 6.9 mg, 60 wt %, 0.17 mmol) in tetrahydrofuran (5 mL) at 0 °C, pyrrolidin-2-one (13 mg, 12 μL, 0.16 mmol) was added. The reaction mixture was allowed to stir at 0 °C for 30 min, then warmed to room temperature and stirred for an additional 30 min until hydrogen gas (H_2_) evolution ceased. A solution containing 2-(2-amino-4-methyl-1*H*-benzo­[*d*]­imidazol-1-yl)-*N*-(4-(bromomethyl)-2,6-dichlorophenyl)­acetamide (70 mg, 0.16 mmol) in THF was added dropwise, and the reaction was allowed to stir at room temperature for 12 h. The reaction was quenched by the addition of water (10 mL) and extracted with 10% methanol in dichloromethane (3 × 15 mL). The combined organic layers were dried over Na_2_SO_4_, concentrated under reduced pressure, and purified by reverse-phase high-performance liquid chromatography (RP-HPLC, using water/ACN with 0.1% TFA as eluent). The product, 2-(2-amino-4-methyl-1*H*-benzo­[*d*]­imidazol-1-yl)-*N*-(2,6-dichloro-4-((2-oxopyrrolidin-1-yl)­methyl)­phenyl)­acetamide, was isolated as a white solid (35 mg, 39%). LCMS: *R*_T_ = 0.831 min, >98%@215 and 254 nm, *m*/*z* = 446.3 [M + H]^+^; ^1^H NMR (400 MHz, DMSO-*d*_6_) δ 10.46 (s, 1H), 8.58 (s, 2H), 7.39 (s, 2H), 7.24–7.07 (m, 3H), 5.15 (s, 2H), 4.37 (s, 2H), 3.28 (m, 2H) 2.44 (s, 3H), 2.30 (t, *J* = 8.1 Hz, 2H), 1.94 (p, *J* = 7.6 Hz, 2H).

### Synthetic Procedure for the Preparation of Analogs **10a**–**c**

#### 2-(2-Amino-4-((4-methylpiperazin-1-yl)­methyl)-1*H*-benzo­[*d*]­imidazol-1-yl)-*N*-(2,4,6-trichlorophenyl)­acetamide (**10a**)

A mixture of 2-(2-amino-4-bromo-1*H*-benzo­[*d*]­imidazol-1-yl)-*N*-(2,4,6-trichlorophenyl)­acetamide (**23**) (30 mg, 66.9 μmol) in THF:H_2_O (10:1, 1 mL) was placed in a microwave vial. Potassium trifluoro­((4-methylpiperazin-1-yl)­methyl)­borate (17.7 mg, 80.3 μmol), PdCl_2_(dtbpf) (5 mg, 0.1 equiv), and Cs_2_CO_3_ (43.6 mg, 133.8 μmol) were added, and the mixture was degassed with nitrogen before sealing. The reaction was heated at 100 °C for 16 h, then cooled to room temperature. The crude mixture was filtered and purified by RP-HPLC (5–95% H_2_O/ACN, 0.1% TFA) to afford 2-(2-amino-4-((4-methylpiperazin-1-yl)­methyl)-1*H*-benzo­[*d*]­imidazol-1-yl)-*N*-(2,4,6-trichlorophenyl)­acetamide (3.2 mg, 10% yield). LCMS: *R*_T_ = 1.344 min, >98%@215 and 254 nm, *m*/*z* = 482.1 [M + H]^+^; ^1^H NMR (400 MHz, DMSO-*d*_6_) δ 10.34 (s, 1H), 7.78 (s, 2H), 6.98 (t, *J* = 8 Hz, 2H), 6.89 (d, *J* = 8 Hz, 1H), 6.64 (s, 2H), 4.94 (s, 2H), 3.78 (s, 2H), 2.57 (br, 7H), 2.36 (s, 3H).

#### 2-(2-Amino-4-(morpholinomethyl)-1*H*-benzo­[*d*]­imidazol-1-yl)-*N*-(2,4,6-trichlorophenyl)­acetamide (**10b**, 5.0 mg, 9.5%)

A procedure analogous to that used to prepare **10a** was employed, using the appropriate starting material. LCMS: *R*_T_ = 0.762 min, >98%@215 and 254 nm, *m*/*z* = 469.4 [M + H]^+^; ^1^H NMR (400 MHz, DMSO-*d*_6_) δ 10.62 (s, 1H), 8.88 (br, 2H), 7.80 (s, 2H), 7.46 (t, *J* = 4 Hz, 1H), 7.37 (d, *J* = 4 Hz, 2H), 5.24 (s, 2H), 4.47 (s, 2H), 3.18 (br, 6H).

#### Methyl 1-[[2-Amino-1-[2-oxo-2-(2,4,6-trichloroanilino)­ethyl]­benzimidazol-4-yl]­methyl]­pyrrolidine-3-carboxylate (**10c**, 7 mg, 35%)

A procedure analogous to that used to prepare **10a** was employed, using the appropriate starting material. LCMS: *R*_T_ = 1.393 min, >98%@215 and 254 nm, *m*/*z* = 506.9 [M + H]^+^; ^1^H NMR (CD_3_OD, 400 MHz) 7.61 (s, 2H), 7.56 (dd, *J* = 1.0, 7.9 Hz, 1H), 7.51 (dd, *J* = 1.1, 7.8 Hz, 1H), 7.47 (d, *J* = 7.6 Hz, 1H), 5.26 (s, 2H), 4.72 (s, 2H), 3.83–3.69 (m, 5H), 3.61–3.53 (m, 2H), 3.52–3.44 (m, 1H), 2.53–2.43 (m, 1H), 2.39–2.31 (m, 1H).

### Synthetic Procedure for the Preparation of Analogs **10d**–**j**

#### (*S*)-2-(4-((3-(Hydroxymethyl)­pyrrolidin-1-yl)­methyl)-2-methyl-1*H*-benzo­[*d*]­imidazol-1-yl)-*N*-(2,4,6-trichlorophenyl)­acetamide (**10d**)

To a solution of 2-(4-(bromomethyl)-2-methyl-1*H*-benzo­[*d*]­imidazol-1-yl)-*N*-(2,4,6-trichlorophenyl)­acetamide (**24**) (45 mg, 98.8 μmol) in DMF (1 mL) was added Cs_2_CO_3_ (64 mg, 197.7 μmol) and (*S*)-pyrrolidin-3-ylmethanol (10 mg, 98.8 μmol). The reaction mixture was allowed to stir at room temperature for 1 h. The mixture was filtered, and the collected solid was purified using RP-HPLC using a gradient (5–95%, H_2_O/ACN, 0.1% TFA), affording (*S*)-2-(4-((3-(hydroxymethyl)­pyrrolidin-1-yl)­methyl)-2-methyl-1*H*-benzo­[*d*]­imidazol-1-yl)-*N*-(2,4,6-trichlorophenyl)­acetamide (20 mg, 42% yield). LCMS: *R*_T_ = 1.286 min, >98%@215 and 254 nm, *m*/*z* = 482.1 [M + H]^+^. ^1^H NMR (400 MHz, CD_3_OD) δ 7.82 (dd, *J* = 4 Hz, *J* = 4 Hz, 1H), 7.62 (s, 2H), 7.59 (m, 2H), 7.55 (d, *J* = 8 Hz, 1H), 5.46 (s, 2H), 4.78 (s, 2H), 3,58 (m, 6H), 2.85 (s, 3H), 2.69 (br s, 1H), 2.24 (br s, 1H), 1.95 (br s, 1H).

#### (*R*)-2-(4-((3-(Hydroxymethyl)­pyrrolidin-1-yl)­methyl)-2-methyl-1*H*-benzo­[*d*]­imidazol-1-yl)-*N*-(2,4,6-trichlorophenyl)­acetamide (**10e**)

Compound **10e** was synthesized using procedures analogous to those reported for the synthesis of **10d**. (R)-2-(4-((3-(hydroxymethyl)­pyrrolidin-1-yl)­methyl)-2-methyl-1*H*-benzo­[*d*]­imidazol-1-yl)-*N*-(2,4,6-trichlorophenyl)­acetamide (35.7 mg, 75%); LCMS: *R*_T_ = 1.292 min, >98%@215 and 254 nm, *m/*z = 482.1 [M + H]^+^. ^1^H NMR (400 MHz, DMSO-*d*_6_) δ 10.53 (s, 1H), 8.23 (s, 1H), 7.78 (s, 1H), 7.44 (d, *J* = 8 Hz,1H), 7.21 (m, 2H), 5.18 (s, 2H), 4.18 (s, 4H), 3.32 (m, 2H), 2.83 (m, 3H), 2.58 (m, 3H), 2.30 (br, 1H), 1.87 (m, 1H), 1.48 (m, 1H).

#### (*R*)-2-(4-((3-(1,1-Dioxidothiomorpholino)­pyrrolidin-1-yl)­methyl)-2-methyl-1*H*-benzo­[*d*]­imidazol-1-yl)-*N*-(2,4,6-trichlorophenyl)­acetamide (**10g**)

Compound **10g** was synthesized using procedures analogous to those reported for the synthesis of **10d**. (R)-2-(4-((3-(1,1-dioxidothiomorpholino)­pyrrolidin-1-yl)­methyl)-2-methyl-1*H*-benzo­[*d*]­imidazol-1-yl)-*N*-(2,4,6-trichlorophenyl)­acetamide (14.2 mg, 45%); LCMS: *R*_T_ = 1.309 min, >98%@215 and 254 nm, *m*/*z* = 586.2 [M + H]^+^. ^1^H NMR (400 MHz, DMSO-*d*_6_) δ 10.62 (s, 1H), 7.79 (s, 2H), 7.70 (d, *J* = 8 Hz, 1H), 7.40 (m, 2H), 5.32 (s, 2H), 4.71 (s, 4H), 3.50 (m, 4H), 3.33 (m, 3H), 3.10 (m, 5H), 2.92 (m, 4H), 2.66 (s, 3H), 2.12 (br s, 2H), 1.93 (br, 2H).

#### (*S*)-2-(4-((3-(1,1-Dioxidothiomorpholino)­pyrrolidin-1-yl)­methyl)-2-methyl-1*H*-benzo­[*d*]­imidazol-1-yl)-*N*-(2,4,6-trichlorophenyl)­acetamide (**10h**)

Compound **10h** was synthesized using procedures analogous to those reported for the synthesis of **10d**. (S)-2-(4-((3-(1,1-dioxidothiomorpholino)­pyrrolidin-1-yl)­methyl)-2-methyl-1*H*-benzo­[*d*]­imidazol-1-yl)-*N*-(2,4,6-trichlorophenyl)­acetamide (15.8 mg, 50%); LCMS: *R*_T_ = 1.326 min, >98%@215 and 254 nm, *m*/*z* = 586.1 [M + H]^+^. ^1^H NMR (400 MHz, DMSO-*d*_6_) δ 10.63 (s, 1H), 7.79 (s, 2H), 7.71 (d, J = 8 Hz, 1H), 7.41 (m, 2H), 5.33 (s, 2H), 4.71 (s, 4H), 3.50 (m, 4H), 3.33 (m, 3H), 3.10 (m, 5H), 2.93 (m, 4H), 2.67 (s, 4H), 2.12 (br, 2H), 1.93 (br s, 2H).

#### 2-(2-Methyl-4-((3-(4-methylpiperidin-1-yl)­pyrrolidin-1-yl)­methyl)-1*H*-benzo­[*d*]­imidazol-1-yl)-*N*-(2,4,6-trichlorophenyl)­acetamide (**10i**)

Compound **10i** was synthesized using procedures analogous to those reported for the synthesis of **10d**. 2-(2-methyl-4-((3-(4-methylpiperidin-1-yl)­pyrrolidin-1-yl)­methyl)-1*H*-benzo­[*d*]­imidazol-1-yl)-*N*-(2,4,6-trichlorophenyl)­acetamide (26.8 mg, 40%); LCMS: *R*_T_ = 1.405 min, >98%@215 and 254 nm, *m*/*z* = 550.2 [M + H]^+^. ^1^H NMR (400 MHz, DMSO-*d*_6_) δ 10.61­(s, 1H), 7.80 (s, 2H), 7.70 (d, *J* = 8 Hz, 1H), 7.39 (m, 2H), 5.33 (s, 2H), 4.62 (s, 2H), 2.96 (br, 3H), 2.89 (s, 1H), 2.73 (s, 1H), 2.66 (s, 3H), 2.30 (m, 4H), 1.83 (br, 2H), 1.64 (s, 2H), 1.33 (s, 3H), 0.92 (d, *J* = 4 Hz, 3H).

#### 2-(4-((3-(4-Cyanopiperidin-1-yl)­pyrrolidin-1-yl)­methyl)-2-methyl-1*H*-benzo­[*d*]­imidazol-1-yl)-*N*-(2,4,6-trichlorophenyl)­acetamide (**10j**)

Compound **10j** was synthesized using procedures analogous to those reported for the synthesis of **10d**. 2-(4-((3-(4-cyanopiperidin-1-yl)­pyrrolidin-1-yl)­methyl)-2-methyl-1*H*-benzo­[*d*]­imidazol-1-yl)-*N*-(2,4,6-trichlorophenyl)­acetamide (48.4 mg, 35%); LCMS: *R*_T_ = 1.279 min, >98%@215 and 254 nm, *m*/*z* = 561.1 [M + H]^+^. ^1^H NMR (400 MHz, DMSO-*d*_6_) δ 10.66 (s, 1H), 7.79 (s, 1H), 7.74 (d, *J* = 8 Hz, 1H), 7.43 (m, 2H), 5.36 (s, 2H), 4.643 (s, 3H), 3.97 (br, 1H), 3.65 (d, *J* = 12 Hz, 1H), 3.51 (dd, *J* = 4, 8 Hz, 1H), 3.27 (m, 7H), 2.69 (s, 3H), 2.32 (m, 1H), 2.15 (m, 3H), 1.93 (br s, 2H).

### Synthetic Procedure for the Preparation of Analog **10f**

#### 2-(2-Amino-4-formyl-1*H*-benzo­[*d*]­imidazol-1-yl)-*N*-(2,4,6-trichlorophenyl)­acetamide (**26**)

To a solution containing 2-(2-amino-4-vinyl-1*H*-benzo­[*d*]­imidazol-1-yl)-*N*-(2,4,6-trichlorophenyl)­acetamide (**25**) (200.0 mg, 0.5 mmol) in a mixture of 1,4-dioxane (8 mL) and water (2 mL) in a 20 mL vial at rt was added OsO_4_ (0.16 mL, 0.025 mmol, 4 wt % in water) and NaIO_4_ (245.0 mg, 1.15 mmol). The mixture was allowed to stir for 6h at rt, then quenched by the addition of a saturated aqueous NaHCO_3_ solution. The aqueous layer was extracted with 10% MeOH/DCM solution. The combined organic layers were dried over anhydrous Na_2_SO_4_, filtered, and concentrated under reduced pressure to provide the crude 2-(2-amino-4-formyl-1*H*-benzo­[*d*]­imidazol-1-yl)-*N*-(2,4,6-trichlorophenyl)­acetamide which was used for next step without purification. R_t_ = 0.644 min, *m*/*z* = 397.1 [M + H]^+^.

IMPORTANT SAFETY NOTE: Osmium tetroxide (OsO_4_) is acutely toxic and a severe irritant. Caution should be exercised in its use, and we urge all researchers to consult Safety Data Sheets and use this reagent in a manner that is consistent with recommended safety practices.

#### 2-(2-Amino-4-((3-morpholinopyrrolidin-1-yl)­methyl)-1*H*-benzo­[*d*]­imidazol-1-yl)-*N*-(2,4,6-trichlorophenyl)­acetamide (**10f**)

To a solution containing 2-(2-amino-4-formyl-1*H*-benzo­[*d*]­imidazol-1-yl)-*N*-(2,4,6-trichlorophenyl)­acetamide (10.0 mg, 0.022 mmol) in DMF (1.5 mL) at rt was added 4-(pyrrolidin-3-yl)­morpholine (9.6 mg, 0.045 mmol) and AcOH (2.7 μL, 0.045 mmol). The reaction mixture was heated at 50 °C for 1 h, then cooled to rt, and NaBH­(OAc)_3_ (14.0 mg, 0.066 mmol) was added. The reaction mixture was allowed to stir for 16 h at rt, then quenched by the addition of MeOH (0.3 mL), dissolved in DMSO, and purified by HPLC to provide 2-(2-amino-4-((3-morpholinopyrrolidin-1-yl)­methyl)-1*H*-benzo­[*d*]­imidazol-1-yl)-*N*-(2,4,6-trichlorophenyl)­acetamide (3.8 mg, 38%). LCMS: *R*_T_ = 1.244 min, >98%@215 and 254 nm, *m*/*z* = 537.01 [M + H]^+^; ^1^H NMR (CD_3_OD, 400 MHz) 7.61 (s, 2H), 7.49 (dd, *J* = 1.6, 7.7 Hz, 1H), 7.44–7.38 (m, 2H), 5.24 (s, 2H), 4.43 (s, 2H), 3.91–3.84 (m, 5H), 3.55–3.40 (m, 3H), 3.23–3.12 (m, 5H), 2.53–2.43 (m, 1H), 2.31–2.22 (m, 1H).

### Synthetic Procedure for the Preparation of Analogs **10k**, **10m**–**o**

#### 2-(4-((1*S*,2*S*)-2-(Hydroxymethyl)­cyclopropyl)-2-methyl-1*H*-benzo­[*d*]­imidazol-1-yl)-*N*-(2,4,6-trichlorophenyl)­acetamide (**10k**)

To a THF (6.0 mL) solution containing (1*S*,2*S*)-2-(2-methyl-1-(2-oxo-2-((2,4,6-trichlorophenyl)­amino)­ethyl)-1*H*-benzo­[*d*]­imidazol-4-yl)­cyclopropane-1-carboxylate (**27**) (197.0 mg, 0.41 mmol) at 0 °C was added LiAlH_4_ (0.31 mL, 0.61 mmol, 2 M solution in THF). The reaction mixture was allowed to warm to rt and stirred for 1.0 h. The reaction was cooled in an ice-bath and carefully quenched by the addition of wet Na_2_SO_4_ and drops of H_2_O. The solution became clear and was extracted with EtOAc. The combined organic laters were concentrated to a crude oil, which was purified by silica gel chromatography to give 2-(4-((1*S*,2*S*)-2-(hydroxymethyl)­cyclopropyl)-2-methyl-1*H*-benzo­[*d*]­imidazol-1-yl)-*N*-(2,4,6-trichlorophenyl)­acetamide (190.0 mg, 90%). LCMS: *R*_T_ = 0.678 min, >98%@215 and 254 nm, *m*/*z* = 440.1 [M + H]^+^.^1^H NMR (CD_3_OD, 400 MHz) δ 7.42 (s, 2H), 7.21–7.14 (m, 2H), 6.74 (d, *J* = 7.3 Hz, 1H), 7.01 (s, 2H), 3.79 (dd, *J* = 5.5, 11.1 Hz, 1H), 3.44 (dd, *J* = 7.8, 11.5 Hz, 1H), 2.65 (s, 3H), 2.43–2.38 (m, 1H), 1.43–1.34 (m, 1H), 1.20–1.15 (m, 1H), 0.99–0.94 (m, 1H).

#### 2-(4-((1*S*,2*S*)-2-Formylcyclopropyl)-2-methyl-1*H*-benzo­[*d*]­imidazol-1-yl)-*N*-(2,4,6-trichlorophenyl)­acetamide (**29**)

To a solution containing 2-(4-((1*S*,2*S*)-2-(hydroxymethyl)­cyclopropyl)-2-methyl-1*H*-benzo­[*d*]­imidazol-1-yl)-*N*-(2,4,6-trichlorophenyl)­acetamide (43.0 mg, 0.098 mmol) in CH_2_Cl_2_ (3.0 mL) at 0 °C was added Dess-Martin Periodinane (66.5 mg, 0.15 mmol). The reaction mixture was allowed to stir at rt for 5 h and quenched by the addition of saturated aqueous Na_2_S_2_O_3_ and NaHCO_3_. The aqueous layer was extracted with 10% MeOH/CH_2_Cl_2_. The combined organic layers were dried over anhydrous Na_2_SO_4_, filtered, and concentrated to provide the crude 2-(4-((1*S*,2*S*)-2-formylcyclopropyl)-2-methyl-1*H*-benzo­[*d*]­imidazol-1-yl)-*N*-(2,4,6-trichlorophenyl)­acetamide (40.0 mg) which was immediately used for the next step. R_t_ = 0.693 min, 1 min method, *m*/*z* = 438.1 [M + H]^+^.

#### 2-(2-Methyl-4-((1*S*,2*S*)-2-(morpholinomethyl)­cyclopropyl)-1*H*-benzo­[*d*]­imidazol-1-yl)-*N*-(2,4,6-trichlorophenyl)­acetamide (**10m**)

To a solution of 2-(4-((1*S*,2*S*)-2-formylcyclopropyl)-2-methyl-1*H*-benzo­[*d*]­imidazol-1-yl)-*N*-(2,4,6-trichlorophenyl)­acetamide (20.0 mg, 0.045 mmol) and DMF (2.0 mL) was added morpholine (10 μL, 0.091 mmol) and AcOH (7.0 μL, 0.045 mmol). The reaction mixture was allowed to stir for 1 h at rt, then NaBH­(OAc)_3_ (28.6 mg, 0.135 mmol) was added. The reaction mixture was allowed to stir for a further 3 h at rt, then quenched by the addition of MeOH (0.3 mL), dissolved in DMSO, directly purified by HPLC to provide 2-(2-methyl-4-((1*S*,2*S*)-2-(morpholinomethyl)­cyclopropyl)-1*H*-benzo­[*d*]­imidazol-1-yl)-*N*-(2,4,6-trichlorophenyl)­acetamide (9.0 mg, 39%). LCMS: *R*_T_ = 0.634 min, >98%@215 and 254 nm, *m*/*z* = 5059.2 [M + H]^+^. ^1^H NMR (CD_3_OD, 400 MHz) δ 7.66 (d, *J* = 8.4 Hz, 1H), 7.62 (s, 2H), 7.53 (t, *J* = 7.8 Hz, 1H), 7.22 (d, *J* = 7.5 Hz, 1H), 5.52 (s, 2H), 4.03–3.88 (m, 4H), 3.54 = 3.40 (m, 4H), 2.94 (s, 3H), 2.55–2.49 (m, 1H), 1.77–1.69 (m, 1H), 1.47–1.42 (m, 1H), 1.39–1.33 (m, 1H).

#### 2-[4-[(1*S*,2*S*)-2-[(3-Hydroxypyrrolidin-1-yl)­methyl]­cyclopropyl]-2-methylbenzimidazol-1-yl]-*N*-(2,4,6-trichlorophenyl)­acetamide (**10n**, 4.3 mg, 40%)

Compound **10n** was synthesized using procedures analogous to those reported for the synthesis of compound **10m**. LCMS: *R*_T_ = 0.615 min, >98%@215 and 254 nm, *m*/*z* = 509.1 [M + H]^+^. ^1^H NMR (CD_3_OD, 400 MHz) δ 7.68 (d, J = 8.4 Hz, 1H), 7.62 (s, 2H), 7.56 (t, J = 7.75 Hz, 1H), 7.25 (d, J = 7.7 Hz, 1H), 5.55 (s, 2H), 4.59 (s, 1H), 3.59–3.48 (m, 2H), 2.96 (s, 3H), 2.55–2.48 (m, 1H), 2.39–2.24 (m, 1H), 2.14–2.05 (m, 1H), 1.80–1.71 (m, 1H), 1.43–1.32 (m, 2H).

#### 2-[2-Methyl-4-[(1*S*,2*S*)-2-(morpholin-4-ylmethyl)­cyclopropyl]­benzimidazol-1-yl]-*N*-(2,4,6-trichlorophenyl)­acetamide (**10o**, 9 mg, 36%)

Compound **10o** was synthesized using procedures analogous to those reported for the synthesis of compound **10m**. LCMS: *R*_T_ = 0.615 min, >98%@215 and 254 nm, *m*/*z* = 578.2 [M + H]^+^. ^1^H NMR (CD_3_OD, 400 MHz) δ 7.68 (d, *J* = 8.4 Hz, 1H), 7.62 (s, 2H), 7.55 (t, *J* = 7.8 Hz, 1H), 7.25 (d, *J* = 7.7 Hz, 1H), 5.54 (s, 2H), 3.88–3.70 (m, 8H), 3.60–3.50 (m, 2H), 3.15–3.01 (m, 4H), 2.95 (s, 3H), 2.56–2.49 (m, 2H), 2.37–2.27 (m, 1H), 1.83–1.74 (m, 1H), 1.42–1.36 (m, 2H).

### Synthetic Procedure for the Preparation of Analogs **10l**, **10p**–**q**

#### 2-(4-((1*S*,2*R*)-2-(Hydroxymethyl)­cyclopropyl)-2-methyl-1*H*-benzo­[*d*]­imidazol-1-yl)-*N*-(2,4,6-trichlorophenyl)­acetamide (**10l**)

To a solution containing 2-(2-methyl-4-((1*S*,2*R*)-2-(((tetrahydro-2*H*-pyran-2-yl)­oxy)­methyl)­cyclopropyl)-1*H*-benzo­[*d*]­imidazol-1-yl)-*N*-(2,4,6-trichlorophenyl)­acetamide (**28**) (124.0 mg, 0.23 mmol) and MeOH (3.0 mL) was added TsOH·H_2_O (67.8 mg, 0.35 mmol). The reaction mixture was allowed to stir for 3 h at rt, then quenched by the addition of saturated aqueous NaHCO_3_. The aqueous layer was extracted by 10% MeOH/DCM. The combined organic layers were dried over anhydrous Na_2_SO_4_, filtered, and concentrated to provide the crude material which was purified by silica-gel chromatography to give 2-(4-((1*S*,2*R*)-2-(hydroxymethyl)­cyclopropyl)-2-methyl-1*H*-benzo­[*d*]­imidazol-1-yl)-*N*-(2,4,6-trichlorophenyl)­acetamide (65.0 mg, 62%,). LCMS: *R*_T_ = 0.787 min, >98%@215 and 254 nm, *m*/*z* = 440.2 [M + H]^+^. ^1^H NMR (CD_3_OD, 400 MHz) δ 7.57 (s, 2H), 7.34 (d, *J* = 8.0 Hz, 1H), 7.21 (t, *J* = 7.57 Hz, 1H), 7.04 (d, *J* = 7.52 Hz, 1H), 5.17 (s, 2H), 3.23–3.14 (m, 2H), 2.68 (s, 3H), 2.59–2.51 (m, 1H), 1.69–1.59 (m, 1H), 1.21–1.15 (m, 1H), 1.00–0.95 (m, 1H).

#### 2-(4-((1*S*,2*R*)-2-Formylcyclopropyl)-2-methyl-1*H*-benzo­[*d*]­imidazol-1-yl)-*N*-(2,4,6-trichlorophenyl)­acetamide (**30**)

To a solution containing oxalyl chloride (18.6 μL, 0.22 mmol) and CH_2_Cl_2_ (3.0 mL) at −78 °C was added DMSO (31.3 μL, 0.44 mmol) dropwise. The reaction mixture was allowed to stir for 30 min at −78 °C, and a solution containing 2-(4-((1*S*,2*R*)-2-(hydroxymethyl)­cyclopropyl)-2-methyl-1*H*-benzo­[*d*]­imidazol-1-yl)-*N*-(2,4,6-trichlorophenyl)­acetamide (65.0 mg, 0.14 mmol) in CH_2_Cl_2_ was added. The reaction mixture was allowed to stir for 40 min at −78 °C, then Et_3_N (0.1 mL, 0.73 mmol) was added. The reaction mixture was allowed to stir for 15 min at −78 °C, then warmed to 0 °C and stirred for a further 30 min. The reaction mixture was quenched by the addition of water and the aqueous layer was extracted with 10% MeOH/DCM. The combined organic layers were dried over anhydrous Na_2_SO_4_, filtered, and concentrated to provide the crude 2-(4-((1*S*,2*R*)-2-formylcyclopropyl)-2-methyl-1*H*-benzo­[*d*]­imidazol-1-yl)-*N*-(2,4,6-trichlorophenyl)­acetamide (58.0 mg) which was used for the next step immediately. *R*_t_ = 0.805 min, 1 min method, *m*/*z* = 438.2 [M + H]^+^.

#### 2-(4-((1*S*,2*R*)-2-(((*S*)-3-(1,1-Dioxidothiomorpholino)­pyrrolidin-1-yl)­methyl)­cyclopropyl)-2-methyl-1*H*-benzo­[*d*]­imidazol-1-yl)-*N*-(2,4,6-trichlorophenyl)­acetamide (**10q**)

To a solution containing 2-(4-((1*S*,2*R*)-2-formylcyclopropyl)-2-methyl-1*H*-benzo­[*d*]­imidazol-1-yl)-*N*-(2,4,6-trichlorophenyl)­acetamide (10.0 mg, 0.022 mmol) in DMF (3.0 mL) was added (*S*)-4-(pyrrolidin-3-yl)­thiomorpholine 1,1-dioxide (12.4 mg, 0.045 mmol) and AcOH (2.7 μL, 0.045 mmol). The reaction mixture was allowed to stir for 1 h at rt, then NaBH­(OAc)_3_ (14.0 mg, 0.066 mmol) was added. The reaction mixture was allowed to stir for 5 h at rt, then quenched by the addition of MeOH (0.3 mL), dissolved in DMSO, and directly purified by RP-HPLC to provide 2-(4-((1*S*,2*R*)-2-(((*S*)-3-(1,1-dioxidothiomorpholino)­pyrrolidin-1-yl)­methyl)­cyclopropyl)-2-methyl-1*H*-benzo­[*d*]­imidazol-1-yl)-*N*-(2,4,6-trichlorophenyl)­acetamide (3.8 mg, 23%). LCMS: *R*_T_ = 0.724 min, >98%@215 and 254 nm, *m*/*z* = 624.9 [M + H]^+^; ^1^H NMR (CD_3_OD, 400 MHz) δ 7.73 (d, *J* = 8.1 Hz, 1H), 7.63 (s, 2H), 7.60–7.56 (m, 1H), 7.39 (d, *J* = 7.4 Hz, 1H), 5.56 (s, 2H), 3.11–3.02 (m, 6H), 3.00–2.89 (m, 6H), 2.78–2.70 (m, 1H), 2.61–2.53 (m, 1H), 2.26–2.17 (m, 1H), 2.05–1.93 (m, 1H), 1.84–1.75 (m, 1H), 1.58–1.51 (m, 1H), 1.43–1.31 (m, 2H).

#### 2-(2-Methyl-4-((1*S*,2*R*)-2-(morpholinomethyl)­cyclopropyl)-1*H*-benzo­[*d*]­imidazol-1-yl)-*N*-(2,4,6-trichlorophenyl)­acetamide (**10p**, 5.5 mg, 30%)

Compound **10p** was synthesized using procedures analogous to those reported for the synthesis of compound **10q**. LCMS: *R*_T_ = 0.718 min, >98%@215 and 254 nm, *m*/*z* = 509.2 [M + H]^+^. [The NMR spectrum showed 10% of the corresponding *trans* isomer **10m**]. ^1^H NMR (CD_3_OD, 400 MHz) δ 7.69 (d, *J* = 8.3 Hz, 1H), 7.62 (s, 2H), 7.54 (t, *J* = 7.5 Hz, 1H), 7.35 (d, *J* = 7.2 Hz, 1H), 5.51 (s, 2H), 3.90–3.81 (m, 4H), 3.26–3.17 (m, 4H), 2.90 (s, 3H), 2.79–2.73 (m, 1H), 2.51–2.44 (m, 1H), 1.84–1.75 (m, 1H), 1.59–1.53 (m, 1H), 1.43–1.38 (m, 1H).

### Synthetic Procedure for the Preparation of Labeled Probes **11a** and **11b**

#### Methyl 5-Amino-4-chloro-2-methylbenzoate (**32**)

To a solution of methyl 4-chloro-2-methyl-5-nitrobenzoate **31** (1.0 g, 4.35 mmol) in MeOH (20 mL) and H_2_O (2.2 mL) was added tin­(II) chloride dihydrate (3.9 g, 17.42 mmol). The reaction mixture was heated at 65 °C for 2 h. DCM (15 mL) and 3 M NaOH (10 mL) were then added, and the mixture was passed through a hydrophobic frit/phase separator. The organic layer was collected, dried over anhydrous Na_2_SO_4_, and concentrated under reduced pressure to afford methyl 5-amino-4-chloro-2-methylbenzoate (700 mg, 3.51 mmol, 80.5%) as a light-yellow solid. LCMS: *R*_T_ = 0.822 min, >98%@215 and 254 nm, *m*/*z* = 200.2 [M + H]^+^.

#### Methyl 5-(2-Bromoacetamido)-4-chloro-2-methylbenzoate (**33**)

To a solution of methyl 5-amino-4-chloro-2-methylbenzoate (750 mg, 3.76 mmol) in toluene (5 mL) was added 2-bromoacetyl chloride (645 mg, 341 μL, 4.10 mmol) dropwise. The reaction mixture was stirred at 90 °C for 12 h, then cooled to room temperature and concentrated to dryness to afford methyl 5-(2-bromoacetamido)-4-chloro-2-methylbenzoate (1.20 g, 3.70 mmol, 100%) as an off-white solid, which was used in the next step without further purification. LCMS: *R*_T_ = 0.944 min, >98%@215 and 254 nm, *m*/*z* = 320.1 [M + H]^+^.

#### Methyl 5-(2-(2-Amino-4-methyl-1*H*-benzo­[*d*]­imidazol-1-yl)­acetamido)-4-chloro-2-methylbenzoate (**34**)

To a stirred solution of 4-methyl-1*H*-benzo­[*d*]­imidazol-2-amine (300 mg, 2.04 mmol) in EtOH (3 mL) was added methyl 5-(2-bromoacetamido)-4-chloro-2-methylbenzoate (712 mg, 2.22 mmol). The reaction vessel was capped and stirred at 90 °C overnight. The crude reaction mixture was concentrated to dryness and purified by column chromatography (0–20% MeOH in DCM) to afford methyl 5-(2-(2-amino-4-methyl-1*H*-benzo­[*d*]­imidazol-1-yl)­acetamido)-4-chloro-2-methylbenzoate (410 mg, 0.95 mmol, 47%, 90% purity) as an off-white solid. LCMS: *R*_T_ = 0.964 min, >90%@215 and 254 nm, *m*/*z* = 387.3 [M + H]^+^.

#### 5-(2-(2-Amino-4-methyl-1*H*-benzo­[*d*]­imidazol-1-yl)­acetamido)-4-chloro-2-methylbenzoic Acid (**35**)

To a stirred solution of methyl 5-(2-(2-amino-4-methyl-1*H*-benzo­[*d*]­imidazol-1-yl)­acetamido)-4-chloro-2-methylbenzoate (205 mg, 530 μmol) in THF (3 mL) under an argon atmosphere was added potassium trimethylsilanolate (122 mg, 954 μmol, 1.8 equiv). The reaction mixture was stirred at room temperature overnight. Upon completion, the mixture was concentrated to dryness and purified by RP-HPLC using a gradient (5–95%, H_2_O/ACN, 0.1% TFA). The appropriate fractions were collected and concentrated to afford 5-(2-(2-amino-4-methyl-1*H*-benzo­[*d*]­imidazol-1-yl)­acetamido)-4-chloro-2-methylbenzoic acid (150 mg, 319 μmol, 60%) as the TFA salt. LCMS: *R*_T_ = 1.416 min, >95%@215 and 254 nm, *m*/*z* = 373.2 [M + H]^+^.

#### *tert*-Butyl (2-(5-(2-(2-Amino-4-methyl-1*H*-benzo­[*d*]­imidazol-1-yl)­acetamido)-4-chloro-*N*,2-dimethylbenzamido)­ethyl)­carbamate (**36**)

To a solution of 5-(2-(2-amino-4-methyl-1*H*-benzo­[*d*]­imidazol-1-yl)­acetamido)-4-chloro-2-methylbenzoic acid (60 mg, 0.15 mmol) in DMF (1 mL) was added N-ethyl-*N*-isopropylpropan-2-amine (0.21 g, 0.29 mL, 1.6 mmol) and *tert*-butyl (2-(methylamino)­ethyl)­carbamate (28 mg, 0.16 mmol). The mixture was stirred for 5 min, followed by the portionwise addition of 2-(3H-[1,2,3]­triazolo­[4,5-*b*]­pyridin-3-yl)-1,1,3,3-tetramethylisouronium hexafluorophosphate­(V) (61 mg, 0.16 mmol). The reaction mixture was stirred at room temperature overnight, then diluted with 5% DCM:MeOH (20 mL) and washed with saturated NaHCO_3_ (2 × 20 mL). The combined organic layers were concentrated, and the crude solid was adsorbed onto a plug of silica gel and purified by column chromatography (0–25% MeOH in DCM, 10 CV) to afford *tert*-butyl (2-(5-(2-(2-amino-4-methyl-1*H*-benzo­[*d*]­imidazol-1-yl)­acetamido)-4-chloro-*N*,2-dimethylbenzamido)­ethyl)­carbamate (22 mg, 42 μmol, 28%) as an off-white solid. LCMS: *R*_T_ = 1.525 min, >85%@215 and 254 nm, *m*/*z* = 529.3 [M + H]^+^.

#### 5-(2-(2-Amino-4-methyl-1*H*-benzo­[*d*]­imidazol-1-yl)­acetamido)-*N*-(2-aminoethyl)-4-chloro-*N*,2-dimethylbenzamide (**37**)

*tert*-Butyl (2-(5-(2-(2-amino-4-methyl-1*H*-benzo­[*d*]­imidazol-1-yl)­acetamido)-4-chloro-*N*,2-dimethylbenzamido)­ethyl)­carbamate (16 mg, 30 μmol) in DCM (200 μL) was treated dropwise with TFA (100 μL) and stirred at rt for 3 h. LC–MS confirmed product formation. The mixture was concentrated to dryness and used in the next step without purification. LCMS: *R*_T_ = 0.692 min, >85%@215 and 254 nm, *m*/*z* = 429.4 [M + H]^+^.

#### 5-(2-(2-Amino-4-methyl-1*H*-benzo­[*d*]­imidazol-1-yl)­acetamido)-4-chloro-*N*-(2-(3-(3′,6′-dihydroxy-3-oxo-3*H*-spiro­[isobenzofuran-1,9′-xanthen]-5-yl)­thioureido)­ethyl)-*N*,2-dimethylbenzamide--2,2,2-trifluoroacetaldehyde (**11a**)

To a reaction vial equipped with a magnetic stir bar containing 5-(2-(2-amino-4-methyl-1*H*-benzo­[*d*]­imidazol-1-yl)­acetamido)-*N*-(2-aminoethyl)-4-chloro-*N*,2-dimethylbenzamide **37** (15 mg, 28 μmol) in DMF (0.5 mL) was added 3′,6′-dihydroxy-5-isothiocyanato-3*H*-spiro­[isobenzofuran-1,9′-xanthen]-3-one (11 mg, 28 μmol), followed by *N*-ethyl-*N*-isopropylpropan-2-amine (44 mg, 61 μL, 0.34 mmol). The reaction mixture was stirred at rt for 2 h. LCMS analysis indicated complete consumption of starting material, the crude reaction mixture was purified by preparative HPLC (Gilson) to afford the desired product 5-(2-(2-amino-4-methyl-1*H*-benzo­[*d*]­imidazol-1-yl)­acetamido)-4-chloro-*N*-(2-(3-(3′,6′-dihydroxy-3-oxo-3*H*-spiro­[isobenzofuran-1,9′-xanthen]-5-yl)­thioureido)­ethyl)-*N*,2-dimethylbenzamide–2,2,2-trifluoroacetaldehyde (11 mg, 40%) as an yellow solid. LCMS: *R*_T_ = 1.555 min, >95%@215 and 254 nm, *m*/*z* = 818.2 [M + H]^+^. ^1^H NMR (400 MHz, CD_3_OD) δ 8.18–7.99 (m, 1H), 7.85–7.76 (m, 1H), 7.75–7.55 (m, 1H), 7.44 (d, *J* = 9.7 Hz, 1H), 7.29–7.00 (m, 4H), 6.72 (d, *J* = 15.0 Hz, 4H), 6.60–6.47 (m, 2H), 5.17 (s, 2H), 3.99 (s, 1H), 3.88–3.69 (m, 2H), 3.19 (s, 1H), 2.97 (s, 2H), 2.53–2.40 (m, 3H), 2.28 (d, *J* = 3.3 Hz, 3H), 2.05 (s, 1H).

#### 5-(2-(2-Amino-4-methyl-1*H*-benzo­[*d*]­imidazol-1-yl)­acetamido)-4-chloro-*N*-(2-(3-(5,5-difluoro-7-(1H-pyrrol-2-yl)-5*H*-5λ^4^,6λ^4^-dipyrrolo­[1,2-c:2′,1′-*f*]­[1,3,2]­diazaborinin-3-yl)­propanamido)­ethyl)-*N*,2-dimethylbenzamide (**11b**)

To a stirred solution of 5-(2-(2-amino-4-methyl-1*H*-benzo­[*d*]­imidazol-1-yl)­acetamido)-*N*-(2-aminoethyl)-4-chloro-*N*,2-dimethylbenzamide **37** (3.0 mg, 6.0 μmol) in DMF (1 mL) was added N,N-diisopropylethylamine (9 μL, 0.05 mmol), followed by 2,5-dioxopyrrolidin-1-yl 3-(5,5-difluoro-7-(1H-pyrrol-2-yl)-5*H*-5λ^4^,6λ^4^-dipyrrolo­[1,2-c:2′,1′-*f*]­[1,3,2]­diazaborinin-3-yl)­propanoate (3.0 mg, 8.0 μmol). The reaction mixture was stirred at rt for 2 h. The solvent was removed under reduced pressure, and the crude residue was purified by preparative reversed-phase HPLC (5–95% H_2_O/MeCN containing 0.1% TFA). Fractions containing the desired product were combined, neutralized with saturated aqueous NaHCO_3_, and extracted with CH_2_Cl_2_. The combined organic extracts were dried over anhydrous Na_2_SO_4_, filtered, and concentrated under reduced pressure to afford 5-(2-(2-amino-4-methyl-1*H*-benzo­[*d*]­imidazol-1-yl)­acetamido)-4-chloro-*N*-(2-(3-(5,5-difluoro-7-(1H-pyrrol-2-yl)-5*H*-5λ^4^,6λ^4^-dipyrrolo­[1,2-c:2′,1′-*f*]­[1,3,2]­diazaborinin-3-yl)­propanamido)­ethyl)-*N*,2-dimethylbenzamide as a blue solid (3.2 mg, 4.3 μmol, 70%). LCMS: *R*_T_ = 1.745 min, >95%@215 and 254 nm, *m*/*z* = 740.2 [M + H]^+^

## Supplementary Material





## Notes

Expanded KLHL12 transcriptomics profile, SPR-determined affinities for selected compounds, X-ray collection and refinement statistics, protein sequence analysis and homology statistics for KLHL family proteins relative to KLHL12, synthetic procedures for intermediates not described in the main text, ^1^H NMR for final compounds, and LC spectra for selected analogs (PDF)
Molecular formula strings (CSV)
