# Supplementary material for: Identification of KLHL12 Ligands Using Fragment-Based Methods
Source: J Med Chem. 2026 Mar 30;69(7):7709–31. doi: 10.1021/acs.jmedchem.5c02931 (PMC13071877; doi:10.1021/acs.jmedchem.5c02931)
Supplement: Supplementary file 1 [file jm5c02931_si_001.pdf]

Supporting Information

Identification of KLHL12 ligands using fragment-based methods

Alex G. Waterson,[1,2,*] Anish Vadukoot,[3] Somnath Jana,[4] Jianwen Cui,[3] Kelvin Luong,[3] Tyson A. Rietz,[3] Ezequiel Alejandro Madrigal-Carrillo,[3] Brian D. Lehmann,[5] John L. Sensintaffar,[3] Bin Zhao,[3] Kangsa Amporndanai,[3] Zoe A. Petros,[3] William Rush Scaggs,[3] Selena Chacon Simon[3], Rakesh H. Vekariya,[3] Kwangho Kim,[2,4] Manikandan Thangaraj,[3] Plamen P. Christov,[4] Taylor M. South,[3] Jiqing Sai,[3] Anusha Thiruvaipati,[3] Charles R. Schmidt,[6] Rebecca Eells,[6] William J. Moore,[7] Edward T. Olejniczak,[3] Jason Phan,[3] Stephen W. Fesik[1,2,3,*]

Author affiliations:

[1]Department of Pharmacology, Vanderbilt University School of Medicine, Nashville, Tennessee 37232, United States

[2]Department of Chemistry, Vanderbilt College of Arts and Sciences, Nashville, Tennessee, 37232, United States

[3]Department of Biochemistry, Vanderbilt University School of Medicine, Nashville, Tennessee 37232, United States

[4] Molecular Design and Synthesis Center, Vanderbilt Institute of Chemical Biology, Nashville, Tennessee, 37232, United States

[5] Department of Medicine, Vanderbilt University Medical Center, Nashville, TN, 37232, United States.

[6]Reaction Biology Corporation, Malvern, Pennsylvania,19355, United States

[7]NCI Center for Cancer Research, Frederick Maryland, 21701, United States

*Corresponding author email addresses:

a.waterson@vanderbilt.edu (A.G. Waterson)

stephen.fesik@vanderbilt.edu (S. W. Fesik)

Contents:

Figure S1. *Expanded KLHL12 transcriptomics profile* — *Pg S2*

Table S1. *SPR-determined affinities for selected compounds* — *Pg S2*

Table S2. *X-ray data collection and refinement statistics of KLHL12 bound with compounds.* — *Pg S3*

Table S3. *Sequence identity percentage of the Kelch domain of the KLHL family proteins to KLHL12 ranked from highest to lowest, UniProt accession codes, and available PDB entries.* — *Pg. S6*

Table S4. *Protein sequence analysis of the Kelch domain of selected KLHL family proteins compared to KLHL12.* — *Pg. S7*

References for sequence analysis — *Pg. S7*

Synthetic procedures and schemes for intermediates — *Pg S8*

Proton NMR data — *Pg S24*

LC traces for selected compounds — *Pg S79*

Figure S1. Expanded transcriptomics profile. Violin plots show the expression of KLHL12 stratified by tissue (**A**) or tumor sample (**B** type.

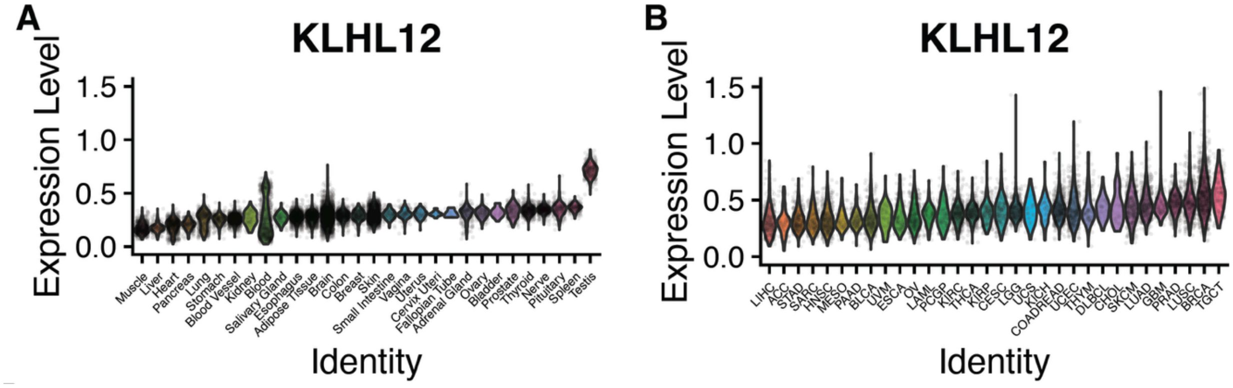

Table S1. SPR-determined affinities for selected compounds.

Cmpd	K_D (μM) [a]	k_a (1/Ms)[c]	k_d(1/s)[c]
**7a**	19.1		
**7h**	17.6		
**7j**	1.4		
**7k**	0.48	3.6 x10^5	1.4x10^{-1}
**9d**	5.6		
**9e**	4.6		
**9h**	2.3		
**9k**	1.5		
**10m**	2.4		
**10n**	1.9		
**10p**	0.34	2.9 x10^5	0.82 x10^{-1}
**10q**	0.29	3.7 x10^5	1.3 x10^{-1}
**11b**	3.6[b]		

[a] SPD K_D determined using steady state fit using His-tagged KLHL12. [b] Determined using biotin-tagged KLHL12. High binding to reference noted. [c]On- and off-rate data collected when kinetic fits were applied.

Table S2. X-ray data collection and refinement statistics of KLHL12 bound with compounds.

Compound	**1**	**7k**	**8e**	**8i**
PDB Accession code	**9Y8J**	**9Y8K**	**9Y8L**	**9Y8M**
**Data collection**				
Space Group	H 3_2	P 4_1 2_1 2	P 4_1 2_1 2	P 4_1 2_1 2
Cell Dimensions				
a, b, c (Å)	135.258, 135.258, 71.996	80.064, 80.064, 76.475	80.287, 80.287, 76.923	79.89, 79.89, 76.67
α, β, γ (°)	90.00, 90.00, 120.00	90.00, 90.00, 90.00	90.00, 90.00, 90.00	90.00, 90.00, 90.00
Resolution (Å)	29.61-2.70 (2.75-2.70)	27.65-1.27 (1.29-1.27)	27.77-1.27 (1.29-1.27)	55.32-1.33 (1.36-1.33)
R_{merge} (%)	0.117 (0.532)	0.072 (0.328)	0.102 (0.587)	0.072 (0.193)
Mean I / σI	11.3 (1.5)	47.6 (9.2)	62.2 (6.5)	22.7 (11.4)
Completeness (%)	99.9 (73.4)	99.9 (97.7)	100 (98.3)	91.4 (100)
CC1/2	0.995 (0.369)	0.996 (0.912)	1.000 (0.933)	0.998 (0.989)
Redundancy	9.7 (5.0)	6.9 (6.0)	18.8 (16.8)	12.8 (12.9)
**Structure Refinement**				
No. Reflections	7,049	66,103	66,661	52,105
R_{work}/R_{free}	0.2931/0.3291	0.1808/0.1914	0.1859/0.1927	0.1582/0.1769
R.m.s. deviations				
Bond lengths (%)	0.007	0.006	0.005	0.012
Bond angles (%)	0.952	0.936	0.886	1.806
Ramachandran				
Preferred regions (%)	91.61	97.54	98.25	97.19
Allowed regions (%)	7.34	2.46	1.75	2.81
Disallowed regions (%)	1.05	0	0	0

*High resolution shells are in parentheses.

Compound	**8m**	**9c**	**9e**	**9h**
PDB Accession code	**9Y8N**	**9Y8O**	**9Y8Q**	**9Y8R**
**Data collection**				
Space Group	P 4_1 2_1 2	P 4_1 2_1 2	P 4_1 2_1 2	P 4_1 2_1 2
Cell Dimensions				
a, b, c (Å)	79.68, 79.68, 74.53	79.67, 79.67, 77.26	79.95, 79.95, 76.80	79.78, 79.78, 76.37
α, β, γ (°)	90.00, 90.00, 90.00	90.00, 90.00, 90.00	90.00, 90.00, 90.00	90.00, 90.00, 90.00
Resolution (Å)	33.72-1.31 (1.33-1.31)	55.53-1.29 (1.31-1.29)	35.78-1.20 (1.22-1.20)	55.23-1.33 (1.37-1.33)
R_{merge} (%)	0.132 (0.561)	0.090 (0.471)	0.071 (0.366)	0.086 (0.245)
Mean I / σI	8.4 (1.0)	10.2 (4.2)	19.0 (5.8)	18.3 (8.2)
Completeness (%)	99.2 (78.6)	100 (100)	100 (99.8)	100 (100)
CC1/2	0.997 (0.382)	0.999 (0.980)	0.999 (0.965)	0.997 (0.984)
Redundancy	11.4 (10.7)	13.0 (13.4)	14.1 (13.7)	13.0 (12.9)
**Structure Refinement**				
No. Reflections	43,584	60,431	74,054	53,686
R_{work}/R_{free}	0.1586/0.2038	0.1557/0.1772	0.1326/0.1521	0.1599/0.1727
R.m.s. deviations				
Bond lengths (%)	0.012	0.012	0.012	0.012
Bond angles (%)	1.806	1.790	1.788	1.86
Ramachandran				
Preferred regions (%)	97.54	96.84	97.19	96.84
Allowed regions (%)	2.46	3.16	2.46	3.16
Disallowed regions (%)	0	0	0.35	0

Compound	**9k**	**10b**	**10j**	**10q**
PDB Accession code	**9Y8S**	**9Y8T**	**9Y8U**	**9Y8V**
**Data collection**				
Space Group	P 4_1 2_1 2	P 4_1 2_1 2	P 4_1 2_1 2	P 4_1 2_1 2
Cell Dimensions				
a, b, c (Å)	79.79, 79.79, 76.89	80.23, 80.23, 76.90	80.04, 80.04, 77.20	79.57, 79.57, 76.61
α, β, γ (°)	90.00, 90.00, 90.00	90.00, 90.00, 90.00	90.00, 90.00, 90.00	90.00, 90.00, 90.00
Resolution (Å)	56.42-1.29 (1.31-1.29)	56.73-1.40 (1.42-1.40)	56.60-1.01 (1.03-1.01)	45.35-1.36 (1.38-1.36)
R_{merge} (%)	0.067 (0.357)	0.158 (1.897)	0.010 (0.306)	0.051 (1.060)
Mean I / σI	19.7 (6.5)	10.7 (1.2)	37.6 (1.4)	25.2 (0.7)
Completeness (%)	100 (100)	99.9 (98.4)	92.8 (42.9)	96.4 (99.6)
CC1/2	0.994 (0.967)	0.997 (0.367)	1.000 (0.758)	1.000 (0.404)
Redundancy	12.6 (12.9)	13.6 (10.4)	10.9 (10.3)	10.7 (10.5)
**Structure Refinement**				
No. Reflections	60,243	50,194	120,772	56,601
R_{work}/R_{free}	0.1507/0.1701	0.1793/0.2084	0.1252/0.1411	0.1604/0.1994
R.m.s. deviations				
Bond lengths (%)	0.011	0.010	0.016	0.011
Bond angles (%)	1.789	1.756	2.025	1.700
Ramachandran				
Preferred regions (%)	96.84	97.19	96.85	96.49
Allowed regions (%)	2.81	2.81	3.15	3.51
Disallowed regions (%)	0.35	0	0	0

Table S3. Sequence identity percentage of the Kelch domain of the KLHL family proteins to KLHL12 ranked from highest to lowest, UniProt accession codes, and available PDB entries.

Protein	Sequence identity	Uniprot accession code	PDB entry	Reference
KLHL12	100	Q53G59	2VPJ	1
KLHL8	39.58	Q9P2G9	No	
KLHL20	39.58	Q9Y2M5	6GY5	2
KLHL18	38.52	O94889	No	
KLHL17	37.81	Q6TDP4	6HRL	
KLHL19 (KEAP1)	37.72	Q14145	1ZGK	3
KLHL2	37.59	O95198	4CHB	4
KLHL28	37.37	Q9NXS3	No	
KLHL1	37.10	Q9NR64	No	
KLHL3	36.52	Q9UH77	4CH9	4
KLHL5	36.40	Q96PQ7	No	
KLHL27	34.98	Q9Y573	No	
KLHL4	34.75	Q9C0H6	No	
KLHL10	34.28	Q6JEL2	No	
KLHL33	28.47	A6NCF5	No	
KLHL21	28.35	Q9UJP4	No	
KLHL41	27.94	O60662	No	
KLHL7	27.80	Q8IXQ5	3II7	1
KLHL36	27.21	Q8N4N3	No	
KLHL23	27.17	Q8NBE8	No	
KLHL24	27.04	Q6TFL4	No	
KLHL35	27.04	Q6PF15	No	
KLHL9	26.57	Q9P2J3	No	
KLHL16	26.50	Q9H2C0	No	
KLHL14	26.39	Q9P2G3	No	
KLHL13	25.93	Q9P2N7	No	
KLHL29	25.91	Q96CT2	No	
KLHL6	25.65	Q8WZ60	No	
KLHL31	25.37	Q9H511	No	
KLHL25	24.91	Q9H0H3	No	
KLHL40	24.91	Q2TBA0	4ASC	1
KLHL15	24.64	Q96M94	No	
KLHL32	24.64	Q96NJ5	No	
KLHL38	24.36	Q2WGJ6	No	
KLHL34	23.21	Q8N239	No	
KLHL30	23.08	Q0D2K2	No	
KLHL26	23.05	Q53HC5	No	
KLHL22	22.88	Q53GT1	8W4J	5
KLHL42	20.90	Q9P2K6	No	
KLHL11	16.25	Q9NVR0	No	

Table S4. Protein sequence analysis of the Kelch domain of selected KLHL family proteins compared to KLHL12.

Protein	%Identity of the Kelch domain	Loop shielding KLHL12 ligand?	%Identity of the ligand pocket	%Similarity of the ligand pocket
**KLHL12**	**100**	**No**	**100**	**100**
KLHL8	39.58	**No**	23.53	29.41
KLHL20	39.58	**No**	5.88	23.53
KLHL18	38.52	**No**	29.41	52.94
KLHL17	37.81	**No**	11.76	29.41
KLHL19 (KEAP1)	37.72	**No**	23.53	52.94
KLHL2	37.59	**No**	17.65	29.41
KLHL28	37.37	**No**	17.65	23.53
KLHL1	37.1	**Yes**	11.76	17.65
KLHL3	36.52	**No**	17.65	29.41
KLHL5	36.4	**Yes**	11.11	16.67

References for sequence analysis

1. Canning, P.; Cooper, C. D. O.; Krojer, T.; Murray, J. W.; Pike, A. C. W.; Chaikuad, A.; Keates, T.; Thangaratnarajah, C.; Hojzan, V.; Marsden, B. D.; Gileadi, O.; Knapp, S.; Von Delft, F.; Bullock, A. N. "Structural Basis for Cul3 Protein Assembly with the BTB-Kelch Family of E3 Ubiquitin Ligases" *Journal of Biological Chemistry* **2013,** *288* (11), 7803–7814. https://doi.org/10.1074/jbc.M112.437996.
2. Chen, Z.; Picaud, S.; Filippakopoulos, P.; D'Angiolella, V.; Bullock, A. N. "Structural Basis for Recruitment of DAPK1 to the KLHL20 E3 Ligase" *Structure* **2019,** *27* (9), 1395-1404.e4. https://doi.org/10.1016/j.str.2019.06.005.
3. Beamer, L. J.; Li, X.; Bottoms, C. A.; Hannink, M. "Conserved Solvent and Side-Chain Interactions in the 1.35 Å Structure of the Kelch Domain of Keap1" *Acta Crystallogr D Biol Crystallogr* **2005,** *61* (10), 1335–1342. https://doi.org/10.1107/S0907444905022626.
4. Schumacher, F. R.; Sorrell, F. J.; Alessi, D. R.; Bullock, A. N.; Kurz, T. "Structural and Biochemical Characterization of the KLHL3-WNK Kinase Interaction Important in Blood Pressure Regulation" *Biochemical Journal* **2014,** *460* (2), 237–246. https://doi.org/10.1042/BJ20140153
5. Teng, F.; Wang, Y.; Liu, M.; Tian, S.; Stjepanovic, G.; Su, M. Y. "Cryo-EM Structure of the KLHL22 E3 Ligase Bound to an Oligomeric Metabolic Enzyme" *Structure* **2023,** *31* (11), 1431-1440.e5. https://doi.org/10.1016/j.str.2023.09.002.

Synthetic procedures for intermediates not described in main text.

General experimental information: All NMR spectra were recorded at room temperature on a 400 MHz AMX Bruker spectrometer. ^1H chemical shifts are reported in δ values in ppm downfield with the deuterated solvent as the internal standard. Data are reported as follows: chemical shift, multiplicity (s = singlet, d = doublet, t = triplet, q = quartet, br = broad, m = multiplet), integration, coupling constant (Hz). Low resolution mass spectra were obtained on an Agilent 1200 series 6140 mass spectrometer with electrospray ionization. All samples were of ≥95% purity as analyzed by LC-UV/vis-MS. Analytical HPLC was performed on an Agilent 1200 series with UV detection at 214 and 254 nm along with ELSD detection. LC/MS parameters were as follows: Phenomenex-C18 Kinetex column, 50 × 2.1 mm, 2 min gradient, 5% (0.1% TFA/ACN)/95% (0.1% TFA/H_2O) to 100% (0.1% TFA/ ACN). Preparative purification was performed on a Gilson HPLC (Phenomenex-C18, 100 × 30 mm, 10 min gradient, 5 → 95% ACN/ H_2O with 0.1% TFA) or by automated flash column chromatography (Isco, Inc. 100sg Combiflash). Solvents for extraction, washing, and chromatography were HPLC grade. All reagents were purchased from chemical suppliers and used without purification.

Scheme S1. Synthesis of the aniline **39** required for **9b**.[a]

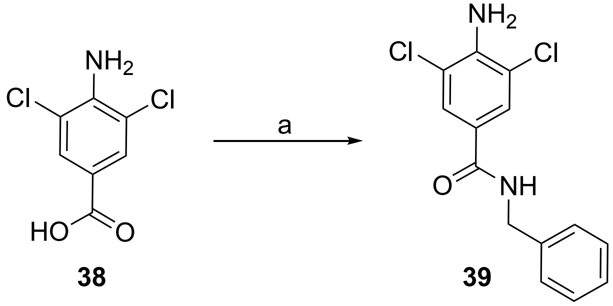

38 **39**

[a]Reagents and conditions: (a) Benzylamine, HATU, DMF, Et_3N, rt,1 h, 90%.

*4-Amino-N-benzyl-3,5-dichlorobenzamide (**39**)*: To a stirred solution of 4-amino-3,5-dichlorobenzoic acid (200 mg, 970 μmol) in DMF (5.0 mL) was added HATU (445 mg, 1.2 equiv), benzylamine (208 mg, 1.94 mmol), and triethylamine (210 μL, 1.46 mmol). The reaction mixture was allowed to stir at room temperature for 1 hour. The reaction mixture was then quenched by the addition of H_2O and extracted with ethyl acetate (3 × 5 mL). The combined organic layers were dried (Na_2SO_4), filtered, and concentrated under reduced pressure to afford the crude product 4-amino-N-benzyl-3,5-dichlorobenzamide as a solid (280 mg, 90% crude yield) which was taken to next step without further purification.

Scheme S2. Synthesis of the aniline **41** required for **9c**.[a]

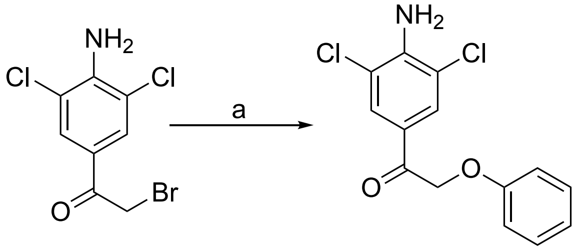

40 **41**

[a]Reagents and conditions: (a) Phenol, K_2CO_3, ACN, 50 °C, 12 h, 97%.

1-(4-Amino-3,5-dichlorophenyl)-2-phenoxyethan-1-one ***(41)***: To a solution containing 1-(4-amino-3,5-dichlorophenyl)-2-bromoethan-1-one (108 mg, 382 µmol) in ACN (3 mL) in a microwave vial was added phenol (35.9 mg, 382 µmol) and potassium carbonate (105 mg, 763 µmol). The vial was capped, and the reaction mixture was heated at 50 °C for 2 hours, then allowed to cool to room temperature, quenched by the addition of 3M NaOH (10 mL), and extracted with EtOAc (3 × 10 mL). The combined organic layers were dried over anhydrous Na_2SO_4 and concentrated to dryness under reduced pressure. The crude product, 1-(4-amino-3,5-dichlorophenyl)-2-phenoxyethan-1-one, was obtained as a solid (110 mg, 97.3%) and used directly in the next step without further purification. LCMS: R_T = 1.125 min, >85% @ 215 and 254 nm, *m/z* = 296.2 $[M + H]^+$.

Scheme S3: Synthesis of anilines **47a** and **47b** required for **9d** and **9e**.[a]

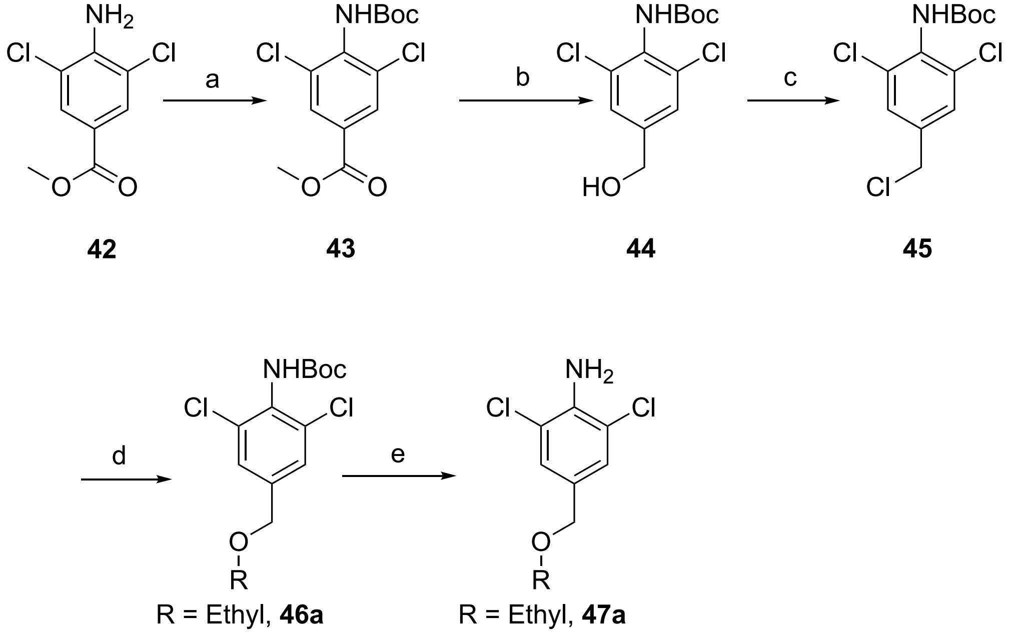

R = Ethyl, **46a**
R = isopropyl, **46b**

R = Ethyl, **47a**
R = isopropyl, **47b**

[a]Reagents and conditions: (a) Boc_2O, DMAP, THF, 41%; (b) LAH, THF, -10 °C to 0 °C; (c) $SOCl_2$, DCM, pyridine; (d) EtOH or *i*-PrOH, K_2CO_3, 60 °C, 3h, 10-25%; (e) 4.0 M HCl, DCM, 3h.

*Methyl 4-((tert-butoxycarbonyl)amino)-3,5-dichlorobenzoate (**43**)*: A solution of methyl 4-amino-3,5-dichlorobenzoate (200 mg, 909 μmol), di-*tert*-butyl dicarbonate (397 mg, 1.82 mmol) and 4-(dimethylamino)pyridine (2 eq.) in tetrahydrofuran (5 mL) was heated at reflux for 12 h. The reaction mixture was concentrated to half the volume, diluted with diethyl ether (0.15 L) and washed with a 2.0 M aqueous solution (0.10 L) of citric acid, then dried over sodium sulfate. The solvents were removed under reduced pressure and the residue was purified with silica gel chromatography (0-30% ethyl acetate/hexane) to give desired product methyl 4-((tert-butoxycarbonyl)amino)-3,5-dichlorobenzoate (120 mg, 41%). LCMS: R_T = 1.489 min, >95% @ 215 and 254 nm, *m/z* = 264.0 [M - 56]. ^{1}H NMR (400 MHz, DMSO-d_6) δ 8.07 (s, 2H), 3.90 (s, 3H), 1.35 (s, 9H).

*tert-Butyl (2,6-dichloro-4-(hydroxymethyl)phenyl)carbamate (**44**)*: A mixture containing lithium aluminum hydride (0.74 mL, 1 M in THF) and anhydrous THF (5 mL) was cooled to -10°C. A solution of methyl 4-((tert-butoxycarbonyl)amino)-3,5-dichlorobenzoate (**43**) (120 mg, 374 μmol) in anhydrous THF (1 mL) was added dropwise at -10°C over a period of 45 minutes under nitrogen. Stirring was continued at -10°C. for 1 hour. The reaction mixture was cooled to 0°C, and then 1 mL H_2O was added slowly, followed by 1 mL of 15 % aqueous sodium hydroxide. An additional 3 x 1 mL H_2O was added, and the mixture was allowed to warm to RT with stirring for 15 min. The solids were removed by filtration and washed with MeOH. The solvents were removed under reduced pressure and the crude product tert-butyl (2,6-dichloro-4-(hydroxymethyl)phenyl)carbamate was carried to the next step without further purification. LCMS: R_T = 1.025 min, >95% @ 215 and 254 nm, *m/z* = 236.2 $[M - 56]^+$; ^{1}H NMR (400 MHz, DMSO-d_6) δ 8.90 (s, 1H), 7.42 (s, 2H), 3.44 (t, *J* =4 Hz, 1H), 4.48 (d, *J* =8 Hz, 2H), 1.36 (s, 9H).

*tert-Butyl (2,6-dichloro-4-(chloromethyl)phenyl)carbamate (**45**)*: To a stirred solution of *tert*-butyl (2,6-dichloro-4-(hydroxymethyl)phenyl)carbamate (35 mg, 120 μmol) in dichloromethane (1 mL) was added pyridine (3 drops), followed by the dropwise addition of thionyl chloride (18 mmoL, 2 eq.). The resulting reaction mixture was stirred for 30 minutes, and then quenched by the addition of H_2O (2 mL). The aqueous layer was extracted with dichloromethane (2 x 5 mL), and the organic layer was dried over sodium sulfate, filtered, and concentrated under reduced pressure to afford tert-butyl (2,6-dichloro-4-(chloromethyl)phenyl)carbamate, which was used immediately in the next step without further purification.

*tert-Butyl (2,6-dichloro-4-(ethoxymethyl)phenyl)carbamate (**46a**)*: To a solution of ethanol (1 mL) was added *tert*-butyl (2,6-dichloro-4-chloromethyl)phenyl)carbamate (30 mg, 96 μmol) and K_2CO_3 (66 mg, 483 μmol). The reaction mixture was heated at 60 °C for 3 h. After reaction completion, H_2O and EtOAc was added. Organic layers were washed with H_2O and brine. The organic solvents were removed under reduced pressure and the residue was purified by silica gel chromatography (0-30% EtOAC/hexane) to afford tert-butyl (2,6-dichloro-4-(ethoxymethyl)phenyl)carbamate (17 mg, 17%); LCMS: R_T = 1.146 min, *m/z* = 220.2 [M - 100]; ^{1}H NMR (400 MHz, DMSO-d_6) δ 8.93 (s, 1H), 7.44 (s, 2H), 4.46 (s, 2H), 3.51 (q, *J* = 8Hz, 2H), 1.44 (s, 9H), 1.15 (t, *J* = 8Hz, 3H). [Isopropyl analog (**46b**) was synthesized in a similar fashion using the appropriate SM]

*2,6-Dichloro-4-(ethoxymethyl)aniline hydrochloride (**47a**)*: To a solution containing *tert*-butyl (2,6-dichloro-4-(ethoxymethyl)phenyl)carbamate (40 mg, 125 µmol) in DCM (1 mL) was added 4.0 M HCl (156 µL, 625 µmol). The reaction mixture was allowed to stir for 3 h, and the solvent was removed under reduced pressure to afford 2,6-dichloro-4-(ethoxymethyl)aniline hydrochloride, which was carried on to the next step without purification. [Isopropyl analog (**47b**) was synthesized in a similar fashion using the appropriate SM]

Scheme S4. Synthesis of the aniline **49** required for **9g** and **9h**.[a]

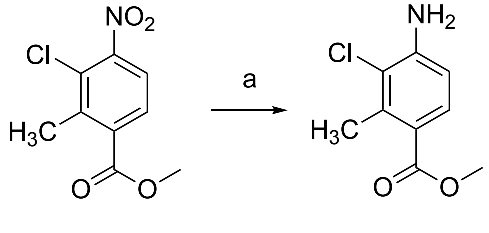

48 **49**

[a]Reagents and conditions: (a) $SnCl_2$, EtOH, H_2O, 65 °C, 1h, 86%.

*Methyl 4-amino-3-chloro-2-methylbenzoate (**49**)*: To a solution containing methyl 3-chloro-2-methyl-4-nitrobenzoate (80 mg, 0.35 mmol), EtOH (3 mL), and H_2O (0.3 mL) was added tin(II) chloride dihydrate (0.31 g, 1.4 mmol). The reaction mixture was heated at 65 °C for 1 hour., then diluted with DCM (15 mL) and 3 M aqueous NaOH (10 mL). The resulting mixture was passed through a hydrophobic frit/phase separator to isolate the organic layer. The organic phase was collected, dried over anhydrous Na_2SO_4, and concentrated under reduced pressure to yield methyl 4-amino-3-chloro-2-methylbenzoate, (60 mg, 86%) as a white solid. The product was used directly in the next step without further purification. LCMS: R_T = 0.902 min, >95% @ 215 and 254 nm, m/z = 200.2 $[M + H]^+$.

Scheme S5: Synthesis of the aniline **54** required for **9i.**[a]

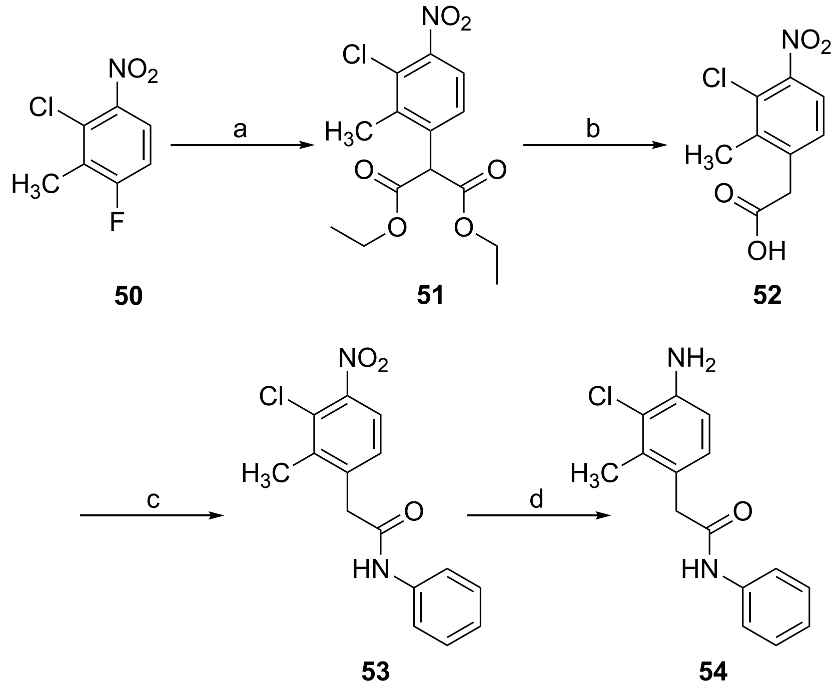

[a]Reagents and conditions: (a) diethyl malonate, NaH, 0-50 °C, 12 h, 47%; (b) 5 M NaOH:MeOH (1:3.5), 70 °C, 12 h, 69%; (c) aniline, TEA, T3P (50% weight solution in DCE), rt, 1 h, 45%; (d) $SnCl_2$, EtOH: H_2O (10:1), 65 °C, 12 h, 87%.

*Diethyl 2-(3-chloro-2-methyl-4-nitrophenyl)malonate (**51**)*: To a solution containing sodium hydride (0.79 g, 60% wt, 20 mmol) in *N*-methyl-2-pyrrolidone (5 mL) under an argon atmosphere at 0 °C was added diethyl malonate (1.9 g, 1.8 mL, 12 mmol). The reaction mixture was allowed to stir at the same temperature for 45 minutes, and 2-chloro-4-fluoro-3-methyl-1-nitrobenzene (1.5 g, 7.9 mmol) was added portion-wise. The reaction mixture was then slowly warmed to 50 °C and stirred overnight, then allowed to cool to rt. EtOAc (30 mL) and water (30 mL) were added to separate the organic layer. The organic layers were combined, dried over anhydrous Na_2SO_4 and concentrated under reduced pressure. The residue was purified by column chromatography (0–20% EtOAc in hexanes) to afford diethyl 2-(3-chloro-2-methyl-4-nitrophenyl)malonate (1.22 g, 47%) as a light yellow oil. LCMS: R_T = 1.094 min, >95% @ 215 and 254 nm, *m/z* = 330.2 $[M + H]^+$.

*2-(3-Chloro-2-methyl-4-nitrophenyl)acetic acid (**52**)*: To a solution containing diethyl 2-(3-chloro-2-methyl-4-nitrophenyl)malonate (1.22 g, 3.70 mmol, 1.0 equiv) in methanol (MeOH, 15 mL) was added 5 M aqueous NaOH (4.3 mL). The reaction mixture was heated at reflux for 12 hours, then allowed to cool to room temperature, and the organic solvent was evaporated under reduced pressure. The residue was acidified with 2 N aqueous HCl, resulting in the precipitation of a solid. The solid was filtered, washed with water, and vacuum dried to afford 2-(3-chloro-2-methyl-4-nitrophenyl)acetic acid (658 mg, 69.0%) as a crude product, which was used directly in the next step without further purification. ^1H NMR (400 MHz, $CDCl_3$) δ 7.57 (d, *J* = 8.3 Hz, 1H), 7.25 (d, *J* = 8.1 Hz, 1H), 3.79 (s, 2H), 2.45 (s, 3H).

2-(3-Chloro-2-methyl-4-nitrophenyl)-N-phenylacetamide (**53**): To a solution containing 2-(3-chloro-2-methyl-4-nitrophenyl)acetic acid (300 mg, 1.31 mmol) in DCM (5 mL) was added aniline (134 mg, 131 µL, 1.44 mmol) and triethylamine (661 mg, 0.92 mL, 6.53 mmol). The reaction mixture was allowed to stir at room temperature for 15 minutes, followed by the addition of 2,4,6-tripropyl-1,3,5,2,4,6-trioxatriphosphine 2,4,6-trioxide (1.16 g, 1.09 mL, 50% wt, 1.83 mmol). The reaction was allowed to stir for an additional 2 hours at room temperature, then subjected to a base/acid workup using saturated aqueous $NaHCO_3$ solution and 2N HCl. The organic compound was extracted with DCM, and the combined organic layers were dried over anhydrous Na_2SO_4. The solvent was removed under reduced pressure to afford the crude product, 2-(3-chloro-2-methyl-4-nitrophenyl)-N-phenylacetamide, as a colorless solid. The product was used directly in the next step without further purification. ^{1}H NMR (400 MHz, DMSO-d_6) δ 10.31 (s, 1H), 7.83 (d, J = 8.3 Hz, 1H), 7.62 – 7.55 (m, 2H), 7.50 (d, J = 8.3 Hz, 1H), 7.36 – 7.23 (m, 2H), 7.10 – 7.00 (m, 1H), 3.94 (s, 2H), 2.42 (s, 3H).

2-(4-Amino-3-chloro-2-methylphenyl)-N-phenylacetamide (**54**): To a solution containing 2-(3-chloro-2-methyl-4-nitrophenyl)-N-phenylacetamide (400 mg, 1.31 mmol) in EtOH (9 mL) and water (0.9 mL) was added tin(II) chloride dihydrate (1.18 g, 5.25 mmol). The reaction mixture was heated to 65 °C and stirred for 2 hours. After completion, the mixture was diluted with DCM (15 mL) and 3 M aqueous NaOH (10 mL). The resulting mixture was passed through a hydrophobic frit/phase separator to isolate the organic layer. The organic phase was collected, dried over anhydrous Na_2SO_4, and concentrated under reduced pressure to yield 2-(4-amino-3-chloro-2-methylphenyl)-N-phenylacetamide (313 mg, 1.14 mmol, 86.8%) as a light yellow solid which was used in the next step without further purification. LCMS: R_T = 0.815 min, >95% @ 215 and 254 nm, m/z = 275.4 $[M + H]^+$.

Scheme S6: Synthesis of the aniline **58** required for **9j**.[a]

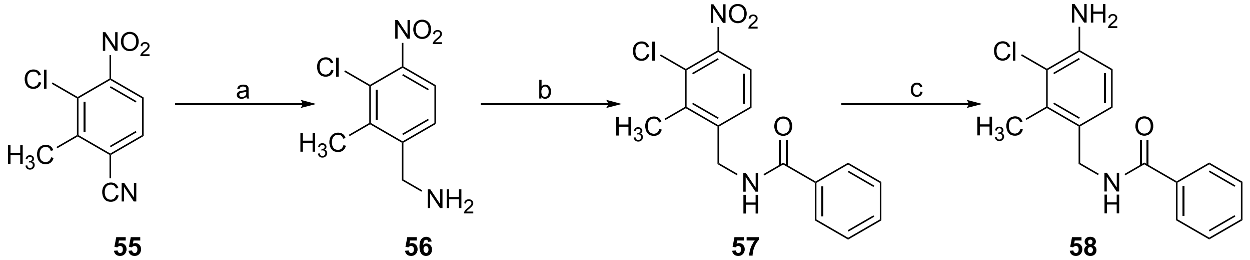

55 **56** **57** **58**

[a]Reagents and conditions: (a) BH_3•THF, THF, rt-75 °C, 2 h, 83%; (b) Benzoic acid, TEA, T3P (50% weight solution in DCE), rt, 1 h, 45%; (c) $SnCl_2$, EtOH: H_2O (10:1), 65 °C, 12h, 71%.

(3-Chloro-2-methyl-4-nitrophenyl)methamine (**56**): To a solution containing 3-chloro-2-methyl-4-nitrobenzonitrile **55** (150 mg, 763 µmol) in THF (10 mL) was added borane-tetrahydrofuran complex (1 M in THF, 3.05 mL, 3.05 mmol) at 25 °C. After the addition was complete, the reaction mixture was allowed to stir at 75 °C for 2 hours. The mixture was then allowed to cool to room temperature and quenched by the addition of MeOH (5 mL). The reaction was diluted with H_2O (15 mL) and extracted with EtOAc (2 × 15 mL). The combined organic layers were dried over anhydrous $_2SO_4$ and concentrated under reduced pressure to yield the crude product, (3-chloro-2-methyl-4-nitrophenyl)methamine,

as a yellow solid (150 mg, 83%). The product was used directly in the next step without further purification. LCMS: R_T = 0.574 min, >80% @ 215 and 254 nm, m/z = 201.2 [M + H]$^+$.

N-(3-Chloro-2-methyl-4-nitrobenzyl)benzamide (***57***): To a solution containing (3-chloro-2-methyl-4-nitrophenyl)methamine (150 mg, 748 μmol) in DCM (3 mL) was added benzoic acid (91.3 mg, 748 μmol) and triethylamine (378 mg, 0.53 mL, 3.74 mmol). The reaction mixture was allowed to stir at room temperature for 15 minutes. Subsequently, 2,4,6-tripropyl-1,3,5,2,4,6-trioxatriphosphine 2,4,6-trioxide (714 mg, 668 μL, 50% wt, 1.12 mmol) was added, and the reaction was allowed to stir for an additional 1 hour at room temperature. After completion, the reaction mixture was subjected to a base/acid workup using saturated aqueous $NaHCO_3$ solution and 2 N HCl. The organic compound was extracted with DCM, and the combined organic layers were dried over anhydrous Na_2SO_4. The solvents were removed under reduced pressure to afford the crude product, N-(3-chloro-2-methyl-4-nitrobenzyl)benzamide, as a colorless solid (158 mg, 45%, 65% purity). The product was used directly in the next step without further purification. LCMS: R_T = 1.043 min, >65% @ 215 and 254 nm, *m/z* = 305.2 [M + H]$^+$.

N-(4-Amino-3-chloro-2-methylbenzyl)benzamide ***(58)***: To a solution containing *N*-(3-chloro-2-methyl-4-nitrobenzyl)benzamide (150 mg, 492 μmol, 1.0 equiv) in EtOH (6 mL) and H_2O (0.6 mL) was added tin(II) chloride dihydrate (444 mg, 1.97 mmol, 4.0 equiv). The reaction mixture was heated to 65 °C and stirred overnight, then diluted with DCM (15 mL) and 3M aqueous OH (10 mL). The resulting mixture was passed through a hydrophobic frit/phase separator to isolate the organic layer. The organic phase was collected, dried over anhydrous Na_2SO_4, and concentrated under reduced pressure to yield *N*-(4-amino-3-chloro-2-methylbenzyl)benzamide (135 mg, 99.8%) as a light yellow solid which was taken to next step without further purification.

Scheme S7: Synthesis of the aniline **61** required for **9k**.[a]

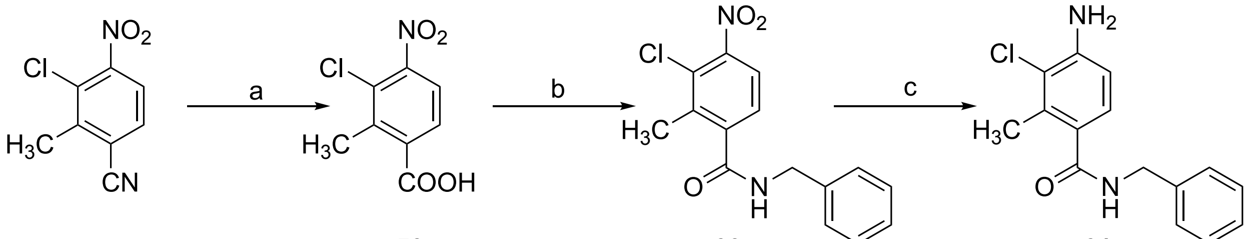

55 **59** **60** **61**

[a]Reagents and conditions: (a) AcOH:H_2O:H_2SO_4 (1:1:1), 120 °C, 5 h, 90%; (b) Benzylamine, TEA, T3P (50% weight solution in DCE), rt, 1h, 65%; (c) $SnCl_2$, EtOH: H_2O (10:1), 65 °C, 12 h, 71%.

3-Chloro-2-methyl-4-nitrobenzoic acid (***59***): A mixture containing 3-chloro-2-methyl-4-nitrobenzonitrile **55** (400 mg, 2.03 mmol, 1.0 equiv), acetic acid (4 mL), deionized H_2O (4 mL), and concentrated sulfuric acid (4 mL) was allowed to stir at 120 °C for 5 hours. The reaction mixture was diluted with deionized water (10 mL) and filtered. The solid residue was collected and dried under vacuum to afford 3-chloro-2-methyl-4-nitrobenzoic acid as an off-

white solid (395 mg, 1.83 mmol, 90% yield). ^{1}H NMR (400 MHz, DMSO-d_6) δ 7.91 (d, J = 8.4 Hz, 1H), 7.85 (d, J = 8.4 Hz, 1H), 2.58 (s, 3H).

*N-Benzyl-3-chloro-2-methyl-4-nitrobenzamide (**60**)*: To a solution containing 3-chloro-2-methyl-4-nitrobenzoic acid (75 mg, 0.35 mmol, 1.0 equiv) in DCM (2 mL) was added phenylmethamine (37 mg, 38 µL, 0.35 mmol, 1.0 equiv) and triethylamine (0.18 g, 0.24 mL, 1.7 mmol, 5.0 equiv). The reaction mixture was allowed to stir at room temperature for 15 minutes, followed by the addition of 2,4,6-tripropyl-1,3,5,2,4,6-trioxatriphosphine 2,4,6-trioxide (T3P, 50% wt in DCE, 0.33 g, 0.31 mL, 0.52 mmol, 1.5 equiv). The reaction was allowed to stir for an additional 1 hour at room temperature and then subjected to a base/acid workup using saturated aqueous sodium bicarbonate and 2N hydrochloric acid. The organic compound was extracted with DCM, and the combined organic layers were dried over anhydrous sodium sulfate and concentrated under reduced pressure. The crude product, N-benzyl-3-chloro-2-methyl-4-nitrobenzamide, was obtained as a colorless solid (92 mg, 0.30 mmol, 65% yield) and used directly in the next step without further purification. LCMS: R_T = 1.050 min, >75% @ 215 and 254 nm, m/z = 305.2 [M + H]$^+$.

*4-Amino-N-benzyl-3-chloro-2-methylbenzamide (**61**)*: To a solution containing N-benzyl-3-chloro-2-methyl-4-nitrobenzamide (110 mg, 361 µmol, 1.0 equiv) in EtOH (6 mL) and H_2O (0.6 mL) was added tin(II) chloride dihydrate (326 mg, 1.44 mmol). The reaction mixture was heated at 65 °C and stirred overnight. After completion, the mixture was diluted with DCM (15 mL) and 3M aqueous NaOH (10 mL). The resulting mixture was passed through a hydrophobic frit/phase separator to isolate the organic layer. The organic phase was collected, dried over anhydrous Na_2SO_4 and concentrated under reduced pressure to yield 4-amino-N-benzyl-3-chloro-2-methylbenzamide as a light yellow solid (88 mg, 71% yield). LCMS: R_T = 0.901 min, >80% @ 215 and 254 nm, m/z = 257.2 [M + H]$^+$.

Scheme S8: Synthesis of the aniline **67**required for **9l.**[a]

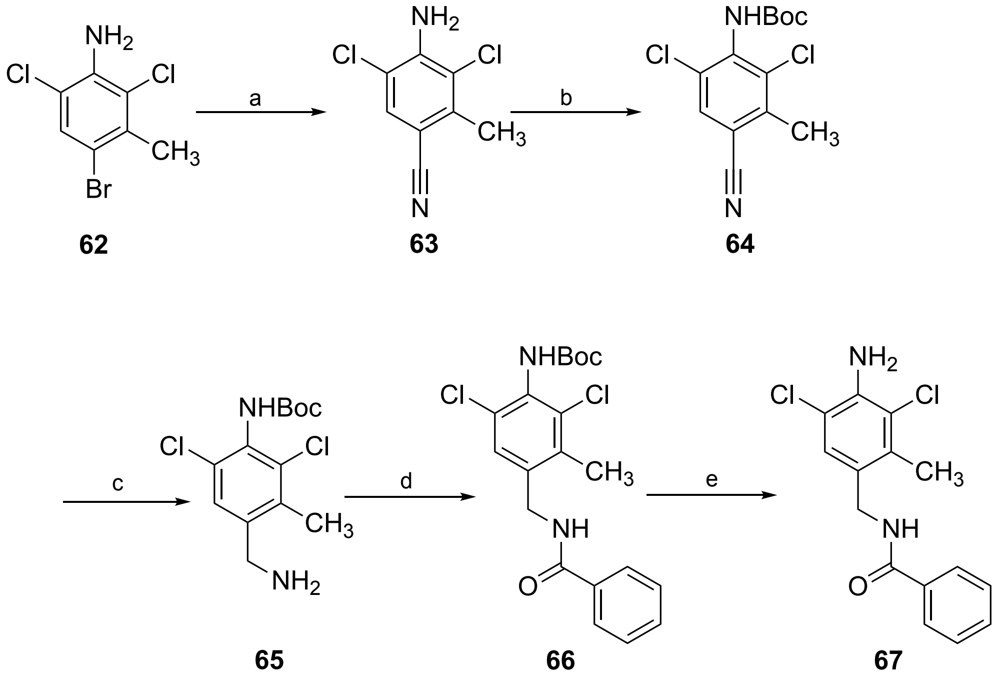

62 **63** **64**

65 **66** **67**

[a]Reagents and conditions: (a) CuCN, DMF, 145 °C, o/n, 80%; (b) Boc_2O, DMAP, THF, 84%; (c) $CoCl_2$, THF/H_2O, 0 °C, then $NaBH_4$, 74%; (d) Benzoic acid, HATU, DMF, Et_2N, rt,1 h, 54%; (e) 4.0 M HCl, DCM, 3 h.

*4-Amino-3,5-dichloro-2-methylbenzonitrile (**63**)*: To a solution containing of 4-bromo-2,6-dichloro-3-methylaniline **62** (1g, 3.92 mmol) in DMF was added copper cyanide (702 mg, 2eq.). The stirred mixture was heated at 145 °C overnight. The reaction mixture was allowed to cool to room temperature and then poured into ice water. EtOAc was added and the insoluble solid was collected by filtration and rinsed with EtOAc. The organic layer was separated, and the aqueous layer was extracted with EtOAc. The combined organic layers were washed with aq. $NaHCO_3$ and brine, dried over na_2SO_4, filtered, and concentrated under reduced pressure. The residue was purified using silica gel chromatography (0-10% MeOH/DCM) to afford 4-amino-3,5-dichloro-2-methylbenzonitrile (630 mg, 80%); LCMS: R_T = 1.035 min, >98% @ 215 and 254 nm, *m/z* = 202.3 $[M + H]^+$. ^1H NMR (400 MHz, DMSO-d_6) δ 7.74 (s, 1H), 6.52 (s, 2H), 2.43 (s, 3H).

*tert-Butyl (2,6-dichloro-4-cyano-3-methylphenyl)carbamate (**64**)*: A solution containing 4-amino-3,5-dichloro-2-methylbenzonitrile (500 mg, 249 mmol), di-*tert*-butyl dicarbonate (1.09g, 4.97 mmol) and 4-(dimethylamino)pyridine (2 eq.) in tetrahydrofuran (5 mL) was heated at reflux for 12 h. The reaction mixture was concentrated to half the volume, diluted with diethyl ether (0.15 L) and washed with a 2.0 M aqueous solution (0.10 L) of citric acid, then dried over sodium sulfate. The solvents were evaporated under reduced pressure and the residue was purified using silica gel chromatography (0-30% ethyl acetate/hexane) to give desired product tert-butyl (2,6-dichloro-4-cyano-3-methylphenyl)carbamate (630 mg, 84%). LCMS: R_T = 1.437 min, >95% @ 215 and 254 nm, *m/z* = 246.4 $[M - 56]^+$.

*tert-Butyl (4-(aminomethyl)-2,6-dichloro-3-methylphenyl)carbamate (**65**)*: A solution of tert-butyl (2,6-dichloro-4-cyano-3-methylphenyl)carbamate (50 mg, 166 µmol) and cobaltous chloride (32 mg, 249 µmol) in 2:1 THF/H_2O (1 mL) was cooled to 0 °C and treated with sodium borohydride (31 mg, 830 µmol), portion-wise over several minutes. The reaction mixture was allowed to warm to room temperature and stirred for 1 h. After the addition of 1 mL of conc NH_4OH, the mixture was stirred for 5 min and then filtered, and the collected solids were washed with 2:1 THF/H_2O (2 mL). The filtrates were concentrated, and the residue was partitioned between dichloromethane (10 mL) and H_2O (5 mL). The aqueous phase was extracted with dichloromethane (2×5 mL), and the combined organic phases were washed with brine and concentrated to afford the desired product tert-butyl (4-(aminomethyl)-2,6-dichloro-3-methylphenyl)carbamate (37 mg, 74%); ^{1}H NMR (400 MHz, DMSO-d_6) δ 7.59 (s, 1H), 3.76 (s, 2H), 2.30 (s, 3H), 1.97 (br, 2H), 1.37 (s, 9H).

*tert-Butyl (4-(benzamidomethyl)-2,6-dichloro-3-methylphenyl)carbamate (**66**)*: To a stirred solution of tert-butyl (4-(aminomethyl)-2,6-dichloro-3-methylphenyl)carbamate (37 mg, 122 µmol) in DMF (3.0 mL) was added HATU (52 mg, 135 µmol), benzoic acid (15 mg, 123 µmol), and triethylamine (35 µl, 2 eq.). The reaction mixture was allowed to stir at rt for 1 h. H_2O was added, and the reaction was extracted with EtOAc (3x5 mL). The organic layers were combined, and the solvent was removed under reduced pressure. The residue was purified using silica gel chromatography (0-30% ethyl acetate/hexane) to give the desired product tert-butyl (4-(benzamidomethyl)-2,6-dichloro-3-methylphenyl)carbamate (27 mg, 54%). LCMS: R_T = 2.121 min, *m/z* = 354.0 $[M - 56]^+$.

N-(4-amino-3,5-dichloro-2-methylbenzyl)benzamide hydrochloride (**67**): To a solution containing tert-butyl (4-(benzamidomethyl)-2,6-dichloro-3-methylphenyl)carbamate (40 mg, 125 µmol) in DCM (1 mL) was added 4.0 M HCl (156 µL, 625 µmol). The reaction mixture was allowed to stir for 3 h, and the solvent was removed under reduced pressure to afford N-(4-amino-3,5-dichloro-2-methylbenzyl)benzamide hydrochloride, which was carried on to the next step without purification.

Scheme S9: Synthesis of bromide **24** required for **10 d-e** and **10 g-j**.[a]

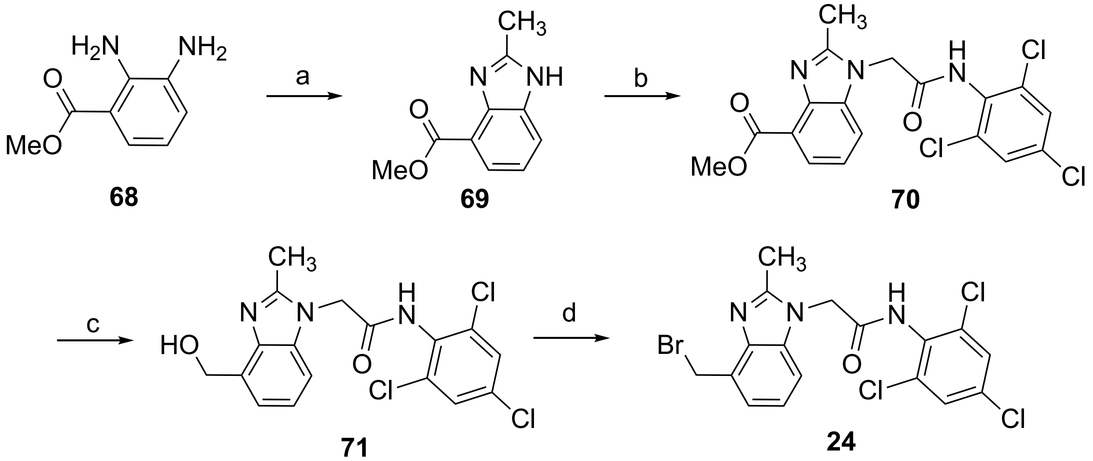

[a]Reagents and Conditions: (a) Trimethyl orthoformate, H_2SO_4, MeOH, 96%; (b) 2-bromo-N-(2,4,6-trichlorophenyl)acetamide, DMF, 70°C, 12 h, 70 %; (c) $LiAlH_4$, THF, 0°C, 50 %; (d) PBr_3, DCM, 0°C, 78%.

*Methyl 2-methyl-1H-benzo[d]imidazole-4-carboxylate (**69**):* Trimethyl orthoacetate (216 mg, 1.81 mmol) was added drop wise to methyl 2,3-diaminobenzoate **68** (100 mg, 602 µmol, 1.0 eq) in MeOH (1 mL). Two drops of H_2SO_4 were added, and the reaction mixture was allowed to stir for 2 h at rt. The solvent was removed under reduced pressure and residue was purified on silica gel column (0-100% EtOAc/hexane) and then repurified by RP-HPLC using a gradient (5–95%, H_2O/ACN, 0.1% TFA) to afford methyl 2-methyl-1H-benzo[d]imidazole-4-carboxylate (110 mg, 96% yield). LCMS: R_T = 1.016 min, >98% @ 215 and 254 nm, *m/z* = 191.1 $[M + H]^+$.

*Methyl 2-methyl-1-(2-oxo-2-((2,4,6-trichlorophenyl)amino)ethyl)-1H-benzo[d]imidazole-4-carboxylate (**70**):* To methyl 2-methyl-1H-benzo[d]imidazole-4-carboxylate (110 mg, 578 µmol) in DMF (1 mL) was added 2-bromo-N-(2,4,6-trichlorophenyl)acetamide (220 mg, 694 µmol). The reaction mixture was heated at 70 °C for 12 h. The crude product was purified on Gilson preparative HPLC to afford methyl 2-methyl-1-(2-oxo-2-((2,4,6-trichlorophenyl)amino)ethyl)-1H-benzo[d]imidazole-4-carboxylate (172.2 mg, 70% yield). LCMS: R_T = 1.474 min, >98% @ 215 and 254 nm, *m/z* = 427.9 $[M + H]^+$. δ ^1H NMR (400 MHz, DMSO-d_6) δ 10.71 (s, 1H), 8.22 (d, *J* = 8.3 Hz, 1H), 8.11 (d, *J* = 7.6 Hz, 1H), 7.80 (s, 2H), 5.60 (s, 2H), 4.00 (s, 3H), 2.91 (s, 3H).

*2-(4-(Hydroxymethyl)-2-methyl-1H-benzo[d]imidazol-1-yl)-N-(2,4,6-trichlorophenyl)acetamide (**71**)*: To methyl 2-methyl-1-(2-oxo-2-((2,4,6-trichlorophenyl)amino)ethyl)-1H-benzo[d]imidazole-4-carboxylate (230 mg, 1 Eq, 539 µmol) in THF (3 mL) at 0 °C under a nitrogen atmosphere was added LAH (24.5 mg, 323 µL, 2 molar, 1.2 Eq, 647 µmol) dropwise. The reaction mixture was allowed to warm to rt and stirred for an additional 3h. After completion of the reaction, the mixture was cooled to 0 °C and quenched by the addition of EtOAc (10 mL) followed by sat. aq. Na_2SO_4 (10 mL). The mixture was diluted with EtOAc and filtered through a pad of celite with additional 5% MeOH/DCM wash. The filtrate was dried (Na_2SO_4), concentrated under reduced pressure, and purified by silica gel chromatography (0-20% MeOH/DCM) to provide 2-(4-(hydroxymethyl)-2-methyl-1H-benzo[d]imidazol-1-yl)-N-(2,4,6-trichlorophenyl)acetamide (107.5 mg, 50% yield). LCMS: R_T = 1.374 min, >98% @ 215 and 254 nm, m/z = 399.9 $[M + H]^+$.

*2-(4-(Bromomethyl)-2-methyl-1H-benzo[d]imidazol-1-yl)-N-(2,4,6-trichlorophenyl)acetamide (**24**):* 2-(4-(Hydroxymethyl)-2-methyl-1H-benzo[d]imidazol-1-yl)-N-(2,4,6-trichlorophenyl)acetamide (230 mg, 577 µmol) was dissolved in CH_2Cl_2 (2 mL) and cooled to 0 °C. Phosphorus tribromide (65.3 µL, 635 µmol) was added to the solution at 0 °C. The reaction mixture was allowed to stir at room temperature for 1 h. After completion of the reaction, the reaction mixture was concentrated under reduced pressure and carried on as crude 2-(4-(bromomethyl)-2-methyl-1H-benzo[d]imidazol-1-yl)-N-(2,4,6-trichlorophenyl)acetamide (207.8 mg, 78% crude yield). LCMS: R_T = 1.526 min, >98% @ 215 and 254 nm, m/z = 461.8 $[M + H]^+$.

Scheme S10: Synthesis of the intermediate **25** required for **10f**. [a]

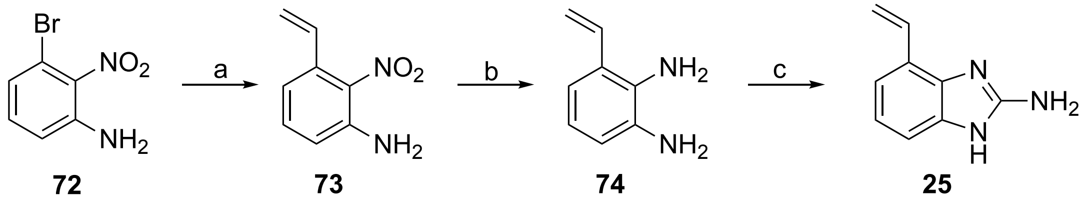

[a]Reagents and conditions: (a) Potassium vinyltrifluoroborate (1.17 g, 8.7 mmol), $PdCl_2$(dppf), Cs_2CO_3, THF, 80 °C, 16 h, 80%; (b) Fe, NH_4Cl, EtOH, H_2O, 80 °C, 1.5 h, quantitative; (c) Br-CN, MeOH:DCM, rt, 16h, 95%.

*2-Nitro-3-vinylaniline (**73**):* A mixture containing 3-bromo-2-nitroaniline **72** (1.0 g, 4.6 mmol), potassium vinyltrifluoroborate (1.17 g, 8.7 mmol), [1,1'-bis(diphenylphosphino)ferrocene] dichloropalladium(II) (336.3 mg, 0.46 mmol) and Cesium carbonate (3.0 g, 9.2 mmol) in THF (20.0 mL) at rt in a 100 mL dry flask was purged with argon and then heated at 80 °C with a condenser overnight. The reaction mixture was allowed to cool to rt, filtered through a celite pad, and washed with EtOAc (20 mL). The solvent was evaporated, and the crude was purified by silica gel chromatography to provide 2-nitro-3-vinylaniline (606.0 mg, 80%). ^1H NMR (400 MHz, $CDCl_3$) δ 7.23 (t, J = 7.8 Hz, 1H), 6.93 (dd, J = 10.8, 17.3 Hz, 1H), 6.84 (d, J = 7.6 Hz, 1H), 6.72 (dd, J = 1.1, 8.2 Hz, 1H), 6.63 (dd, J = 1.1, 17.1 Hz, 1H), 5.34 (dd, J = 1.1, 10.9 Hz, 1H).

*3-Vinylbenzene-1,2-diamine (**74**)*: To a solution containing 2-nitro-3-vinylaniline (100.0 mg, 0.6 mmol) in a mixture of EtOH (4.0 mL) and H_2O (0.4 mL) was added Iron powder (204.3 mg, 3.6 mmol) and ammonium chloride (96.2 mg, 1.8 mmol) at rt. The reaction mixture was heated at 80 °C overnight. The mixture was allowed to cool to rt, filtered through a celite pad, and washed with MeOH. The organic layer was concentrated to provide crude 3-vinylbenzene-1,2-diamine (120.0 mg) which was used for the next step without further purification. LCMS: R_t = 0.158 min, >90% @ 215 and 254 nm, m/z = 135.3 $[M + H]^+$.

*4-Vinyl-1H-benzo[d]imidazol-2-amine (**25**)*: To a solution containing crude 3-vinylbenzene-1,2-diamine (400.0 mg, 2.77 mmol) in a mixture of MeOH (6 mL) and CH_2Cl_2 (3 mL) was added cyanogen bromide (1.39 mL, 4.16 mmol) dropwise at rt. The reaction mixture was allowed to stir at rt for 16 h and concentrated under reduced pressure. The crude product was purified by silica-gel chromatography (10% MeOH/DCM) to yield 4-vinyl-1*H*-benzo[d]imidazol-2-amine (467.0 mg, 95%). LCMS: R_t = 0.488 min, >90% @ 215 and 254 nm, m/z = 160. $[M + H]^+$.

Scheme S11: Synthesis of the *trans* cyclopropane-containing ester **27** required for **10m-o**.[a]

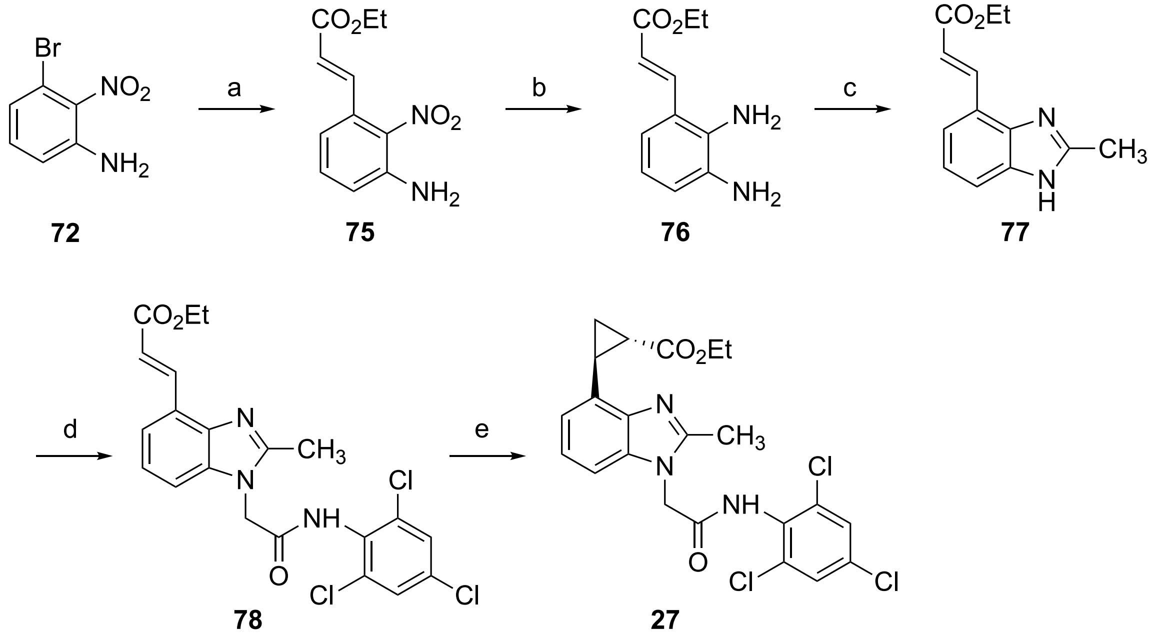

[a]Reagents and conditions: (a) ethyl acrylate, $Pd(OAc)_2$, Ph_3P, TEA, DMF, 140 °C, 5 h, 66%; (b) Fe, NH_4Cl, EtOH, H_2O, 80 °C, 16 h, 90%; (c) Triethyl orthoacetate, H_2SO_4 (cat), EtOH, rt, 3 h, 76%; (d) 2-bromo-*N*-(2,4,6-trichlorophenyl)acetamide, EtOH, 90 °C, 18 h, 50%; (e) trimethylsulfonium iodide, NaH, DMSO, 55 °C, 1.5 h, 56%.

*Ethyl (E)-3-(3-amino-2-nitrophenyl)acrylate (**75**)*: To a solution containing 3-bromo-2-nitroaniline **72** (1.0 g, 4.6 mmol) in DMF (10.0 mL) in a 20 mL microwave vial was added ethyl acrylate (0.98 mL, 9.21 mmol), $Pd(OAc)_2$, (30.9 mg, 0.13 mmol), Ph_3P (72.2 mg, 0.27 mmol), and NEt_3 (1.68 mL, 11.9 mmol). The mixture was heated at 140 °C with a condenser for 5h. The reaction mixture was allowed to cool down to rt and quenched by the addition of saturated aqueous $NaHCO_3$. The aqueous layer was extracted with EtOAc (3 x 20 mL). The combined organic layers were dried with anhydrous Na_2SO_4 and the crude material was purified by silica gel chromatography to provide ethyl (*E*)-3-(3-amino-2-nitrophenyl)acrylate, 82 (725.0 mg, 66%). LCMS R_t = 0.748 min, >90% @ 215 and 254 nm, *m/z* = 254.1 [M +18]$^+$. ^1H NMR (400 MHz, $CDCl_3$) δ 7.91 (d, *J* = 15.7 Hz, 1H), 7.29 (d, *J* = 7.9 Hz, 1H), 6.84-6.81 (m, 2H), 6.24 (d, *J* = 15.6 Hz, 1H), 4.26 (q, *J* = 7.0 Hz, 2H), 1.33 (t, *J* = 7.1 Hz, 3H).

*Ethyl (E)-3-(2,3-diaminophenyl)acrylate (**76**)*: To a solution containing ethyl (*E*)-3-(3-amino-2-nitrophenyl)acrylate (725.0 mg, 3.06 mmol) in EtOH (16.0 mL)/H_2O(1.6 mL) at rt in a 20 mL vial, was added iron powder (857.3 mg, 15.3 mmol) and ammonium chloride (327.3 mg, 6.1 mmol). The reaction mixture was heated at 80 °C overnight. The mixture was allowed to cool to rt, then filtered through a celite pad and washed with MeOH. The organic layer was concentrated to provide crude material which was purified by silica gel chromatography to yield ethyl (*E*)-3-(2,3-diaminophenyl)acrylate (575.0 mg, 90%). LCMS: R_t = 0.520 min, >90% @ 215 and 254 nm, *m/z* = 207.1 [M + H]$^+$.

*Ethyl-(E)-3-(2-methyl-1H-benzo[d]imidazol-4-yl)acrylate (**77**)*: To a solution containing ethyl (E)-3-(2,3-diaminophenyl)acrylate (700.0 mg, 3.39 mmol) in EtOH (8 mL) was added Triethyl orthoacetate (1.87 mL, 10.1 mmol) and concentrated H_2SO_4 (3 drops). The solution turned into a slurry and the reaction mixture was allowed to stir at rt for 3 h (mixture turned into a clear solution). The reaction mixture was concentrated under reduced pressure and the crude was dissolved in 10% MeOH/DCM (25 mL) and added to a saturated aqueous $NaHCO_3$ solution. The aqueous layer was extracted with 10% MeOH/DCM. The combined organic layers were dried over anhydrous Na_2SO_4, filtered, and concentrated to give crude ethyl (*E*)-3-(2-methyl-1*H*-benzo[*d*]imidazol-4-yl)acrylate (593.0 mg, 76%). The crude material was used for the next step without further purification. LCMS: R_t = 0.557 min, >90% @ 215 and 254 nm, *m/z* = 231.1 $[M + H]^+$.

*Ethyl-(E)-3-(2-methyl-1-(2-oxo-2-((2,4,6-trichlorophenyl)amino)ethyl)-1H-benzo[d]imidazol-4-yl)acrylate (**79**)*: To a solution containing (*E*)-3-(2-methyl-1*H*-benzo[*d*]imidazol-4-yl)acrylate (165.0 mg, 0.72 mmol) in EtOH (3.0 mL) was added 2-bromo-*N*-(2,4,6-trichlorophenyl)acetamide (302.7 mg, 0.31 mmol) and DIPEA (0.4 mL, 2.34 mmol). The reaction mixture was heated at 90 °C for 16 h and concentrated under reduced pressure. The crude product was purified by silica-gel chromatography (35% DCM/EtOAc) to give ethyl (*E*)-3-(2-methyl-1-(2-oxo-2-((2,4,6-trichlorophenyl)amino)ethyl)-1*H*-benzo[d]imidazol-4-yl)acrylate (167.0 mg, 50%). LCMS: R_t = 0.768 min, >90% @ 215 and 254 nm, *m/z* = 468.1 $[M + H]^+$.

*Ethyl-(1S,2S)-2-(2-methyl-1-(2-oxo-2-((2,4,6-trichlorophenyl)amino)ethyl)-1H-benzo[d]imidazol-4-yl)cyclopropane-1-carboxylate (**27**)*: To a solution containing Trimethylsulfonium iodide (102.2 mg, 1.08 mmol) in DMSO (2.5 mL) was added NaH (52.0 mg, 1.30 mmol, 60 wt%) at rt. The solution was allowed to stir at rt for 1 h, then added dropwise to a DMSO (2.0 mL) solution containing (*E*)-3-(2-methyl-1-(2-oxo-2-((2,4,6-trichlorophenyl)amino)ethyl)-1*H*-benzo[d]imidazol-4-yl)acrylate (338.0 mg, 0.72 mmol) at rt. The reaction mixture was heated at 55 °C for 1.5 h. The reaction was quenched by the addition of a H_2O /brine mixture and the aqueous layer was extracted with EtOAc. The combined organic layers were dried over anhydrous Na_2SO_4, filtered, and concentrated to provide the crude material, which was purified by silica-gel chromatography to provide ethyl (1*S*,2*S*)-2-(2-methyl-1-(2-oxo-2-((2,4,6-trichlorophenyl)amino)ethyl)-1*H*-benzo[d]imidazol-4-yl)cyclopropane-1-carboxylate (197.0 mg, 56%). LCMS: R_t = 0.768 min, >90% @ 215 and 254 nm, *m/z* = 482.1 $[M + H]^+$.

Scheme S12. Synthesis of the *cis* cyclopropane-containing ether **28** required for **10p-q.**[a]

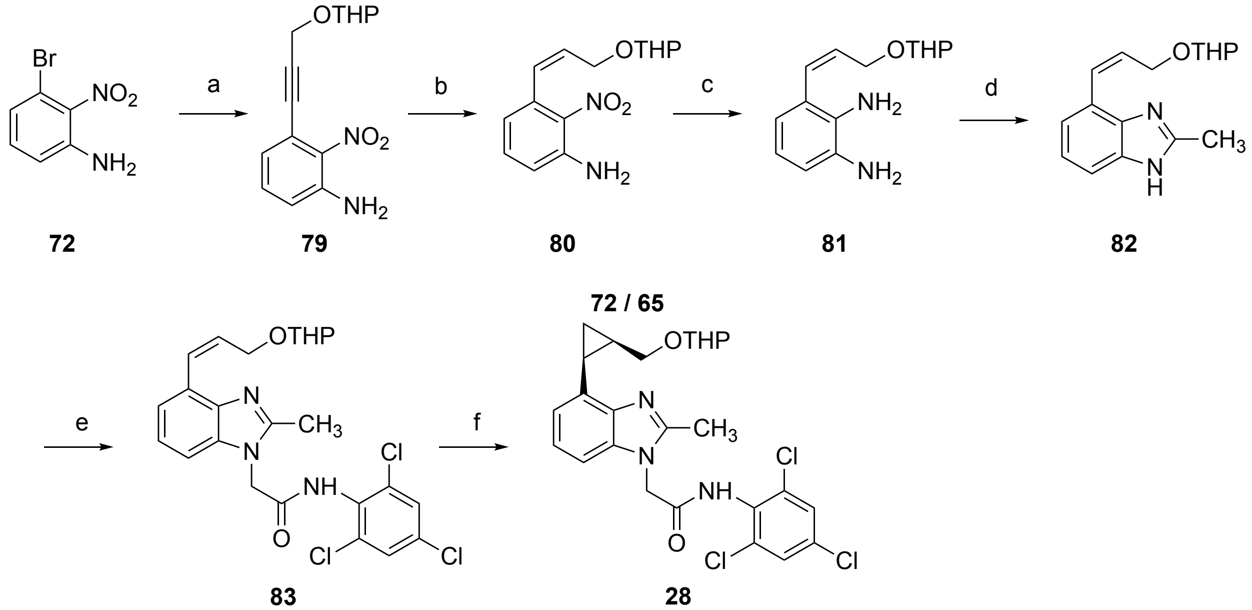

[a]Reagents and conditions: (a) 2-(prop-2-yn-1-yloxy)tetrahydro-2*H*-pyran, $PdCl_2(Ph_3P)_2$, CuI, TEA, 70 °C, 16 h, 53%; (b) Lindlar's catalyst, MeOH, H_2, 80 °C, 5 h; (c) Fe, NH_4Cl, EtOH, H_2O, 80 °C, 16h; (d) triethyl orthoacetate, H_2SO_4 (cat.), EtOH, rt, 3 h, quantitative; (e) 2-bromo-*N*-(2,4,6-trichlorophenyl)acetamide, EtOH, 90 °C, 18 h, 36%; (f) Et_2Zn, CH_2I_2, CH_2Cl_2, 0 °C-rt, 1 h, 42%.

*2-Nitro-3-(3-((tetrahydro-2H-pyran-2-yl)oxy)prop-1-yn-1-yl)aniline (**79**)*: To a solution containing 3-bromo-2-nitroaniline **72** (0.5 g, 2.3 mmol) in Et_3N (10.0 mL) in a 20 mL microwave vial was added $PdCl_2(Ph_3P)_2$ (32.2 mg, 0.04 mmol). The solution was allowed to stir for 10 min at rt, then 2-(prop-2-yn-1-yloxy)tetrahydro-2*H*-pyran (387.0 mg, 2.76 mmol) and CuI (17.5 mg, 0.09 mmol) were added. The reaction mixture was degassed with Argon for 15 min, then heated at 70 °C for 16 h. The reaction mixture was allowed to cool to rt and concentrated under reduced pressure. The crude material was purified by silica-gel chromatography to provide 2-nitro-3-(3-((tetrahydro-2*H*-pyran-2-yl)oxy)prop-1-yn-1-yl)aniline (338.0 mg, 53%). ^1H NMR (400 MHz, $CDCl_3$) δ 7.19 (dd, *J* = 7.6, 8.1 Hz, 1H), 6.90 (dd, *J* = 1.2, 7.5 Hz, 1H), 6.75 (dd, *J* = 1.2, 8.3 Hz, 1H), 5.47 (s, 2H), 4.94 (dd, *J* = 2.9, 3.7 Hz, 1H), 4.52 (s, 3H), 3.91-3.85 (m, 1H), 3.60-3.54 (m, 1H), 1.90-1.74 (m, 2H), 1.69-1.50 (m, 5H).

*(Z)-2-nitro-3-(3-((tetrahydro-2H-pyran-2-yl)oxy)prop-1-en-1-yl)aniline (**80**)*: To a solution containing 2-nitro-3-(3-((tetrahydro-2*H*-pyran-2-yl)oxy)prop-1-yn-1-yl)aniline (338.0 mg, 1.22 mmol) and MeOH (6.0 mL) was added Lindlar's catalyst (33.0 mg). The reaction mixture was allowed to stir under a H_2-balloon for 5 h. The reaction mixture was filtered through a celite pad and washed with MeOH. The organic layer was concentrated to provide crude (*Z*)-2-nitro-3-(3-((tetrahydro-2*H*-pyran-2-yl)oxy)prop-1-en-1-yl)aniline (305.0 mg) which was used without purification.

*(Z)-3-(3-((tetrahydro-2H-pyran-2-yl)oxy)prop-1-en-1-yl)benzene-1,2-diamine (**81**)*: To a solution containing (*Z*)-2-nitro-3-(3-((tetrahydro-2*H*-pyran-2-yl)oxy)prop-1-en-1-yl)aniline (670.0 mg, 2.41 mmol) in EtOH (12.0 mL)/H_2O(1.2 mL) in a 20 mL vial was added iron powder

(673.3 mg, 12.0 mmol) and ammonium chloride (389.6 mg, 7.2 mmol). The reaction mixture was heated at 80 °C overnight. The mixture was filtered through a celite pad and the solid was washed with MeOH/DCM. The organic layer was concentrated to provide crude (*Z*)-3-(3-((tetrahydro-2*H*-pyran-2-yl)oxy)prop-1-en-1-yl)benzene-1,2-diamine (580.0 mg) which was used for next step without purification. LCMS: R_t = 0.127 min, >90% @ 215 and 254 nm, *m/z* = 249.21 $[M + H]^+$.

*(Z)-2-Methyl-4-(3-((tetrahydro-2H-pyran-2-yl)oxy)prop-1-en-1-yl)-1H-benzo[d]imidazole (**82**)*: To a solution containing ethyl (*E*)-3-(2,3-diaminophenyl) acrylate (135.0 mg, 0.54 mmol) in MeOH (6 mL) at 0 °C was added trimethyl orthoacetate (0.2 mL, 1.63 mmol) and AcOH (3 drops). The solution turned into a slurry and the reaction mixture was allowed to stir at 0 °C for 1 h (mixture turned into a clear solution). The reaction mixture was concentrated under reduced pressure and the crude (*Z*)-2-methyl-4-(3-((tetrahydro-2*H*-pyran-2-yl)oxy)prop-1-en-1-yl)-1*H*-benzo[*d*]imidazole (177.0 mg) was used for the next step without purification. LCMS: R_t = 0.673 min, >90% @ 215 and 254 nm, m/z = 273.2 $[M + H]^+$.

*(Z)-2-(2-Methyl-4-(3-((tetrahydro-2H-pyran-2-yl)oxy)prop-1-en-1-yl)-1H-benzo[d]imidazol-1-yl)-N-(2,4,6-trichlorophenyl)acetamide (**83**)*: To a solution containing (*Z*)-2-methyl-4-(3-((tetrahydro-2*H*-pyran-2-yl)oxy)prop-1-en-1-yl)-1*H*-benzo[*d*]imidazole (177.0 mg, 0.65 mmol) in EtOH (5.0 mL) was added 2-bromo-*N*-(2,4,6-trichlorophenyl)acetamide (268.7 mg, 0.84 mmol) and DIPEA (0.34 mL, 1.95 mmol). The reaction mixture was heated at 90 °C for 16 h and concentrated under reduced pressure. The crude material was purified by ISCO silica-gel chromatography (50% DCM/EtOAc) to give (*Z*)-2-(2-methyl-4-(3-((tetrahydro-2*H*-pyran-2-yl)oxy)prop-1-en-1-yl)-1*H*-benzo[*d*]imidazol-1-yl)-*N*-(2,4,6-trichlorophenyl)acetamide (167.0 mg, 36%). LCMS: R_t = 0.922 min, >90% @ 215 and 254 nm, *m/z* = 510.2 $[M + H]^+$.

*2-(2-Methyl-4-((1S,2R)-2-(((tetrahydro-2H-pyran-2-yl)oxy)methyl)cyclopropyl)-1H-benzo[d]imidazol-1-yl)-N-(2,4,6-trichlorophenyl)acetamide (**28**)*: To a solution containing CH_2I_2 (0.13 mL, 1.66 mmol) and CH_2Cl_2 (6.0 mL) at 0 °C was added Et_2Zn (1.66 mL,1.66 mmol, 1 M solution in Hexane). A white precipitate formed, and the reaction mixture was allowed to stir for 0 °C for 40 min. Then, a solution containing (*Z*)-2-(2-methyl-4-(3-((tetrahydro-2*H*-pyran-2-yl)oxy)prop-1-en-1-yl)-1*H*-benzo[*d*]imidazol-1-yl)-*N*-(2,4,6-trichlorophenyl)acetamide (284.0 mg, 0.554 mmol) and CH_2Cl_2 (3.0 mL) was added dropwise. The reaction mixture was allowed to stir at 0 °C for 1 h and allowed to warm to rt and stirred for a further 1 h. The reaction mixture was quenched by the addition of saturated aqueous NH_4Cl. The aqueous layer was extracted with 10% MeOH/CH_2Cl_2. The combined organic layers were dried over anhydrous Na_2SO_4, filtered, and concentrated to provide the crude material which was purified by silica-gel chromatography to give 2-(2-methyl-4-((1*S*,2*R*)-2-(((tetrahydro-2*H*-pyran-2-yl)oxy)methyl)cyclopropyl)-1*H*-benzo[d]imidazol-1-yl)-*N*-(2,4,6-trichlorophenyl)acetamide (124.0 mg, 42%). LCMS: R_t = 0.929 min, >90% @ 215 and 254 nm, *m/z* = 524.2 $[M + H]^+$.

1HNMR data for final compounds

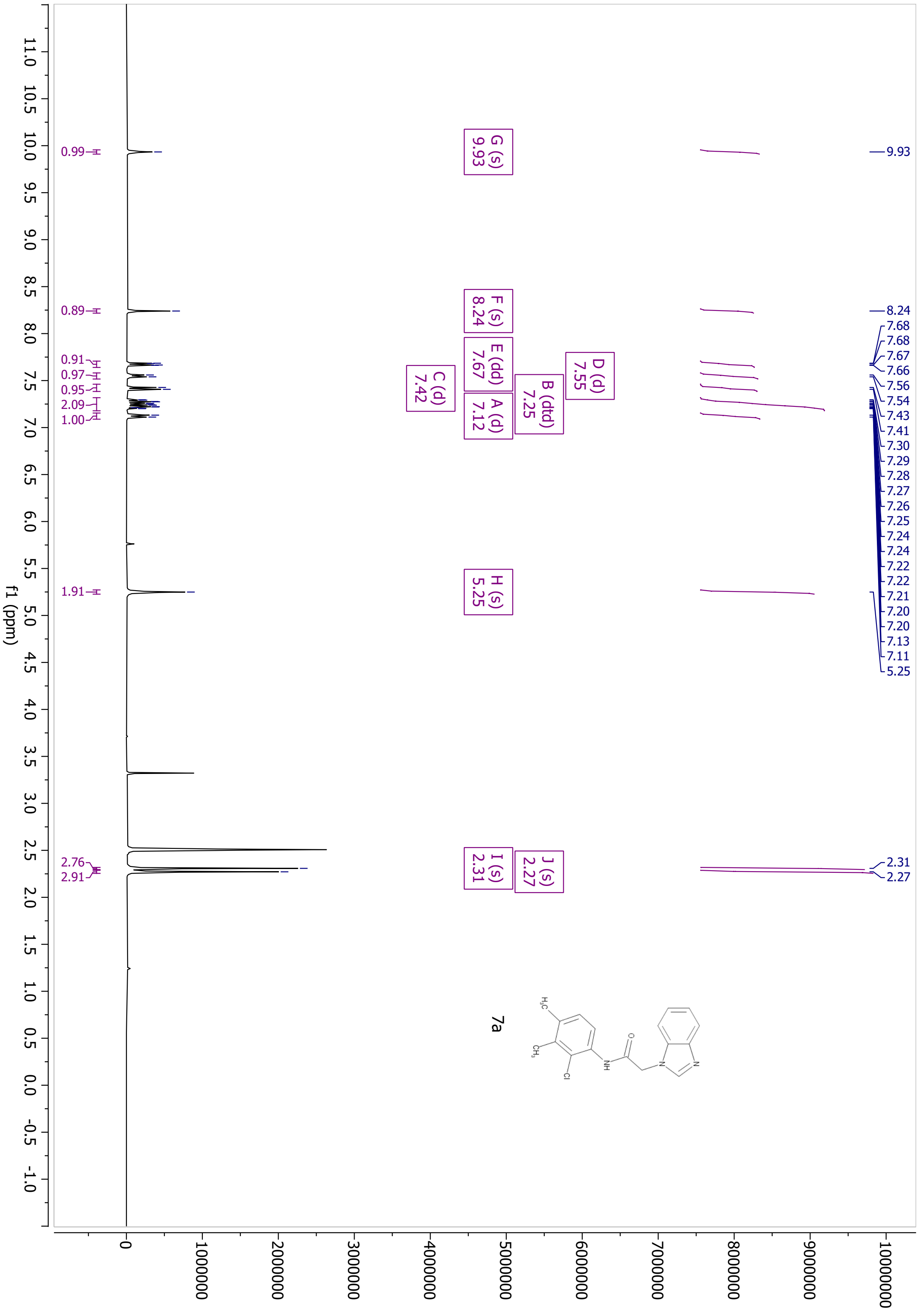

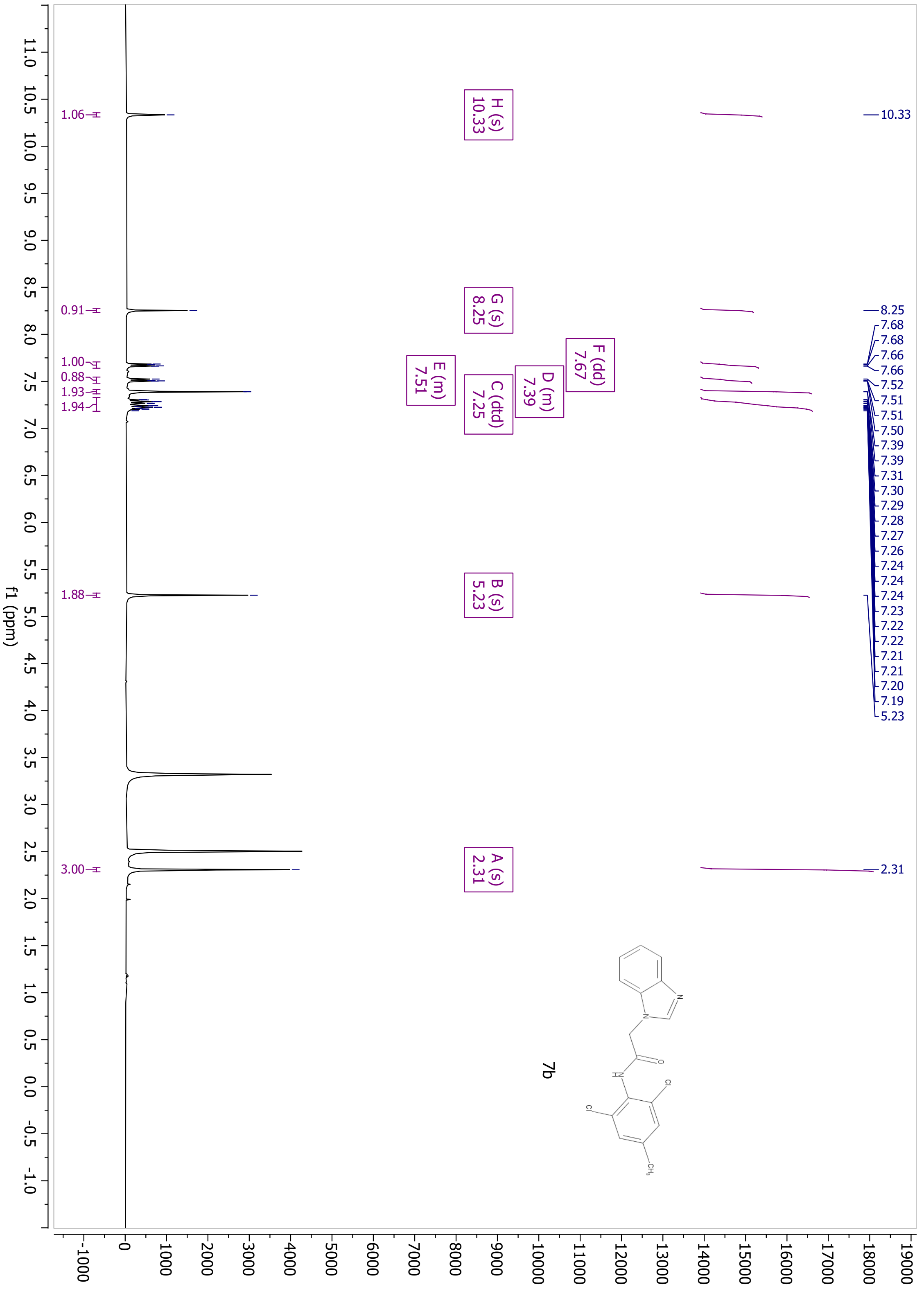

f1 (ppm)
H (s) 10.33
G (s) 8.25
F (dd) 7.67
D (m) 7.39
E (m) 7.51
C (dtd) 7.25
B (s) 5.23
A (s) 2.31
1.06
0.91
1.00
0.88
1.93
1.94
1.88
3.00
10.33
8.25
7.68
7.68
7.66
7.66
7.52
7.51
7.51
7.50
7.39
7.39
7.31
7.30
7.29
7.28
7.27
7.26
7.24
7.24
7.24
7.23
7.22
7.22
7.21
7.21
7.20
7.19
5.23
2.31
7b

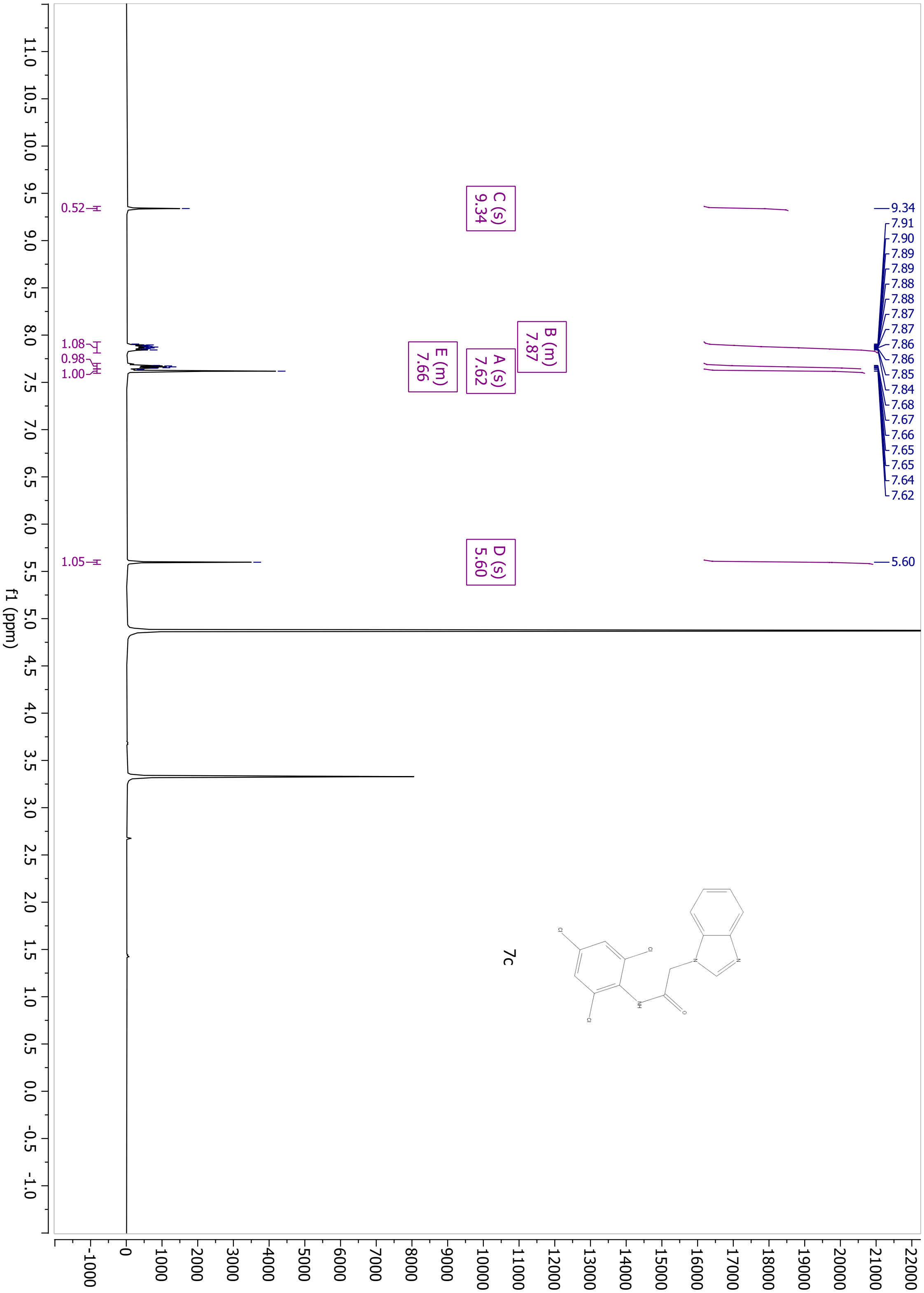
C (s)
9.34
B (m)
7.87
A (s)
7.62
E (m)
7.66
D (s)
5.60
0.52
1.08
0.98
1.00
1.05
9.34
7.91
7.90
7.89
7.89
7.88
7.88
7.87
7.87
7.86
7.86
7.85
7.84
7.68
7.67
7.66
7.65
7.65
7.64
7.62
5.60
f1 (ppm)
7c

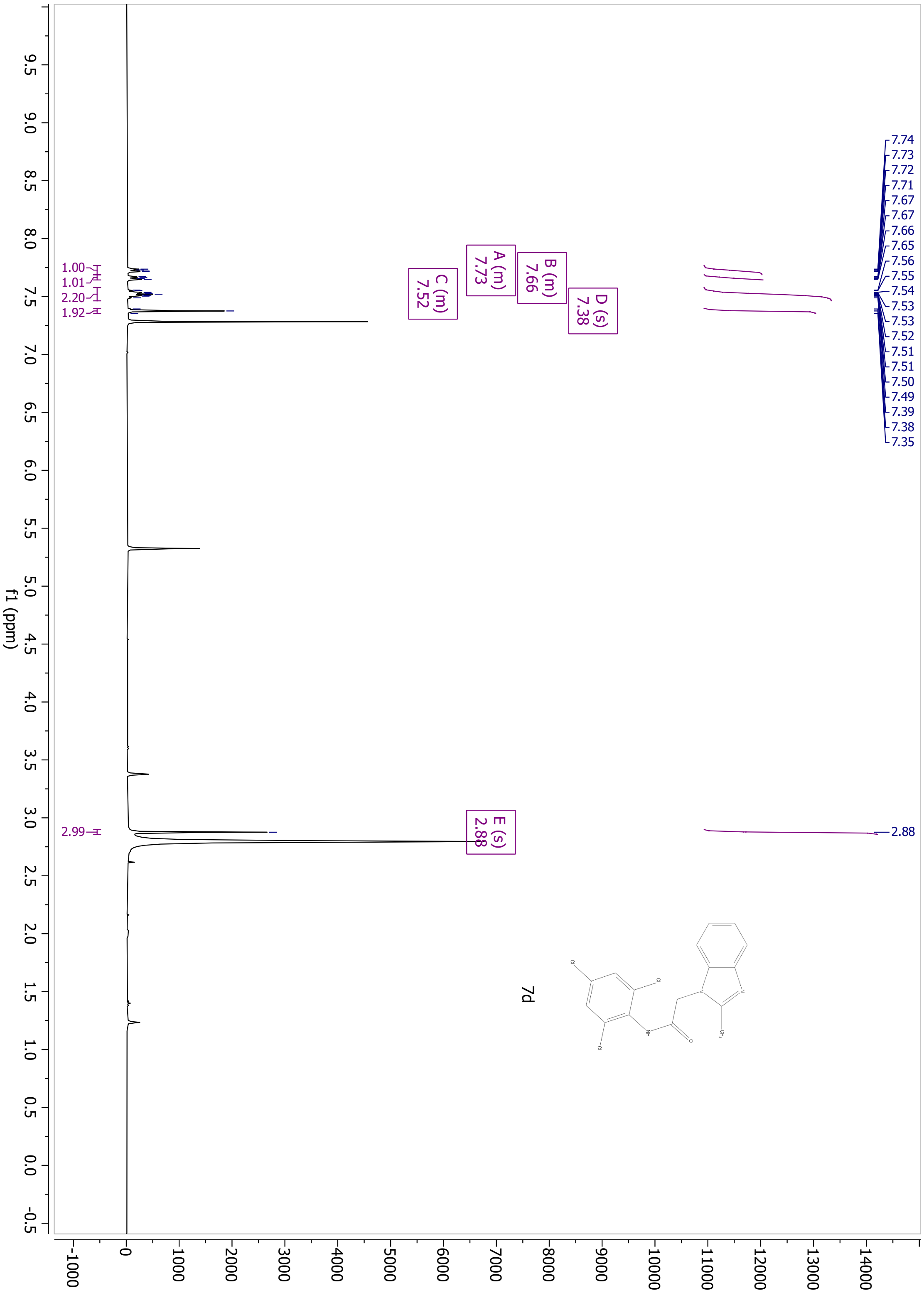

7d
f1 (ppm)
A (m) 7.73
B (m) 7.66
C (m) 7.52
D (s) 7.38
E (s) 2.88
1.00
1.01
2.20
1.92
2.99
7.74
7.73
7.72
7.71
7.67
7.67
7.66
7.65
7.56
7.55
7.54
7.53
7.53
7.52
7.51
7.51
7.50
7.49
7.39
7.38
7.35
2.88

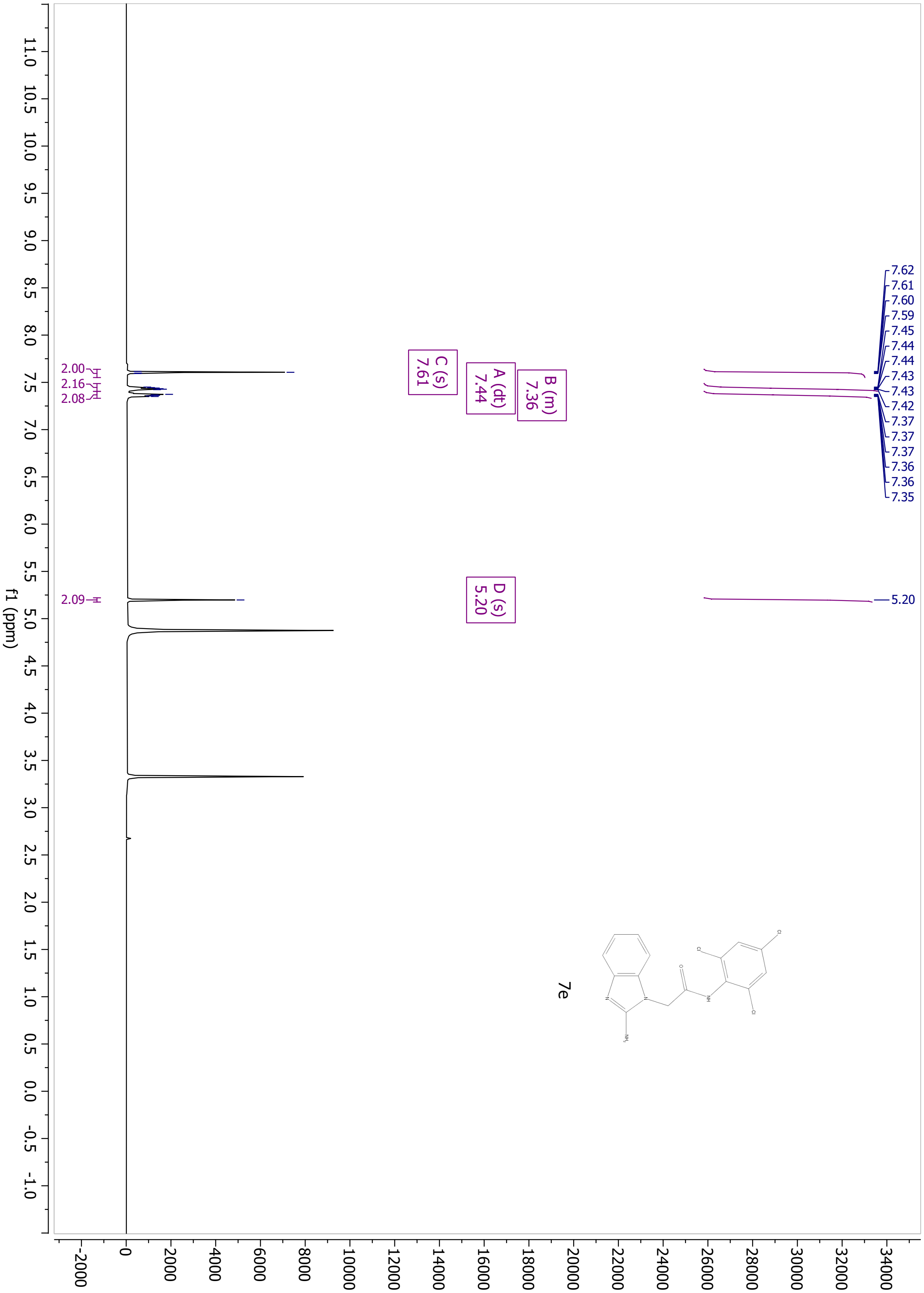
7e
C (s)
7.61
A (dt)
7.44
B (m)
7.36
D (s)
5.20
2.00
2.16
2.08
2.09
7.62
7.61
7.60
7.59
7.45
7.44
7.44
7.43
7.43
7.42
7.37
7.37
7.37
7.36
7.36
7.35
5.20
f1 (ppm)

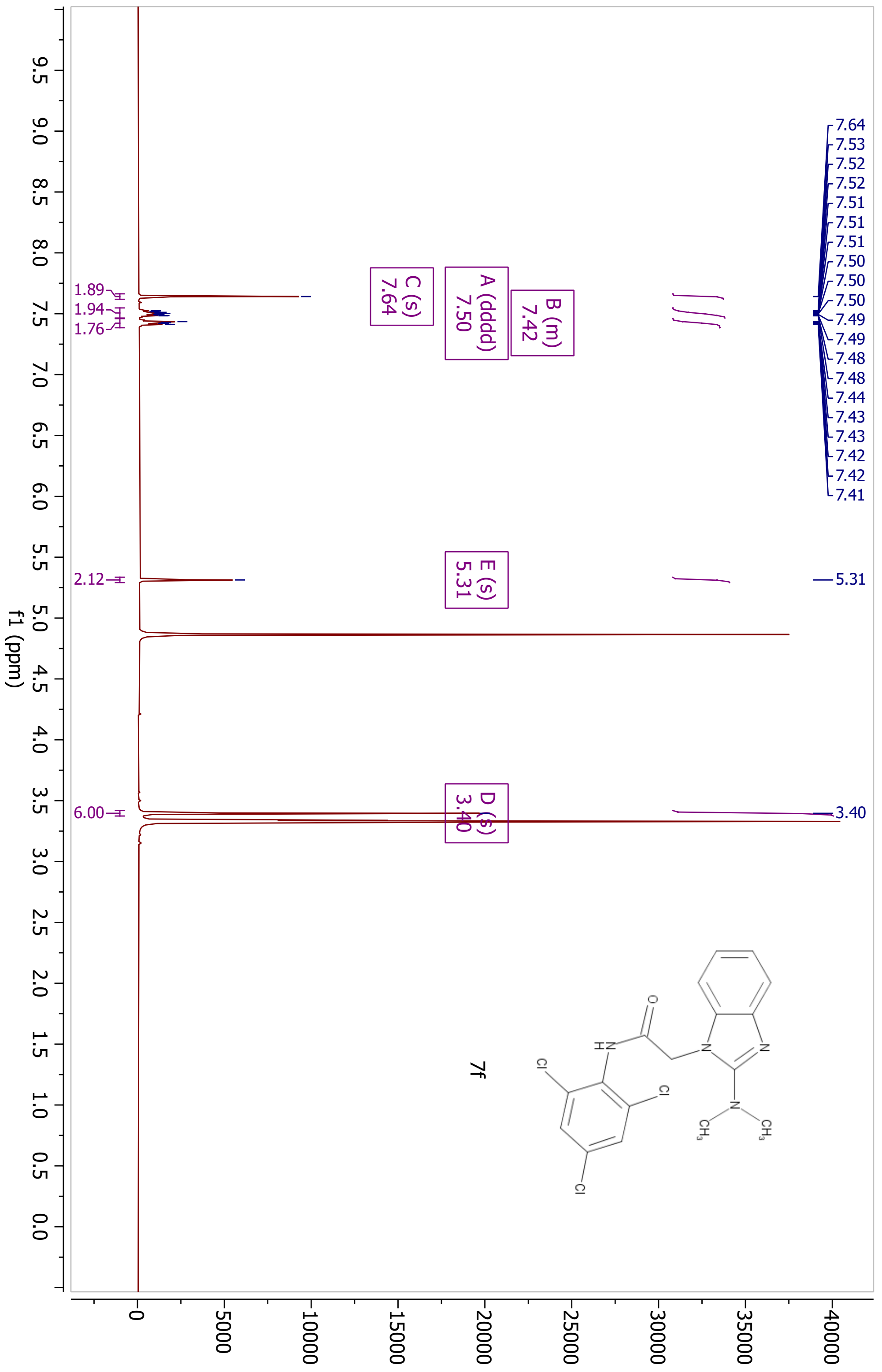

7f
C (s) 7.64
A (ddd) 7.50
B (m) 7.42
E (s) 5.31
D (s) 3.40
1.89
1.94
1.76
2.12
6.00
f1 (ppm)

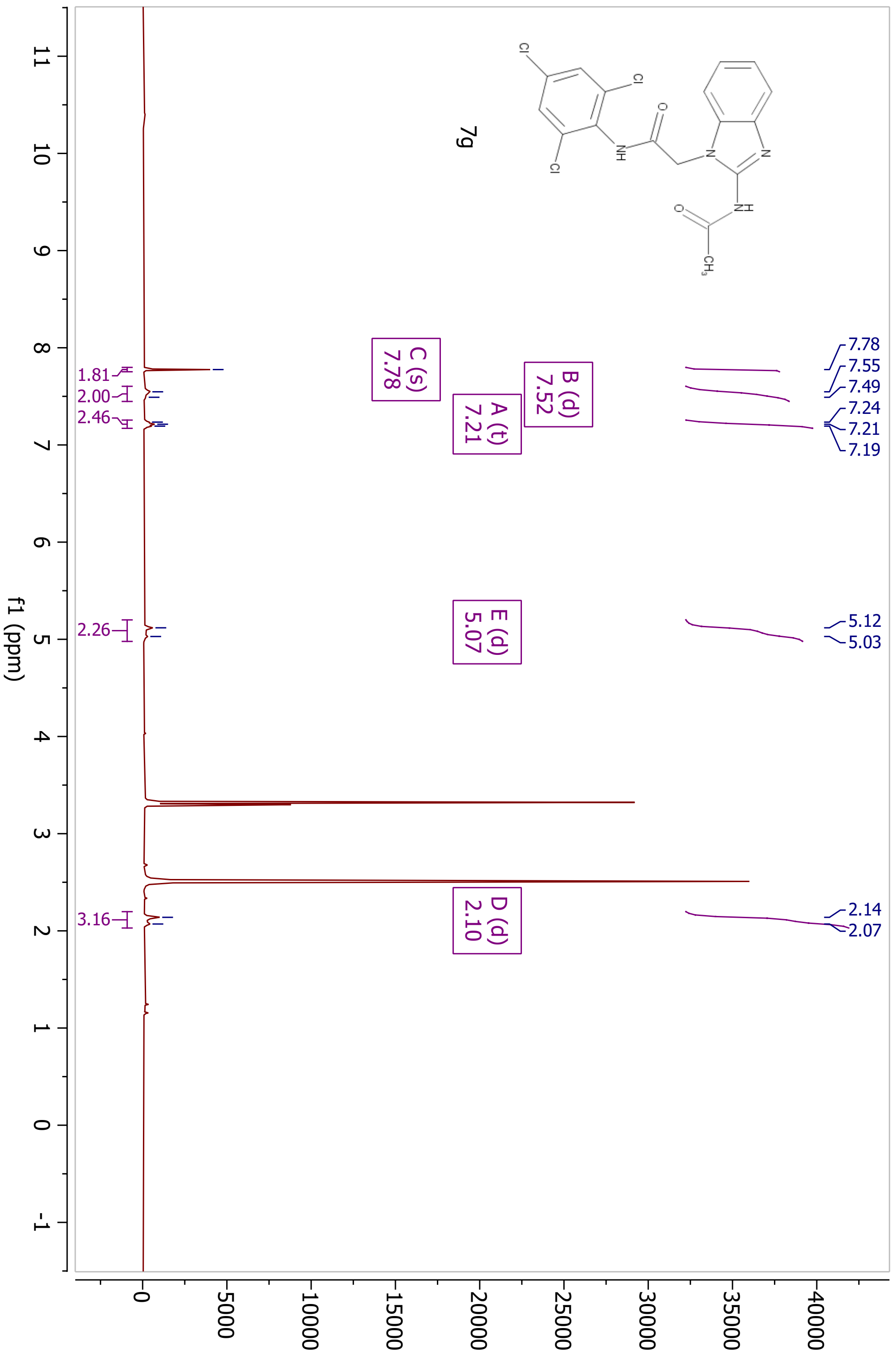
7g
C (s)
7.78
B (d)
7.52
A (t)
7.21
E (d)
5.07
D (d)
2.10
7.78
7.55
7.49
7.24
7.21
7.19
5.12
5.03
2.14
2.07
1.81
2.00
2.46
2.26
3.16
f1 (ppm)

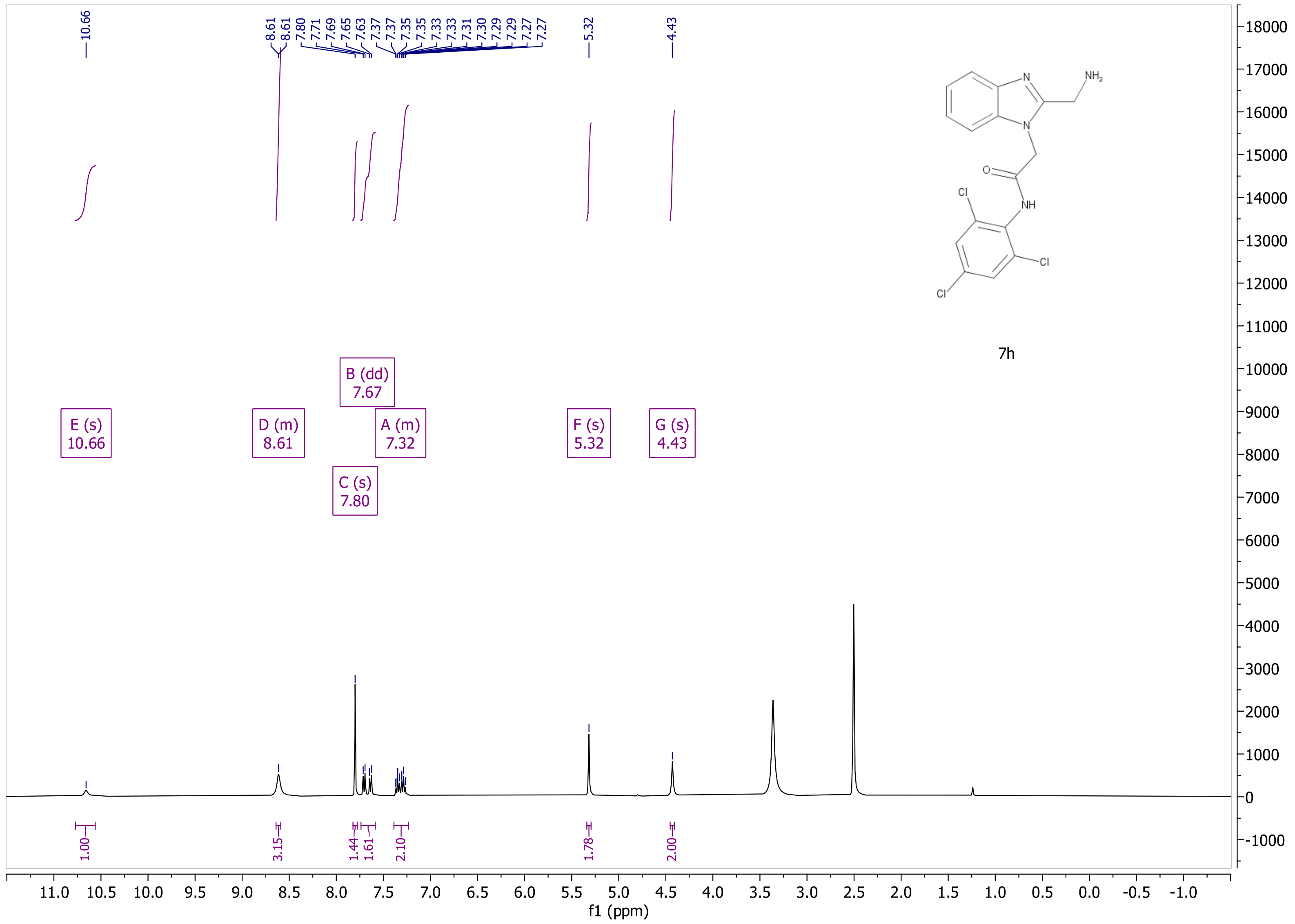
10.66
8.61
8.61
7.80
7.71
7.69
7.65
7.63
7.37
7.37
7.35
7.35
7.33
7.33
7.31
7.30
7.29
7.29
7.27
7.27
5.32
4.43
E (s)
10.66
D (m)
8.61
B (dd)
7.67
A (m)
7.32
C (s)
7.80
F (s)
5.32
G (s)
4.43
1.00
3.15
1.44
1.61
2.10
1.78
2.00
7h
f1 (ppm)

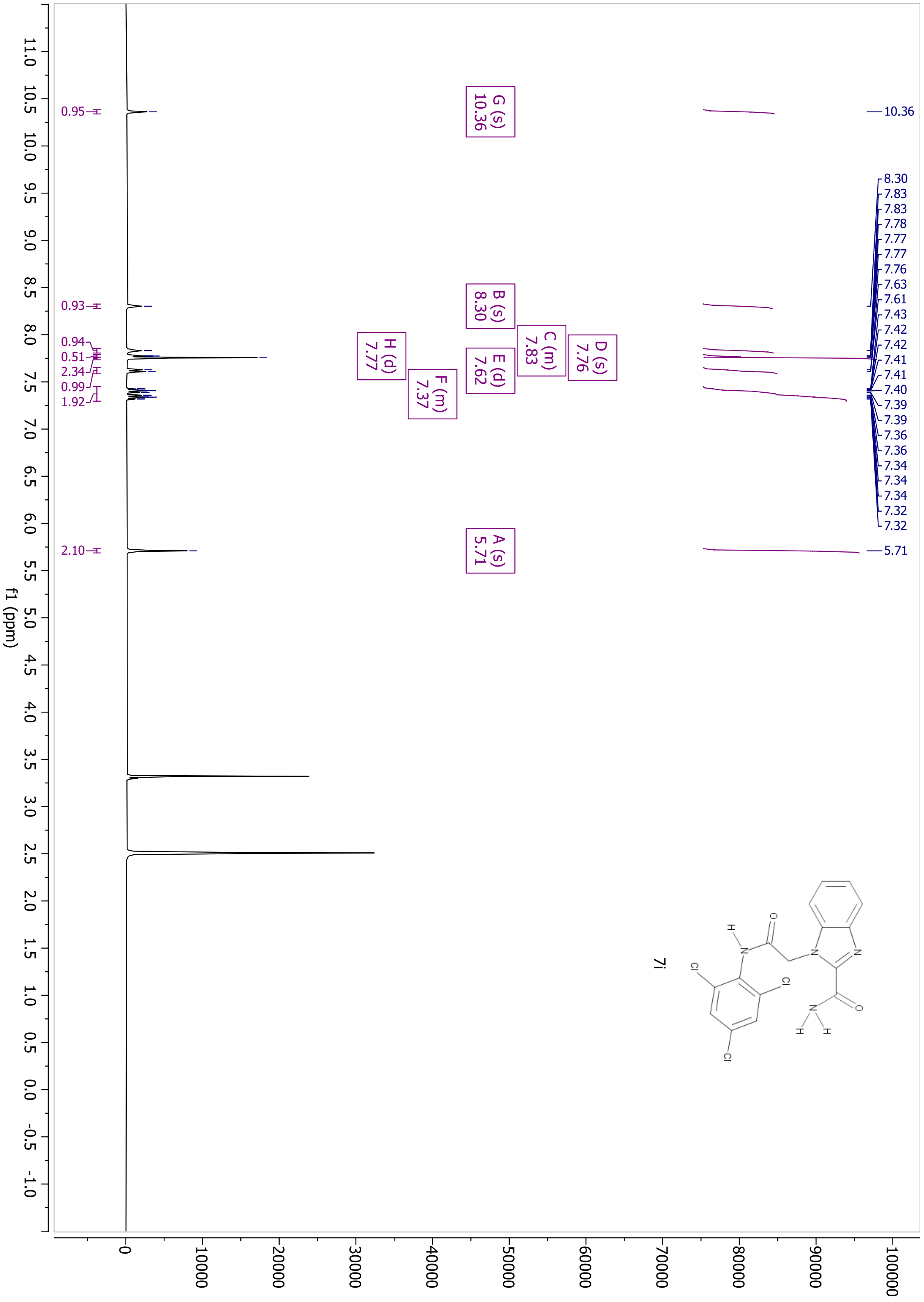
G (s)
10.36
B (s)
8.30
C (m)
7.83
D (s)
7.76
E (d)
7.62
H (d)
7.77
F (m)
7.37
A (s)
5.71
f1 (ppm)
7i

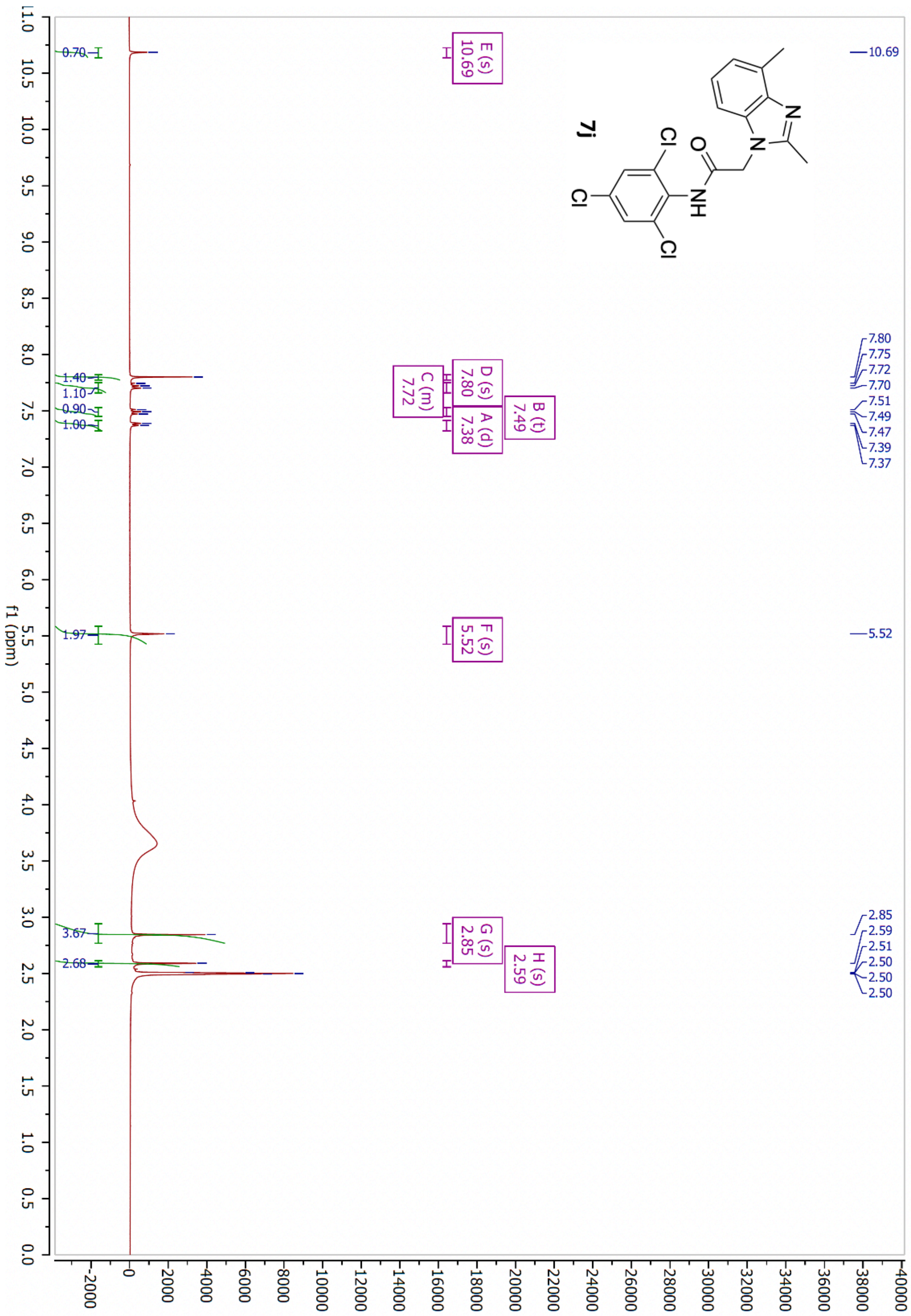
7j
E (s) 10.69
D (s) 7.80
C (m) 7.72
B (t) 7.49
A (d) 7.38
F (s) 5.52
G (s) 2.85
H (s) 2.59
10.69
7.80
7.75
7.72
7.70
7.51
7.49
7.47
7.39
7.37
5.52
2.85
2.59
2.51
2.50
2.50
2.50
0.70
1.40
1.10
0.90
1.00
1.97
3.67
2.68
f1 (ppm)

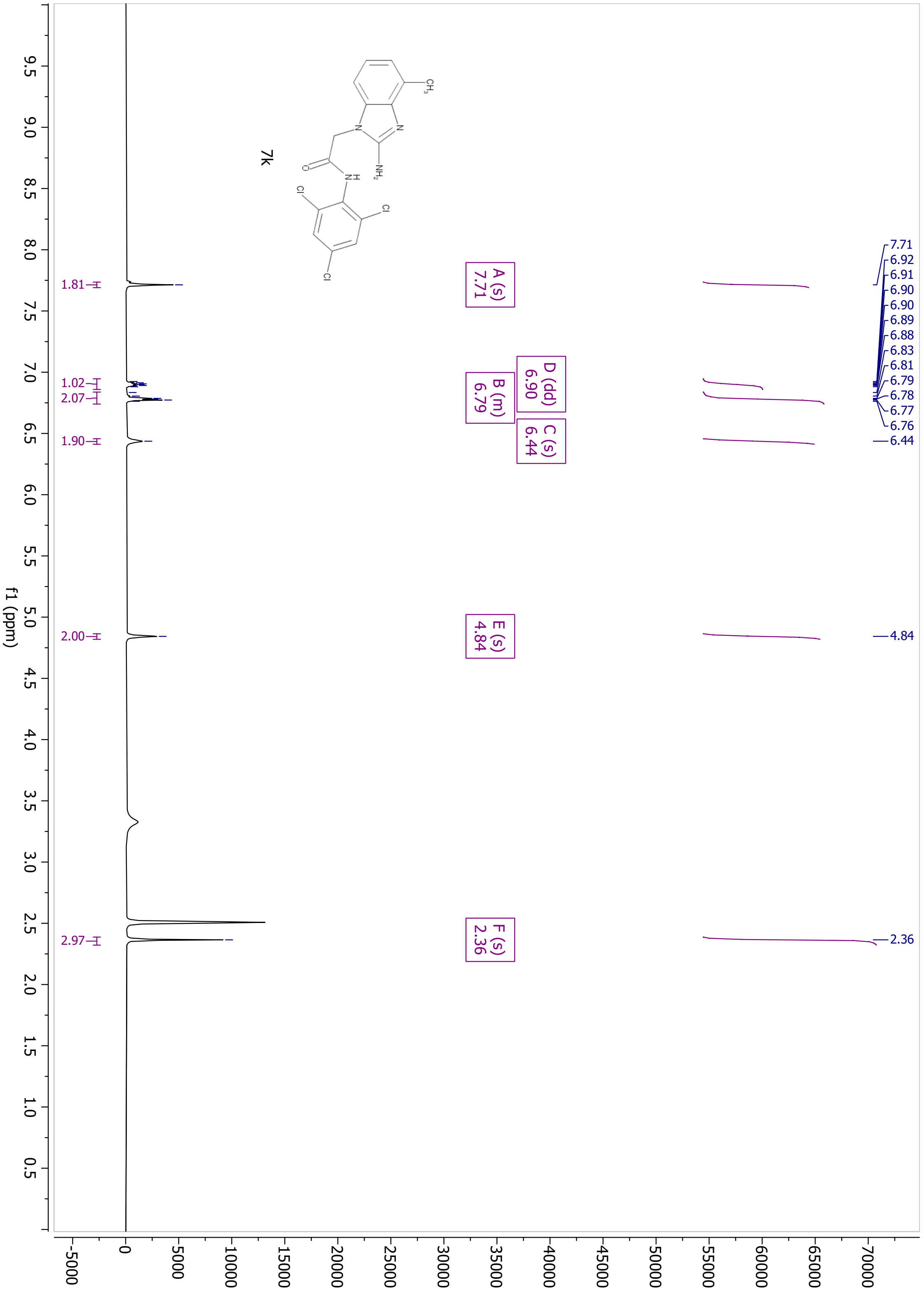
7k
f1 (ppm)
A (s) 7.71
B (m) 6.79
C (s) 6.44
D (dd) 6.90
E (s) 4.84
F (s) 2.36
1.81
1.02
2.07
1.90
2.00
2.97
7.71
6.92
6.91
6.90
6.90
6.89
6.88
6.83
6.81
6.79
6.78
6.77
6.76
6.44
4.84
2.36

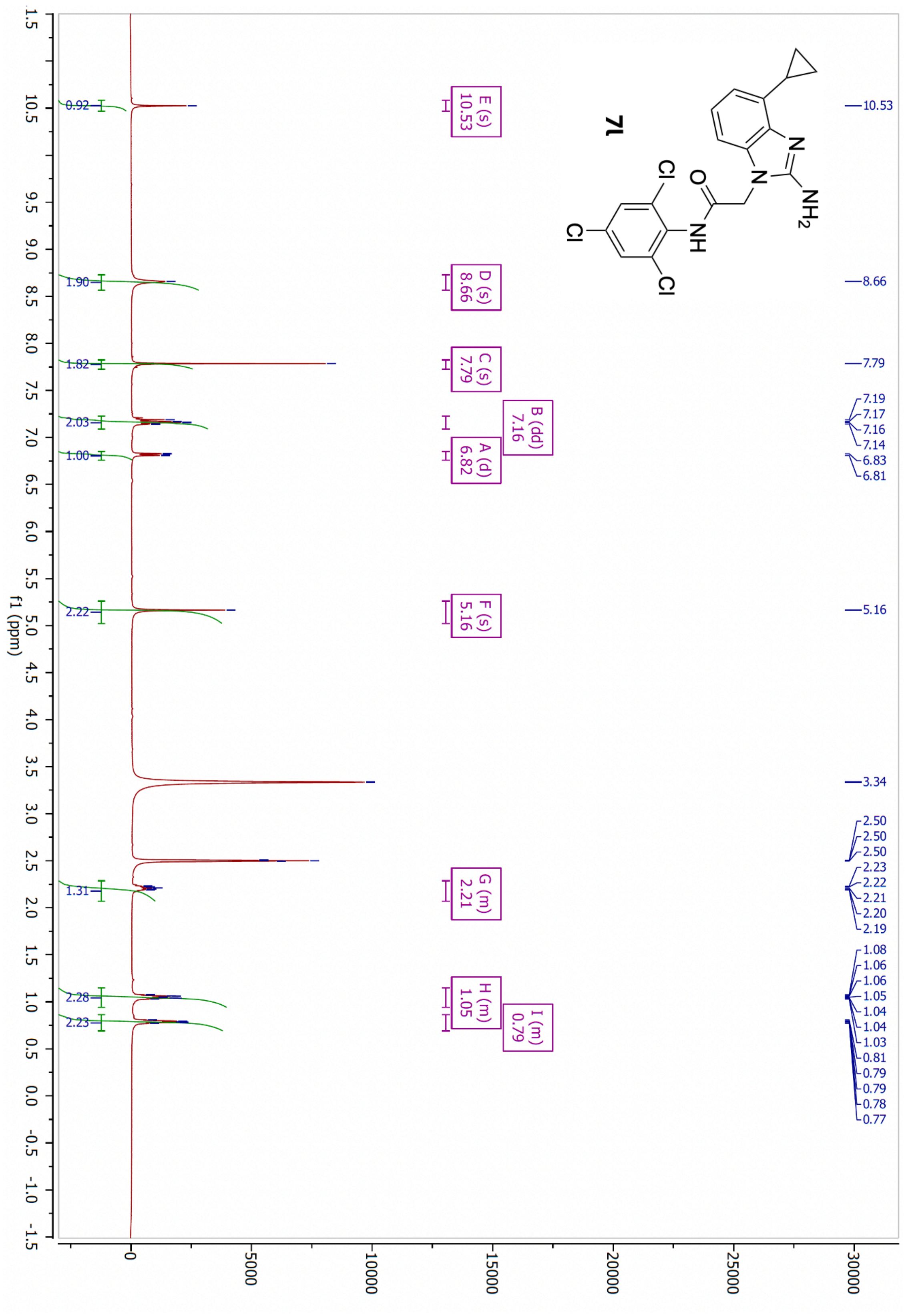
7l
NH2
N
O
Cl
NH
E (s) 10.53
D (s) 8.66
C (s) 7.79
B (dd) 7.16
A (d) 6.82
F (s) 5.16
G (m) 2.21
H (m) 1.05
I (m) 0.79
0.92
1.90
1.82
2.03
1.00
2.22
1.31
2.28
2.23
10.53
8.66
7.79
7.19
7.17
7.16
7.14
6.83
6.81
5.16
3.34
2.50
2.50
2.50
2.23
2.22
2.21
2.20
2.19
1.08
1.06
1.06
1.05
1.04
1.04
1.03
0.81
0.79
0.79
0.78
0.77
f1 (ppm)

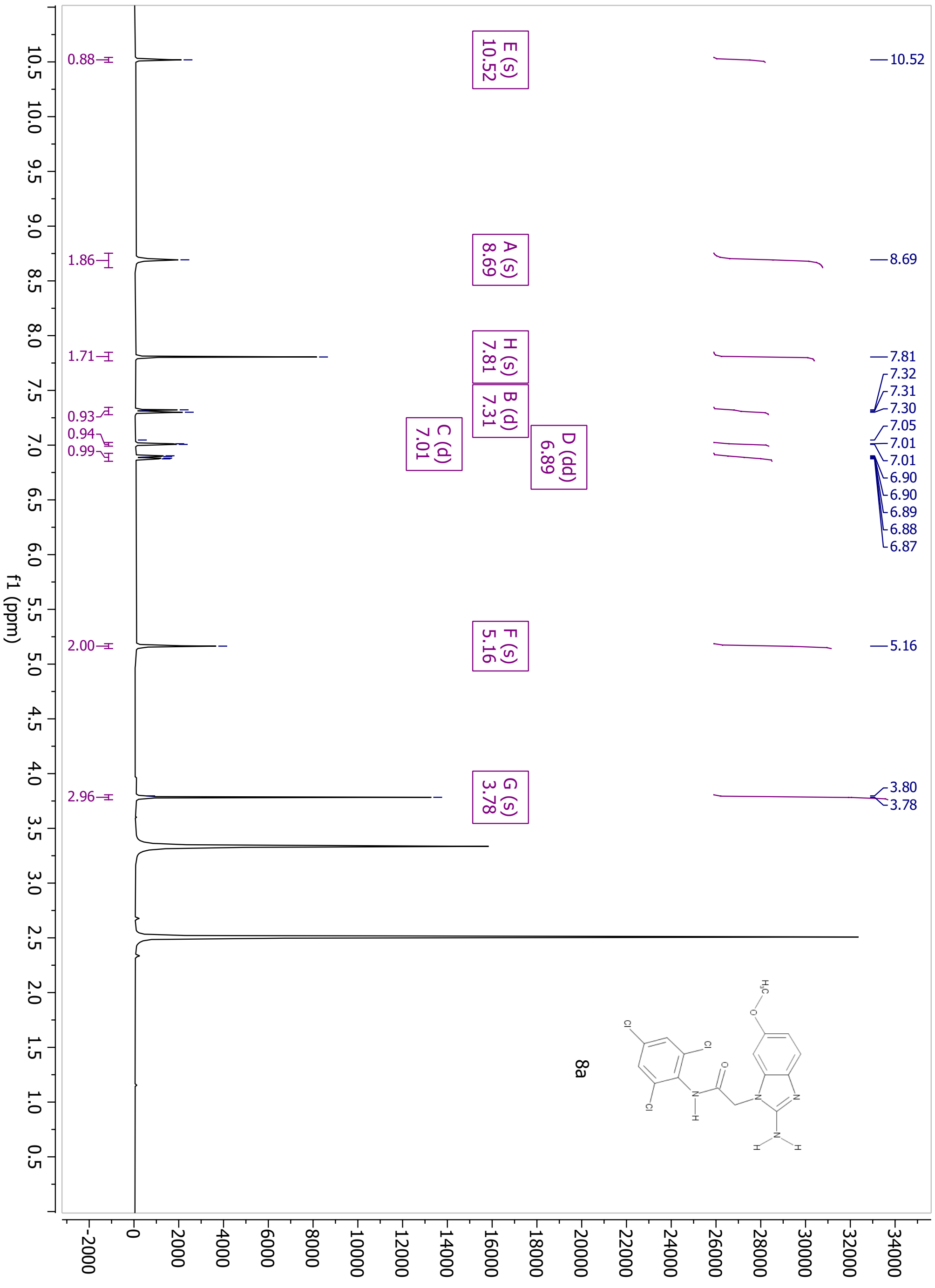
E (s) 10.52
A (s) 8.69
H (s) 7.81
B (d) 7.31
C (d) 7.01
D (dd) 6.89
F (s) 5.16
G (s) 3.78
0.88
1.86
1.71
0.93
0.94
0.99
2.00
2.96
10.52
8.69
7.81
7.32
7.31
7.30
7.05
7.01
7.01
6.90
6.90
6.89
6.88
6.87
5.16
3.80
3.78
8a
f1 (ppm)

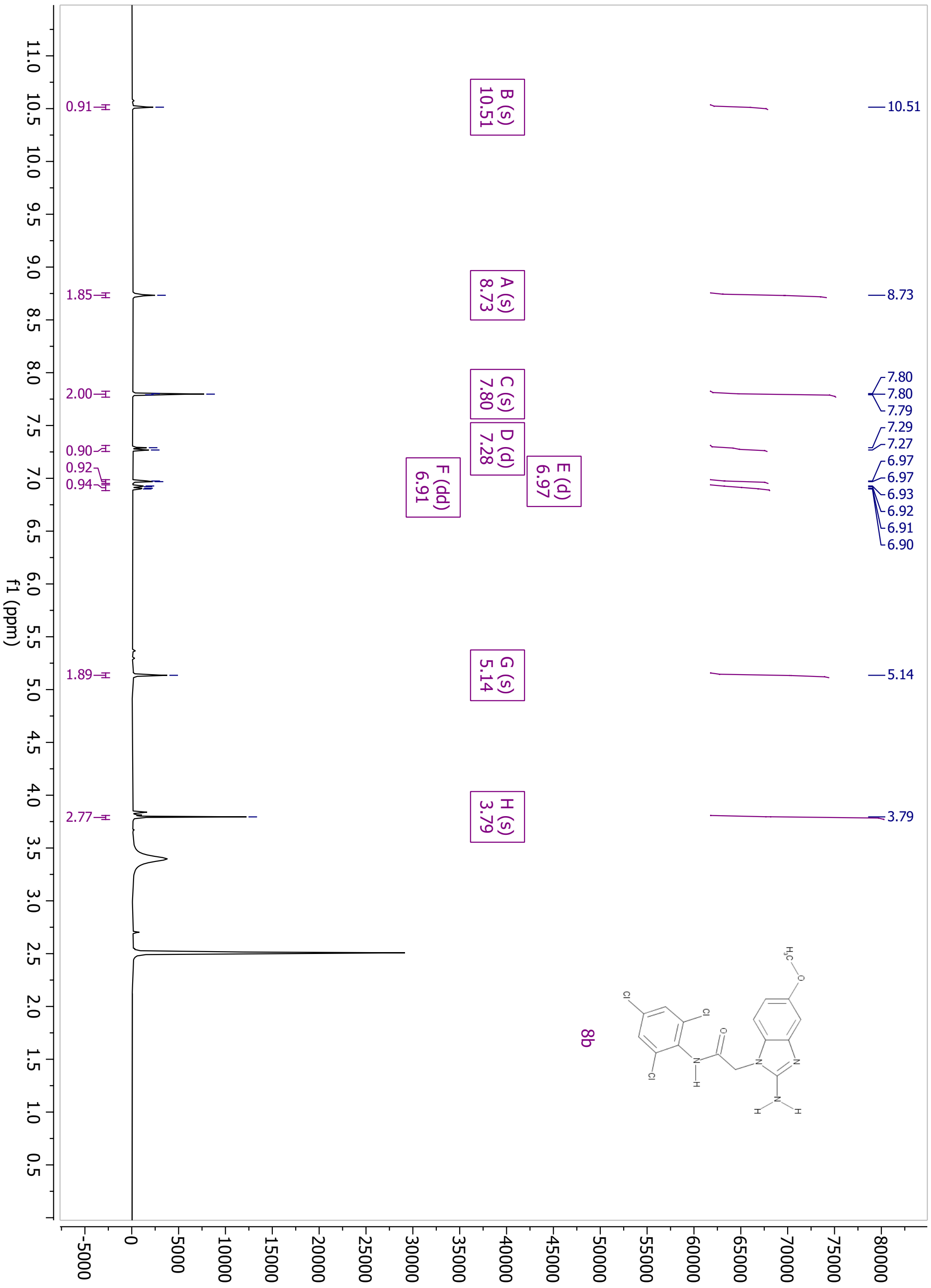

8b
B (s) 10.51
A (s) 8.73
C (s) 7.80
D (d) 7.28
E (d) 6.97
F (dd) 6.91
G (s) 5.14
H (s) 3.79
f1 (ppm)

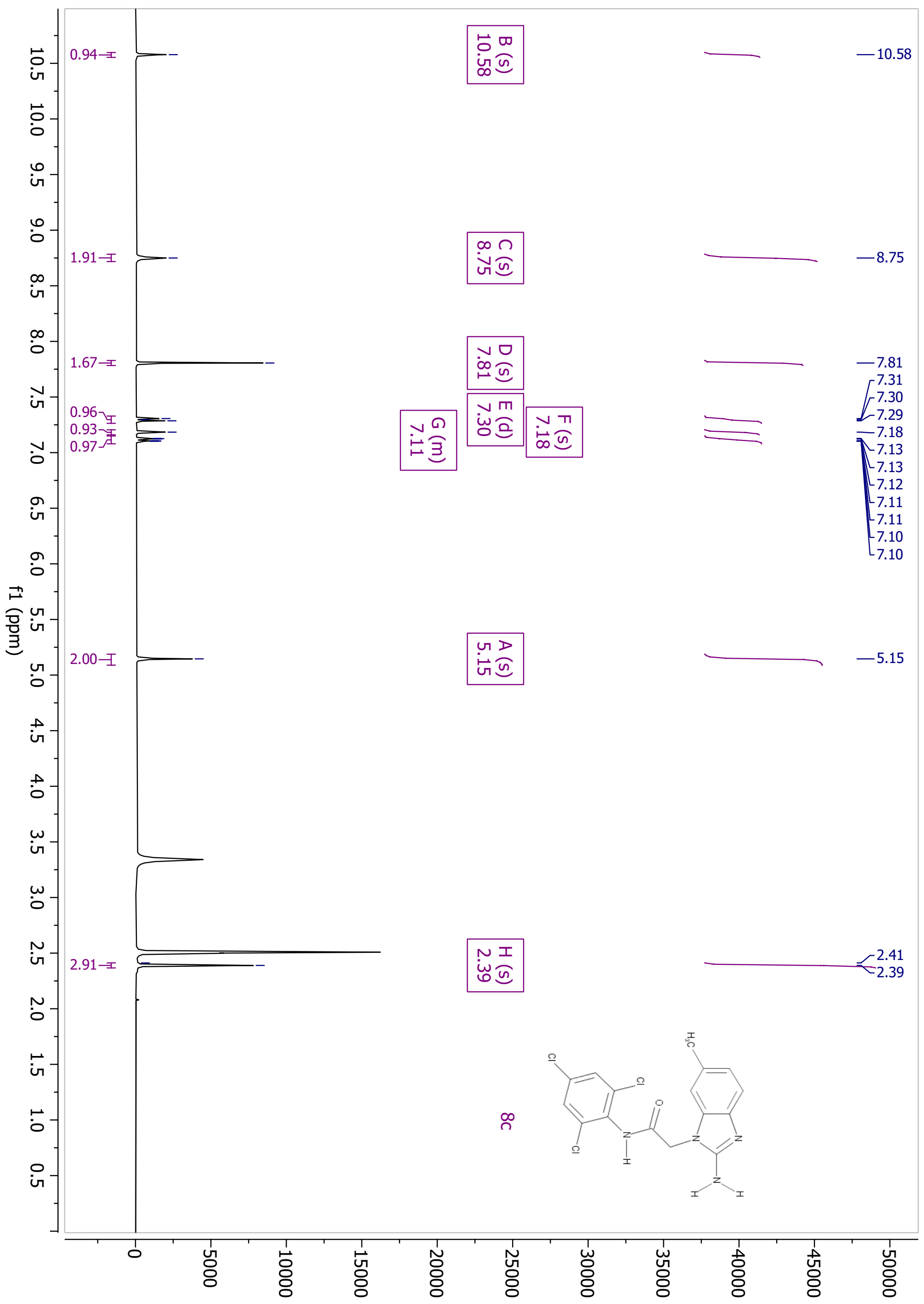

B (s) 10.58
C (s) 8.75
D (s) 7.81
E (d) 7.30
F (s) 7.18
G (m) 7.11
A (s) 5.15
H (s) 2.39
0.94
1.91
1.67
0.96
0.93
0.97
2.00
2.91
10.58
8.75
7.81
7.31
7.30
7.29
7.18
7.13
7.13
7.12
7.11
7.11
7.10
7.10
5.15
2.41
2.39
8c
f1 (ppm)

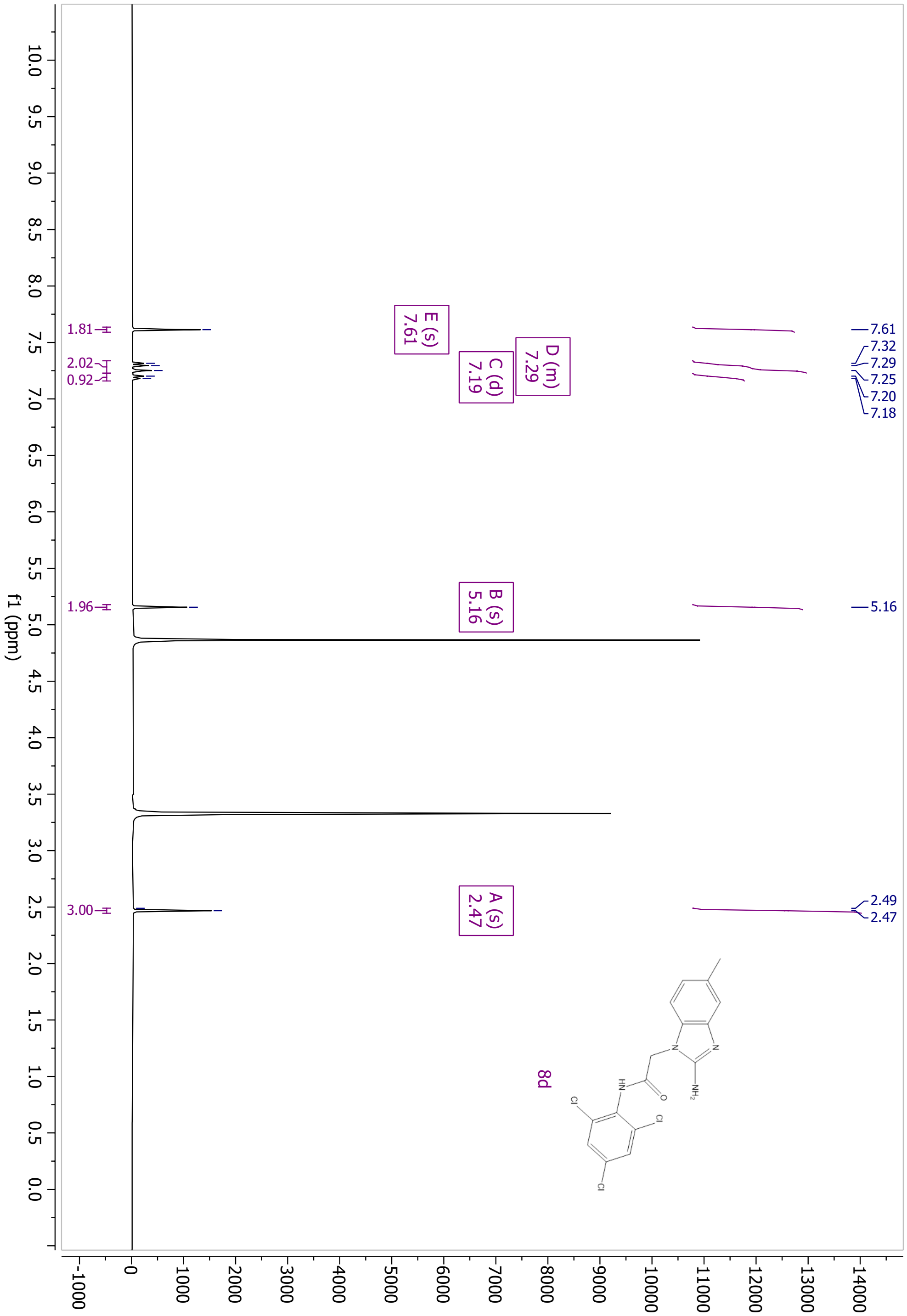
8d
E (s) 7.61
D (m) 7.29
C (d) 7.19
B (s) 5.16
A (s) 2.47
7.61
7.32
7.29
7.25
7.20
7.18
5.16
2.49
2.47
1.81
2.02
0.92
1.96
3.00
f1 (ppm)

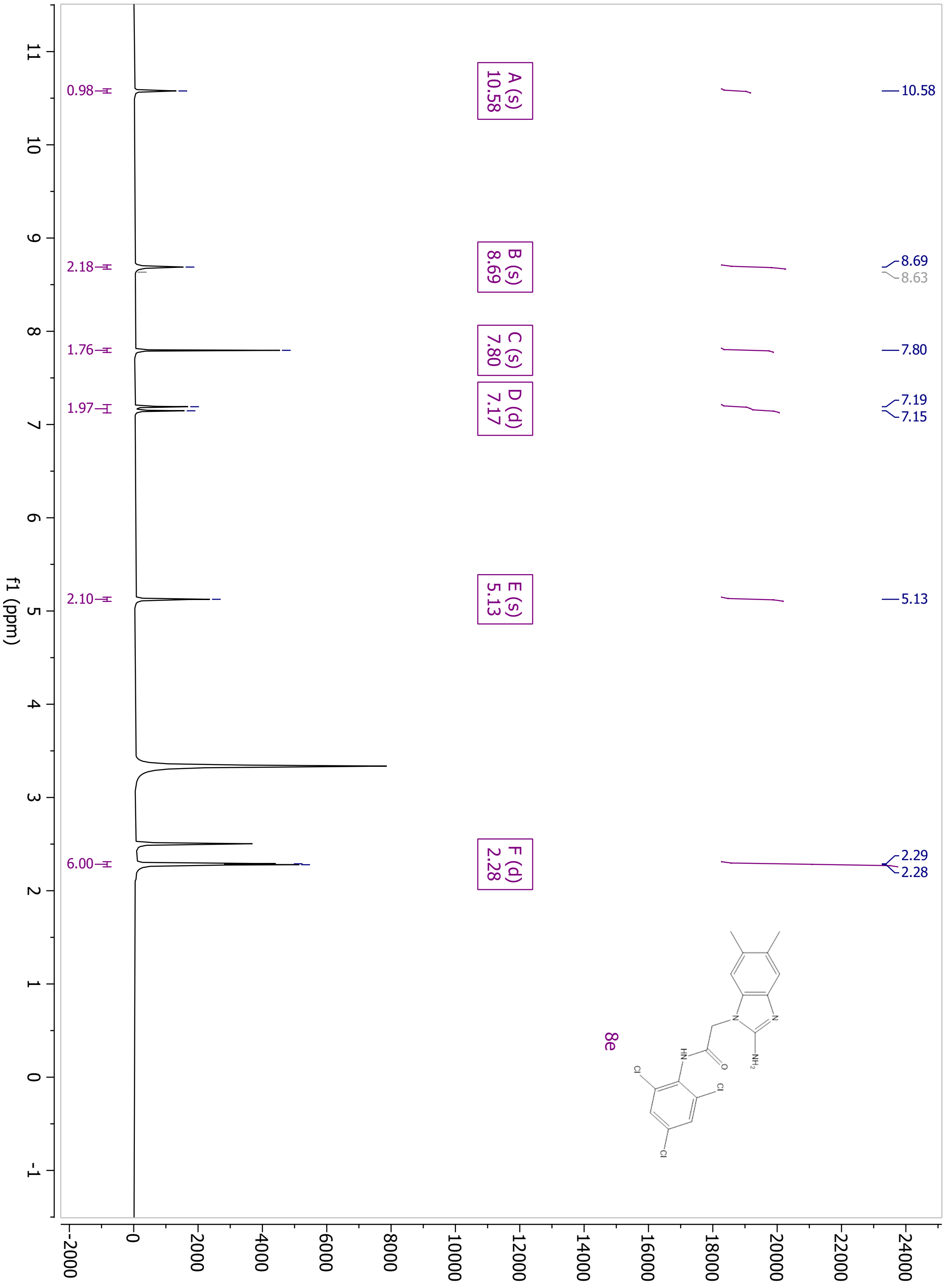
A (s)
10.58
B (s)
8.69
C (s)
7.80
D (d)
7.17
E (s)
5.13
F (d)
2.28
0.98
2.18
1.76
1.97
2.10
6.00
10.58
8.69
8.63
7.80
7.19
7.15
5.13
2.29
2.28
f1 (ppm)
8e

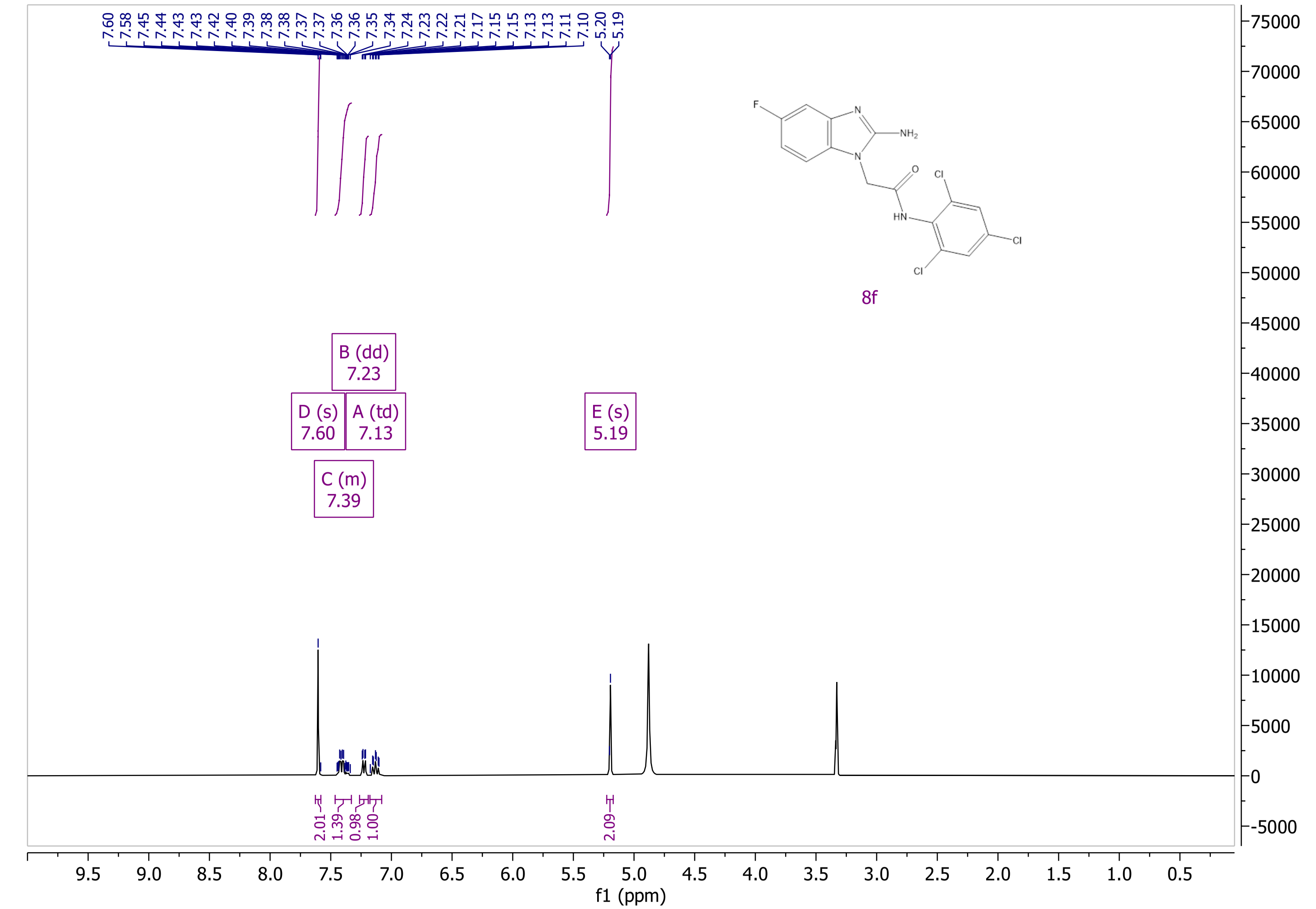
7.60
7.58
7.45
7.44
7.43
7.43
7.42
7.40
7.39
7.38
7.38
7.37
7.37
7.36
7.36
7.35
7.34
7.24
7.23
7.22
7.21
7.17
7.15
7.15
7.13
7.13
7.11
7.10
5.20
5.19
B (dd) 7.23
D (s) 7.60
A (td) 7.13
C (m) 7.39
E (s) 5.19
8f
F
N
NH2
O
Cl
HN
Cl
Cl
2.01
1.39
0.98
1.00
2.09
f1 (ppm)

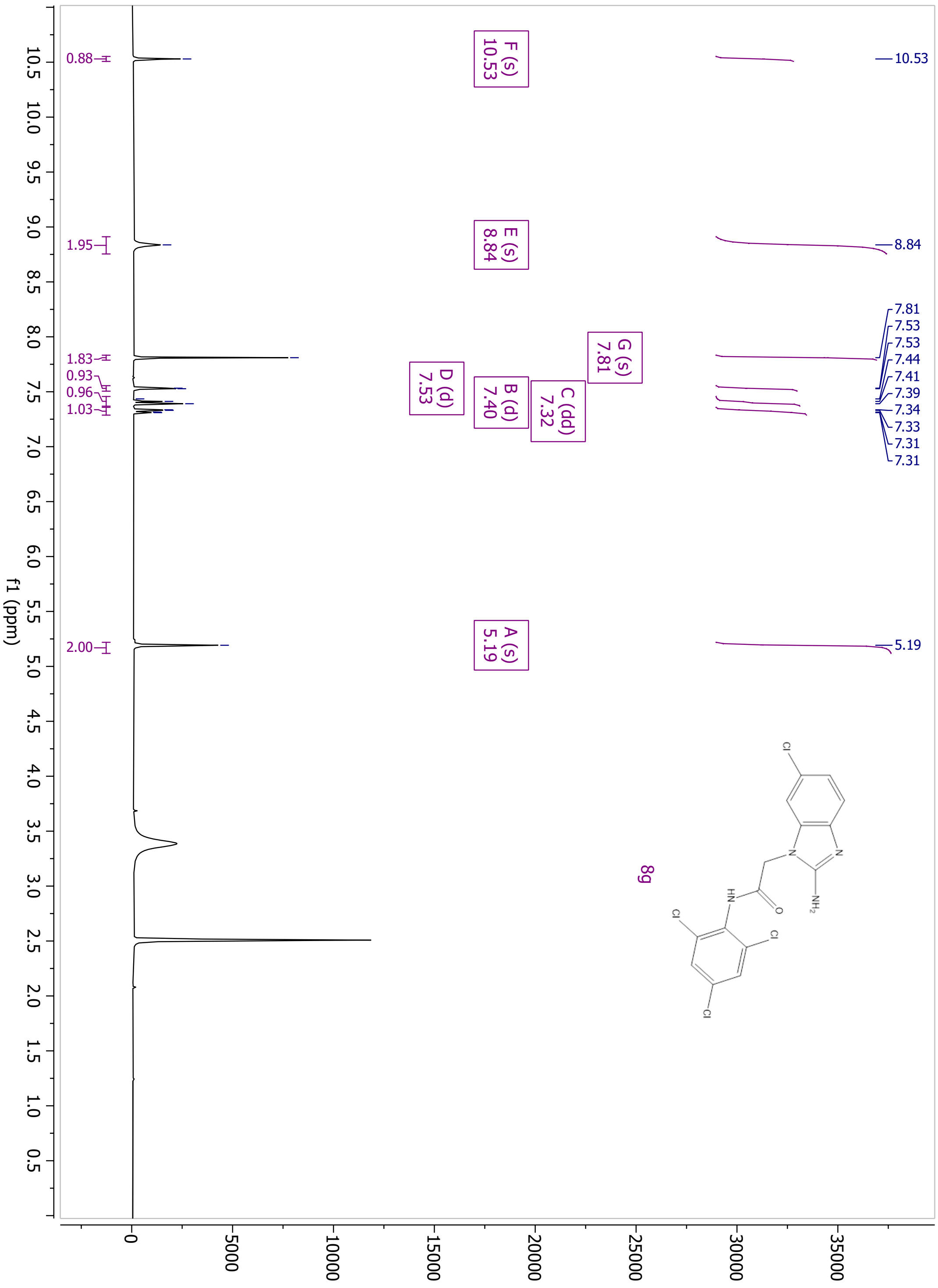
F (s) 10.53
E (s) 8.84
G (s) 7.81
D (d) 7.53
B (d) 7.40
C (dd) 7.32
A (s) 5.19
8g
f1 (ppm)

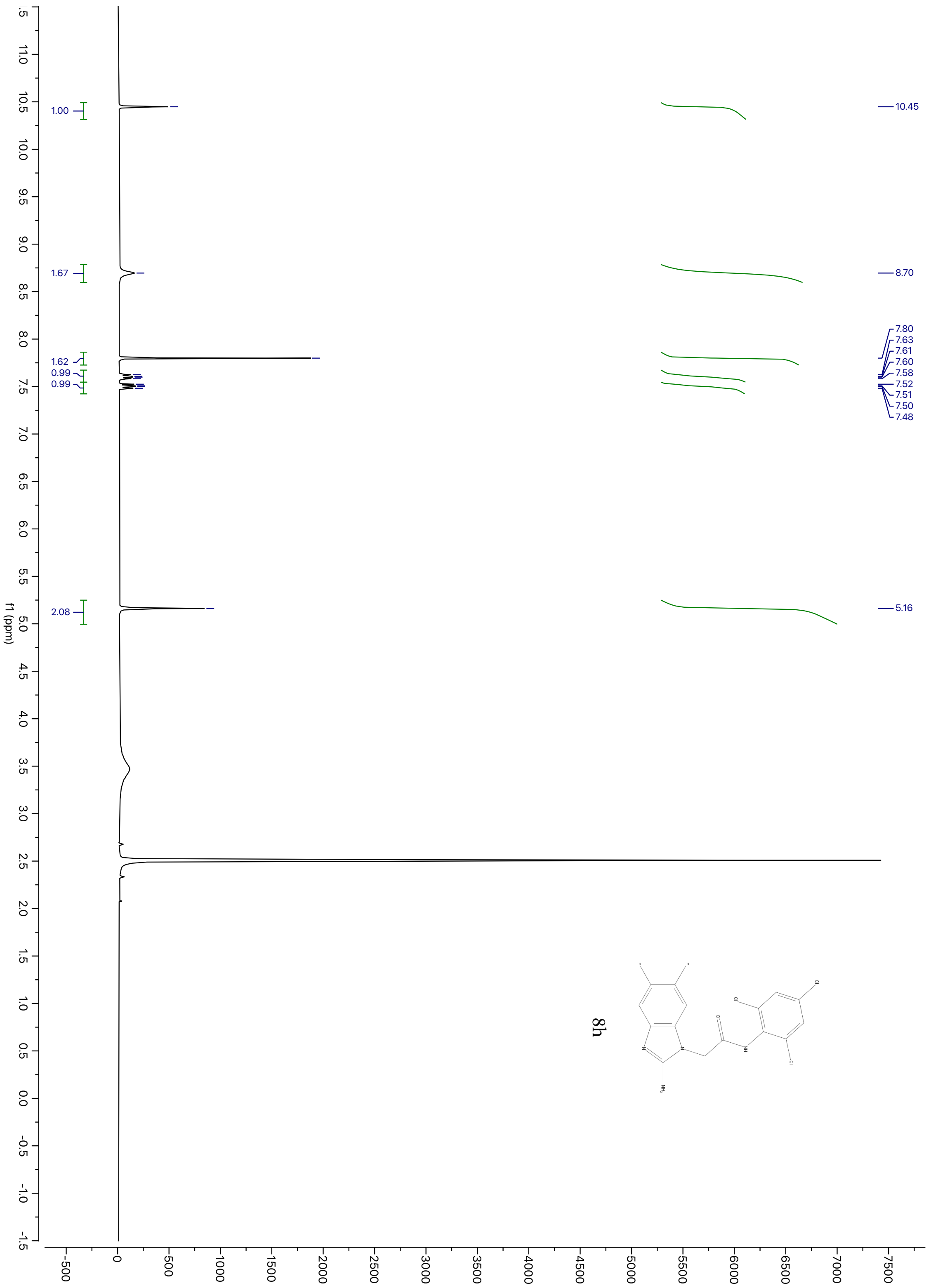
8h
10.45
8.70
7.80
7.63
7.61
7.60
7.58
7.52
7.51
7.50
7.48
5.16
1.00
1.67
1.62
0.99
0.99
2.08
f1 (ppm)

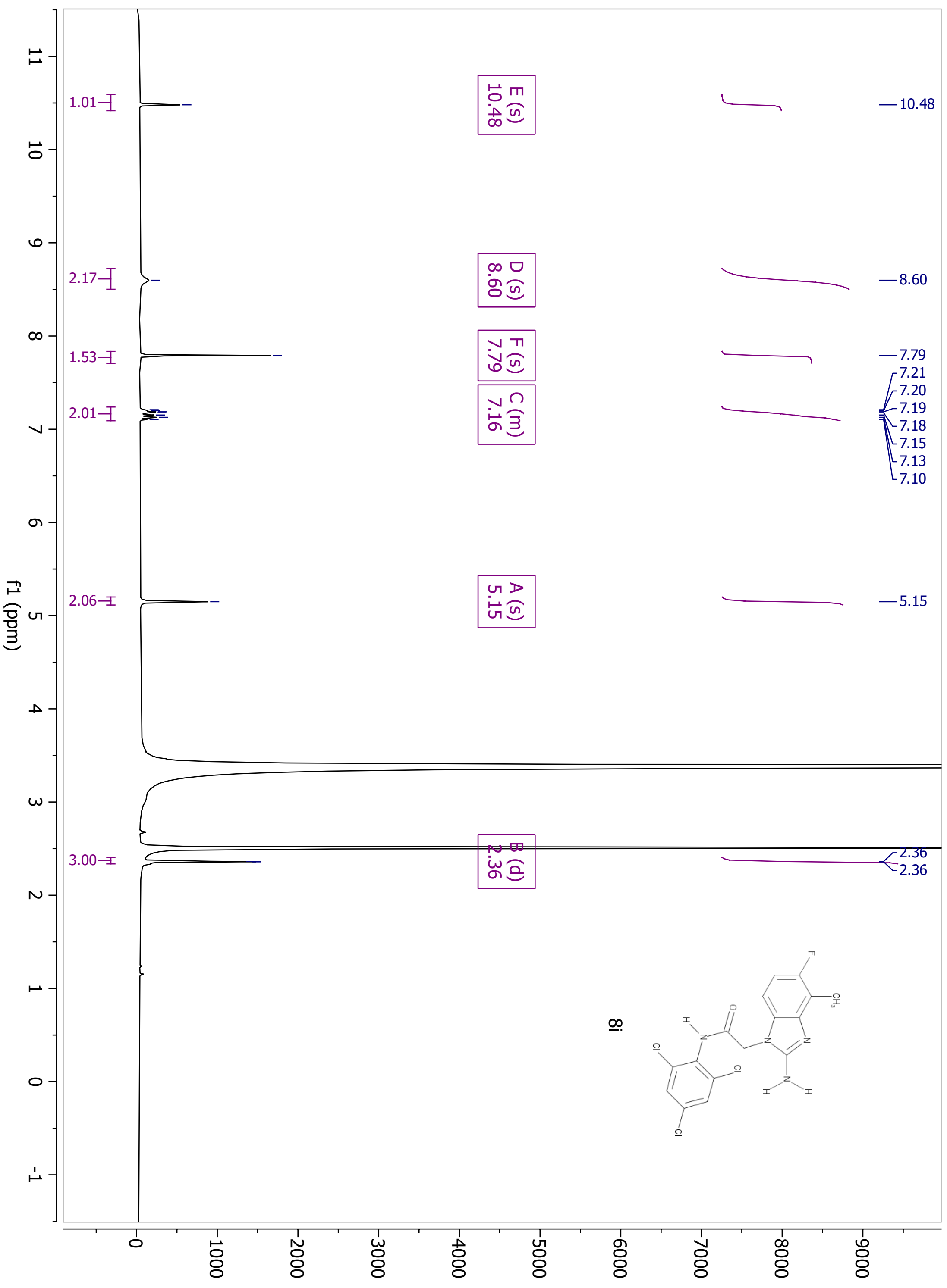
E (s) 10.48
D (s) 8.60
F (s) 7.79
C (m) 7.16
A (s) 5.15
B (d) 2.36
10.48
8.60
7.79
7.21
7.20
7.19
7.18
7.15
7.13
7.10
5.15
2.36
2.36
1.01
2.17
1.53
2.01
2.06
3.00
f1 (ppm)
8i

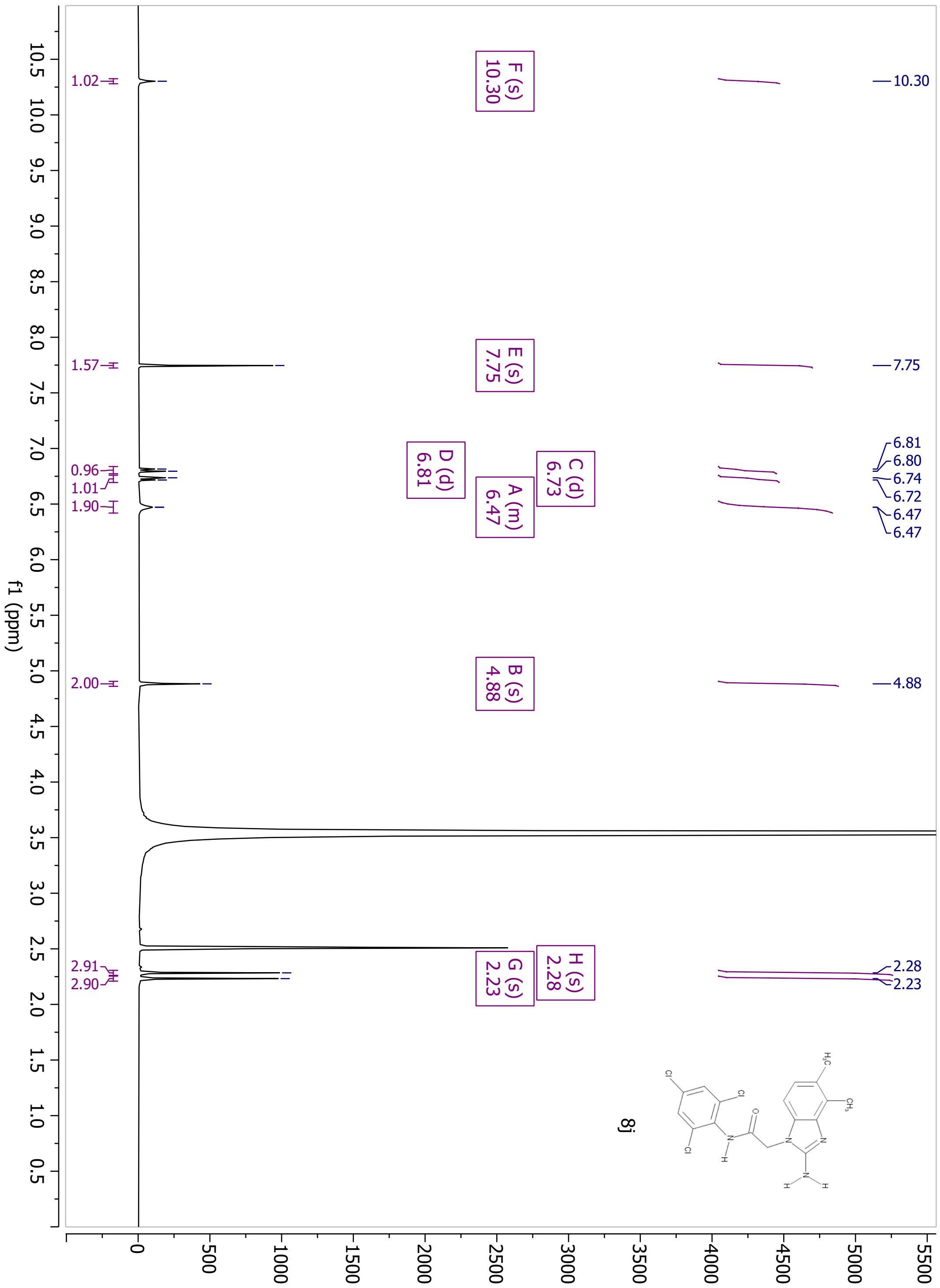
F (s) 10.30
E (s) 7.75
D (d) 6.81
C (d) 6.73
A (m) 6.47
B (s) 4.88
H (s) 2.28
G (s) 2.23
8j
f1 (ppm)

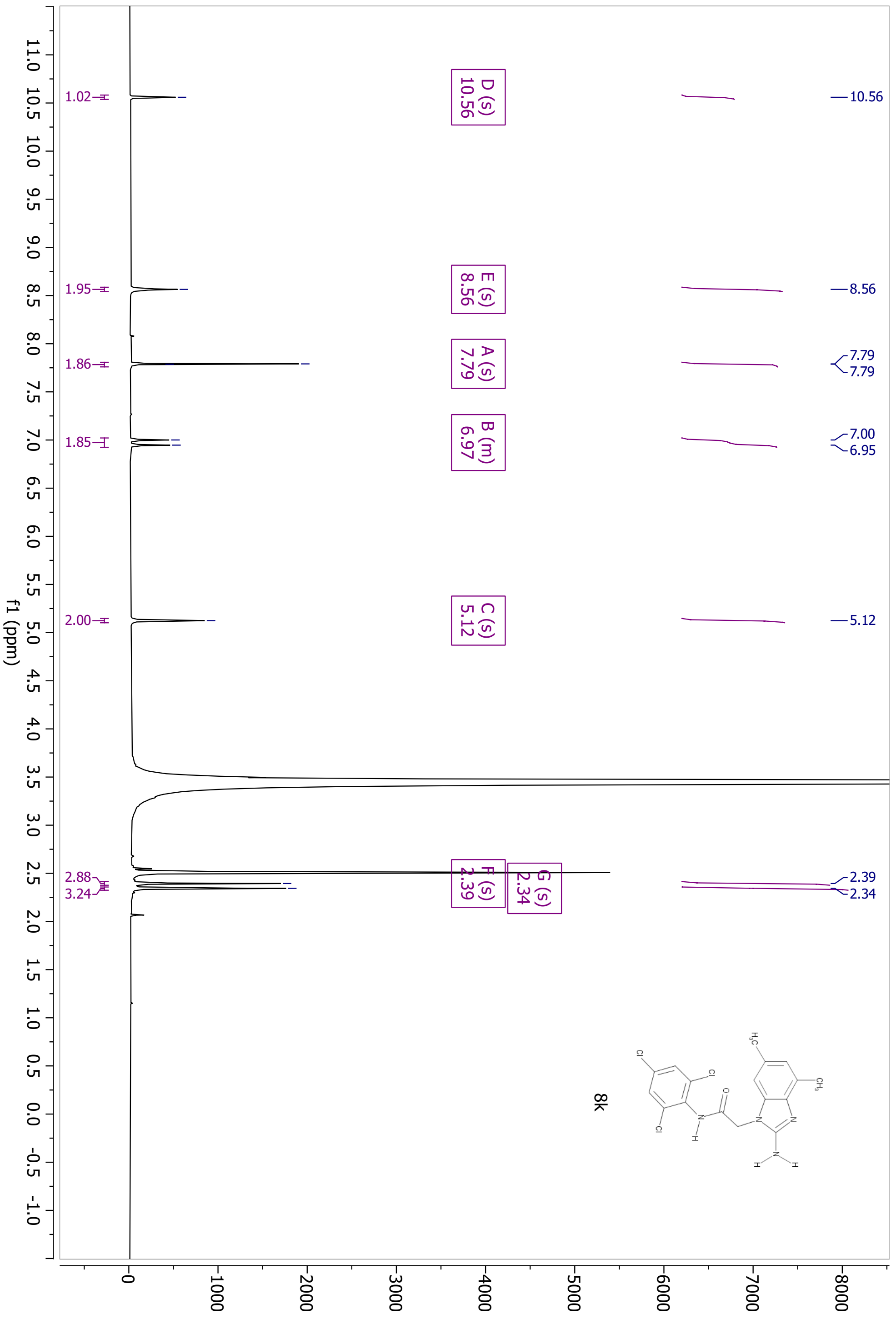

D (s)
10.56
E (s)
8.56
A (s)
7.79
B (m)
6.97
C (s)
5.12
F (s)
2.39
G (s)
2.34
1.02
1.95
1.86
1.85
2.00
2.88
3.24
10.56
8.56
7.79
7.79
7.00
6.95
5.12
2.39
2.34
f1 (ppm)
8k

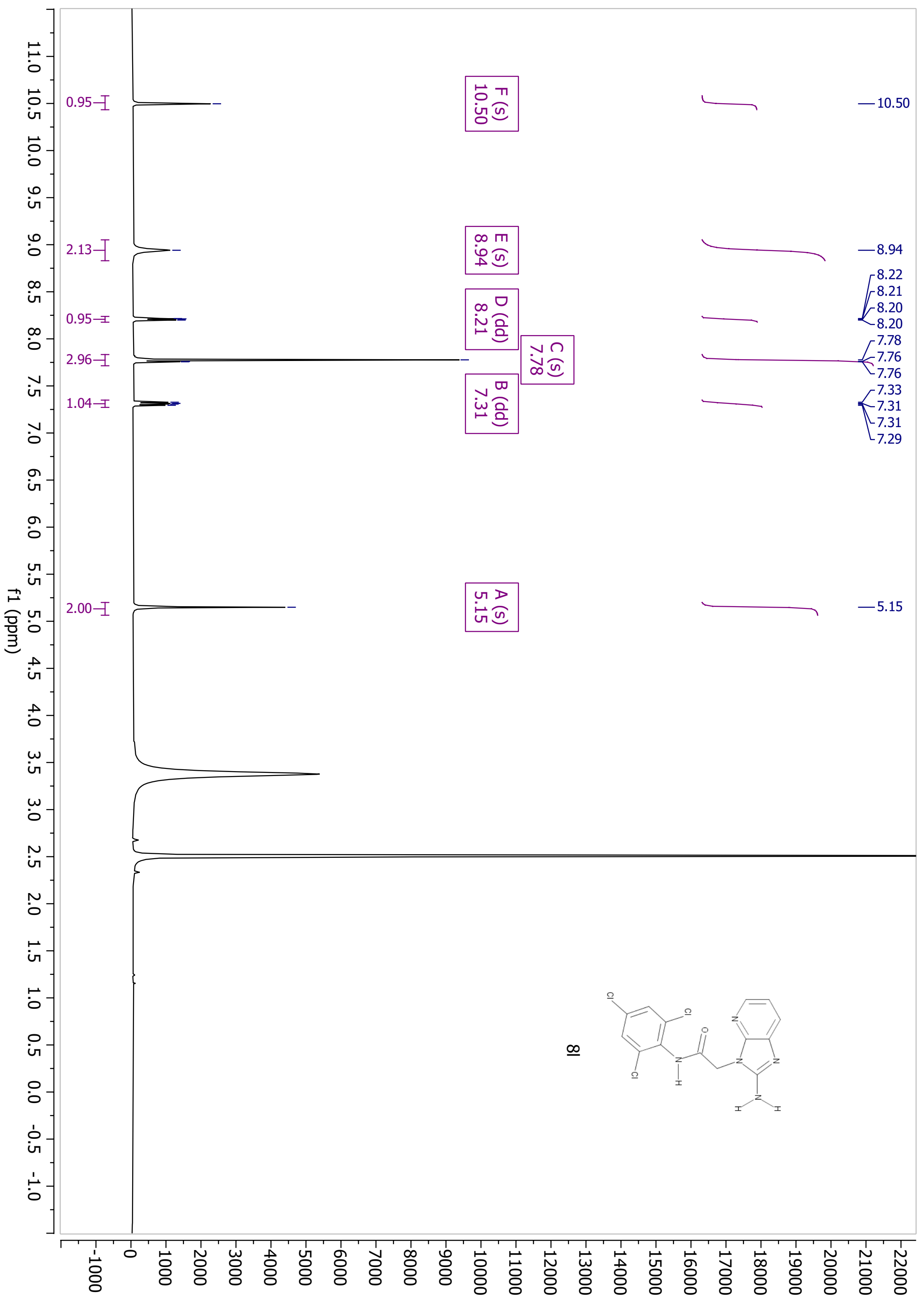

F (s) 10.50
E (s) 8.94
D (dd) 8.21
C (s) 7.78
B (dd) 7.31
A (s) 5.15
0.95
2.13
0.95
2.96
1.04
2.00
10.50
8.94
8.22
8.21
8.20
8.20
7.78
7.76
7.76
7.33
7.31
7.31
7.29
5.15
f1 (ppm)
8l

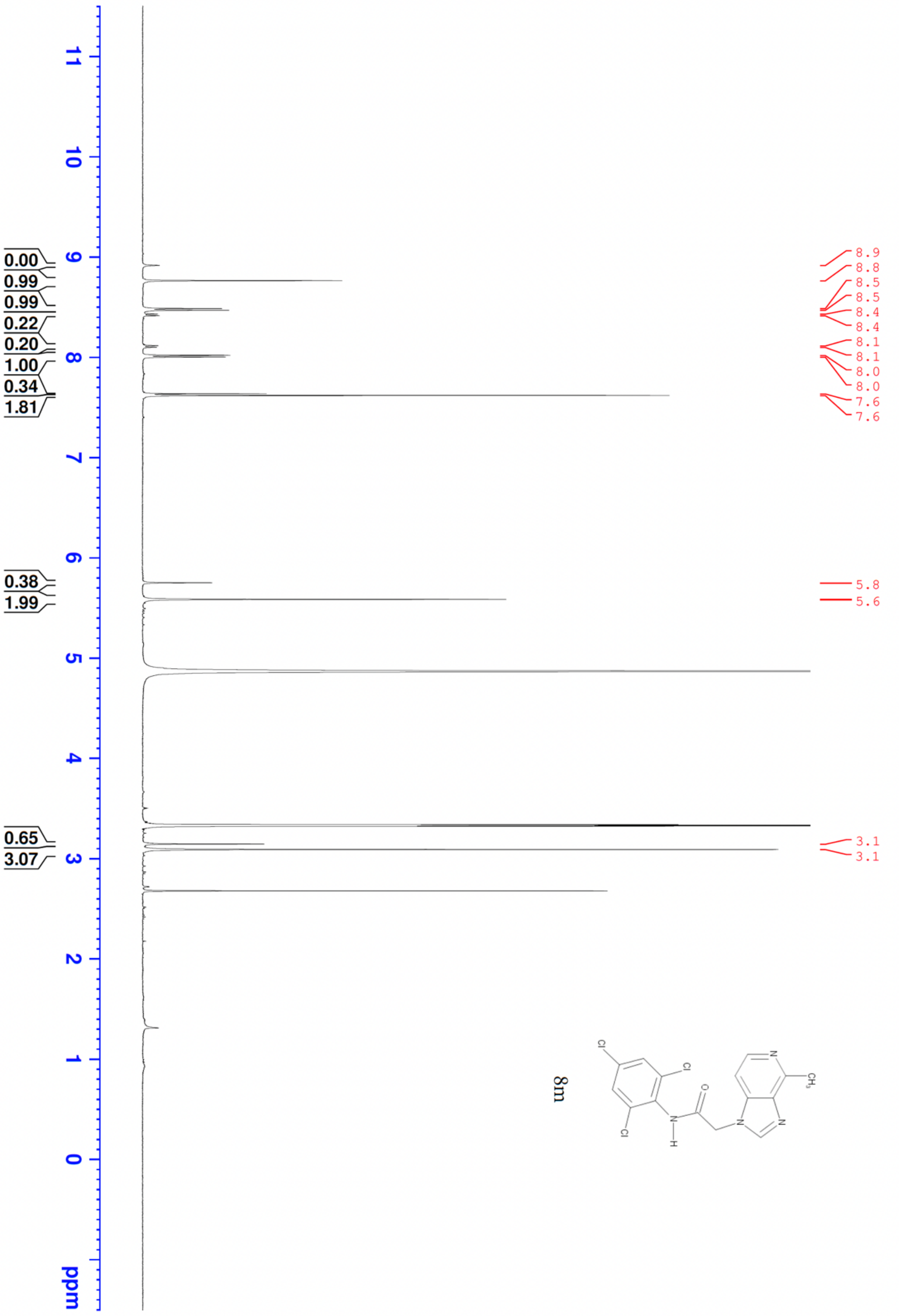
8m
ppm

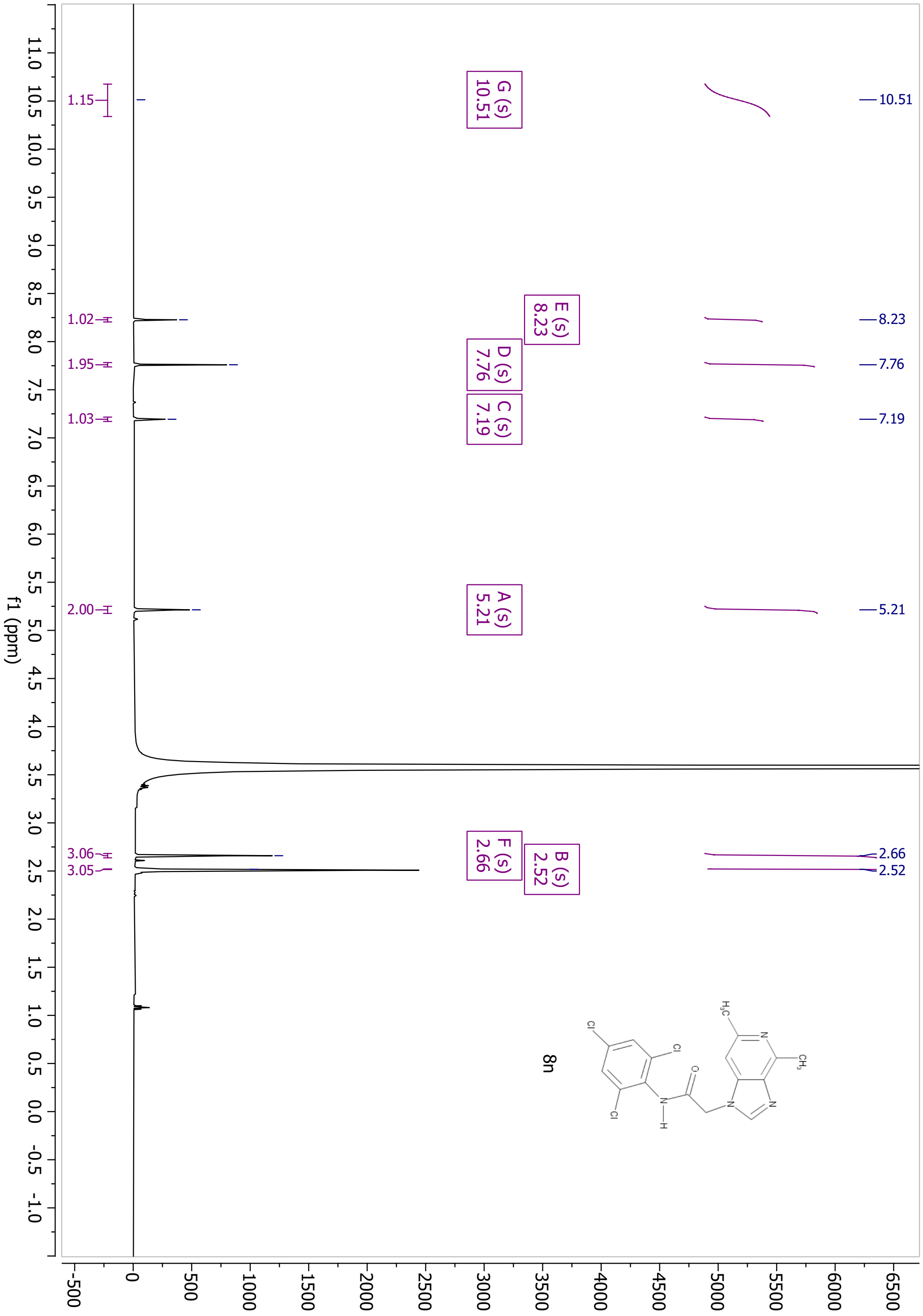
G (s)
10.51
E (s)
8.23
D (s)
7.76
C (s)
7.19
A (s)
5.21
F (s)
2.66
B (s)
2.52
1.15
1.02
1.95
1.03
2.00
3.06
3.05
10.51
8.23
7.76
7.19
5.21
2.66
2.52
f1 (ppm)
8n

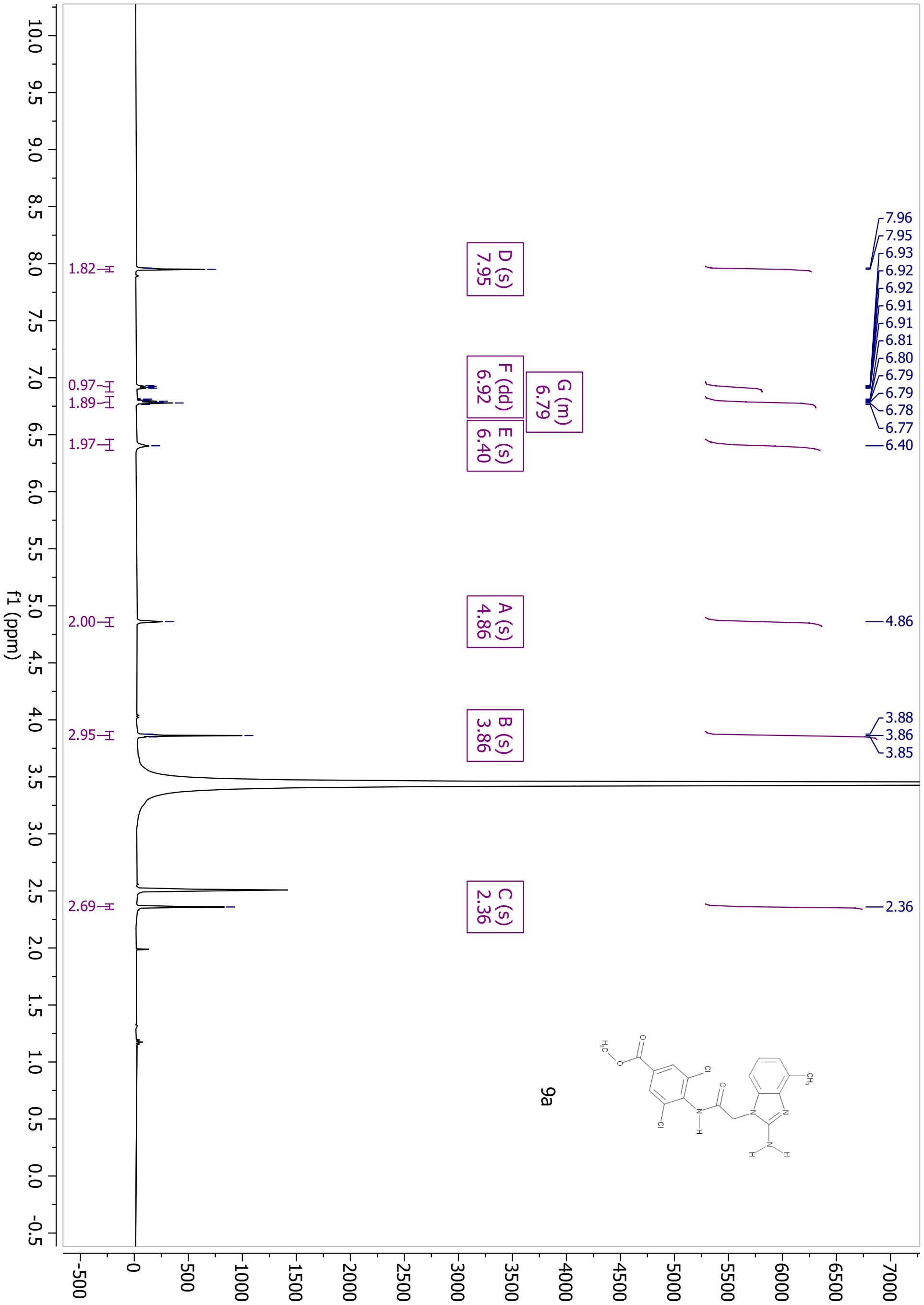
f1 (ppm)
D (s) 7.95
F (dd) 6.92
G (m) 6.79
E (s) 6.40
A (s) 4.86
B (s) 3.86
C (s) 2.36
1.82
0.97
1.89
1.97
2.00
2.95
2.69
7.96
7.95
6.93
6.92
6.92
6.91
6.91
6.81
6.80
6.79
6.79
6.78
6.77
6.40
4.86
3.88
3.86
3.85
2.36
9a

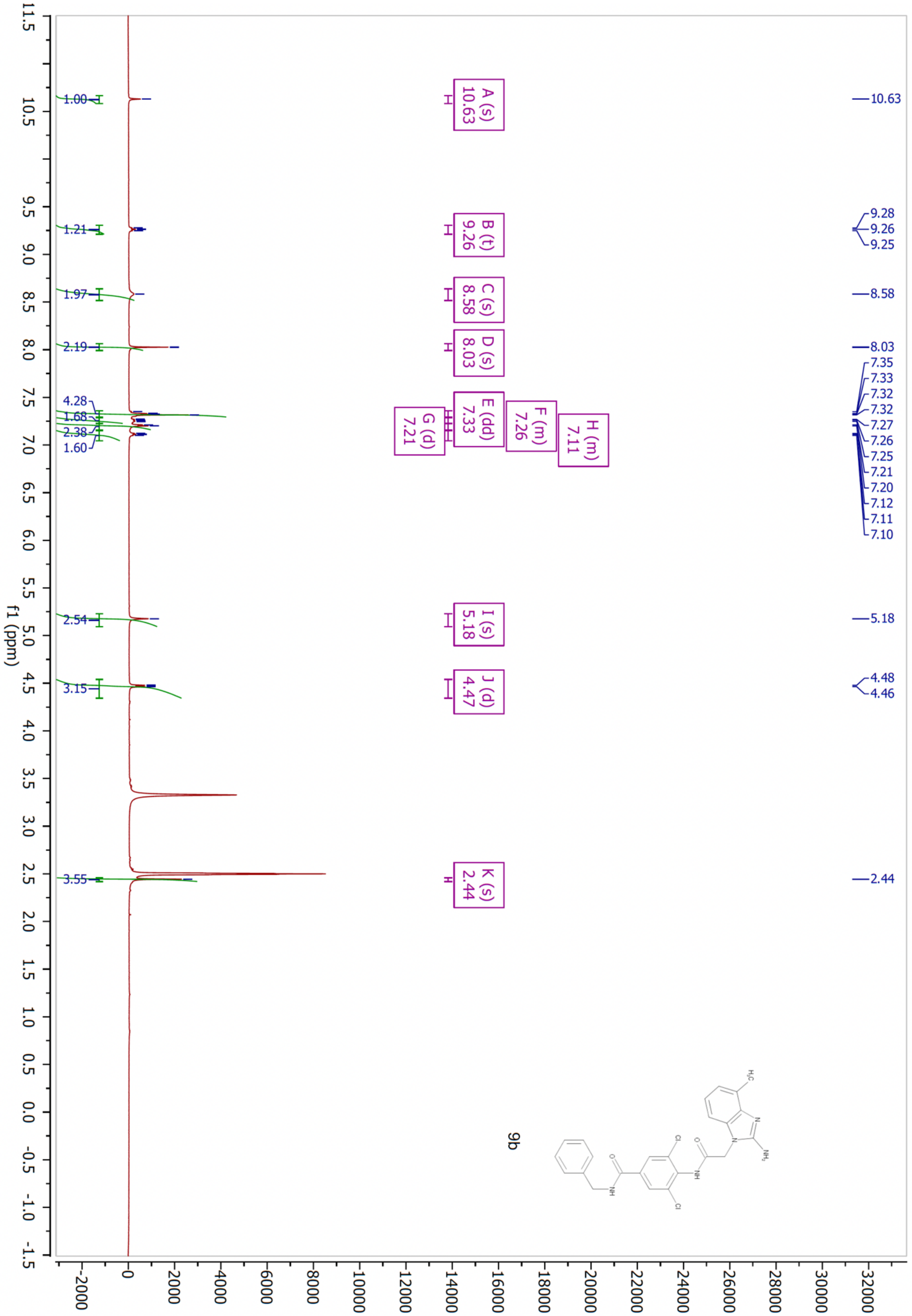

A (s)
10.63
B (t)
9.26
C (s)
8.58
D (s)
8.03
E (dd)
7.33
F (m)
7.26
G (d)
7.21
H (m)
7.11
I (s)
5.18
J (d)
4.47
K (s)
2.44
10.63
9.28
9.26
9.25
8.58
8.03
7.35
7.33
7.32
7.32
7.27
7.26
7.25
7.21
7.20
7.12
7.11
7.10
5.18
4.48
4.46
2.44
1.00
1.21
1.97
2.19
4.28
1.68
2.38
1.60
2.54
3.15
3.55
f1 (ppm)
9b

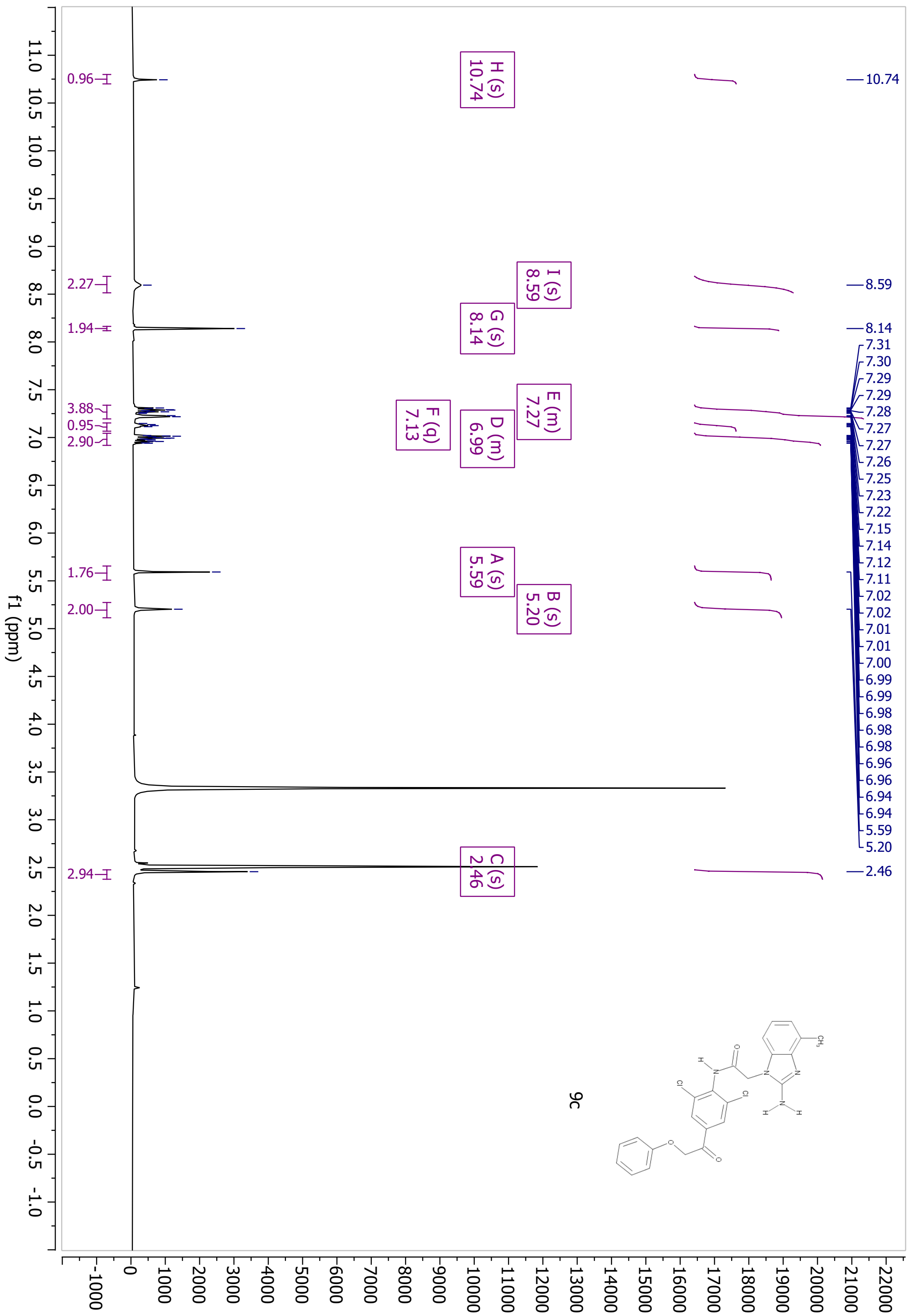
f1 (ppm)
9c
H (s) 10.74
I (s) 8.59
G (s) 8.14
E (m) 7.27
F (q) 7.13
D (m) 6.99
A (s) 5.59
B (s) 5.20
C (s) 2.46

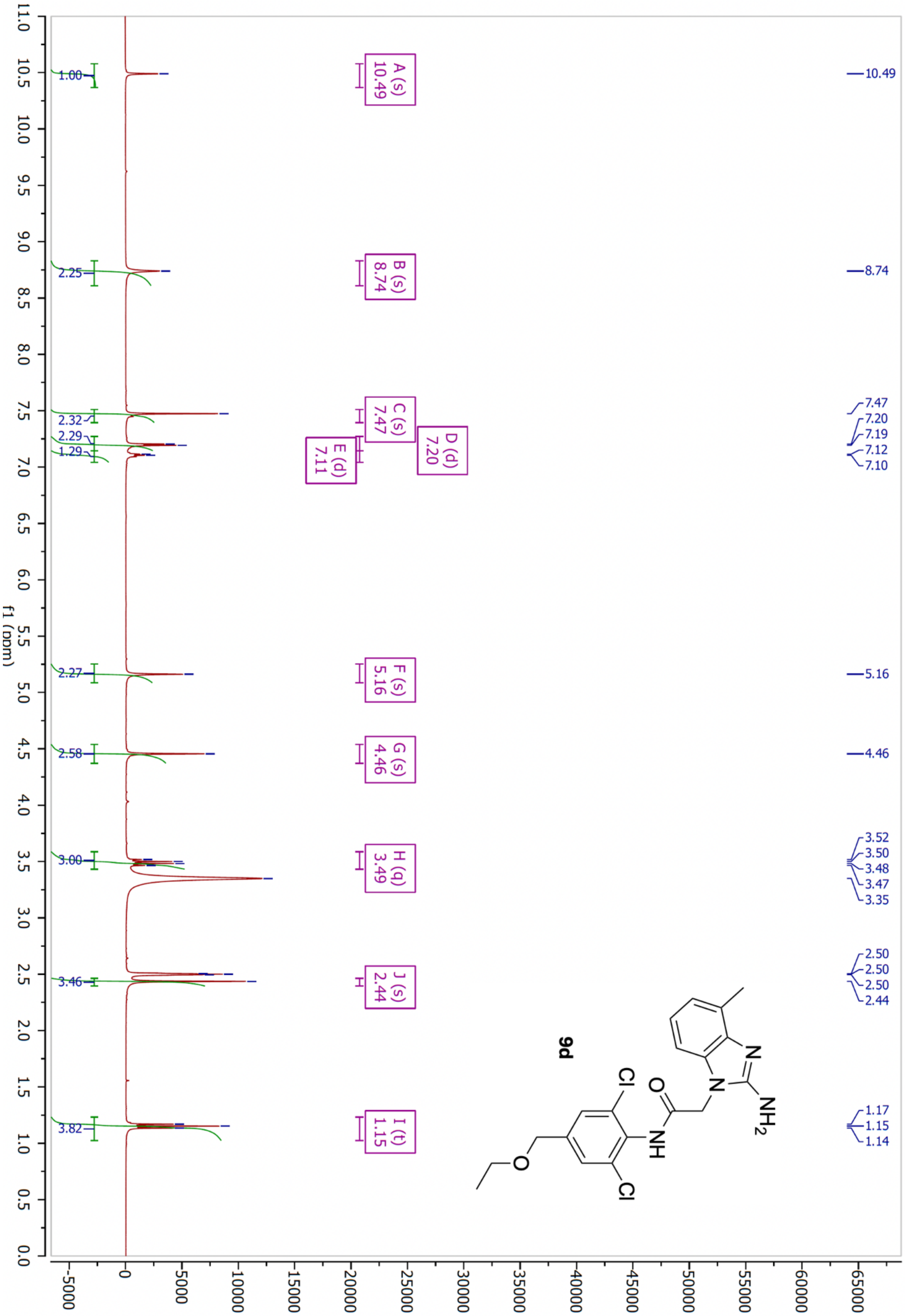

A (s) 10.49
B (s) 8.74
C (s) 7.47
D (d) 7.20
E (d) 7.11
F (s) 5.16
G (s) 4.46
H (q) 3.49
J (s) 2.44
I (t) 1.15
1.00
2.25
2.32
2.29
1.29
2.27
2.58
3.00
3.46
3.82
10.49
8.74
7.47
7.20
7.19
7.12
7.10
5.16
4.46
3.52
3.50
3.48
3.47
3.35
2.50
2.50
2.50
2.44
1.17
1.15
1.14
9d
f1 (ppm)

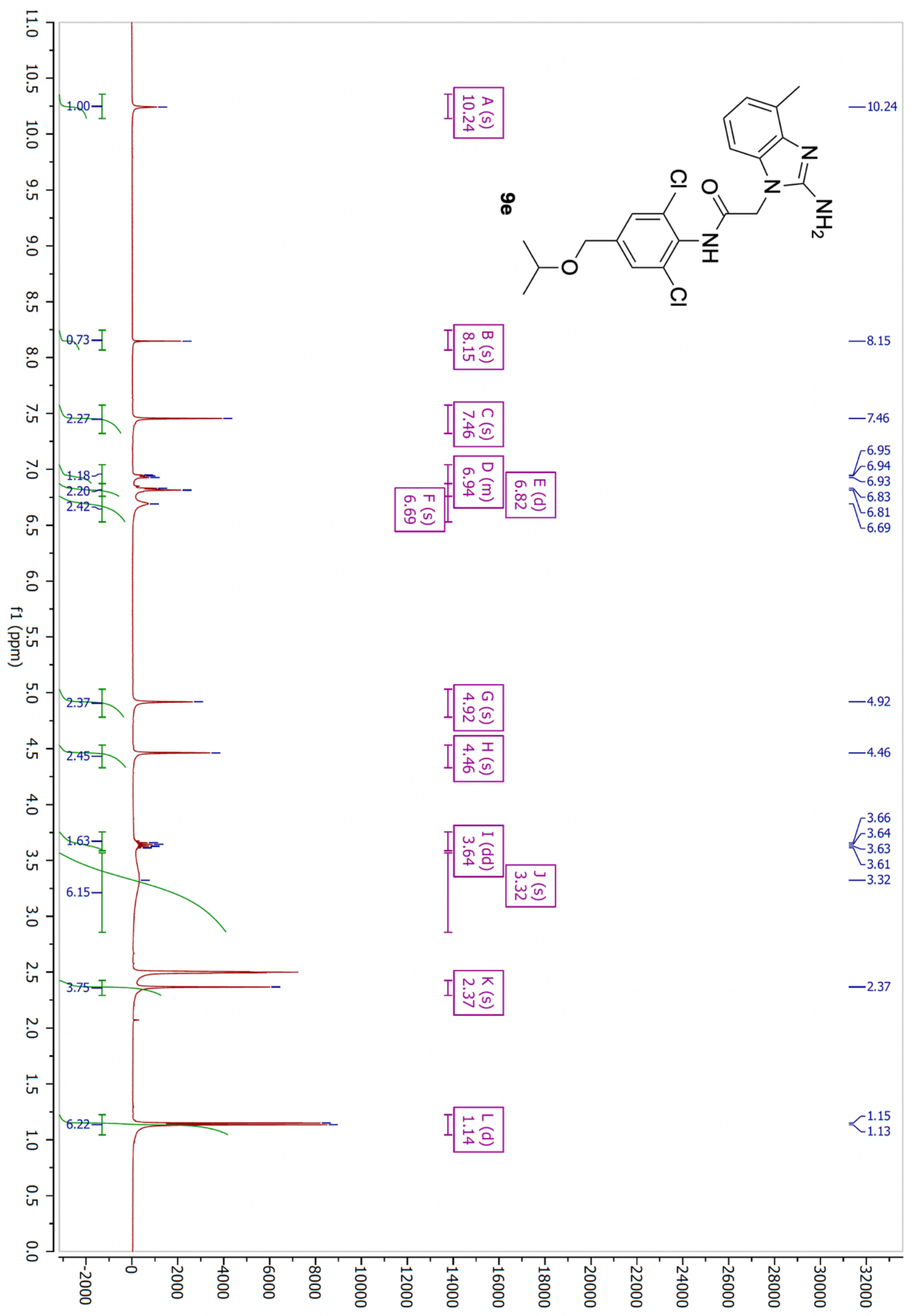
9e
NH2
N
N
O
NH
Cl
Cl
O
f1 (ppm)
A (s) 10.24
B (s) 8.15
C (s) 7.46
D (m) 6.94
E (d) 6.82
F (s) 6.69
G (s) 4.92
H (s) 4.46
I (dd) 3.64
J (s) 3.32
K (s) 2.37
L (d) 1.14
1.00
0.73
2.27
1.18
2.20
2.42
2.37
2.45
1.63
6.15
3.75
6.22
10.24
8.15
7.46
6.95
6.94
6.93
6.83
6.81
6.69
4.92
4.46
3.66
3.64
3.63
3.61
3.32
2.37
1.15
1.13

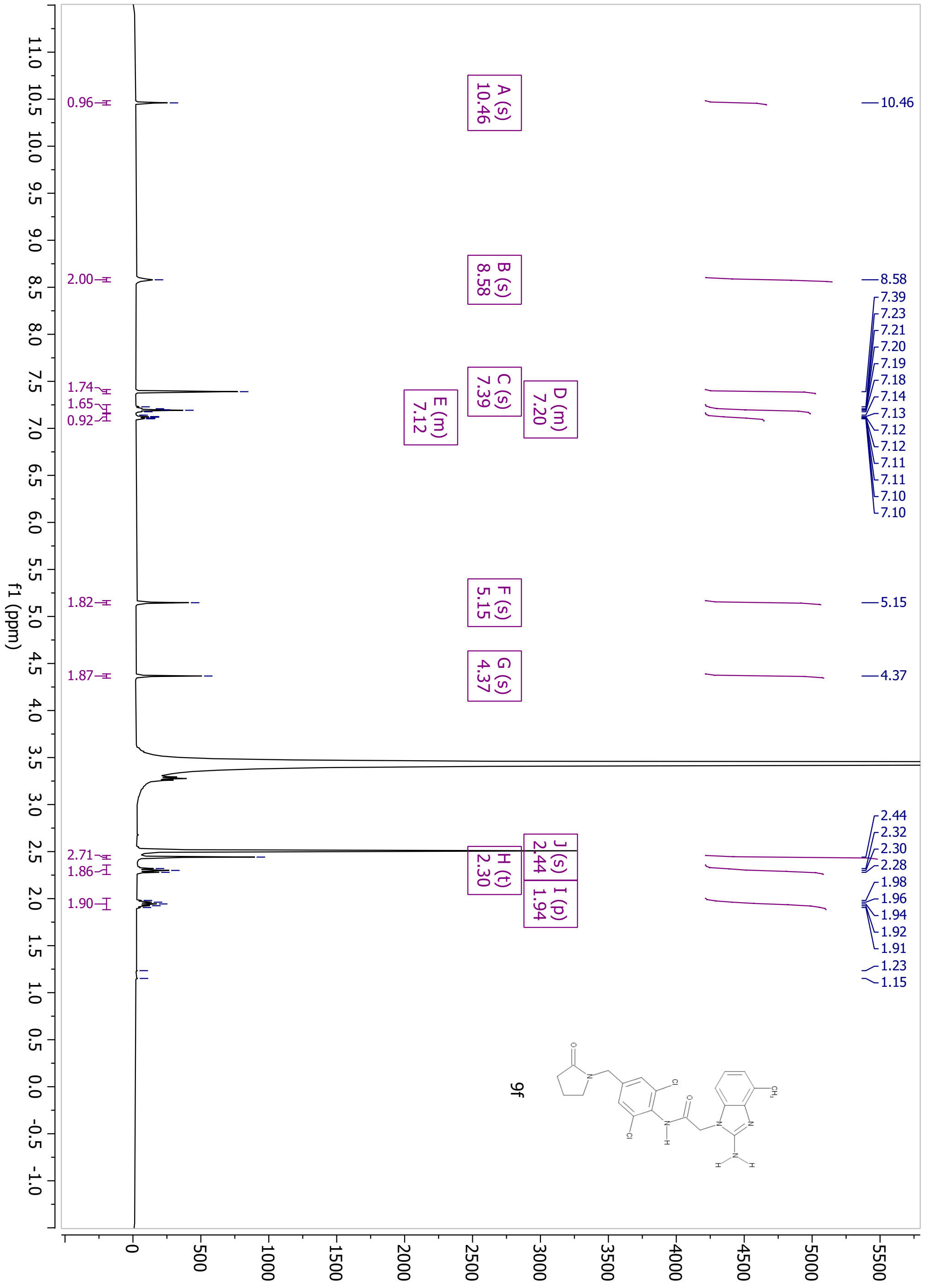
f1 (ppm)
A (s) 10.46
B (s) 8.58
C (s) 7.39
D (m) 7.20
E (m) 7.12
F (s) 5.15
G (s) 4.37
H (t) 2.30
I (p) 1.94
J (s) 2.44
0.96
2.00
1.74
1.65
0.92
1.82
1.87
2.71
1.86
1.90
10.46
8.58
7.39
7.23
7.21
7.20
7.19
7.18
7.14
7.13
7.12
7.12
7.11
7.11
7.10
7.10
5.15
4.37
2.44
2.32
2.30
2.28
1.98
1.96
1.94
1.92
1.91
1.23
1.15
9f

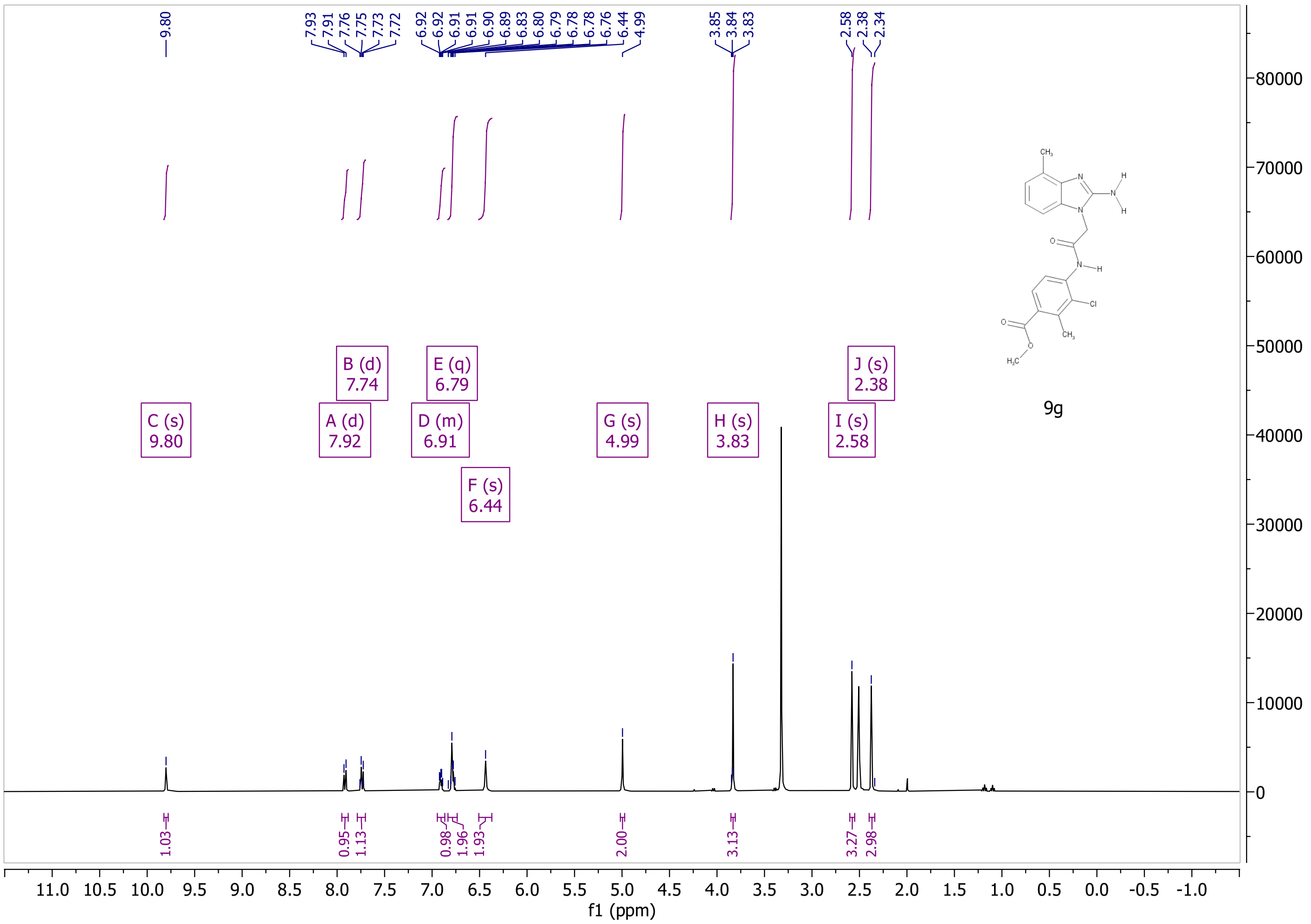
9.80
7.93 7.91 7.76 7.75 7.73 7.72
6.92 6.92 6.91 6.91 6.90 6.89 6.83 6.80 6.79 6.78 6.78 6.76 6.44 4.99
3.85 3.84 3.83
2.58 2.38 2.34
C (s) 9.80
A (d) 7.92
B (d) 7.74
D (m) 6.91
E (q) 6.79
F (s) 6.44
G (s) 4.99
H (s) 3.83
I (s) 2.58
J (s) 2.38
9g
1.03
0.95
1.13
0.98
1.96
1.93
2.00
3.13
3.27
2.98
11.0 10.5 10.0 9.5 9.0 8.5 8.0 7.5 7.0 6.5 6.0 5.5 5.0 4.5 4.0 3.5 3.0 2.5 2.0 1.5 1.0 0.5 0.0 -0.5 -1.0
f1 (ppm)
80000 70000 60000 50000 40000 30000 20000 10000 0

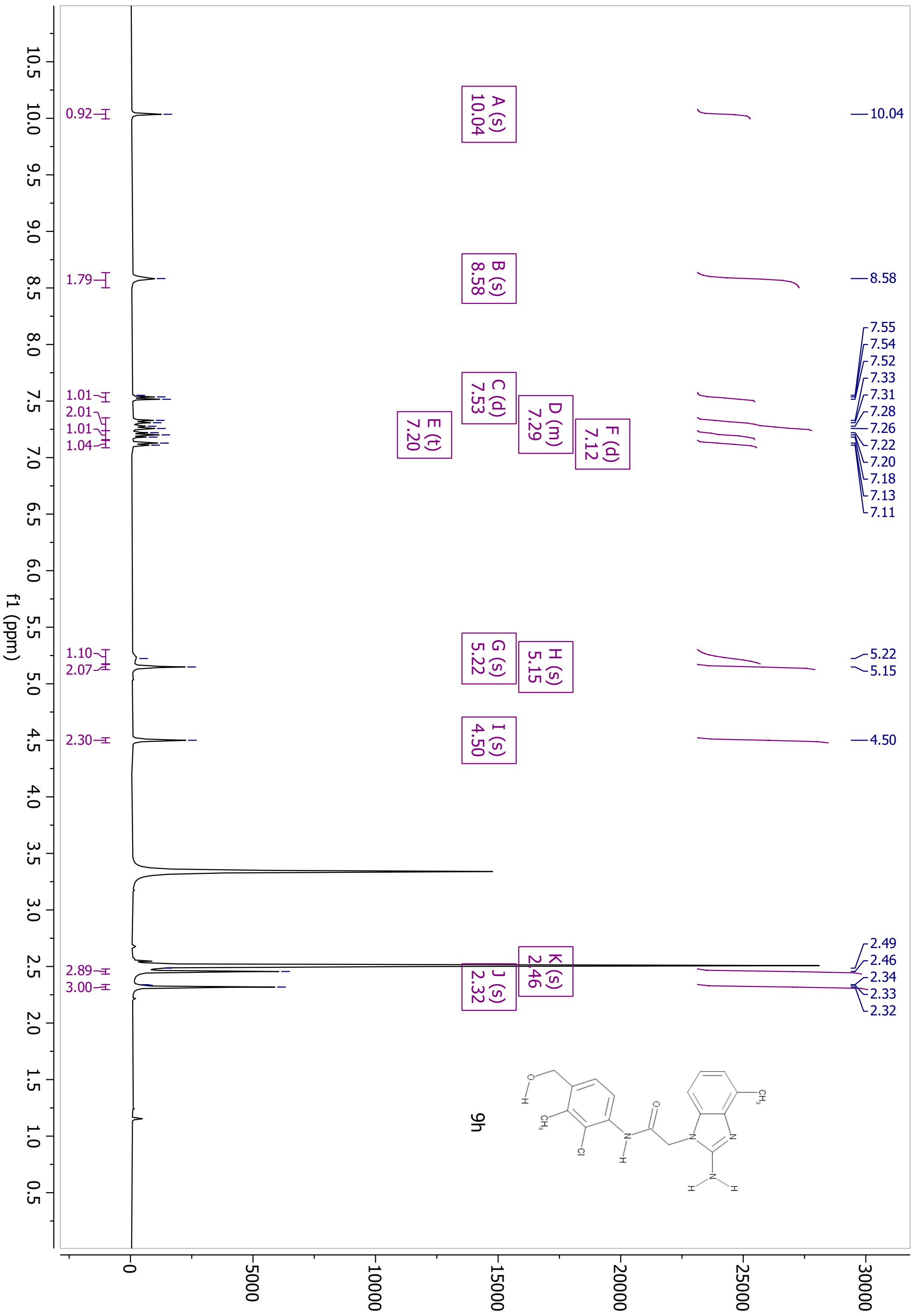

A (s) 10.04
B (s) 8.58
C (d) 7.53
D (m) 7.29
E (t) 7.20
F (d) 7.12
G (s) 5.22
H (s) 5.15
I (s) 4.50
J (s) 2.32
K (s) 2.46
10.04
8.58
7.55
7.54
7.52
7.33
7.31
7.28
7.26
7.22
7.20
7.18
7.13
7.11
5.22
5.15
4.50
2.49
2.46
2.34
2.33
2.32
0.92
1.79
1.01
2.01
1.01
1.04
1.10
2.07
2.30
2.89
3.00
f1 (ppm)
9h

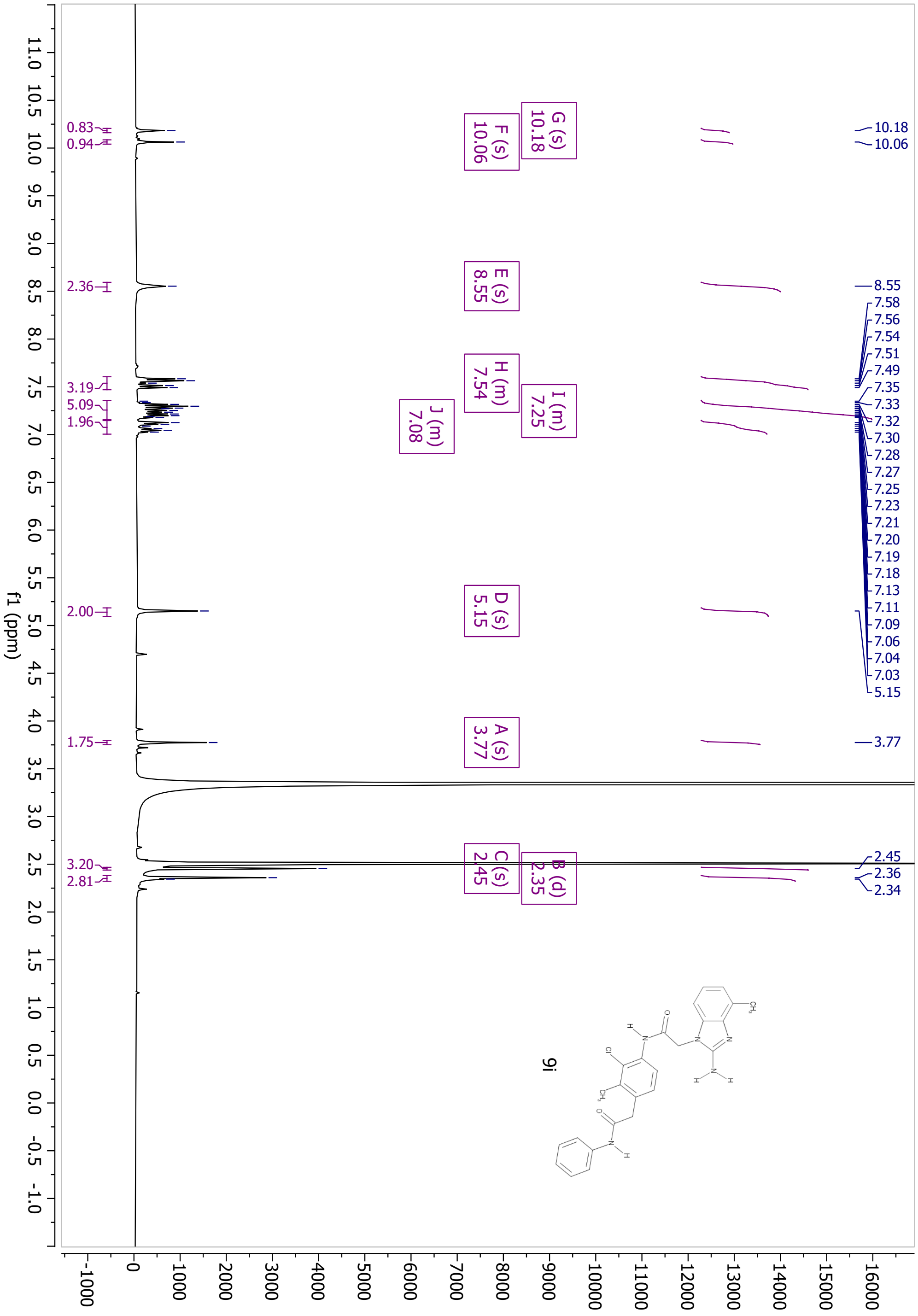

G (s)
10.18
F (s)
10.06
E (s)
8.55
H (m)
7.54
I (m)
7.25
J (m)
7.08
D (s)
5.15
A (s)
3.77
C (s)
2.45
B (d)
2.35
0.83
0.94
2.36
3.19
5.09
1.96
2.00
1.75
3.20
2.81
10.18
10.06
8.55
7.58
7.56
7.54
7.51
7.49
7.35
7.33
7.32
7.30
7.28
7.27
7.25
7.23
7.21
7.20
7.19
7.18
7.13
7.11
7.09
7.06
7.04
7.03
5.15
3.77
2.45
2.36
2.34
f1 (ppm)
9i

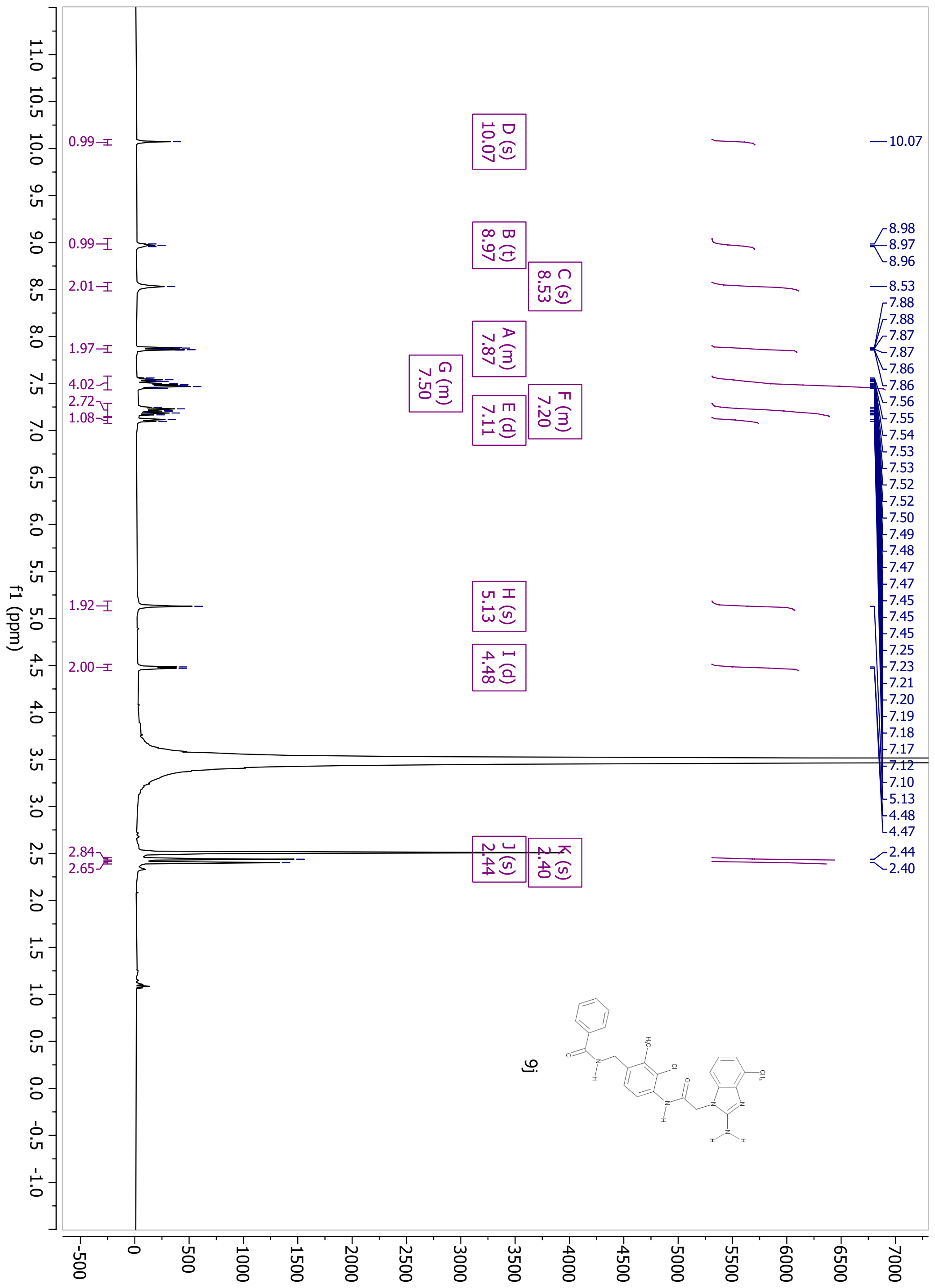
f1 (ppm)
9j
D (s) 10.07
B (t) 8.97
C (s) 8.53
A (m) 7.87
G (m) 7.50
F (m) 7.20
E (d) 7.11
H (s) 5.13
I (d) 4.48
J (s) 2.44
K (s) 2.40
0.99
0.99
2.01
1.97
4.02
2.72
1.08
1.92
2.00
2.84
2.65
10.07
8.98
8.97
8.96
8.53
7.88
7.88
7.87
7.87
7.86
7.86
7.56
7.55
7.54
7.53
7.53
7.52
7.52
7.50
7.49
7.48
7.47
7.47
7.45
7.45
7.45
7.25
7.23
7.21
7.20
7.19
7.18
7.17
7.12
7.10
5.13
4.48
4.47
2.44
2.40

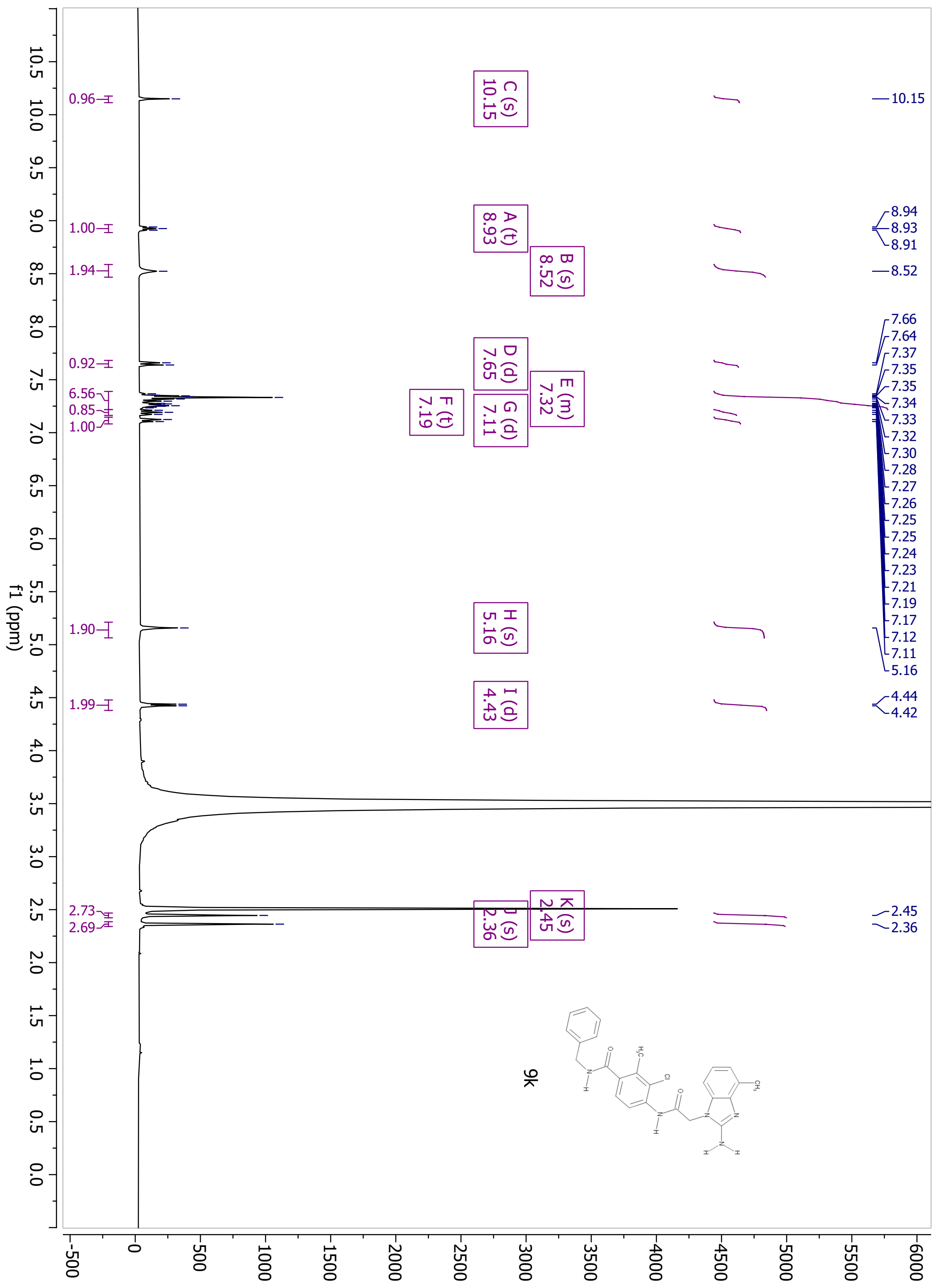

C (s)
10.15
A (t)
8.93
B (s)
8.52
D (d)
7.65
E (m)
7.32
G (d)
7.11
F (t)
7.19
H (s)
5.16
I (d)
4.43
K (s)
2.45
J (s)
2.36
9k
f1 (ppm)
0.96
1.00
1.94
0.92
6.56
0.85
1.00
1.90
1.99
2.73
2.69
10.15
8.94
8.93
8.91
8.52
7.66
7.64
7.37
7.35
7.35
7.34
7.33
7.32
7.30
7.28
7.27
7.26
7.25
7.25
7.24
7.23
7.21
7.19
7.17
7.12
7.11
5.16
4.44
4.42
2.45
2.36

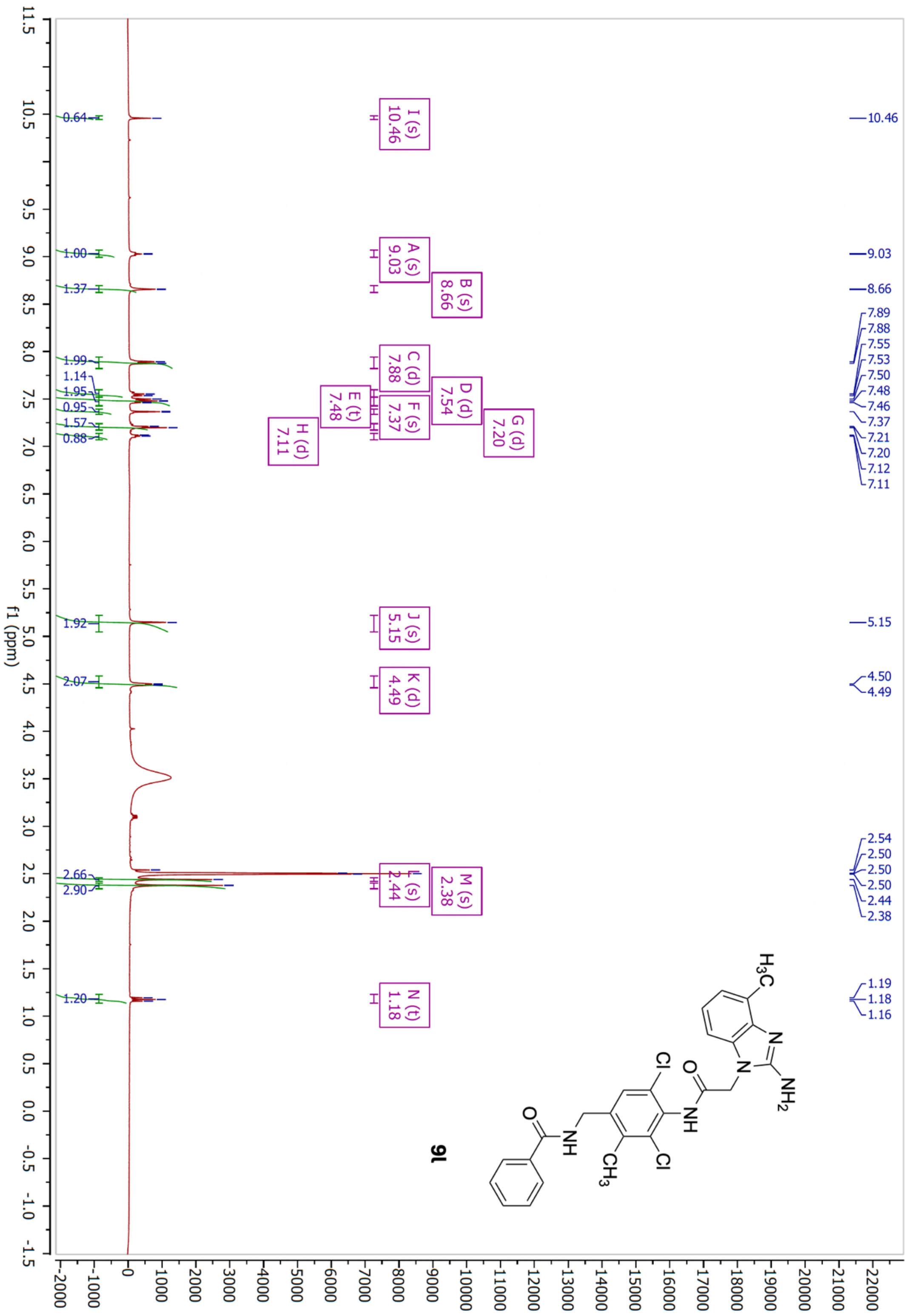

9l
f1 (ppm)
I (s) 10.46
A (s) 9.03
B (s) 8.66
C (d) 7.88
D (d) 7.54
E (t) 7.48
F (s) 7.37
G (d) 7.20
H (d) 7.11
J (s) 5.15
K (d) 4.49
L (s) 2.44
M (s) 2.38
N (t) 1.18

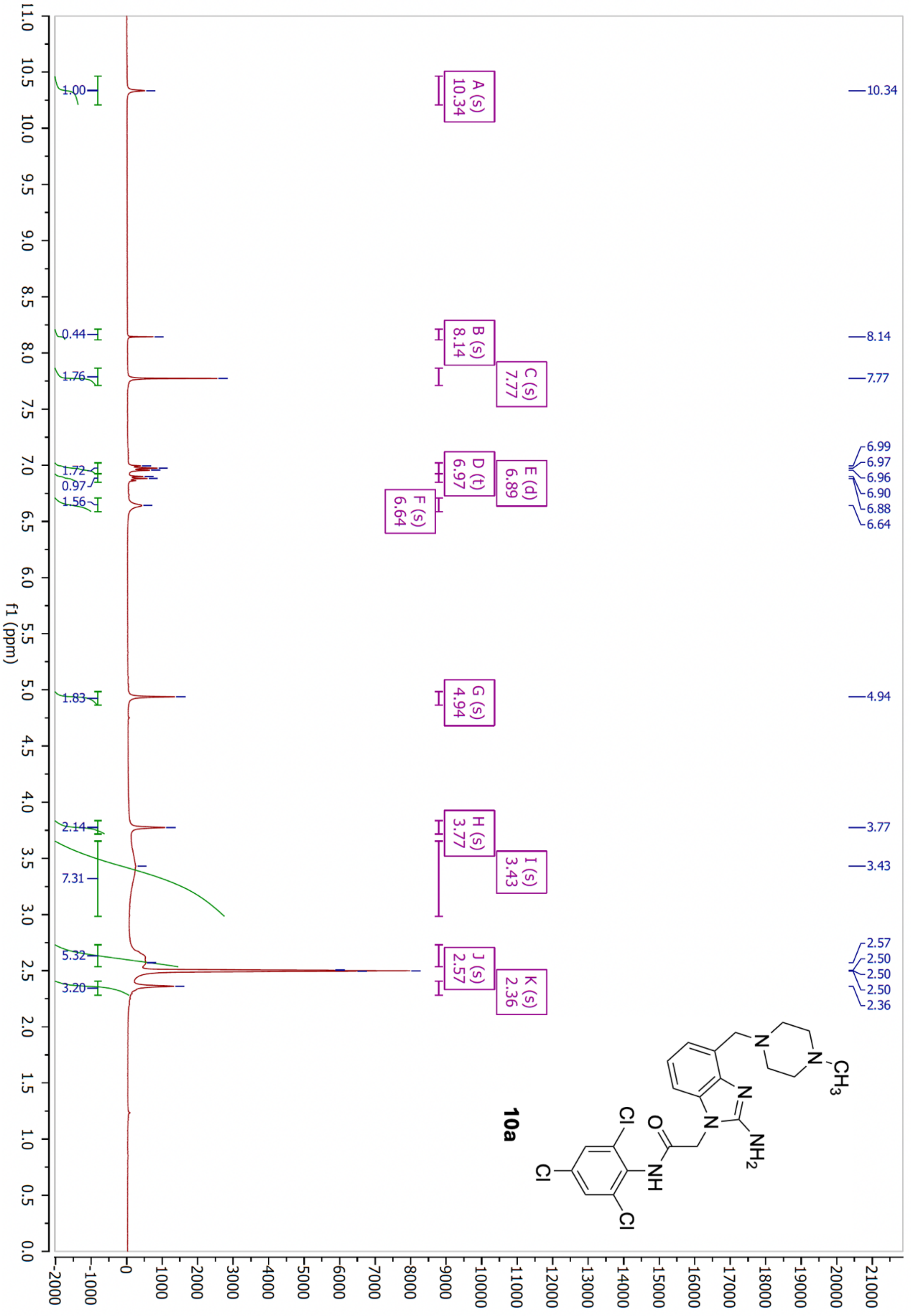
10.34
8.14
7.77
6.99
6.97
6.96
6.90
6.88
6.64
4.94
3.77
3.43
2.57
2.50
2.50
2.50
2.36
A (s) 10.34
B (s) 8.14
C (s) 7.77
D (t) 6.97
E (d) 6.89
F (s) 6.64
G (s) 4.94
H (s) 3.77
I (s) 3.43
J (s) 2.57
K (s) 2.36
1.00
0.44
1.76
1.72
0.97
1.56
1.83
2.14
7.31
5.32
3.20
f1 (ppm)
10a

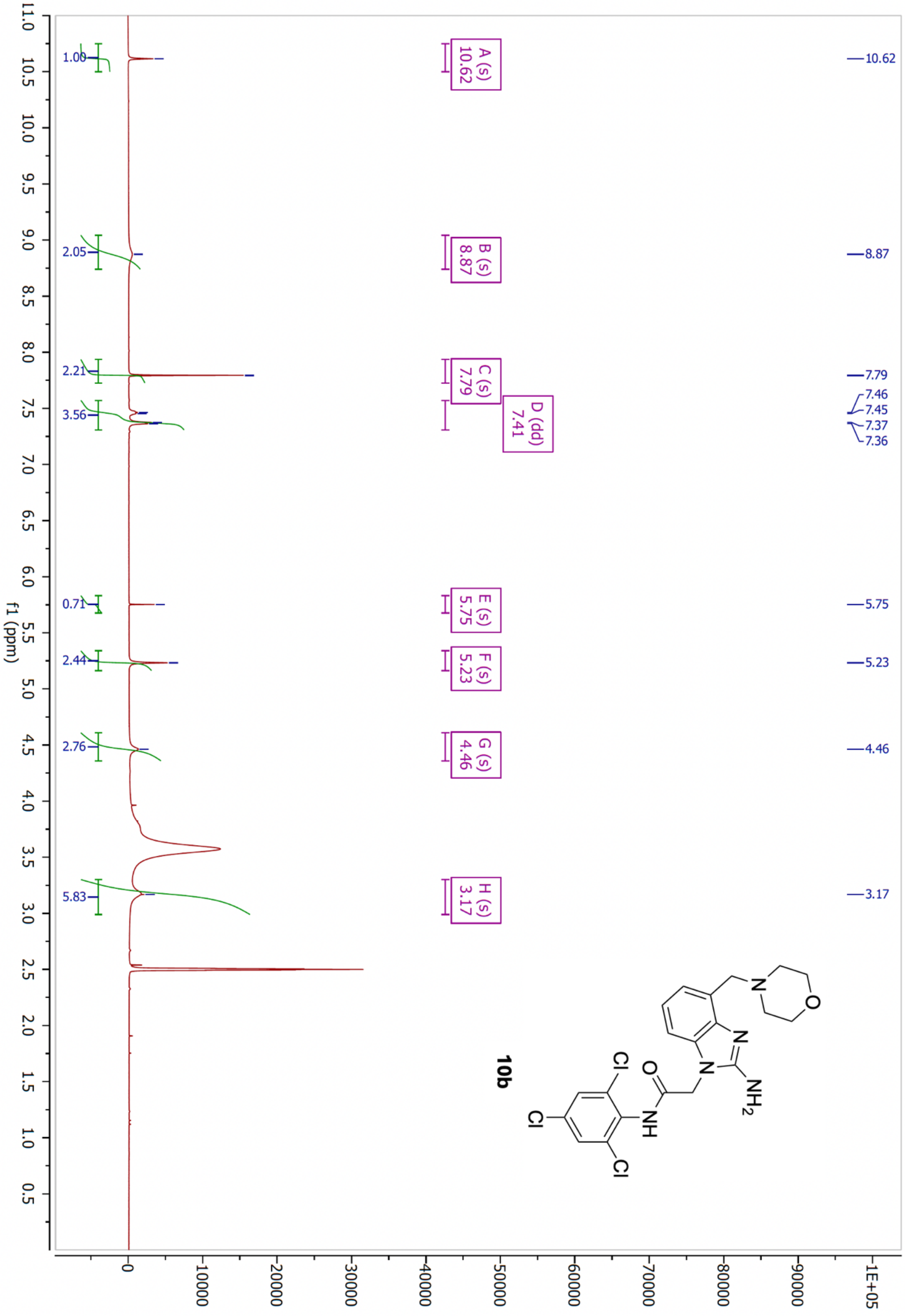

A (s) 10.62
B (s) 8.87
C (s) 7.79
D (dd) 7.41
E (s) 5.75
F (s) 5.23
G (s) 4.46
H (s) 3.17
10.62
8.87
7.79
7.46
7.45
7.37
7.36
5.75
5.23
4.46
3.17
1.00
2.05
2.21
3.56
0.71
2.44
2.76
5.83
f1 (ppm)
10b

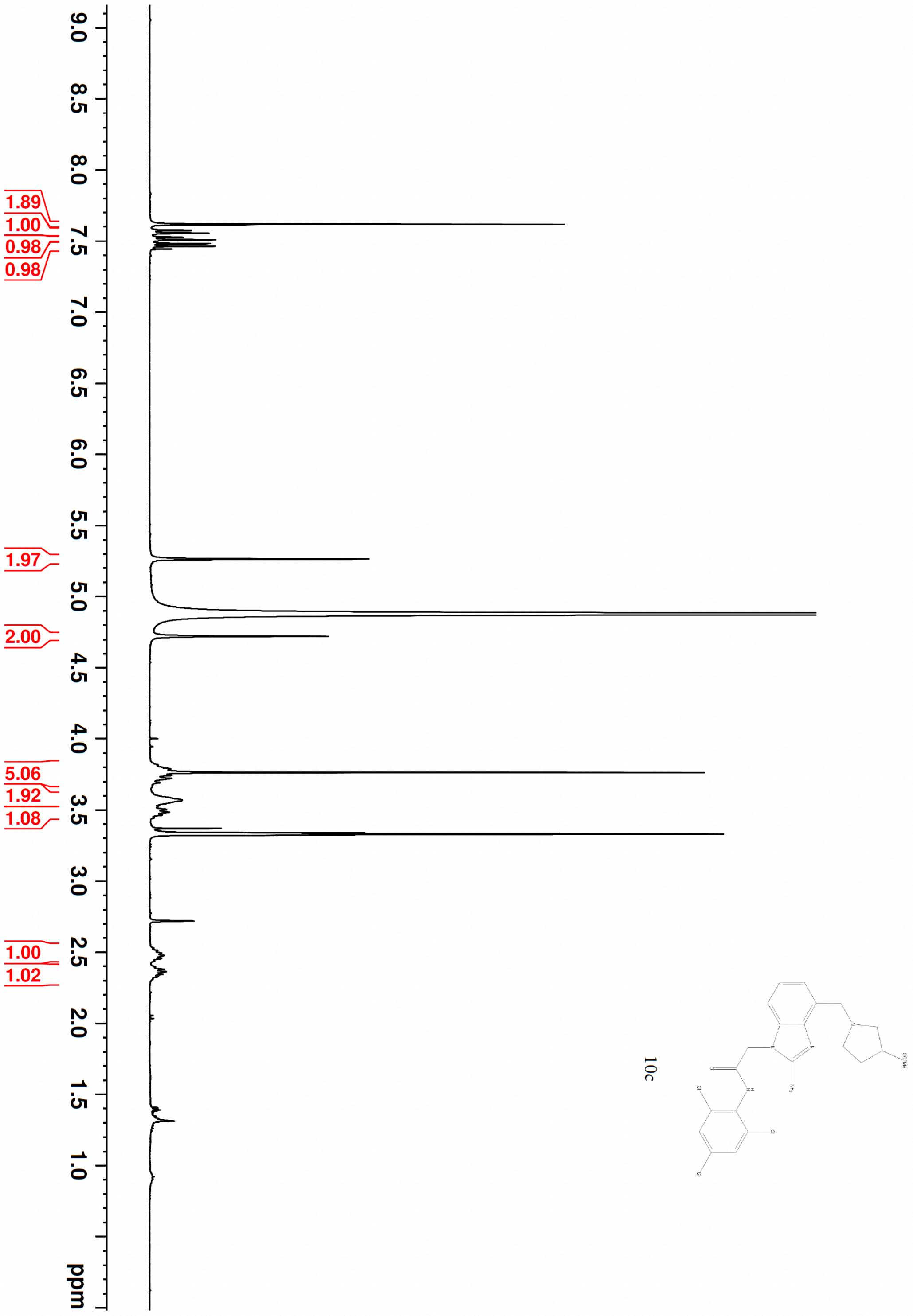
9.0
8.5
8.0
7.5
7.0
6.5
6.0
5.5
5.0
4.5
4.0
3.5
3.0
2.5
2.0
1.5
1.0
ppm
1.89
1.00
0.98
0.98
1.97
2.00
5.06
1.92
1.08
1.00
1.02
10c

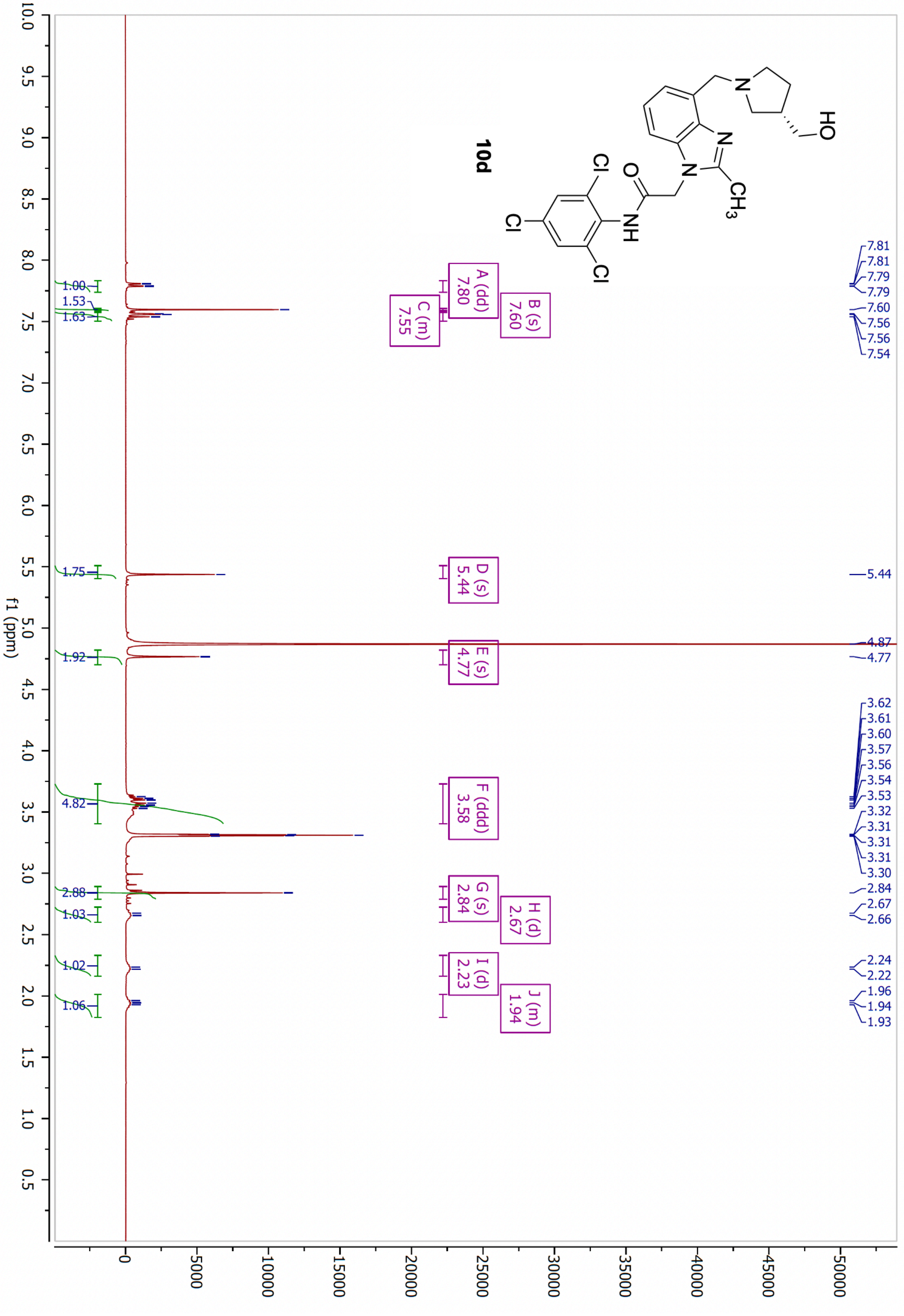
10d
A (dd) 7.80
B (s) 7.60
C (m) 7.55
D (s) 5.44
E (s) 4.77
F (ddd) 3.58
G (s) 2.84
H (d) 2.67
I (d) 2.23
J (m) 1.94
f1 (ppm)

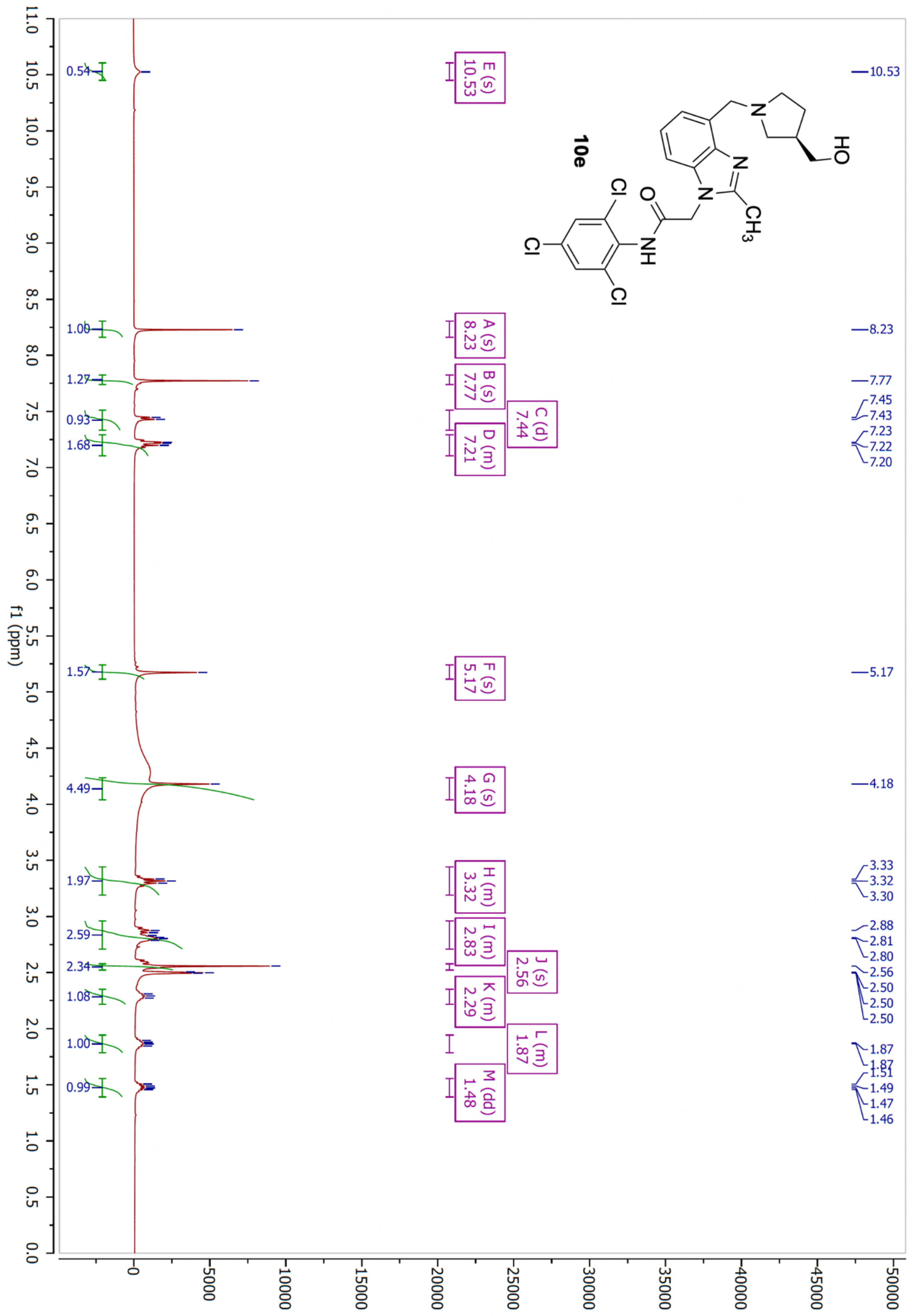
10e
E (s) 10.53
A (s) 8.23
B (s) 7.77
C (d) 7.44
D (m) 7.21
F (s) 5.17
G (s) 4.18
H (m) 3.32
I (m) 2.83
J (s) 2.56
K (m) 2.29
L (m) 1.87
M (dd) 1.48
f1 (ppm)

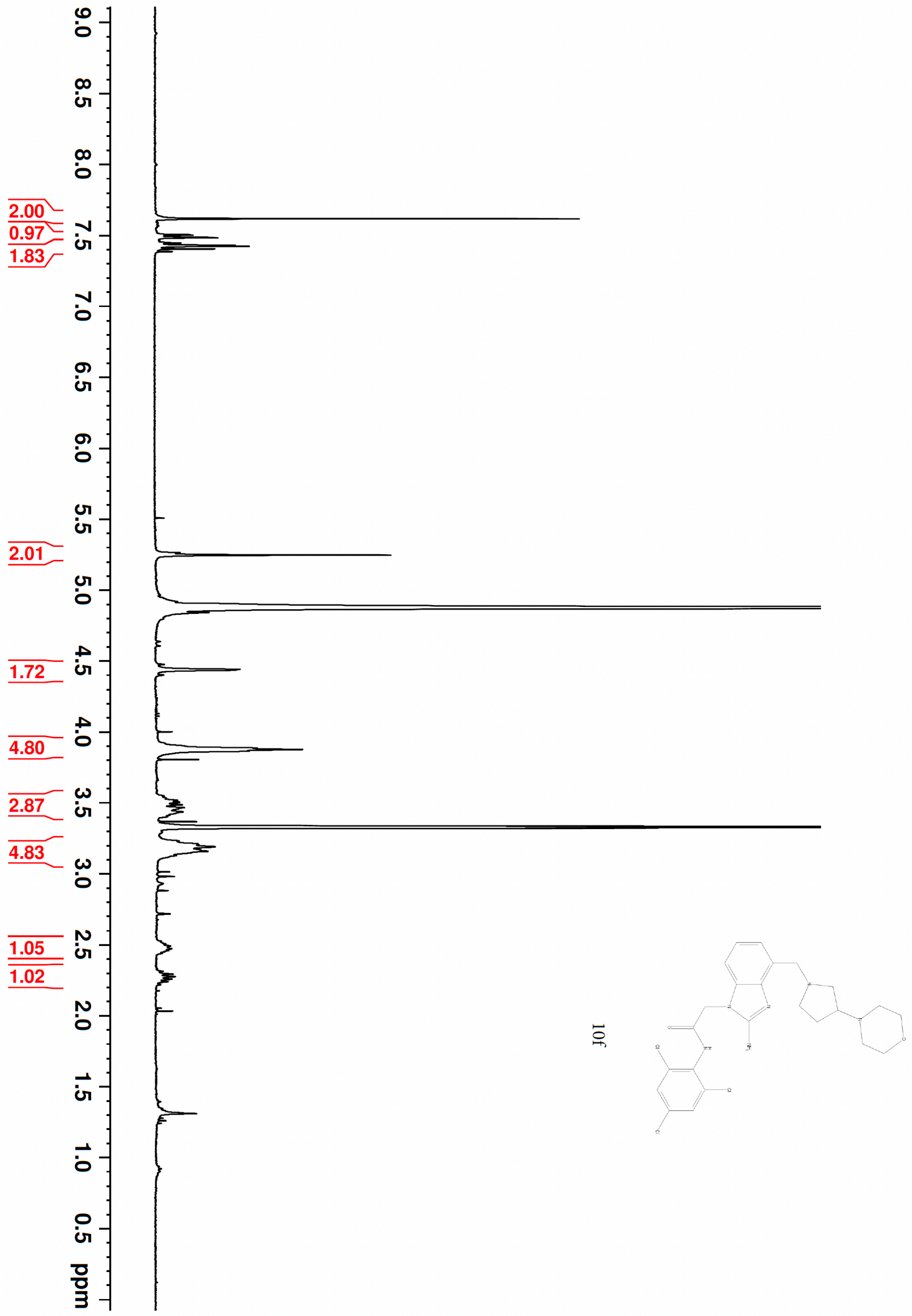

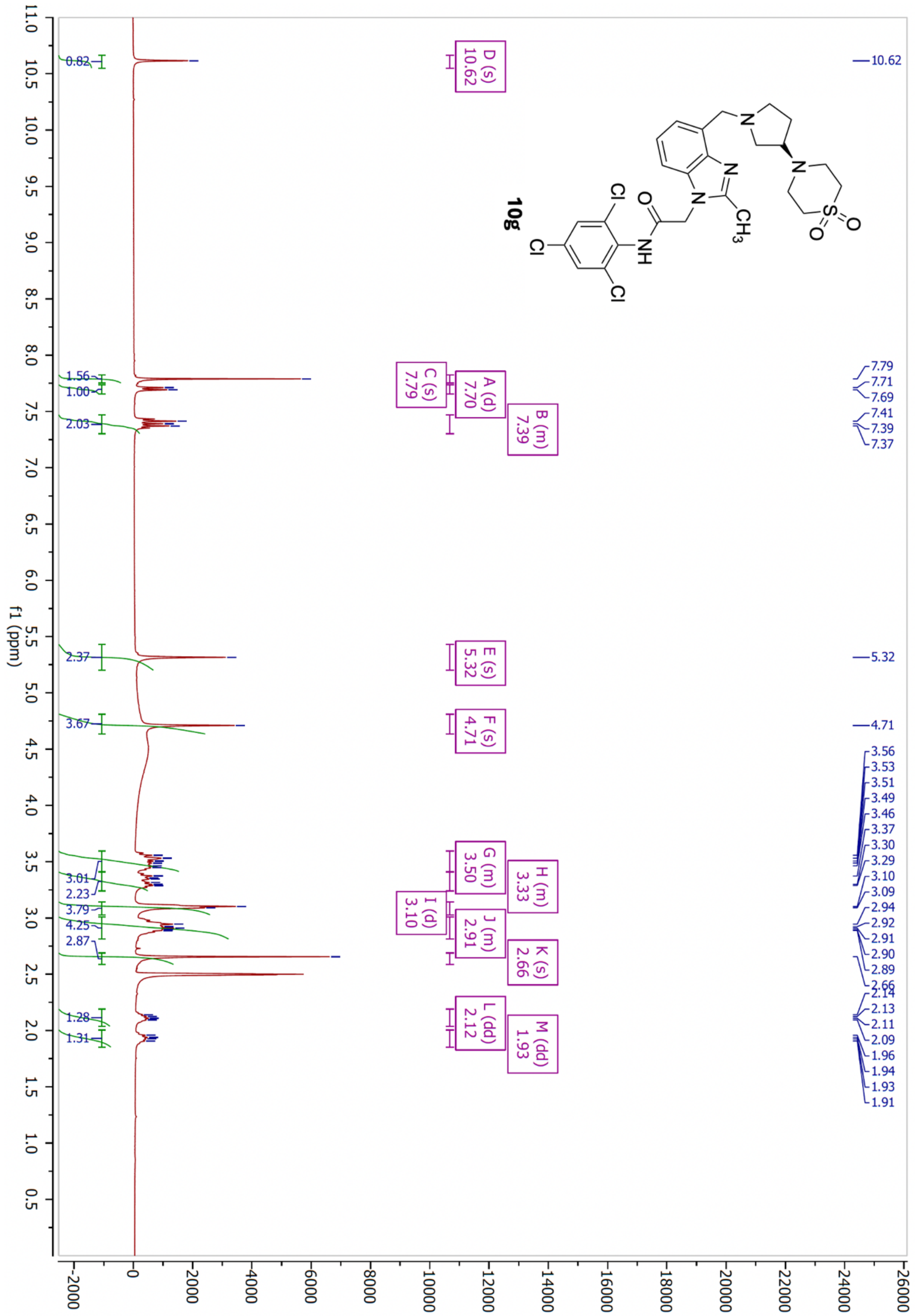
10g
D (s) 10.62
C (s) 7.79
A (d) 7.70
B (m) 7.39
E (s) 5.32
F (s) 4.71
G (m) 3.50
H (m) 3.33
I (d) 3.10
J (m) 2.91
K (s) 2.66
L (dd) 2.12
M (dd) 1.93
f1 (ppm)

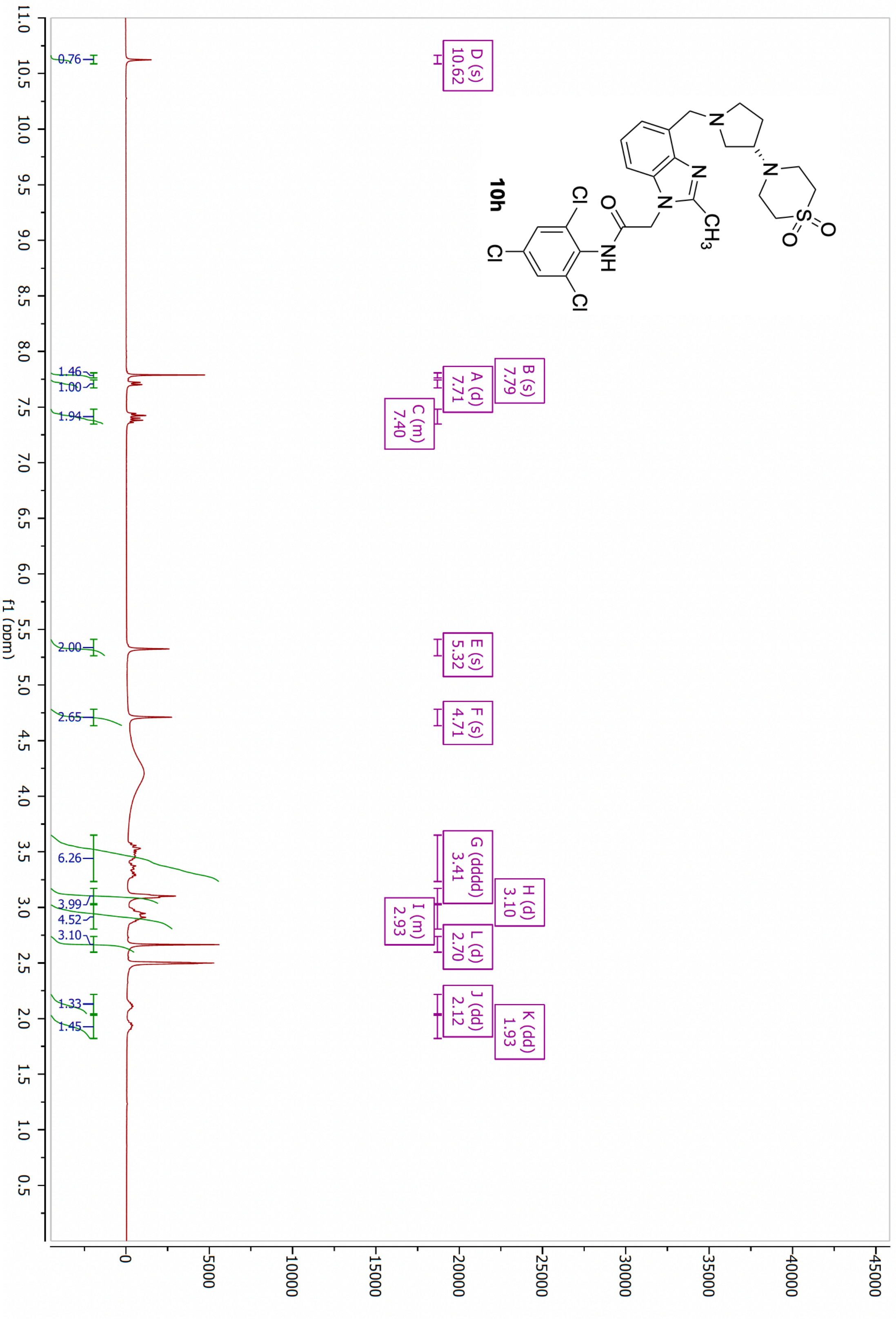
10h
D (s) 10.62
B (s) 7.79
A (d) 7.71
C (m) 7.40
E (s) 5.32
F (s) 4.71
G (dddd) 3.41
H (d) 3.10
I (m) 2.93
L (d) 2.70
J (dd) 2.12
K (dd) 1.93
f1 (ppm)

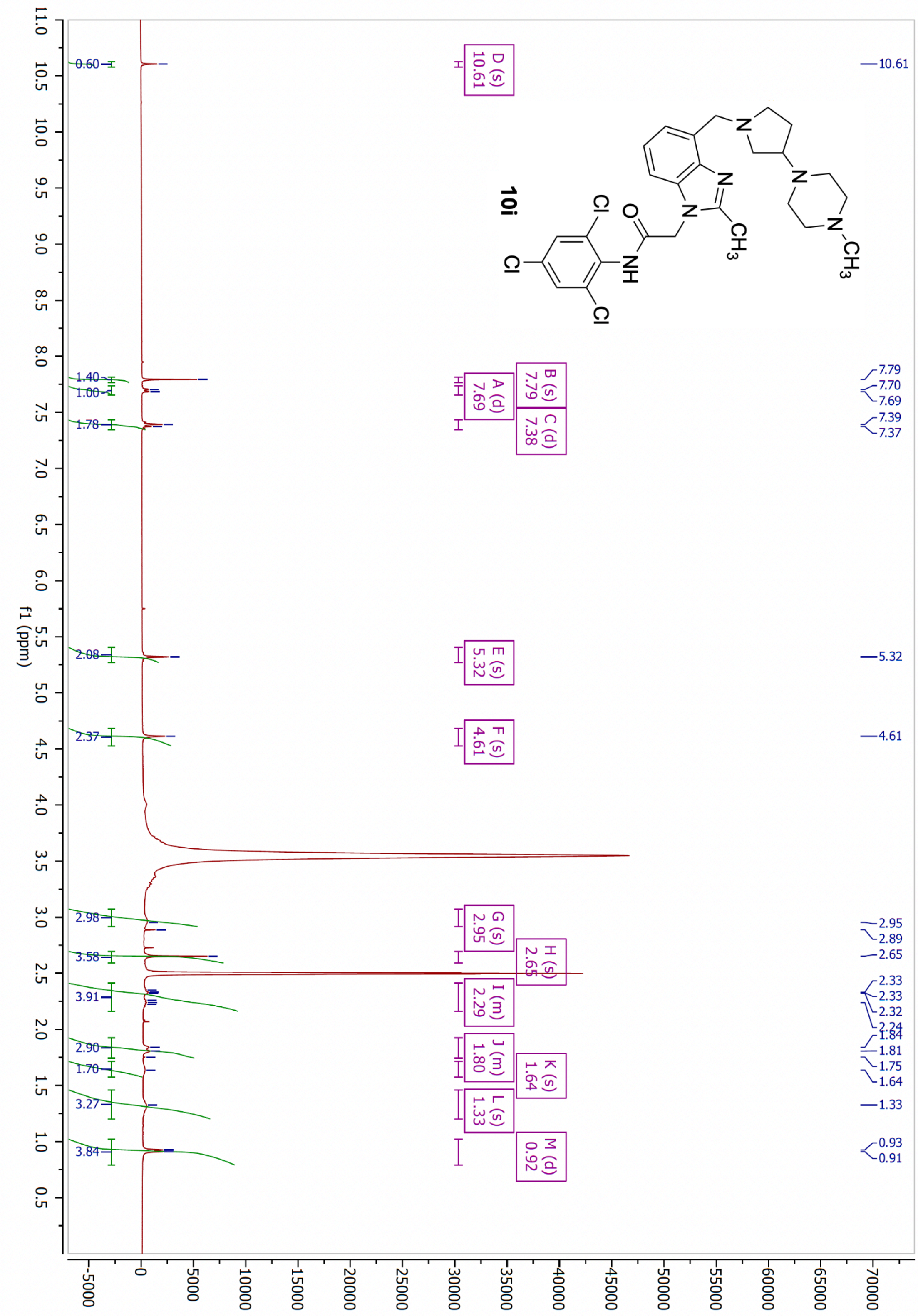
10i
D (s) 10.61
B (s) 7.79
A (d) 7.69
C (d) 7.38
E (s) 5.32
F (s) 4.61
G (s) 2.95
H (s) 2.65
I (m) 2.29
J (m) 1.80
K (s) 1.64
L (s) 1.33
M (d) 0.92
10.61
7.79
7.70
7.69
7.39
7.37
5.32
4.61
2.95
2.89
2.65
2.33
2.33
2.32
2.24
1.84
1.81
1.75
1.64
1.33
0.93
0.91
0.60
1.40
1.00
1.78
2.08
2.37
2.98
3.58
3.91
2.90
1.70
3.27
3.84
f1 (ppm)

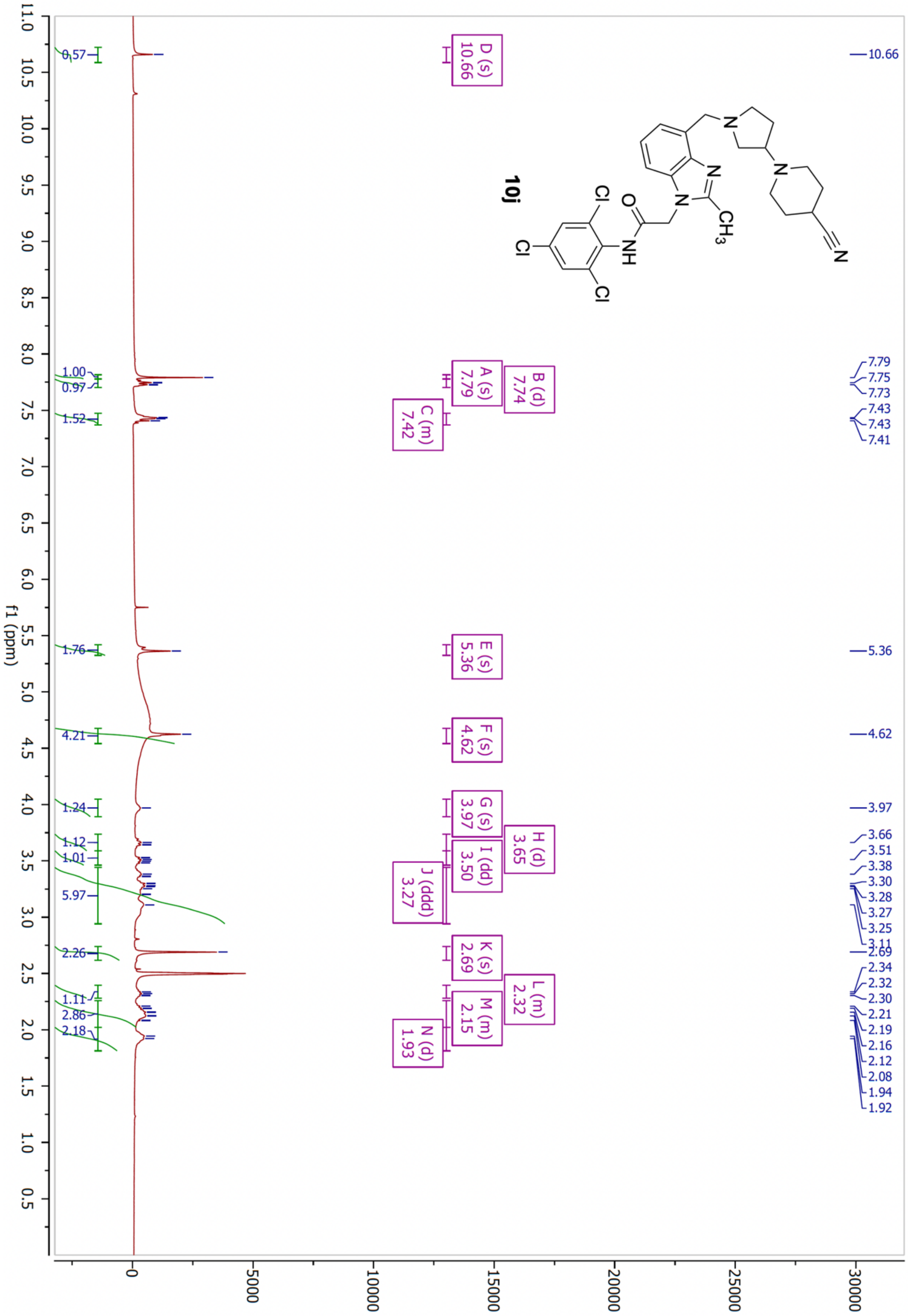
10j
f1 (ppm)
D (s) 10.66
A (s) 7.79
B (d) 7.74
C (m) 7.42
E (s) 5.36
F (s) 4.62
G (s) 3.97
H (d) 3.65
I (dd) 3.50
J (ddd) 3.27
K (s) 2.69
L (m) 2.32
M (m) 2.15
N (d) 1.93

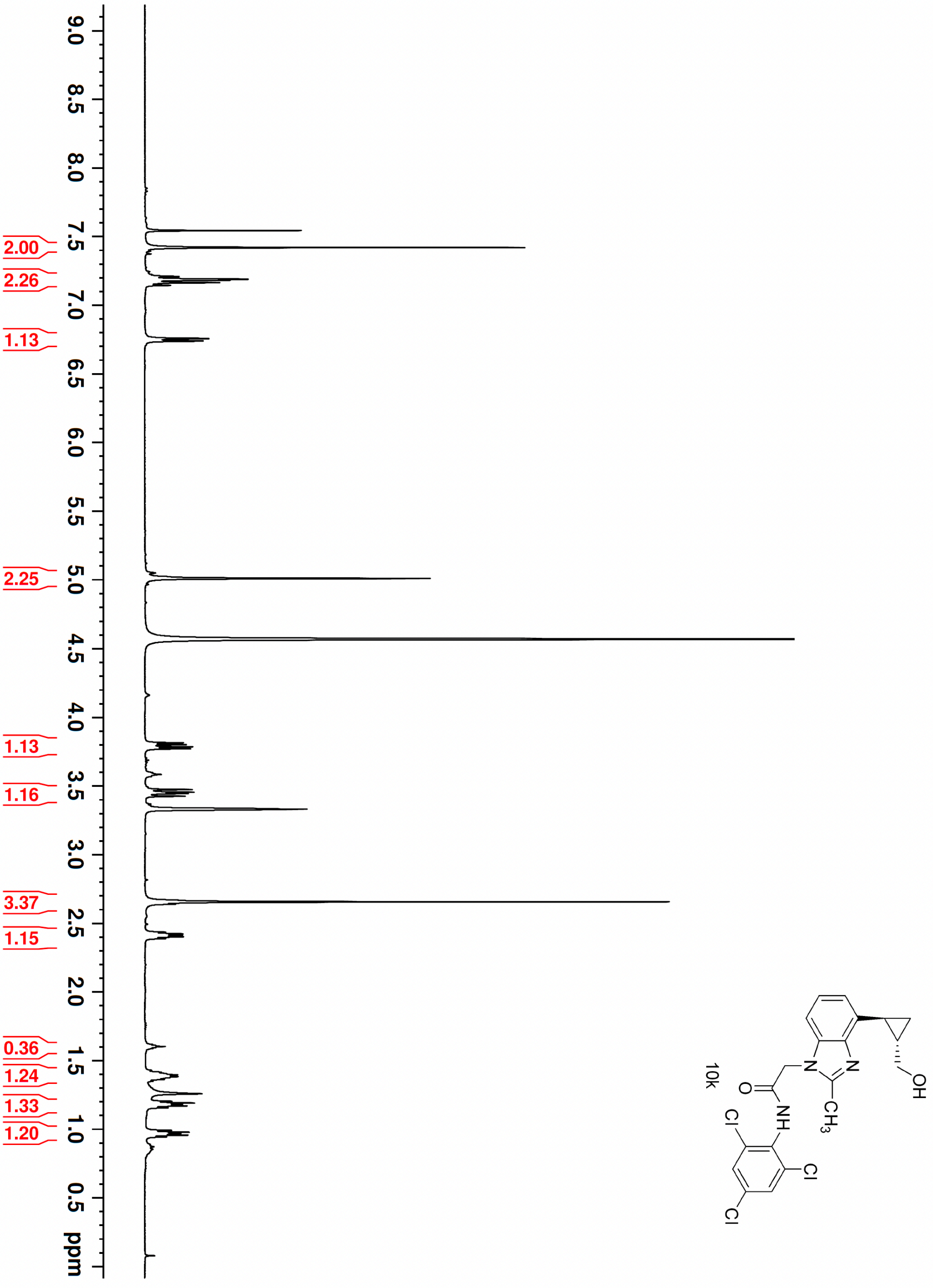

9.0
8.5
8.0
7.5
7.0
6.5
6.0
5.5
5.0
4.5
4.0
3.5
3.0
2.5
2.0
1.5
1.0
0.5
ppm
2.00
2.26
1.13
2.25
1.13
1.16
3.37
1.15
0.36
1.24
1.33
1.20
OH
N
N
CH3
O
NH
Cl
Cl
Cl

10k

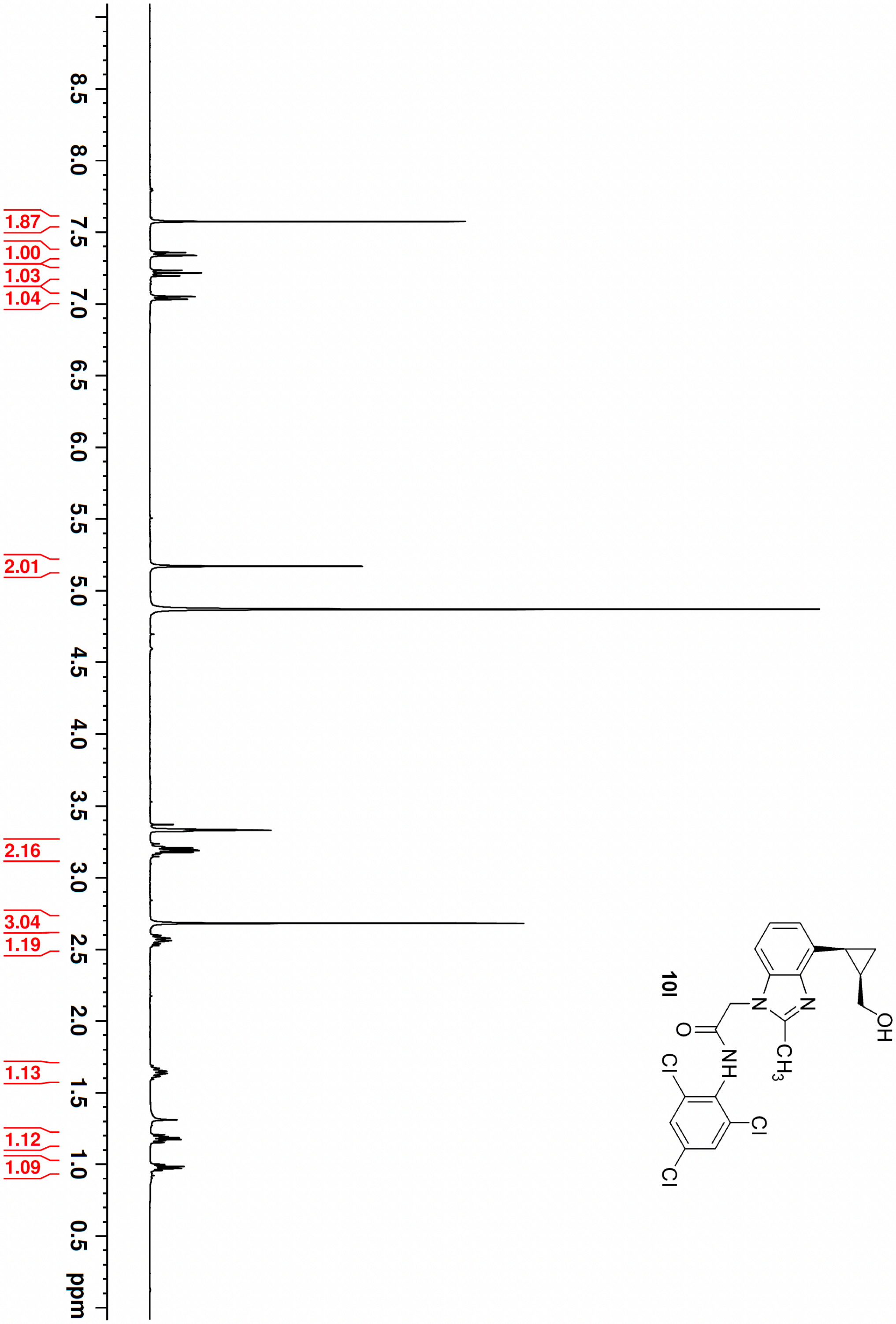

8.5
8.0
7.5
7.0
6.5
6.0
5.5
5.0
4.5
4.0
3.5
3.0
2.5
2.0
1.5
1.0
0.5
ppm
1.87
1.00
1.03
1.04
2.01
2.16
3.04
1.19
1.13
1.12
1.09
10l
O
NH
N
N
CH3
OH
Cl
Cl
Cl

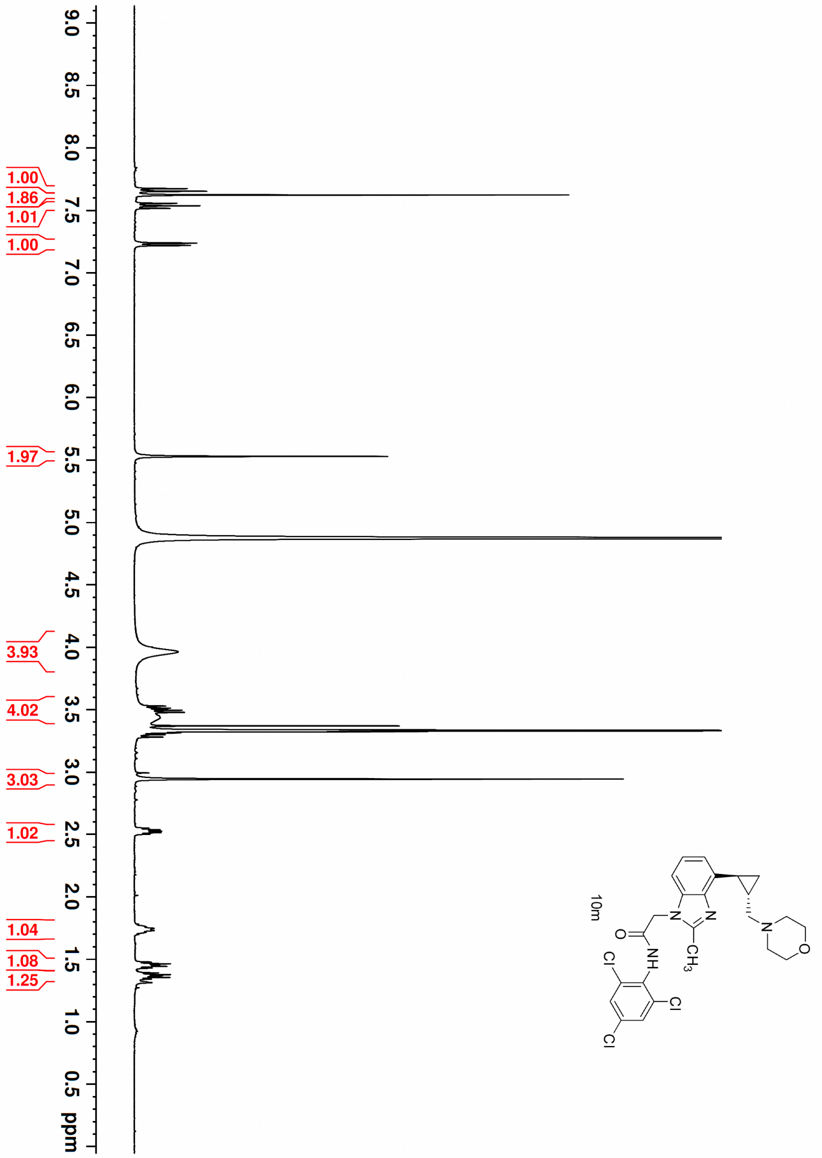
9.0
8.5
8.0
7.5
7.0
6.5
6.0
5.5
5.0
4.5
4.0
3.5
3.0
2.5
2.0
1.5
1.0
0.5
ppm
1.00
1.86
1.01
1.00
1.97
3.93
4.02
3.03
1.02
1.04
1.08
1.25
10m

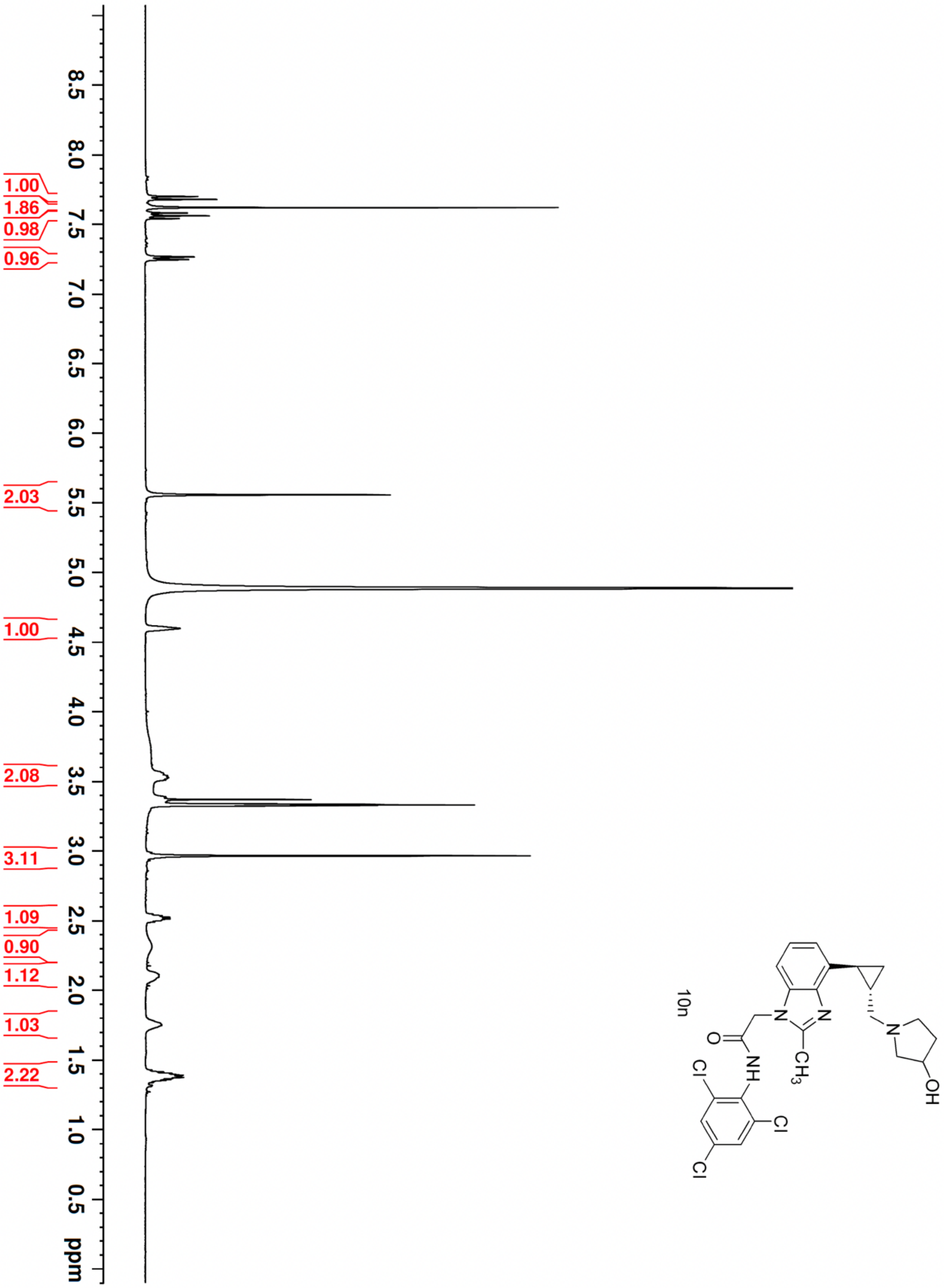

8.5
8.0
7.5
7.0
6.5
6.0
5.5
5.0
4.5
4.0
3.5
3.0
2.5
2.0
1.5
1.0
0.5
ppm
1.00
1.86
0.98
0.96
2.03
1.00
2.08
3.11
1.09
0.90
1.12
1.03
2.22
10n
N
N
CH3
O
NH
Cl
Cl
Cl
N
OH

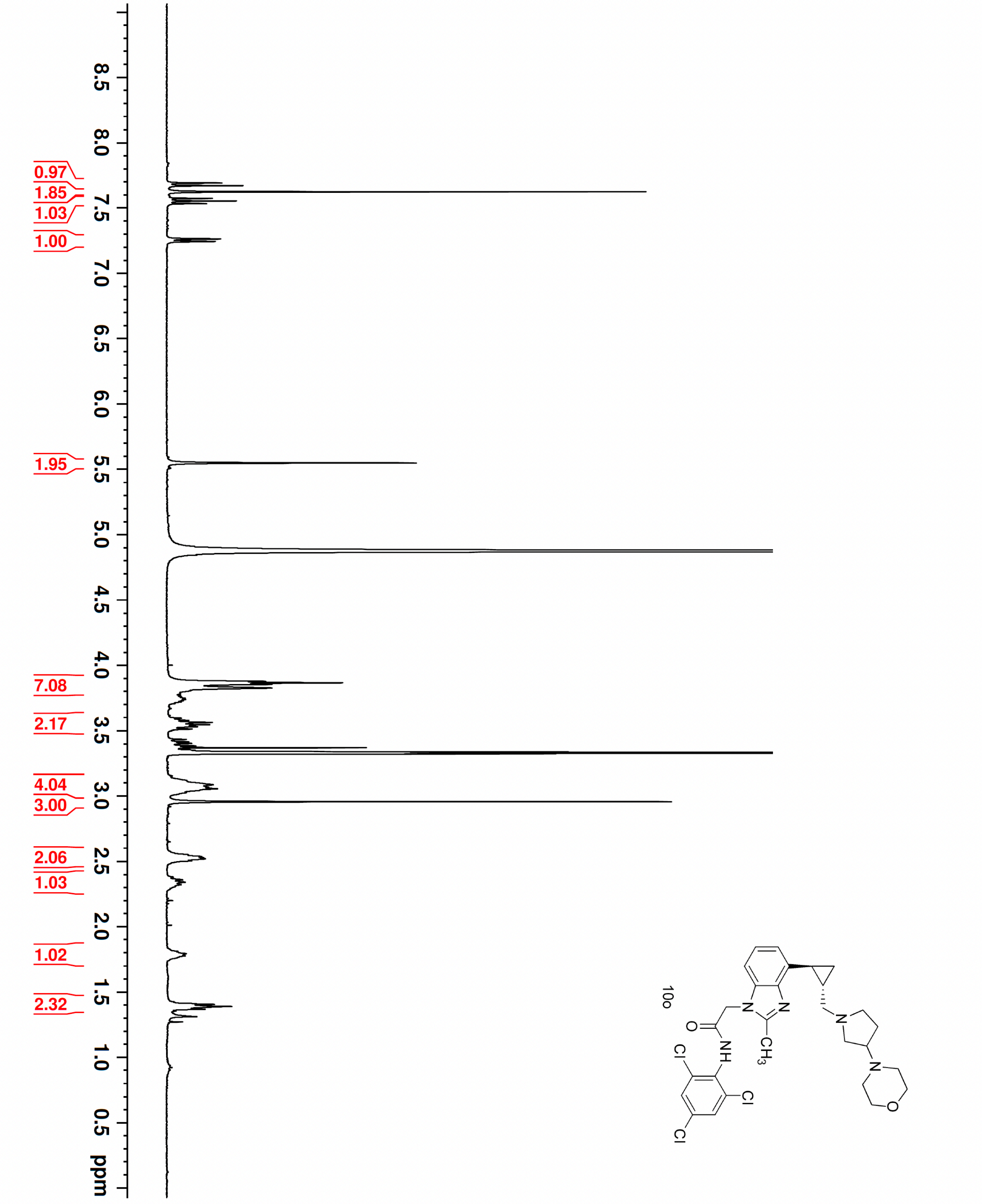
8.5
8.0
7.5
7.0
6.5
6.0
5.5
5.0
4.5
4.0
3.5
3.0
2.5
2.0
1.5
1.0
0.5
ppm
0.97
1.85
1.03
1.00
1.95
7.08
2.17
4.04
3.00
2.06
1.03
1.02
2.32
10o
N
N
CH3
O
NH
Cl
Cl
Cl
N
N
O

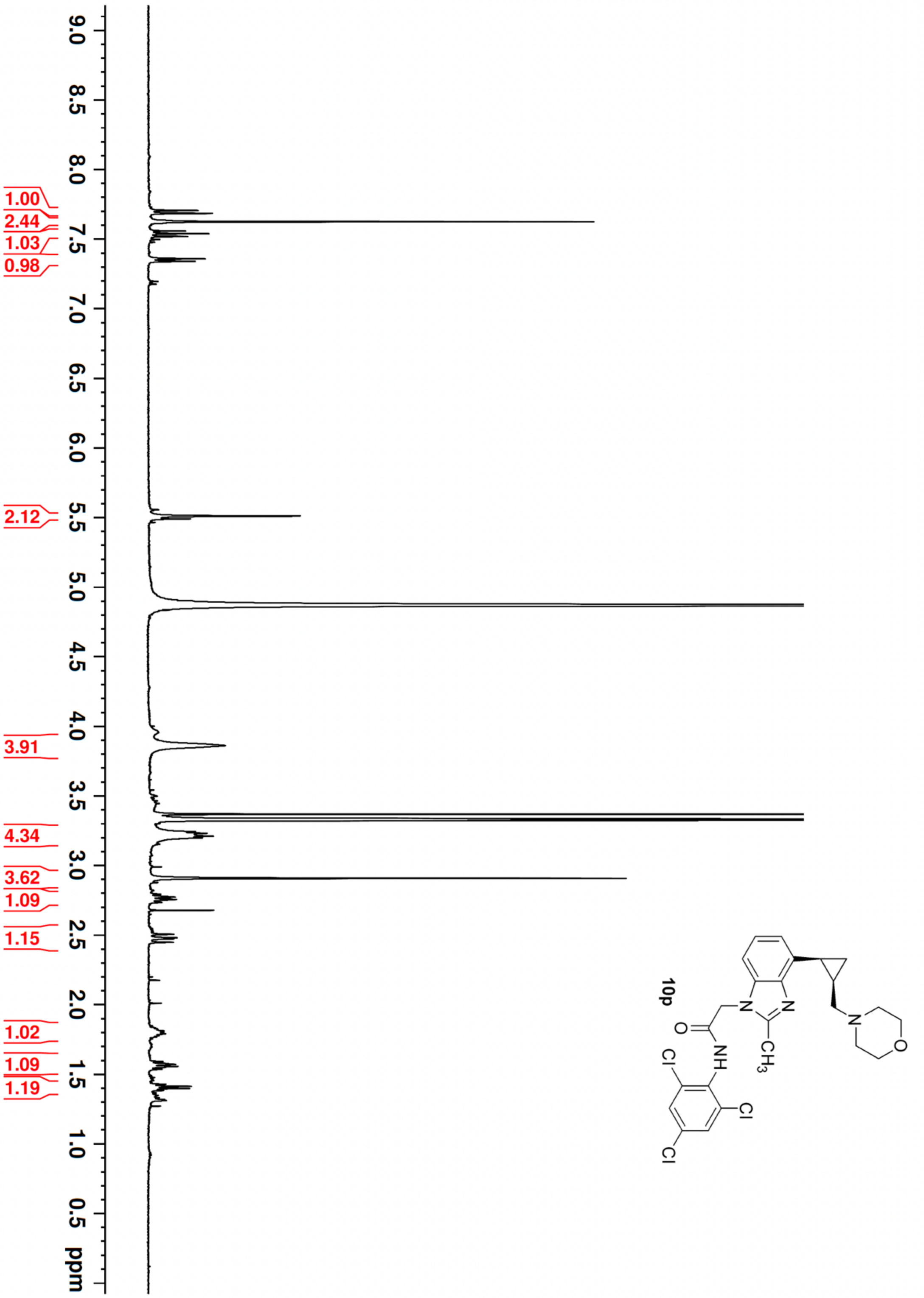
10p
ppm
1.00
2.44
1.03
0.98
2.12
3.91
4.34
3.62
1.09
1.15
1.02
1.09
1.19

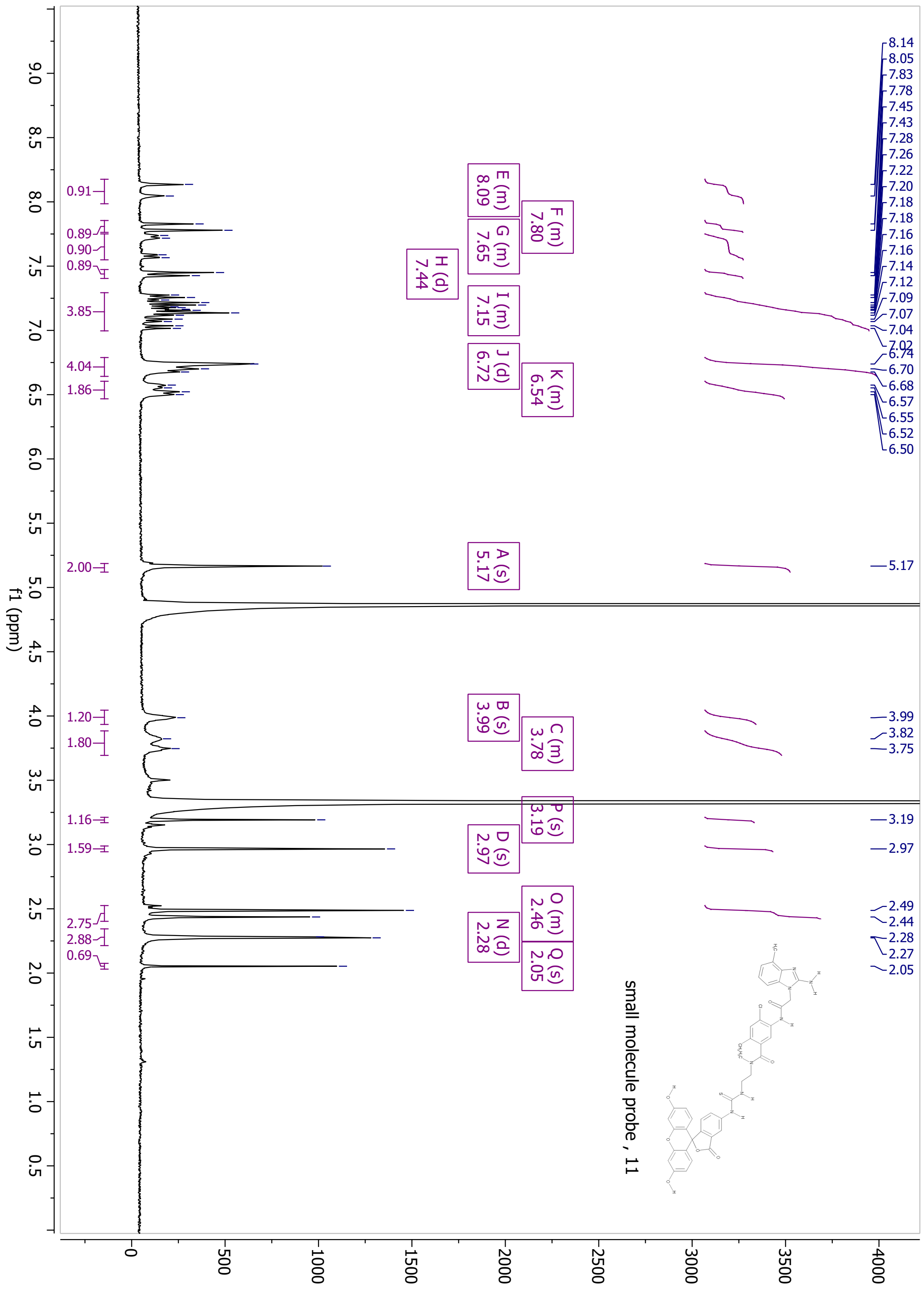
small molecule probe , 11
f1 (ppm)
A (s) 5.17
B (s) 3.99
C (m) 3.78
D (s) 2.97
E (m) 8.09
F (m) 7.80
G (m) 7.65
H (d) 7.44
I (m) 7.15
J (d) 6.72
K (m) 6.54
N (d) 2.28
O (m) 2.46
P (s) 3.19
Q (s) 2.05
8.14
8.05
7.83
7.78
7.45
7.43
7.28
7.26
7.22
7.20
7.18
7.18
7.16
7.16
7.14
7.12
7.09
7.07
7.04
7.02
6.74
6.70
6.68
6.57
6.55
6.52
6.50
5.17
3.99
3.82
3.75
3.19
2.97
2.49
2.44
2.28
2.27
2.05
0.91
0.89
0.90
0.89
3.85
4.04
1.86
2.00
1.20
1.80
1.16
1.59
2.75
2.88
0.69

LCMS data for selected compounds

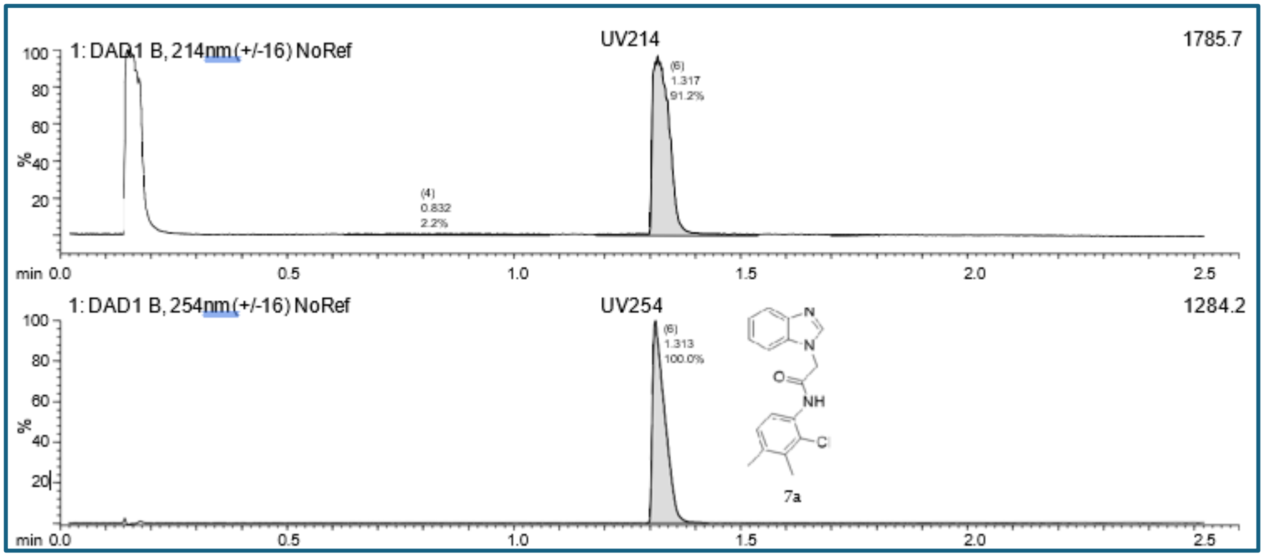

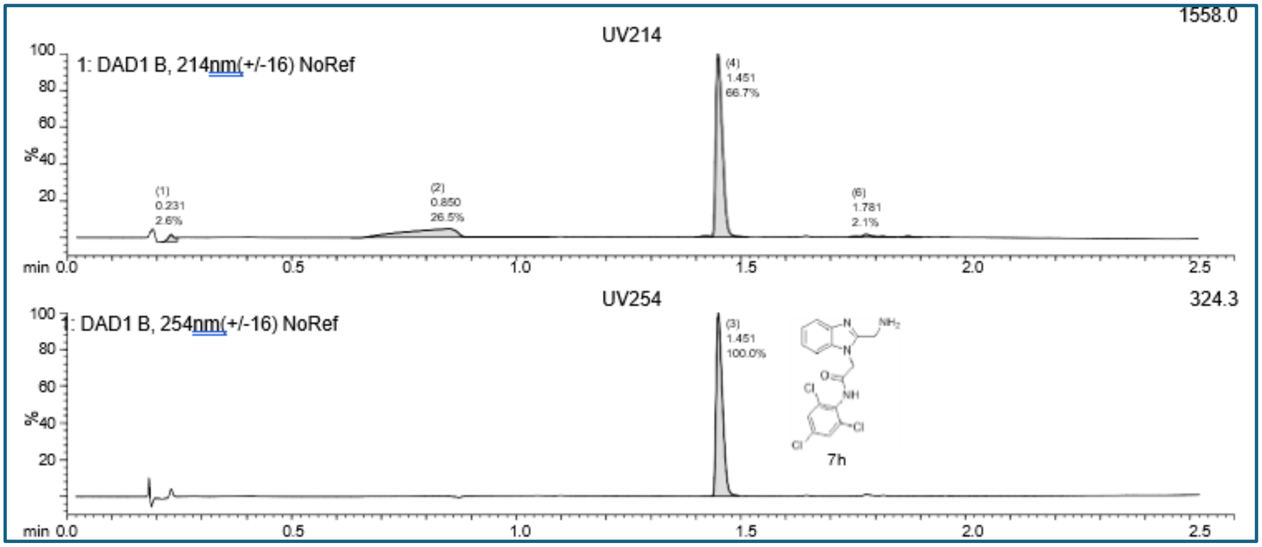

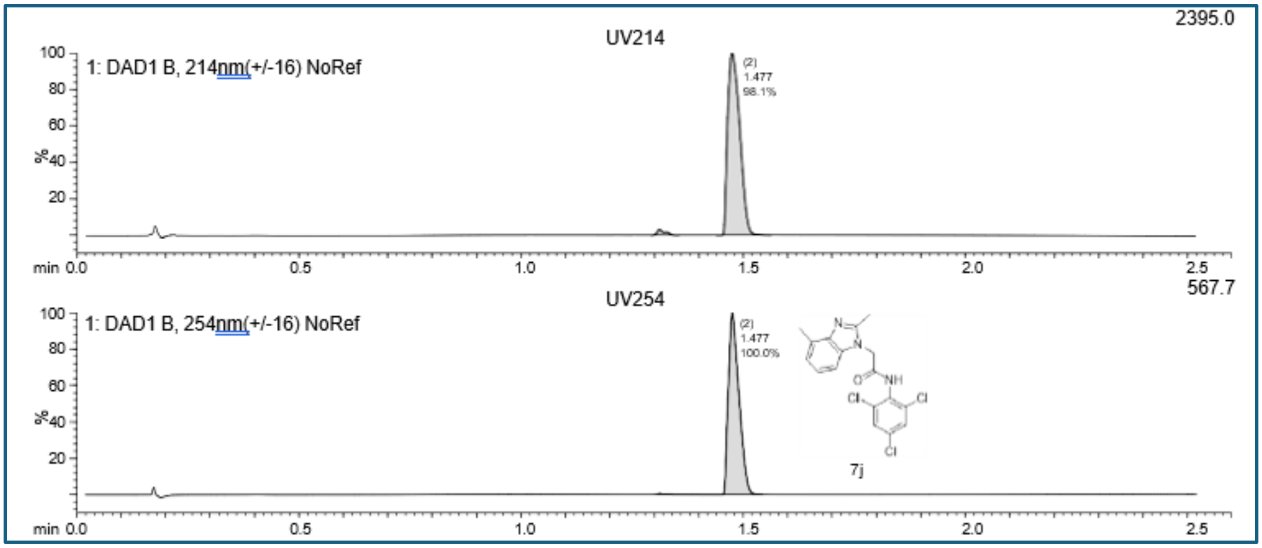
UV214
2395.0
1: DAD1 B, 214nm(+/-16) NoRef
(2)
1.477
98.1%
UV254
567.7
1: DAD1 B, 254nm(+/-16) NoRef
(2)
1.477
100.0%
7j
min
%

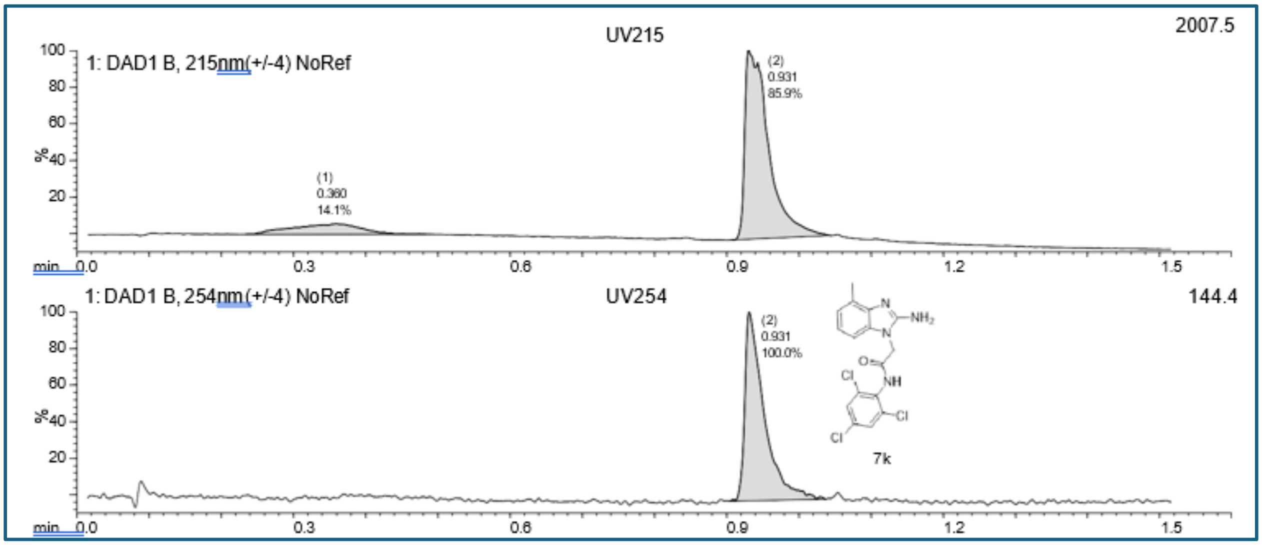
UV215
2007.5
1: DAD1 B, 215nm(+/-4) NoRef
(1)
0.360
14.1%
(2)
0.931
85.9%
UV254
144.4
1: DAD1 B, 254nm(+/-4) NoRef
(2)
0.931
100.0%
7k
min
%

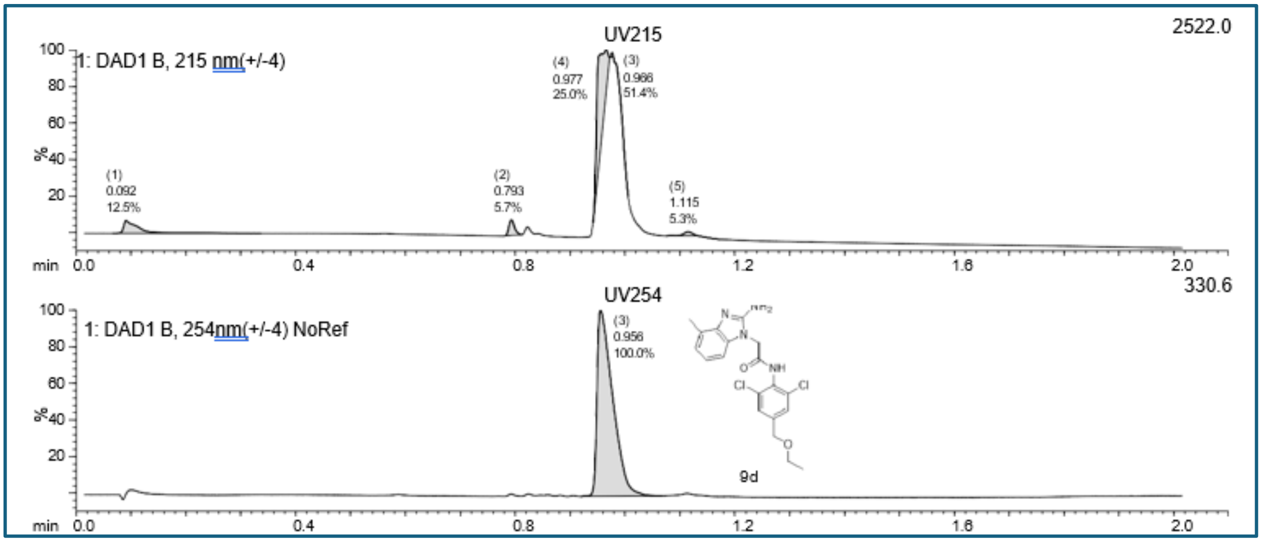
UV215
2522.0
1: DAD1 B, 215 nm(+/-4)
(1) 0.092 12.5%
(2) 0.793 5.7%
(4) 0.977 25.0%
(3) 0.966 51.4%
(5) 1.115 5.3%
UV254
330.6
1: DAD1 B, 254nm(+/-4) NoRef
(3) 0.956 100.0%
9d

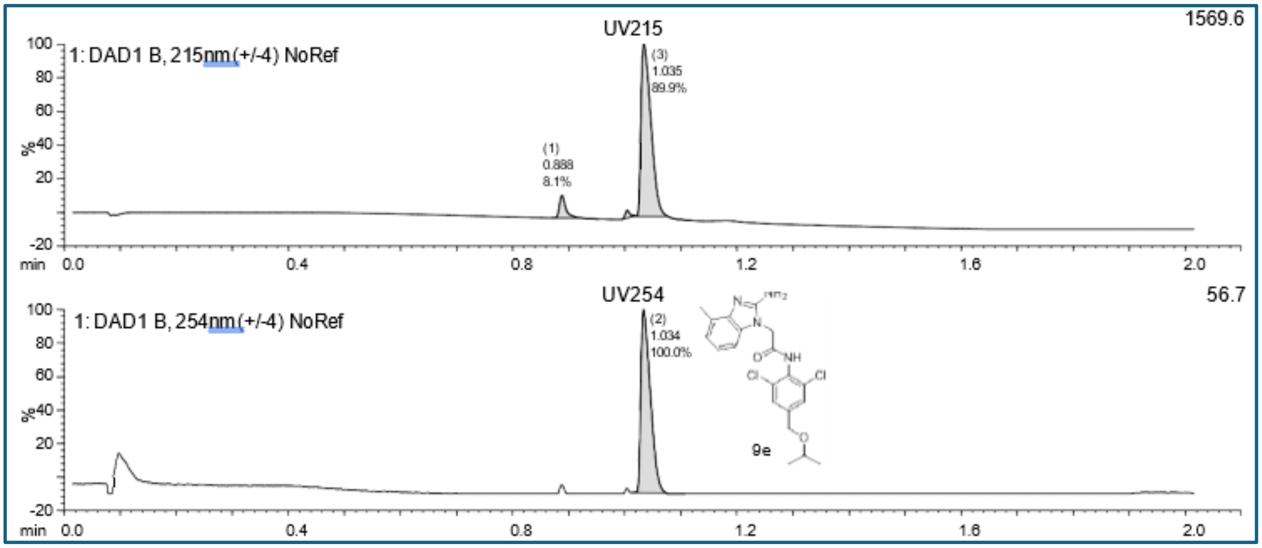
UV215
1569.6
1: DAD1 B, 215nm(+/-4) NoRef
(1) 0.888 8.1%
(3) 1.035 89.9%
UV254
56.7
1: DAD1 B, 254nm(+/-4) NoRef
(2) 1.034 100.0%
9e

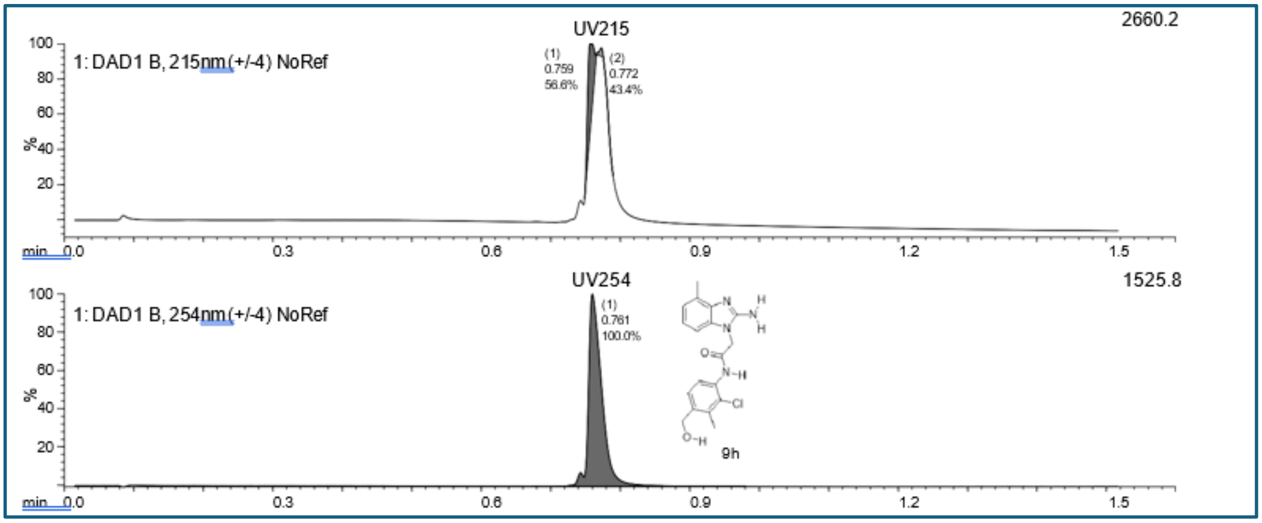
UV215
2660.2
1: DAD1 B, 215nm(+/-4) NoRef
(1)
0.759
56.6%
(2)
0.772
43.4%
%
min
0.0
0.3
0.6
0.9
1.2
1.5
UV254
1525.8
1: DAD1 B, 254nm(+/-4) NoRef
(1)
0.761
100.0%
9h

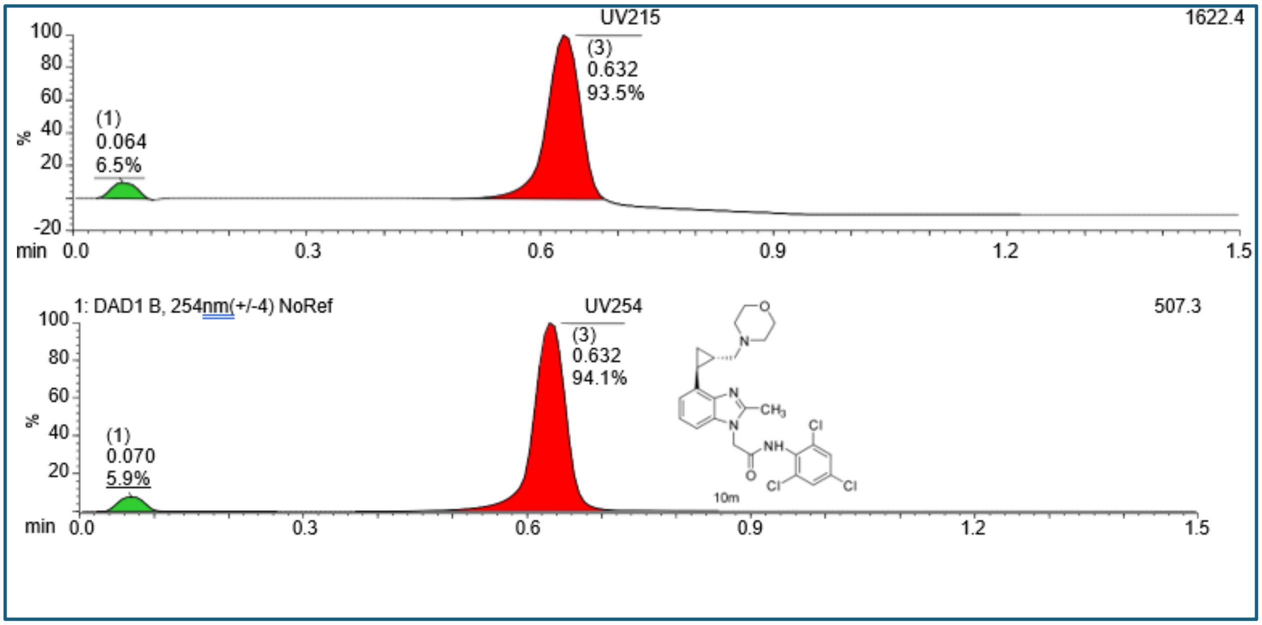
UV215
1622.4
(1)
0.064
6.5%
(3)
0.632
93.5%
%
min
0.0
0.3
0.6
0.9
1.2
1.5
1: DAD1 B, 254nm(+/-4) NoRef
UV254
507.3
(1)
0.070
5.9%
(3)
0.632
94.1%
10m

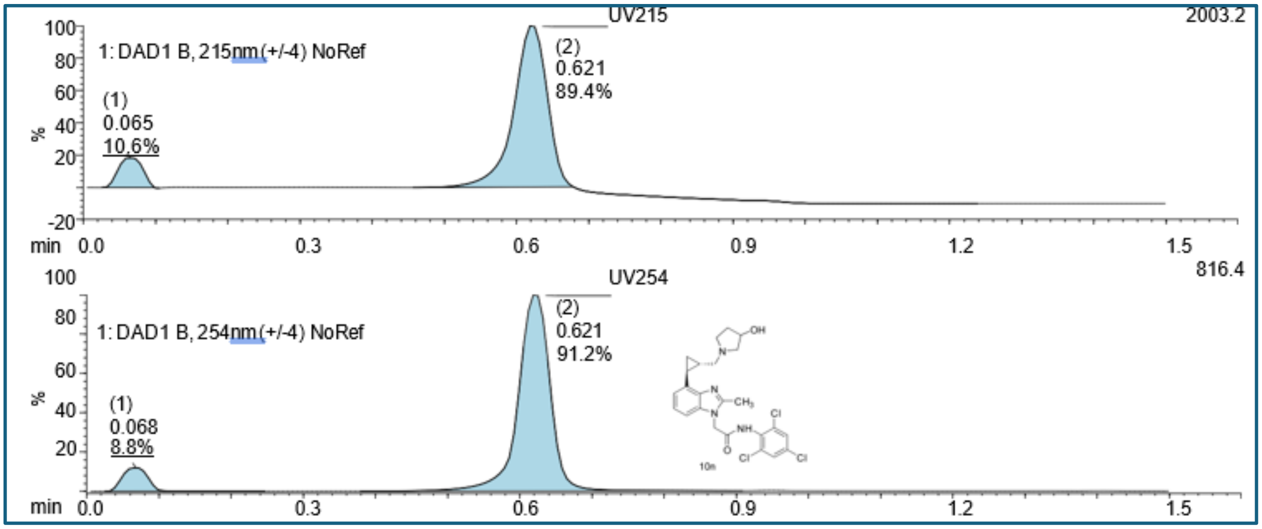
UV215
2003.2
1: DAD1 B, 215nm (+/-4) NoRef
(1)
0.065
10.6%
(2)
0.621
89.4%
UV254
816.4
1: DAD1 B, 254nm (+/-4) NoRef
(1)
0.068
8.8%
(2)
0.621
91.2%
min
%

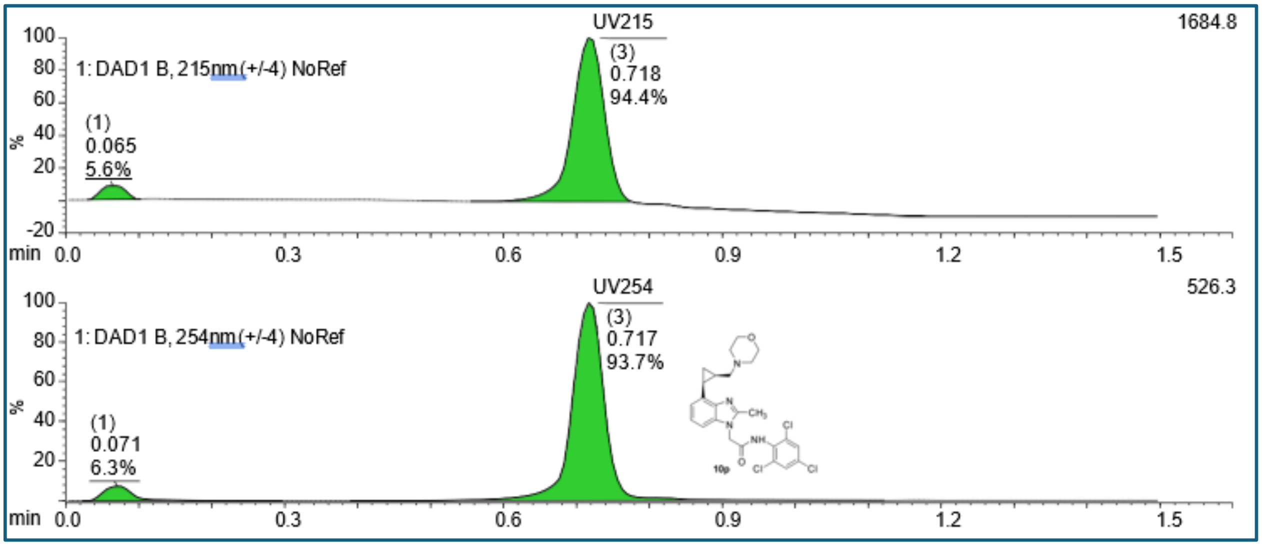
UV215
1684.8
1: DAD1 B, 215nm (+/-4) NoRef
(1)
0.065
5.6%
(3)
0.718
94.4%
UV254
526.3
1: DAD1 B, 254nm (+/-4) NoRef
(1)
0.071
6.3%
(3)
0.717
93.7%
min
%

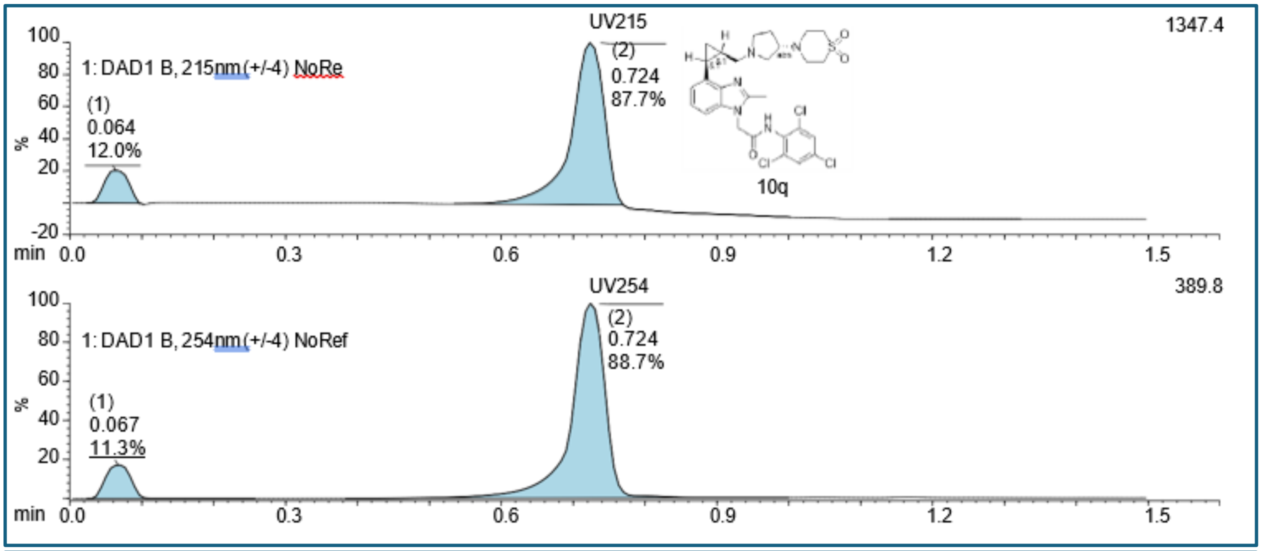
UV215
1347.4
1: DAD1 B, 215nm(+/-4) NoRe
(1) 0.064 12.0%
(2) 0.724 87.7%
10q
1: DAD1 B, 254nm(+/-4) NoRef
UV254
389.8
(1) 0.067 11.3%
(2) 0.724 88.7%
%
min 0.0 0.3 0.6 0.9 1.2 1.5

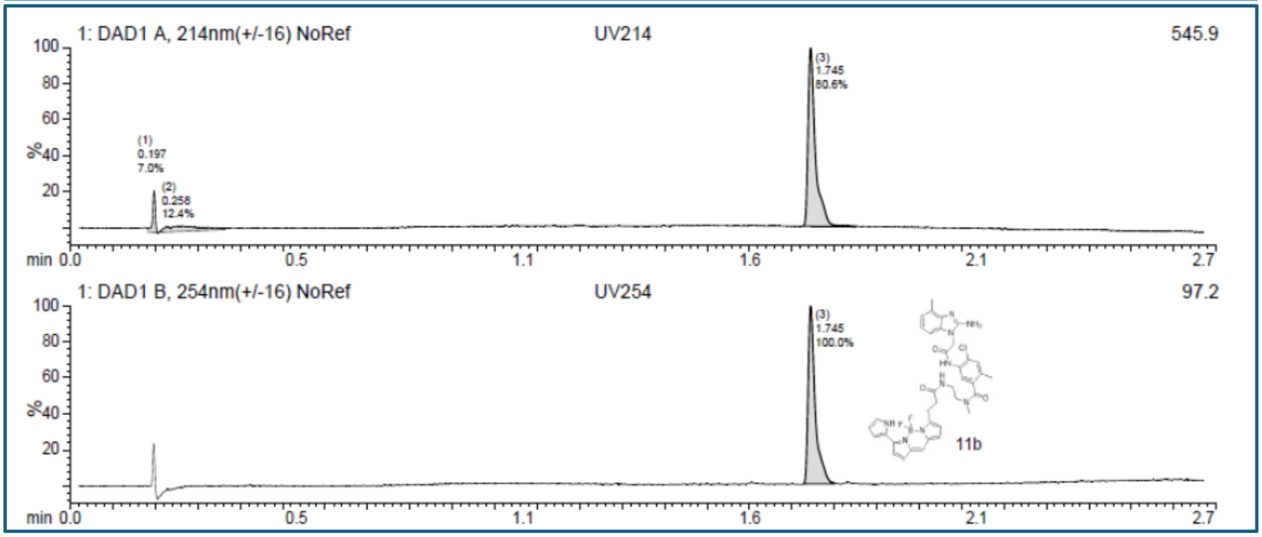
1: DAD1 A, 214nm(+/-16) NoRef
UV214
545.9
(1) 0.197 7.0%
(2) 0.258 12.4%
(3) 1.745 80.6%
1: DAD1 B, 254nm(+/-16) NoRef
UV254
97.2
(3) 1.745 100.0%
11b
%
min 0.0 0.5 1.1 1.6 2.1 2.7
